# Vaccination with mRNA-encoded nanoparticles drives early maturation of HIV bnAb precursors in humans

**DOI:** 10.1126/science.adr8382

**Published:** 2025-07-31

**Authors:** Jordan R. Willis, Madhu Prabhakaran, Michelle Muthui, Ansuya Naidoo, Troy Sincomb, Weiwei Wu, Christopher A. Cottrell, Elise Landais, Allan C. deCamp, Nahid R. Keshavarzi, Oleksandr Kalyuzhniy, Jeong Hyun Lee, Linda M. Murungi, Wilfrida A Ogonda, Nicole L. Yates, Martin M. Corcoran, Swastik Phulera, Joel Musando, Amanda Tsai, Gabrielle Lemire, Yiakon Sein, Michael Muteti, Praveen Alamuri, Jennifer A. Bohl, Drienna Holman, Sunny Himansu, Brett Leav, Caroline Reuter, Li-An Lin, Baoyu Ding, Chunla He, Walter L. Straus, Kellie J. MacPhee, Isabel Regadas, Diana V. Nyabundi, Ruth ChirChir, Omu Anzala, John N. Kimotho, Caleb Kibet, Kelli Greene, Hongmei Gao, Erica Beatman, Kiara Benson, Dominick Laddy, David M. Brown, Rhianna Bronson, Jalen Jean-Baptiste, Suprabhath Gajjala, Zahra Rikhtegaran-Tehrani, Alison Benner, Mukundhan Ramaswami, Danny Lu, Nushin Alavi, Sonya Amirzehni, Michael Kubitz, Ryan Tingle, Erik Georgeson, Nicole Phelps, Yumiko Adachi, Alessia Liguori, Claudia Flynn, Katherine McKenney, Xiaoya Zhou, David C. Owour, Sharon A. Owuor, Soo-Young Kim, Michael Duff, Ju Yeong Kim, Grace Gibson, Sabyasachi Baboo, Jolene Diedrich, Torben Schiffner, Marisa Shields, Mabela Matsoso, Jennifer Santos, Kristen Syvertsen, Allison Kennedy, Melissa Schroeter, Johan Vekemans, John R. Yates, James C. Paulson, Ollivier Hyrien, Adrian B. McDermott, Pholo Maenetje, Julien Nyombayire, Etienne Karita, Rosine Ingabire, Vinodh Edward, Vincent Muturi-Kioi, Janine Maenza, Adrienne E. Shapiro, M. Juliana McElrath, Srilatha Edupuganti, Barbara S. Taylor, David Diemert, Gabriel Ozorowski, Richard A. Koup, David Montefiori, Andrew B. Ward, Gunilla B. Karlsson Hedestam, Georgia Tomaras, Devin J. Hunt, Daniel Muema, Devin Sok, Dagna S. Laufer, Sarah F. Andrews, Eunice W. Nduati, William R. Schief

**Affiliations:** 1 Department of Immunology and Microbial Science, The Scripps Research Institute, La Jolla, CA 92037, USA; 2 IAVI Neutralizing Antibody Center, The Scripps Research Institute, La Jolla, CA 92037, USA; 3 Center for HIV/AIDS Vaccine Development, The Scripps Research Institute, La Jolla, CA 92037, USA; 4 Vaccine Research Center, National Institute of Allergy and Infectious Diseases, National Institutes of Health, Bethesda, MD 20892, USA; 5 Kenya Medical Research Institute – Wellcome Trust Research Programme, P. O. Box 230 - 80108, Kilifi, Kenya; 6 IAVI, 125 Broad Street, 9th floor, New York, NY 10004, USA; 7 Vaccine and Infectious Disease Division, Fred Hutchinson Cancer Center, Seattle, WA 98109, USA; 8 IAVI, The Address Building, 11^th^ Floor Muthangari Drive, Nairobi, Kenya 00202; 9 Center for Human Systems Immunology, Departments of Surgery, Immunology, Molecular Genetics and Microbiology, Duke University, Durham, NC 27701, USA; 10 Department of Microbiology, Tumor and Cell Biology, Karolinska Institutet, SE-171 77 Stockholm, Sweden; 11 Department of Integrative Structural and Computational Biology, The Scripps Research Institute, La Jolla, CA 92037, USA; 12 KAVI – Institute of Clinical Research, University of Nairobi, P.O Box 19676-00202, Nairobi, Kenya; 13 Moderna Inc., Cambridge, MA 02139, USA; 14 Duke University Medical Center, Durham, NC 27701, USA; 15 The Foundation for the National Institutes of Health, North Bethesda, MD 20852, USA; 16 Vaccines Research and Development, Sanofi, Swiftwater, PA 18370, USA; 17 The Aurum Institute, Rustenburg Research Centre, Rustenburg, South Africa; 18 Center For Family Health Research, KK 19 Av, No. 57, Kicukiro, Kigali, Rwanda, BP 780 Kigali, Rwanda; 19 Department of Medicine, Vanderbilt University, Nashville, TN, USA; 20 School of Health Sciences, College of Health Sciences, Westville Campus, University of KwaZulu-Natal, Durban, South Africa; 21 Department of Medicine, University of Washington, Seattle, WA 98195, USA; 22 Division of Infectious Diseases, Department of Medicine, Emory University School of Medicine, Atlanta, GA, USA; 23 Division of Infectious Diseases, Department of Medicine, University of Texas Health Science Center at San Antonio, San Antonio, Texas; 24 Department of Microbiology, Immunology and Tropical Medicine, School of Medicine and Health Sciences, The George Washington University, Washington, DC 20037, USA; 25 Department of Medicine, School of Medicine and Health Sciences, The George Washington University, Washington, DC 20037, USA

## Abstract

A leading HIV vaccine strategy requires a priming immunogen to induce broadly neutralizing antibody (bnAb) precursors, followed by a series of heterologous boosters to elicit somatic hypermutation (SHM) and produce bnAbs. In two randomized, open-label phase 1 clinical trials, IAVI-G002 in the United States and IAVI-G003 in Rwanda and South Africa, we evaluated the safety and immunogenicity of mRNA-encoded nanoparticles as priming immunogens (both trials) and first-boosting immunogens (IAVI-G002). The vaccines were generally safe and well tolerated, except 18% of IAVI-G002 participants experienced skin reactions. Priming induced bnAb precursors with substantial frequencies and SHM; heterologous boosting elicited increased SHM, affinity, and neutralization activity toward bnAb development; and elicited antibodies exhibited precise bnAb structural mimicry. The results establish clinical proof of concept that heterologous boosting can advance bnAb-precursor maturation and demonstrate bnAb priming in Africa where the HIV burden is highest.

## One-Sentence Summary

mRNA-encoded nanoparticle vaccines drive B cells toward expressing HIV bnAbs in clinical trials in North America and Africa.

Broadly neutralizing antibodies (bnAbs) to HIV can neutralize diverse viral isolates and protect against infection in non-human primates and humans (1–4). Vaccine protection against HIV is widely thought to require induction of bnAbs.

Germline-targeting vaccine design represents a promising but unproven strategy for inducing bnAbs against HIV and other highly antigenically diverse pathogens (5–14). In this strategy, a priming immunogen first activates bnAb precursor B cells sharing key genetic features for a particular class of bnAb, producing a pool of bnAb precursor-derived germinal center (GC) B cells and memory B cells. Then, a series of heterologous boost immunogens induces successive waves of germinal center activity to generate increased bnAb-like SHM, which should produce bnAbs, first in GC and memory B cells, and ultimately in plasma cells and serum (fig. S1).

## IAVI G002: Seeking clinical proof of concept for heterologous boosting to drive bnAb-precursor maturation

The IAVI G001 trial established clinical proof of concept that a germline-targeting priming immunogen can induce bnAb precursors to appreciable frequencies and with substantial productive SHM consistently across vaccine recipients (15–17). However, bnAb induction has not yet been achieved in humans. Indeed, the field has not yet conducted a clinical test of the second fundamental concept required for bnAb induction in the germline-targeting vaccine strategy, namely that a suitably designed heterologous boost can increase maturation toward bnAb development while maintaining high bnAb precursor frequencies in memory B cells consistently across vaccine recipients (fig. S1). Numerous challenges confront a heterologous boost, including: (i) whether the affinities for bnAb-precursor B cells induced by the prior immunogen are high enough to drive further maturation in GCs but not so high as to select for only minimal additional mutations or to primarily cause production of plasma cells that cannot mature further toward bnAbs (18–20); (ii) whether the boost can avoid priming of undesired bnAb-precursor competitors or boosting of such competitors induced by the prior immunogen; (iii) whether serum antibodies induced by the prior immunogen corresponding to bnAb-precursors or competitors will bind to and mask the boost immunogen, thereby inhibiting interaction of the booster with its target bnAb-precursor B cells (21–23); and (iv) whether the boosting process might be inherently limited by inefficiencies of either feeding ongoing GC reactions or returning memory B cells to GCs for continued maturation, inefficiencies that could potentially restrict the magnitude, consistency, polyclonality, and maturation of the booster response (24, 25). If a single heterologous booster cannot be designed to overcome these challenges and advance maturation toward bnAb consistently and effectively in humans, then the strategy of delivering multiple heterologous boosters in series will likely fail.

Pre-clinical experiments in stringent mouse models have shown that adjuvanted protein or mRNA-lipid nanoparticle (LNP) delivery of the priming immunogen tested in IAVI G001, eOD-GT8 60mer, followed by delivery of the heterologous booster core-g28v2 60mer, can prime and then drive additional favorable maturation of VRC01-class bnAb precursors directed to the HIV gp120 CD4-binding site (CD4bs) (26, 27). VRC01-class antibodies are defined as CD4bs-specific antibodies having heavy chain (HC) V gene alleles VH1-2*02 or *04 and any light chain (LC) complementarity determining region 3 (LCDR3) with a length of five amino acids (5, 6, 28, 29). Achieving those results in mice allowed for accelerated translation to clinical testing with an mRNA-LNP platform that has been validated as safe and highly effective through COVID-19 and other vaccines (30, 31). Indeed, mRNA-LNP technology has promise to accelerate progress through the multiple iterative clinical trials likely needed to evaluate and optimize the multi-stage heterologous boosting vaccine regimens that we hypothesize will be required to induce bnAbs.

In the IAVI G002 trial, we sought to determine if immunization of human volunteers with eOD-GT8 60mer mRNA-LNP has an acceptable safety profile and is effective for inducing VRC01-class IgG B cell responses, and if heterologous boosting with core-g28v2 60mer mRNA-LNP is safe and effective for driving VRC01-class B cell maturation further toward bnAb development. In this phase 1, open-label, randomized trial (clinicaltrials.gov NCT05001373), sixty HIV-uninfected adults in good general health were assigned to groups receiving either 100 μg eOD-GT8 60mer mRNA-LNP at weeks 0 and 8 (group 1; n=17), 100 μg eOD-GT8 60mer mRNA-LNP at week 0 followed by 100 μg core-g28v2 60mer mRNA-LNP at week 8 (group 2; n=17), 100 μg eOD-GT8 60mer mRNA-LNP at weeks 0 and 8 followed by 100 μg core-g28v2 60mer mRNA-LNP at week 16 (group 3; n=18), or 100 μg core-g28v2 60mer mRNA-LNP at week 0 (group 4; n=8) (fig. S2 and tables S1–S3).

## IAVI G003: Seeking clinical proof of concept for germline-targeting priming in Africa

Africa bears the greatest burden of HIV, including the highest incidence of new infections (32), hence a vaccine is most needed in this region. However, African populations have high genetic heterogeneity (33) that could impact the allelic composition of their naive immunoglobulin repertoires (34–36), which in turn could affect responsiveness to germline-targeting immunogens that target specific alleles of immunoglobulin genes. Indeed, eOD-GT8 60mer only induces VRC01-class bnAb precursors in individuals having at least one VH1-2*02 or VH1-2*04 allele (15, 17). In addition, African populations are exposed to multiple infectious pathogens that could modulate vaccine responsiveness by modifying the responding B cell repertoires (37) and elevating immune activation and dysregulation (38–40). A recent study showed similarity in VRC01-class bnAb precursor frequencies in unvaccinated, HIV seronegative African and North American populations (41), but no data are available on clinical testing of eOD-GT8 60mer or any other germline-targeting immunogen in the African region.

In the IAVI G003 phase 1, open-label, single-arm clinical trial, we evaluated the safety and immunogenicity of eOD-GT8 60mer mRNA-LNP in Rwanda and South Africa (clinicaltrials.gov NCT05414786). Eighteen study participants were immunized with 100 μg eOD-GT8 60mer mRNA-LNP at weeks 0 and 8 (fig. S2 and tables S4–S6). Hereafter we will refer to IAVI G001, IAVI G002, and IAVI G003 as G001, G002, and G003, respectively.

## Vaccine schedules and procedures for both trials

**Fig. 1.**
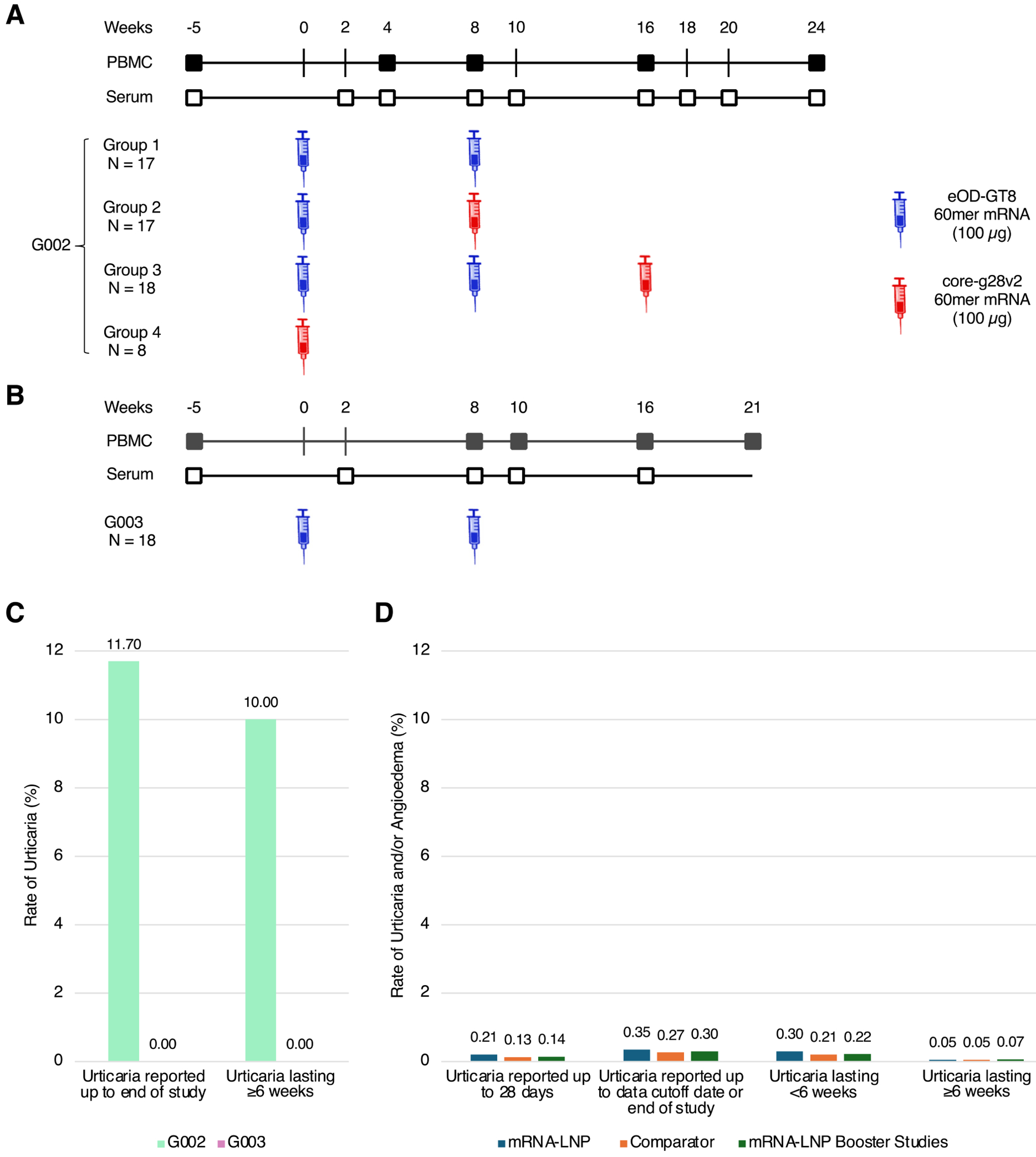
Trial schema and rates of urticaria compared to Moderna studies. (A) G002 schema. (B) G003 schema. (C) rates of urticaria in G002 and G003. (D) rates of urticaria and/or angioedema in pooled Moderna non-HIV studies described in the text. In (D), mRNA-LNP represents Moderna vaccines; Comparator represents placebo or non-mRNA vaccines included in the Moderna studies; and mRNA-LNP Booster Studies represents mRNA-1273 booster studies.

For both G002 and G003, vaccines were administered intramuscularly in the same deltoid for any one participant. The vaccination schedules and PBMC sampling timepoints essential for assessing VRC01-class responses are depicted in Fig. 1A and table S1 (G002) and Fig. 1B (G003). The full schedules of procedures for safety and immunogenicity evaluation are given in tables S7 to S10 (G002) and in tables S11 and S12 (G003).

## G002 study participation, safety, and reactogenicity

In G002, fifty-two of 60 study participants received all assigned vaccinations, and 58 of 60 completed the full 6-month study follow-up period following the final vaccination. Only 3.1% of all 1,199 visits were missed (fig. S2, table S13, and Data S1). Seven participants discontinued vaccinations but continued study visits, four due to experiencing skin adverse events (AEs) after vaccination in this trial, discussed below, and three after receiving information on skin AEs experienced by other participants (fig. S2 and table S2). Two participants were lost to follow-up during the study, one before completing assigned vaccinations, neither of whom reported skin AEs (fig. S2 and table S2).

No serious adverse events (SAEs) or adverse events of special interest (AESIs, defined by the protocol as thrombocytopenia, anaphylaxis, myocarditis/pericarditis, or a new onset or worsening of specific neurologic diseases) were reported (table S14), and no protocol-defined pause criteria were triggered. No participants acquired HIV-1 infection or developed serum positivity for HIV. Local and systemic solicited AEs were reported by 98.3% and 95% of participants, respectively, and 75% of participants reported unsolicited AEs (figs. S3 and S4, tables S14 and S15, and Data S2 to S5). AEs were generally mild or moderate (grade 1 or 2), with 11.7%, 21.7%, and 3.3% of participants experiencing solicited local, solicited systemic, or unsolicited AEs of grade 3 (severe) or higher, respectively (figs. S3 and S4, tables S14 and S15, and Data S2 to S5). Most (75%) AEs resolved within 3 days after onset (tables S16 and S17).

Eleven of 60 participants (18%) experienced a total of 14 AEs of urticaria (hives) and/or dermatographism (pressure-induced urticaria) and/or pruritus (itching), among which seven participants (11.7%) experienced a total of 7 AEs of urticaria and/or dermatographism (Fig. 1C, fig. S5 and tables S18 to S19). We refer to these AEs as “skin AEs”. All 14 skin AEs were deemed either possibly or probably related to the G002 Investigational Product (IP) by the clinical site Principal Investigator. There were no cases of angioedema or anaphylaxis (table S18 and Data S4). Most (11 of 14) skin AEs occurred after one or two doses of eOD-GT8 60mer mRNA-LNP (6 or 5 events, respectively); the remainder of the skin AEs occurred after one dose of core-g28v2 60mer mRNA-LNP preceded by one or two doses of eOD-GT8 60mer mRNA-LNP (1 or 2 events, respectively) (fig. S5 and tables S18 and S19). The skin AEs occurred at various body locations (not limited to the injection site), were delayed in onset (time to onset ranged from 7 to 40 days post vaccination; median time to onset was 19.5 days), and typically lasted for several months (duration ranged from 1 to 369 days; median duration was 8.1 weeks; 11 (79%) of 14 skin AEs lasted ≥6 weeks). Six participants (10%) experienced urticaria or dermatographism lasting for ≥6 weeks (Fig. 1C, fig. S5 and table S18). The severity of the skin AEs was generally mild to moderate (all events were grade 1 or 2 except for a single grade 3 event lasting four days). All participants with skin AEs were treated with oral antihistamines (fig. S5). The one participant who experienced grade 3 symptoms received a short course of an oral corticosteroid, with improvement of symptoms. Twelve of fourteen (86%) skin AEs resolved within six months (fig. S5 and table S18), and all skin AEs were resolved during the study.

In an exploratory, post-hoc, case-control analysis not pre-specified in the protocol, we summarized baseline participant information collected prior to vaccination, including demographic characteristics, medical history of allergy and/or atopy, and select previous or concomitant medication including COVID-19 vaccines, for participants presenting with and without skin AEs of related pruritus, urticaria, or angioedema (case definition 1) (tables S20 and S21). It is important to note that this phase 1, uncontrolled, open-label study was not designed to evaluate potential associations between baseline factors and reported skin AEs. The limited number of cases and small sample size of this trial resulted in relatively wide, overlapping confidence intervals around the point estimates (tables S20 and S21) and prevented a more thorough multivariate analysis to investigate potential predictors of the observed skin AEs. Given the small number of cases, the lack of placebo controls, the potential for reporting bias after informing participants of the observed risks, and the limited data collection on possible covariates, it is our judgment that any findings in this post-hoc analysis require further investigation and do not provide sufficient evidence to identify risk factors for skin AEs. Overall, we concluded that the vaccines in G002 had acceptable safety and tolerability profiles, except for the relatively high rate and chronicity of skin AEs, which requires further investigation and mitigation in future studies.

## G003 study participation, safety and reactogenicity

In G003, eighteen participants were enrolled in Rwanda (n=10) and South Africa (n=8). All were of black African ancestry, with median age of 33.7 years, and 8 of 18 (44.4%) were female (fig. S2 and tables S4 and S5). All enrolled participants in G003 received both vaccinations and completed all per protocol study follow-up visits (fig. S2 and table S22).

No SAEs or AESIs were reported (table S22); there were no withdrawals from study due to AEs; and no participants acquired HIV-1 or developed serum positivity for HIV. All participants (100%) experienced local solicited AEs, the most common of which were pain and axillary swelling (fig. S6 and table S22). Fourteen of 18 participants (77.8%) experienced systemic solicited AEs, the most common of which were headache, fatigue, and chills (fig. S7 and table S22). Eight of 18 participants (44.4%) experienced unsolicited AEs (table S22). Among those, pruritus, considered related to the study vaccine by the clinical site Investigator, was reported in two G003 participants (11.1%) after receipt of the second dose of the vaccine. Both episodes resolved after a duration of eight days and following treatment with an antihistamine. No urticaria or dermatographism was reported, in contrast to G002 (Fig. 1C). Of the 105 AEs experienced by all participants, 104 (99%) were mild or moderate in severity (table S22). The median duration of solicited AEs was 1 day (tables S23 and S24). The frequency, severity, and duration of AEs were similar to or lower than in North American participants in G002 group 1 who only received eOD-GT8 60mer mRNA-LNP (figs. S6 and S7, and tables S22 to S24). Overall, the eOD-GT8 60mer mRNA-LNP vaccine had an acceptable safety profile and was generally well tolerated in the 18 African G003 participants.

## Rate of urticaria in investigational Moderna vaccine studies

To gain perspective on the rate of urticaria in G002, we conducted a pooled analysis to determine the rate of urticaria and/or angioedema across other clinical studies evaluating Moderna mRNA-LNP vaccines (including those still in development) employing the same SM-102 LNP infectious disease vaccine formulation as the investigational vaccines in G002. The analysis included all Moderna-sponsored studies that were completed or had interim data submitted to a global regulatory authority as of October 4, 2024 (tables S25 and S26). Two sets of studies were pooled separately, and the rate of urticaria and/or angioedema was analyzed independently in each. The first set included studies testing investigational mRNA-LNP vaccines administered as a primary series (table S25). The total number of participants in the first set was 121,660, of which 69,105 (57%) received at least one dose of an mRNA-LNP vaccine and 52,555 (43%) received placebo or a non-mRNA comparator vaccine. The vast majority (89%) of the mRNA-LNP vaccine recipients in this set received either mRNA-1273 (COVID-19; various dose levels) in a two-dose primary series (n=27,248) or a single dose (various dose levels) of either mRNA-1345 (RSV; n=18,564) or mRNA-1010 (influenza; n=15,896). The second set of pooled studies included trials testing a single dose of mRNA-1273 or mRNA-1283, a next-generation COVID-19 vaccine (42, 43), administered as a booster. With a total of 47,239 participants, the second set included those who received a mRNA-1273 booster after previously receiving a primary series of mRNA-1273 in the first set or under emergency use authorization (n=25,674; most were also included in the first set) and participants in other trials who received either mRNA-1273 or mRNA-1283 as a booster (n=21,565) (table S26). The overall rates of urticaria or angioedema observed up to end of study in the first set were 0.35% (244/69,105) for mRNA-LNP vaccine recipients and 0.27% (140/52,555) for placebo or non-mRNA comparator vaccine recipients (Fig. 1D and tables S27 and S28). In the second set, the rate of urticaria or angioedema observed up to end of study was 0.30% (142/47,239) (Fig. 1D and table S28).

Chronic urticaria is defined as wheals (hives), angioedema (swelling), or both occurring continuously or intermittently for at least 6 weeks (44). In the first pooled set, the rate of urticaria or angioedema lasting for ≥6 weeks (chronic urticaria) was 0.05% (37/69,105) for mRNA-LNP vaccine recipients and 0.05% (27/52,555) for placebo/non-mRNA vaccine recipients, whereas urticaria or angioedema lasting <6 weeks was experienced by 0.30% (205/69,105) of mRNA-LNP vaccine recipients and 0.21% (111/52,555) of placebo/non-mRNA vaccine recipients (table S29). In the second set (mRNA-1273/mRNA-1283 booster studies), the rate of urticaria or angioedema lasting ≥6 weeks was 0.07% (33/47,239) and <6 weeks was 0.22% (102/47,239) (table S29).

The G002 participants with urticaria or dermatographism had received a median of four Moderna mRNA vaccinations prior to onset of symptoms (two COVID-19 and two G002 vaccinations) (table S18), which prompted us to probe whether the rate of urticaria might increase with the number of Moderna mRNA vaccinations. To address this question with a substantially larger dataset than in G002, we analyzed data from the NextCOVE phase 3 trial (mRNA-1283 P301 in table S26) (43) in which participants received a booster of mRNA-1273 or mRNA-1283 and their history of prior Moderna COVID-19 vaccinations was recorded (table S30). We selected this trial for further analysis from among our pooled studies, because it was the larger (n=11,417 vs. n=540) of the two studies of Moderna COVID-19 vaccines in which prior Moderna COVID-19 vaccination was recorded and where it was possible to identify participants who had received >2 Moderna COVID-19 vaccinations prior to enrollment; the smaller study meeting that criteria had no cases of urticaria. Across the entire NextCOVE study (n=11,417), the rate of urticaria or angioedema up to end of study was 0.11% (12/11,417). Among the 5,435 participants with no prior Moderna COVID-19 vaccinations, the rate was 0.15% (8/5,435). Among the 5,980 participants with at least one prior Moderna mRNA COVID-19 vaccination, all had received between 1 and 5 prior Moderna COVID-19 vaccinations, and the rate of urticaria or angioedema up to end of study was 0.04% (4/5,980). For participants having received 3, 4, or 5 Moderna COVID-19 vaccinations prior to receiving a Moderna COVID-19 booster in the NextCOVE trial, the rates of urticaria or angioedema up to end of study were 0% (0/1,660), 0.1% (1/1,049), and 0% (0/452), respectively. Therefore, in this large, phase 3 study of Moderna COVID-19 vaccines in a study population enriched for previous exposure to Moderna COVID-19 vaccination, there was no association between the number of Moderna COVID-19 vaccinations and the rate of urticaria or angioedema, at least up to six vaccinations.

We identified several caveats to comparing rates of urticaria or angioedema in the Moderna studies and rates of urticaria or dermatographism in G002, including factors that might have elevated the rates in G002 relative to those in the Moderna studies (G002 was an open-label study and included communications with participants about urticaria, dermatographism, or pruritus after cases arose, whereas the Moderna studies were double-blind, placebo-controlled studies and did not actively solicit for these specific adverse events) and factors that might have elevated the rates in the Moderna studies relative to those in G002 (the cases in G002 were all assessed as related to vaccination, whereas the Moderna cases included all events regardless of investigator assessment of relatedness). Nevertheless, we considered that making comparisons between the Moderna studies and G002 would provide important context for the observations in G002. We found that the rates of urticaria or angioedema for Moderna mRNA-LNP vaccine recipients in the first set (0.35%) or second set (0.30%) were >33-fold lower than in G002 (11.7%) (Fig. 1C and D). We also found that the overall rates of chronic urticaria or angioedema for mRNA-LNP vaccine recipients in the first set (0.05%) or second set (0.07%) were >140-fold lower than the rate of chronic urticaria observed in G002 (10%) (Fig. 1C and D).

## Serum antibody binding responses

All G002 and G003 recipients of eOD-GT8 60mer mRNA-LNP produced serum IgG responses to the eOD-GT8 60mer, eOD-GT8 monomer and eOD-GT8 CD4bs after the first vaccination (fig. S8 and tables S31 and S32). The response magnitudes to eOD-GT8 60mer and monomer increased with the second vaccination, whereas the eOD-GT8 CD4bs responses declined by approximately 50%, a decline not seen in G001 (tables S33 and S34). For comparison to G001, data from G001 low (20 μg) and high (100 μg) dose groups, which had similar serum IgG responses (15), were pooled. The G002 response magnitudes to eOD-GT8 60mer, monomer, and CD4bs were significantly (*P*-values <0.05 and false discovery rate [FDR] *Q*-values <0.2 (45, 46)) higher than those in G001 at nearly all post-vaccination timepoints measured for both trials by the same assay in the same laboratory at Duke University (fig. S8 and table S32). (From here on, all claims of significance will have P<0.05 and Q<0.2, and P and Q values for all statistical tests will be provided in supplementary tables). G003 serum IgG responses to eOD-GT8 60mer and monomer, measured in a laboratory at the KAVI Institute of Clinical Research using an assay that had good concordance with the Duke assay for these antigens (fig. S9), were also significantly higher than in G001 at weeks 2, 8, and 10 (fig. S8 and table S32). G003 response magnitudes to eOD-GT8 60mer and monomer were significantly lower than in G002 at weeks 2, 8, 10, and 16 (fig. S8 and table S32). Prevaccination reactivity to Lumazine Synthase (LS), the interior nanoparticle in both eOD-GT8 60mer and core-g28v2 60mer, was detected in nearly all G002 and G003 participants (fig. S8). Reactivity to LS increased with vaccination in both G002 and G003 (fig. S8I and J, tables S33 and S34), as was found in G001 (15). We concluded that eOD-GT8 60mer mRNA-LNP consistently induced class-switched, antigen-specific and eOD-GT8 CD4bs-specific serum IgG responses in North American and African individuals.

All G002 recipients of core-g28v2 60mer mRNA-LNP (in groups 2, 3, and 4) produced serum IgG responses to the core-g28v2 60mer, core-g28v2 monomer, and core-g28v2 CD4bs (fig. S10 and tables S35 and S36). Response rates and magnitudes to core-g28v2 monomer and core-g28v2 CD4bs at weeks 16 through 24 were significantly higher for group 2 (recipients of eOD→core) than for recipients of two vaccinations with eOD-GT8 60mer mRNA-LNP (eOD→eOD, participants pooled from groups 1 and 3) (fig. S10 and table S36), demonstrating that the heterologous boost was immunogenic in group 2. Response rates and magnitudes to core-g28v2 monomer and core-g28v2 CD4bs at weeks 20 and 24 were significantly higher for group 3 (recipients of eOD→eOD→core) than for recipients of eOD→eOD (fig. S10 and table S36), demonstrating that the group 3 heterologous boost was immunogenic. We concluded that the core-g28v2 60mer mRNA-LNP heterologous boost in G002 was highly immunogenic and consistently induced class-switched, antigen-specific and core-g28v2 CD4bs-specific serum IgG responses.

## bnAb precursor responses to the priming immunogen

To determine if eOD-GT8 60mer mRNA-LNP could induce VRC01-class IgG responses in G002 and G003, we established analysis workflows (different for each trial) to determine and interpret B cell receptor (BCR) sequences for eOD-GT8 CD4bs-specific B cells using bulk sorting of epitope-specific B cells, droplet-based single-cell RNAseq to obtain paired heavy and light chain BCR sequences, and bioinformatic analysis (figs. S11 to S19 and tables S37 to S39). In both G002 and G003, we analyzed IgD^−^ memory B cells from peripheral blood mononuclear cell (PBMC) samples taken before and at multiple timepoints after eOD-GT8 60mer immunization (Fig. 1A–B, figs. S20 and S21). In G002 we also analyzed IgD^+^ B cells at baseline. Complete data from sorting and sequencing, including sequences for the heavy and light chain of at least one eOD-GT8 CD4bs-specific BCR per sample, was obtained for 92% (192/208) of planned G002 samples, whether considering BCRs of all isotypes (table S41) or restricting to IgG (table S42). For G003, complete data was obtained for 86% (77/90) or 82% (74/90) of planned samples, considering all isotypes (table S43) or IgG only (table S44), respectively. In G002, analysis of responses to eOD-GT8 60mer mRNA-LNP was pooled at each timepoint from all participants having received the same number of eOD-GT8 60mer vaccinations prior to that timepoint, meaning that analysis at weeks −5, 4, and 8 included data from all participants in groups 1 to 3; week 16 analysis included data from all participants in groups 1 and 3; and week 24 analysis was from group 1 only (Fig. 1A). In the narrative below, inter-trial statistical comparisons were restricted to comparisons between G001 and G002 for the frequency and SHM of VRC01-class IgG B cells, as described in the methods section *Cross-calibration of B cell workflows from G001, G002, and G003*.

**Fig. 2.**
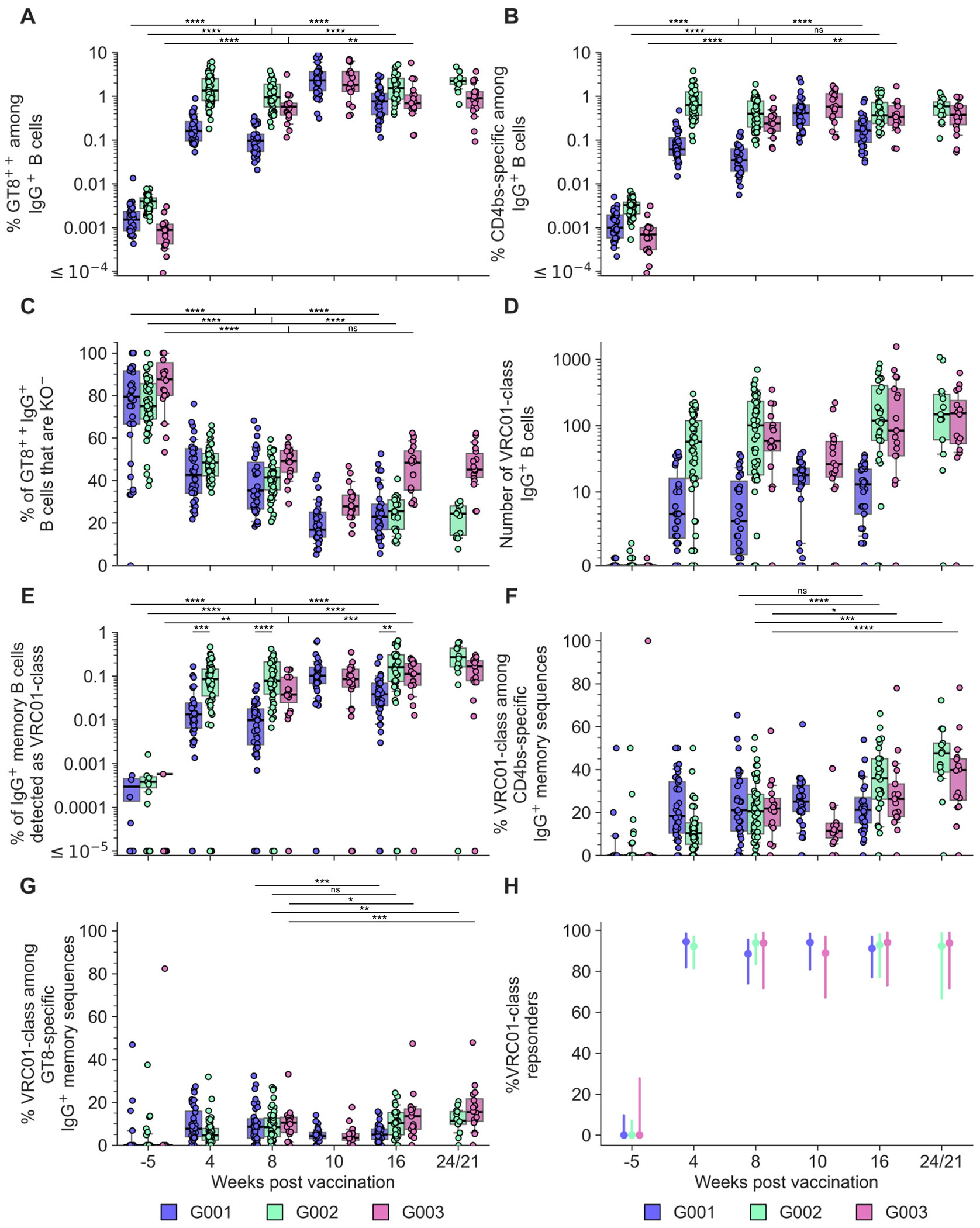
B cell frequency analyses related to priming by eOD-GT8 60mer mRNA-LNP in G002 and G003, with comparisons to adjuvanted protein vaccination in G001. **(A** and **B)** Percentages of IgG memory B cells that are eOD-GT8–specific (GT8^++^) (A) or eOD-GT8 CD4bs-specific (B). **(C)** Percentage of GT8^++^ IgG memory B cells that are CD4bs-specific (KO^−^). **(D)** Number of VRC01-class IgG B cells detected. At weeks 4 and 16, G001 and G002 analyzed 100 million PBMCs; at week 16, G003 analyzed 200 million. **(E** to **G)** Percentage of VRC01-class IgG B cells among IgG memory B cells (E), CD4bs-specific IgG B cells (F), or GT8^++^ IgG B cells (G). **(H)** Percentage of participants with a VRC01-class IgG B cell response (defined as having a VRC01-class IgG B cell frequency higher than at baseline), at each timepoint. In (A) through (G), circles represent participants, thick lines indicate median values, boxes indicate 25% and 75% quantiles, and whiskers approximate 90% and 10% quantiles. In (E), medians and quantiles were computed over nonzero values only because nonresponders were accounted for in (H). In (H), circles indicate median values, and lines indicate 95% CIs computed using the Wilson score method. Data from G001 are from both 100 μg and 20 μg dose groups combined. Comparisons over time within each trial in (A), (B), (C), (E), and (F), were done using the Wilcoxon signed-rank test for paired data (tables S46 and S47). Testing between G001 and G002 in (E) was done using a B cell workflow-adjusted bootstrap (table S48). Significant differences had FDR Q-value ≤0.2 and P-values of <0.05 (*), <0.01 (**), <0.001 (***), or <0.0001 (****); ns indicated not significant. All B cells in this figure were sorted with eOD probes. Source data can be found in Data S10.

All G002 and G003 participants vaccinated with eOD-GT8 60mer mRNA-LNP produced eOD-GT8-specific and eOD-GT8 CD4bs-specific IgG B cells at week 8 after the first vaccination with frequencies significantly above baseline (Fig. 2, A and B, and table S45). These responses exhibited high magnitudes; for example, median week 8 frequencies of eOD-GT8 CD4bs-specific IgG B cells in G002 and G003 were 0.40% (>120-fold above baseline) and 0.24% (>340-fold above baseline), respectively (Fig. 2, A and B, and table S46). After the second vaccination at week 8, frequencies of eOD-GT8-specific IgG B cells in G002 and G003 increased further at week 16, similar to findings in G001 (15) (Fig. 2, A and B, and table S47). Frequencies of eOD-GT8 CD4bs-specific IgG B cells also increased significantly over time after week 8 in G003 but in G002 were relatively constant (Fig. 2, A and B, and table S47). The fractions of eOD-GT8-specific B cells that were CD4bs-specific in G002 and G003 were relatively high (median values of 75% and 88%, respectively) at baseline but declined significantly after vaccination (e.g. week 8 median values of 41% and 49%, respectively), reflecting the induction of off-target (non-CD4bs) B cells that was also seen in G001 (Fig. 2C and tables S46 and S47).

VRC01-class IgG B cells were detected after the first vaccination in substantial numbers (e.g. week 8 median numbers per participant were 101 in G002 and 59 in G003; Fig. 2D) and at substantial frequencies (e.g. week 8 median frequencies per participant were 0.08% in G002 and 0.04% in G003; Fig. 2E). VRC01-class IgG B cell median frequencies increased significantly to 0.15% at week 21 (G003) and 0.25% at week 24 (G002 group 1), suggesting robust germinal center activity for many weeks following the last vaccination at week 8 (Fig. 2E and table S47). The frequencies of VRC01-class IgG B cells were significantly higher for G002 compared to G001 at all postvaccination timepoints tested in both trials (weeks 4, 8, and 16, where geometric mean frequencies were higher by 3.2-, 6.1-, and 2.6-fold, respectively, after accounting for methodological differences) (Fig. 2E and table S48). CD4bs-specific and VRC01-class responses were dominated by IgG in G002 and G003 (figs. S22 and S23), as was true in G001 (15), and we further found in G002 and G003 that the IgG1 subclass was dominant (figs. S22 and S23). The VRC01-class fractions of CD4bs-specific and eOD-GT8-specific BCRs following eOD-GT8 60mer vaccination were relatively low at week 8 in all three trials (e.g. median values of 20-22% for CD4bs-specific and 8.5-10.5% for GT8-specific) but increased significantly over time in G002 and G003 (e.g. median values for CD4bs-specific were 48% for G002 at week 24 and 40% for G003 at week 21; Fig. 2, F and G, and table S47), demonstrating VRC01-class frequency gains against CD4bs-specific competitors, which was not observed in G001 (15). The VRC01-class response rates among G002 and G003 participants were high, with 49/52 G002 responders and 17/18 G003 responders across all postvaccination timepoints (Fig. 2H), and with non-responders (three in G002 and one in G003) all associated with lack of permissive VH1-2 alleles (fig. S24 and tables S49 to S52), as was encountered in G001 (15, 17).

**Fig. 3.**
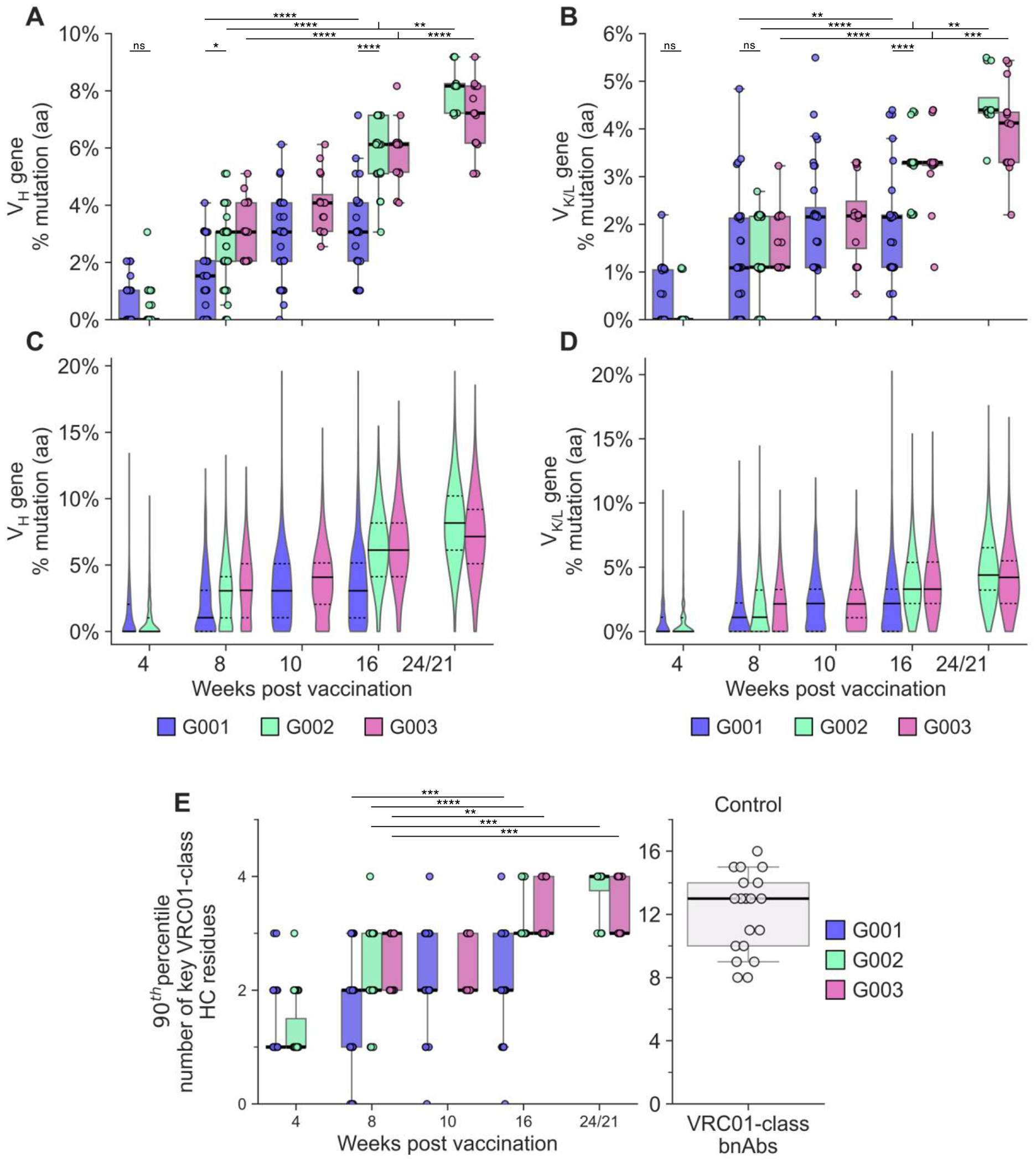
Mutation analyses in VRC01-class responses to eOD-GT8 60mer mRNA-LNP in G002 and G003, with comparisons to adjuvanted protein vaccination in G001. **(A** and **B)** VRC01-class BCR percent amino acid (aa) mutation in V_H_ (A) and V_K/L_ (B), with circles representing the median per participant per timepoint. **(C** and **D)** VRC01-class BCR percent amino acid (aa) mutation in V_H_ (C) and V_K/L_ (D), with violin plots representing the distribution of all BCRs per group per timepoint. **(E)** Number of key VRC01-class HC residues, with circles indicating the 90% quantile per participant per timepoint (left), and for VRC01-class bnAbs (right, different y-axis scale), with circles denoting bnAbs. In (A) and (B), thick lines indicate median values, box plots show 25% and 75% quantiles, and whiskers approximate 90% and 10% quantiles. In (C) and (D), solid lines are medians, and dashed lines show 25% and 75% quantiles. Data from G001 includes both dose groups. Comparisons over time within one trial in (A), (B), and (E), were tested using the Wilcoxon signed-rank test for paired data (table S55). Testing between G001 and G002 in (A) and (B) was done using a B cell workflow-adjusted bootstrap (table S53). Significant differences had FDR Q-value ≤0.2 and P-values of <0.05 (*), <0.01 (**), <0.001 (***), or <0.0001 (****); ns indicated not significant. All B cells in this figure were sorted as eOD-GT8 CD4bs-specific. Source data can be found in Data S10.

SHM in G002 and G003 VRC01-class responses to eOD-GT8 60mer mRNA-LNP was remarkably high, with median per participant amino acid mutation in VH1-2 reaching 3% at week 8 and 6% at week 16 in both trials, values twice as high as those in G001 (Fig. 3, A to D, fig. S25, and tables S53 to S56). Median SHM in VH1-2 increased significantly from week 16 to week 21 (7% in G003) and week 24 (8% in G002), consistent with long-lived GC activity (Fig. 3A and table S55). The 90^th^ percentile number of key VRC01-class heavy chain residues per participant, representing the best 20% of BCRs per participant (15, 26), increased significantly over time, with the median value increasing from 2.0 to 4.0 between weeks 8 and 24 in G002, and the range increasing from [2.0, 3.0] at week 8 to [3.0, 4.0] at week 21 in G003 (Fig. 3E and table S55). Additional sequence-based metrics of VRC01-class BCRs, including light chain V-gene usage (fig. S26A), LCDR3 sequences (fig. S26, B to E), and Trp_103-5_ usage (fig. S26F), showed that substantial fractions of vaccine-induced VRC01-class BCRs in all three trials shared important features with known VRC01-class bnAbs (15). VRC01-class antibody affinities for eOD-GT8 increased with vaccination in all three trials (figs. S27 and S28A) and displayed major affinity superiority over CD4bs-specific, non-VRC01-class antibodies (e.g. >6000-fold higher median affinities at week 16 in both G002 and G003) (fig. S28B). BCR clustering analysis demonstrated that the high degree of polyclonality in VRC01-class sequences seen in G001 (15), an essential feature of germline-targeting priming, was also seen in G002 and G003 (figs. S29 and S30). We concluded that 100 μg of eOD-GT8 60mer mRNA-LNP induced VRC01-class responses of high magnitude and quality in North American and African individuals in G002 and G003, respectively.

## bnAb precursor responses to the heterologous boost immunogen

**Fig. 4.**
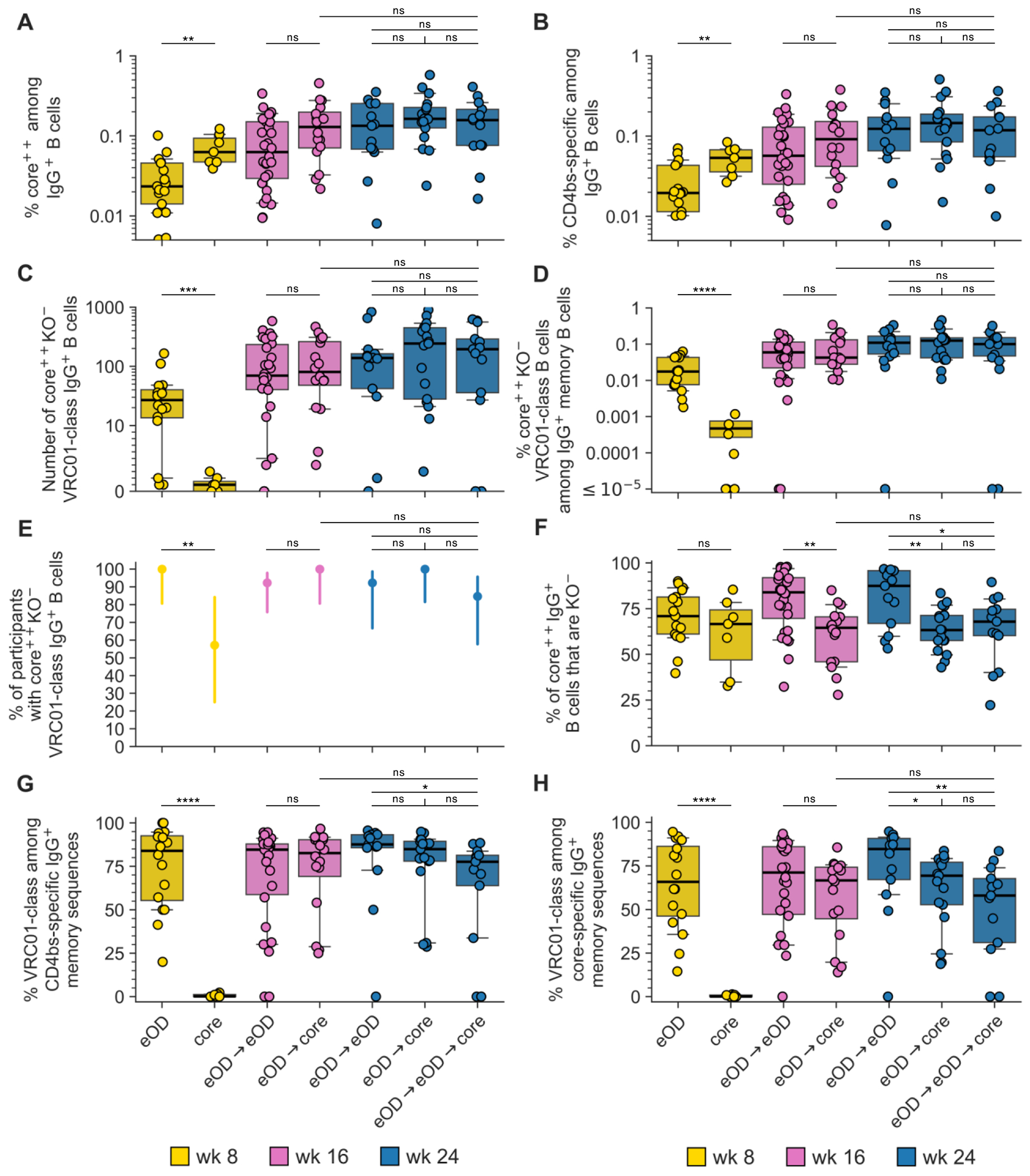
B cell frequency analyses related to boosting by core-g28v2 60mer mRNA-LNP. **(A** and **B)** Percentages of IgG memory B cells that are core-g28v2–specific (core^++^) (A) or core-g28v2 CD4bs-specific (B). **(C)** Number of core^++^KO^−^ VRC01-class IgG B cells detected. **(D)** Percentage of core^++^KO^−^ VRC01-class B cells among IgG memory B cells. **(E)** Percentage of participants with core^++^KO^−^ VRC01-class IgG B cells detected at each timepoint. **(F)** Percentage of core^++^ IgG memory B cells that are CD4bs-specific (KO^−^). **(G** and **H)** Percentage of VRC01-class B cells among core^++^KO^−^ IgG B cells (G), or core^++^ IgG B cells (H). In (A) through (D) and (F) through (H), circles represent participants, thick lines indicate median values, boxes indicate 25% and 75% quantiles, and whiskers approximate 90% and 10% quantiles. In (D), medians and quantiles were computed over nonzero values only because nonresponders were accounted for in (E). In (E), circles indicate median values, and lines indicate 95% CIs computed using the Wilson score method. Statistical testing between groups was done using the Wilcoxon rank-sum test for continuous outcomes [all panels except (E)] and Barnard’s exact test for the binary outcome in panel (E) (table S57). Significant differences had FDR Q-value ≤0.2 and P-values of <0.05 (*), <0.01 (**), <0.001 (***), or <0.0001 (****); ns indicated not significant. All B cells in this figure were sorted with core-g28v2 probes. Source data can be found in Data S10.

To determine if the core-g28v2 60mer mRNA-LNP boost in G002 could increase maturation of VRC01-class IgG B cell responses primed by eOD-GT8 60mer mRNA-LNP, we established an analysis workflow to determine and interpret BCR sequences for core-g28v2 CD4bs-specific B cells analogous to our workflow for eOD-GT8 (figs. S11 to S14, S17A and S18A, and tables S37 and S38). We analyzed core-g28v2 CD4bs-specific IgD^−^ B cells from PBMC samples collected after one immunization with eOD-GT8 60mer mRNA-LNP (group 2 at week 8), two immunizations with eOD-GT8 60mer mRNA-LNP (groups 1 and 3 at week 16, and group 1 at 24), before and after core-g28v2 60mer mRNA-LNP priming at weeks −5, 4, and 8 in group 4, and after core-g28v2 60mer mRNA-LNP boosting in group 2 (weeks 16 and 24) or group 3 (week 24) (Fig. 4, figs. S31 and S32, and table S37). Complete data from sorting and sequencing, including sequences for the heavy and light chain of at least one core-g28v2 CD4bs-specific BCR per sample, was obtained for 84% (122/145) of planned samples, for BCRs of all isotypes (table S41) and for IgG BCRs only (table S42).

A single priming immunization with core-g28v2 60mer mRNA-LNP (in group 4) induced substantial frequencies of core-g28v2-specific and core-g28v2 CD4bs-specific IgG B cells at weeks 4 and 8 (Fig. 4, A and B, and fig. S32, A and B). However, among those CD4bs-specific B cells, we detected VRC01-class B cells from only 2 of 8 participants at week 4, and 4 of 7 participants with sequencing data at week 8 (samples were not available for sorting for one participant at week 8) (Fig. 4C, fig. S32C, and tables S49 and S50). Four of eight participants failed to produce a detectable VRC01-class response at week 4 or 8, and VH1-2 genotype could only explain one of those VRC01-class non-responders (tables S49 and S50). For the responders, the median frequency of core-g28v2 CD4bs-specific VRC01-class IgG B cells at week 8 was 0.00047%, which was 37- or 166-fold lower than the median frequencies of VRC01-class IgG B cells that were core-g28v2 CD4bs-specific (Fig. 4D) or eOD-GT8 CD4bs-specific (Fig. 2E), respectively, after a single immunization with eOD-GT8 60mer mRNA-LNP. Hence, a single priming immunization with core-g28v2 60mer mRNA-LNP did not induce a substantial VRC01-class response.

To evaluate core-g28v2 60mer mRNA-LNP as a boost immunogen, we compared the results from a core-g28v2 60mer mRNA-LNP boost following an eOD-GT8 60mer mRNA-LNP prime (eOD→core; samples available from group 2 at weeks 16 [n=16] and 24 [n=17]) to the results from two vaccinations with eOD-GT8 60mer mRNA-LNP (eOD→eOD; samples available from groups 1 and 3 at week 16 [n=26] and group 1 at week 24 [n=13]) (tables S41 and S42). The eOD→core and eOD→eOD regimens induced similar frequencies of core-g28v2-specific (Fig. 4A), core-g28v2 CD4bs-specific (Fig. 4, B), and core-g28v2 CD4bs-specific, VRC01-class IgG B cells (Fig. 4, C and D), with similar VRC01-class response rates among participants (Fig. 4E), at weeks 16 and 24 (table S57). These responses were quite robust; for example, the eOD→core regimen produced a 100% response rate for core-g28v2 CD4bs-specific, VRC01-class responses at weeks 16 and 24, with median VRC01-class frequencies of 0.043% (1 in 2330) and 0.126% (1 in 794), respectively. Thus, the core-g28v2 60mer boost performed similarly to the eOD-GT8 60mer boost in terms of the magnitude of the VRC01-class response, despite the fact that core-g28v2 had a CD4bs epitope substantially closer to wild-type Env than eOD-GT8 (26), had substantially (>450-fold) lower affinity compared to eOD-GT8 for the week 8 post-eOD-GT8 60mer VRC01-class memory B cells targeted by the second vaccination [median *K*_D_ values were 8 nM for eOD-GT8 and ≥100 μM for core-g28v2; among the 26% binders to core-g28v2, the geomean *K*_D_ was 3.6 μM (fig. S33)], and had little capacity to prime naïve VRC01-class responses (discussed above).

We also compared the results from a core-g28v2 60mer mRNA-LNP boost following two immunizations with eOD-GT8 60mer mRNA-LNP (eOD→eOD→core; n=15 participants from group 3 with available samples [tables S41 and S42]) to the results from the eOD→eOD and eOD→core regimens at week 24. We found that eOD→core produced similar VRC01-class frequencies and response rates compared to eOD→eOD→core or eOD→eOD (Fig. 4, D and E, and table S57). Furthermore, comparing responses at 8 weeks post-core vaccination, we found that the responses to eOD→core at week 16 were statistically indistinguishable from the responses to eOD→eOD→core at week 24, as judged by VRC01-class frequencies (Fig. 4D) or response rates (Fig. 4E) or any other measure in Figure 4 (table S57). Thus, these data identified no advantage to the extra vaccination in the eOD→eOD→core regimen compared to the eOD→core regimen. The VRC01-class responses to all prime-boost regimens were dominated by the IgG isotype and the IgG1 subclass (fig. S34).

## Immunofocusing to bnAb precursor responses

The eOD→core and eOD→eOD→core regimens both induced significantly higher frequencies of off-target, non-CD4bs responses compared to eOD→eOD as measured by core-g28v2 sorting at weeks 16 and 24 (Fig. 4F and table S57). Nevertheless, the eOD→core regimen maintained high immunofocusing on VRC01-class responses when assayed by core-g28v2 sorting, with median VRC01-class responses at week 16 comprising 83% of core-g28v2 CD4bs-specific (Fig. 4G) and 67% of core-g28v2-specific responses (Fig. 4H), similar to values for the eOD→eOD regimen (table S57). These VRC01-class percentages were significantly higher than what we measured for the eOD→eOD regimen with eOD-GT8 sorting, where the median VRC01-class responses at week 16 comprised 37% of eOD-GT8 CD4bs-specific responses (Fig. 2F) and 11% of eOD-GT8-specific responses (Fig. 2G) (table S58). The fact that eOD→eOD responses assayed by core-g28v2 sorts were dominated by VRC01-class responses demonstrated that core-g28v2 preferentially binds to VRC01-class responses induced by eOD-GT8 60mer rather than non-VRC01-class responses, as intended by design (26). However, our finding that eOD→core responses assayed by core-g28v2 sorts were dominated by VRC01-class responses to a similar degree was more surprising, as this indicated not only that the core-g28v2 60mer mRNA-LNP immunization boosted VRC01-class responses primed by eOD-GT8 60mer mRNA-LNP but also that it only induced relatively weak competing non-VRC01-class responses to core-g28v2 CD4bs and non-CD4bs epitopes. Such competing responses could have been induced either by boosting non-VRC01-class responses primed by eOD-GT8 60mer mRNA-LNP or by priming core-g28v2-specific non-VRC01-class responses. These results provide confirmation in humans for a critical aspect of the germline-targeting sequential vaccination strategy, namely that a suitably designed heterologous boost immunogen can selectively expand bnAb precursors while minimizing both the boosting of pre-existing competitors and the induction of naive boost-immunogen-specific competing responses.

## Polyclonality of bnAb precursors following heterologous boost

A successful heterologous boost should drive maturation of highly polyclonal bnAb-related responses, to advance as many maturation trajectories as possible toward bnAb development. Hierarchical clustering of core-g28v2-sorted VRC01-class BCR sequences showed high polyclonality in responses to all heterologous prime-boost regimens, with most participants at weeks 16 and 24 having >70% of their VRC01-class BCRs correspond to unique clones or lineages (fig. S35A). The eOD→eOD and eOD→core regimens had similar polyclonality at weeks 16 and 24, whereas the eOD→eOD→core regimen had lower polyclonality compared to eOD→eOD at week 24 (fig. 35A and table S59). Polyclonality was significantly lower at week 16 for eOD→eOD or eOD→core compared to week 8 for eOD, which was associated with an increase in the median number of unique clones per participant over time, from *n*=27 at week 8 for eOD to *n*=72 at week 16 for eOD→eOD or *n*=81 at week 16 for eOD→core (fig. 35B and table S60).

The high polyclonality indicated that our PBMC sorting substantially undersampled the VRC01-class IgG B cell populations in these participants. Indeed, clustering of all VRC01-class IgG BCRs sorted by either eOD-GT8 or core-g28v2 from all timepoints revealed that >80% of clones were singletons and >96% of clones had size ≤3 (fig. S36). Hence, analyses of clonal expansion was not possible for most of the data. However, clustering did reveal several large clones (24 clones with ≥ 20 sequences each), which provided an opportunity to assess SHM within B cell lineages over time. For further analysis, we selected the three largest lineages: two from the eOD→core regimen containing 99 and 46 sequences, respectively (fig. S37), and one from the eOD→eOD regimen containing 83 sequences (fig. S38). All three lineages exhibited measurable evolution over time (figs. S37, C and D, and S38B), accompanied by time-dependent increases in affinity for core-g28v2 (figs. S37, E and F, and S38C). These analyses provided examples of VRC01-class B cell lineage development over time induced by vaccination.

## bnAb precursor maturation via heterologous boost

**Fig. 5.**
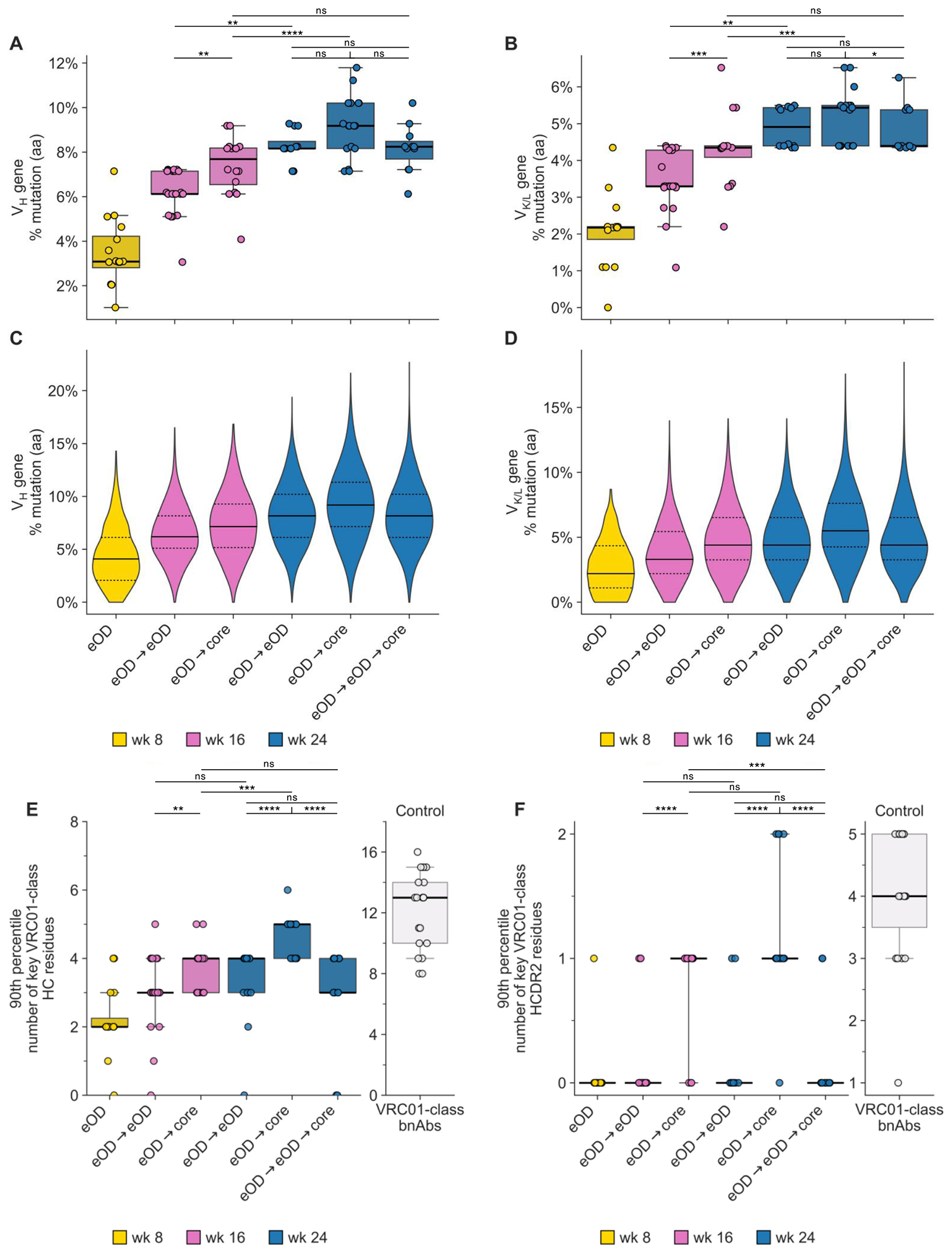
Mutation analyses in VRC01-class responses to prime-boost regimens in G002. **(A** and **B)** VRC01-class BCR V_H_ (A) and V_K/L_ (B) percent amino acid (aa) mutation, with circles representing the median per participant per timepoint. **(C** and **D)** VRC01-class BCR V_H_ (C) and V_K/L_ (D) percent amino acid (aa) mutation, with violin plots representing the distribution of all BCRs per group per timepoint. **(E)** Number of key VRC01-class HC residues, with circles indicating the 90% quantile per participant at each timepoint (left), and for VRC01-class bnAbs (right, different y-axis scale), with symbols denoting bnAbs. **(F)** Number of key VRC01-class HCDR2 residues, with circles indicating the 90% quantile per participant at each timepoint (left), and for VRC01-class bnAbs (right, different y-axis scale), with symbols denoting bnAbs. In (A), (B), (E), and (F), thick lines indicate median values, box plots show 25% and 75% quantiles, and whiskers approximate 90% and 10% quantiles. In (C) and (D), solid lines indicate median values, and dashed lines show 25% and 75% quantiles. Testing between groups was done using a Wilcoxon signed-rank test for continuous outcomes, and testing within a group over time was done using a Wilcoxon signed-rank test for continuous outcomes and paired data (table S61). Significant differences had FDR Q-value ≤0.2 and P-values of <0.05 (*), <0.01 (**), <0.001 (***), or <0.0001 (****); ns indicated not significant. All B cells in this figure were sorted as core-g28v2 CD4bs-specific. Source data can be found in Data S10.

A key goal of the heterologous boost strategy is to select for increased SHM toward bnAb development. Compared to the eOD→eOD homologous boost, the eOD→core regimen induced significantly higher amino acid mutation levels in V_H_ and V_K/L_ genes of VRC01-class BCRs at week 16 (Fig. 5, A to D, and table S61). SHM increased significantly from week 16 to week 24 for both eOD→eOD and eOD→core (Fig. 5, A and B, and table S61), reflecting ongoing GC activity as was evident in the frequency data. At week 24, SHM in V_H_ and V_K/L_ genes of VRC01-class BCRs was similar for all three regimens, except that V_K/L_ mutation was higher for eOD→core than for eOD→eOD→core (Fig. 5, A and B, and table S61). In terms of the quality of the selected mutations, eOD→core was superior to both eOD→eOD and eOD→eOD→core, eliciting populations of VRC01-class BCRs at weeks 16 and 24 with significantly higher values for the 90^th^ percentile number of key VRC01-class HC residues and the 90^th^ percentile number of key VRC01-class HCDR2 residues, important metrics for VRC01-class maturation (15, 26, 47) (Fig. 5, E and F, fig. S39, and table S61). Comparing responses at 8 weeks after a core boost, the degree (Fig. 5, A and B) and quality (Fig. 5, E and F) of SHM elicited by eOD→core at week 16 was similar to that of eOD→eOD→core at week 24, except that eOD→core elicited a higher 90^th^ percentile number of key VRC01-class HCDR2 residues (table S61). Regarding other VRC01-class quality metrics, all prime-boost regimens produced VRC01-class responses predominantly using light chain V genes used by bnAbs (fig. S40A) and had similar percentages of bnAb-matching LCDR3s (fig. S40B), but the eOD→core regimen produced higher usage of Q or E at LCDR3 position 96 compared to eOD→eOD at week 16 or eOD→eOD→core at week 24 (figs. S40C and S40D), and higher Trp_103-5_ usage in the HCDR3 compared to eOD→eOD at week 16 or either of the other regimens at week 24 (fig. S40E) (table S62). Thus, the SHM and key mutations data indicated that eOD→core was the best regimen for advancing SHM toward bnAb development.

## Affinity maturation due to heterologous boost

**Fig. 6.**
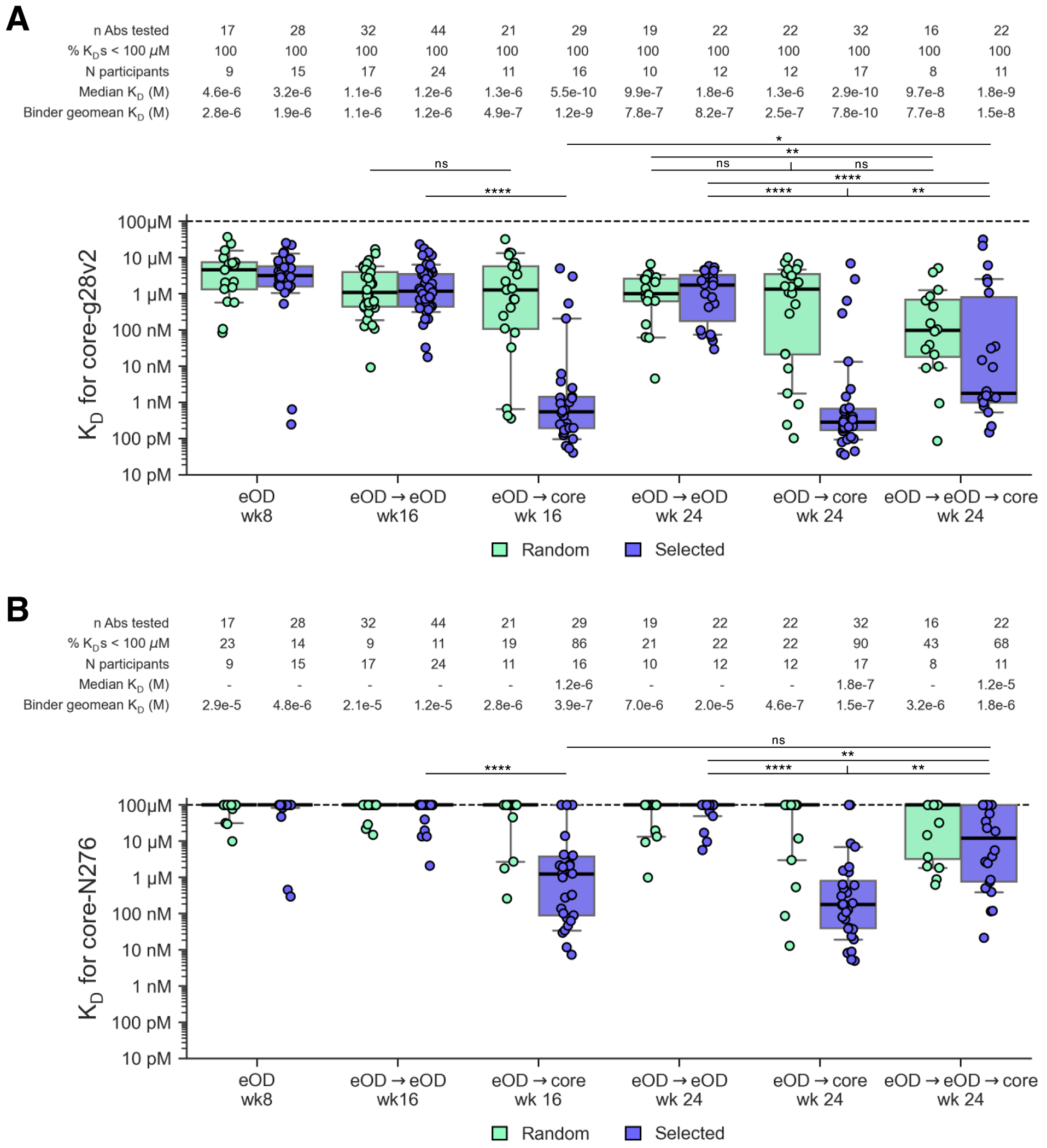
SPR analysis of VRC01-class BCR affinities for core and core-N276. **(A** and **B)** Monovalent *K*_D_ values for “random” and “selected” antibody ligands binding to core-g28v2 monomer analyte (A) and core-N276 analyte (B). Thick lines indicate median values, boxes show 25% and 75% quantiles, and whiskers approximate 10% and 90% quantiles. For median *K*_D_, “-” indicates median ≥ 100 μM. The 608 *K*_D_ values in this figure were each measured twice. Statistical comparisons were made using a marginal mean model fit with generalized estimating equations (table S63). Significant differences had FDR Q-value ≤0.2 and P-values of <0.05 (*), <0.01 (**), <0.001 (***), or <0.0001 (****); ns indicated not significant. All mAbs in this figure correspond to B cells sorted as core-g28v2 CD4bs-specific. Source data can be found in Data S10.

To evaluate affinity maturation in VRC01-class responses in the different regimens, we identified and produced two types of VRC01-class BCRs and evaluated their binding to various antigens by surface plasmon resonance (SPR). For unbiased comparisons, we selected, at random, two VRC01-class BCRs from a subset of participants (at least half of each group sorted by core-g28v2) at each timepoint, and we refer to these as the “random” set. To compare the best (closest to bnAb) clones isolated for each regimen, we selected two VRC01-class BCRs with the highest number of key VRC01-class heavy chain mutations from each participant and timepoint, and we refer to these as the “selected” set (figs. S41 and S42). By assessing binding to monomeric core-g28v2 (Fig. 6A), we found no appreciable difference in median dissociation constant (*K*_D_) between eOD→eOD and eOD→core at week 16 or 24 for the “random” set (e.g. median *K*_D_s for core-g28v2 were 1.1 μM for eOD→eOD and 1.3 μM for eOD→core at week 16). However, for the “selected” set, the eOD→core regimen produced antibodies with affinities that were three orders of magnitude higher than those from eOD→eOD at both weeks 16 and 24: median *K*_D_s for core-g28v2 were 0.6 nM and 0.3 nM for eOD→core at weeks 16 and 24, respectively, but only 1167 nM and 1762 nM for eOD→eOD, at weeks 16 and 24, respectively (Fig. 6A and table S63). The higher affinities for the eOD→core “selected” mAbs were associated with ~1000-fold decreases in off-rate and ~2-fold increases in on-rate (fig. S43). Evidently, heterologous boost provided little affinity advantage at the median level for the entire VRC01-class BCR population (represented by the “random” set) but generated a massive affinity advantage at the median level of the BCRs with the most favorable sequence maturation (represented by the “selected” set). This observation illustrated the value of assessing maturation of both the entire population and the favorable tail of the population and indicated that subsequent heterologous boosts should aim to drive maturation of the best members of the VRC01-class memory/GC pool. Overall, the core-g28v2 SPR data demonstrated that heterologous boosting was capable of generating a small but elite fraction of responses with substantially higher affinity than was achieved by homologous boosting, and eOD→core performed best in generating that elite response.

Gaining the capacity to accommodate the N276 glycan is a critical aspect of VRC01-class maturation that primarily requires acquiring light chain mutations in LCDR1 and LFW3 that interact with or reduce clashes with this glycan (29, 47, 48). As the core boost immunogen tested here did not include the N276 glycan, selection of such N276-interacting mutations was not expected, and in fact we detected only very low levels of key VRC01-class light chain mutations in the post-boost antibodies (fig. S44). However, selection of favorable heavy chain mutations that increase affinity in VRC01-class BCRs can in principle allow for some degree of binding to antigens bearing the N276 glycan, as the increased affinity through improved heavy chain interactions may offset the loss of affinity due to the presence of the N276 glycan. We evaluated the “random” and “selected” antibodies from the different boosting regimens for binding to a variant of core-g28v2 that contains the N276 glycan, a variant we refer to as core-N276 (Fig. 6B and figs. S45 and S46). The data showed that only the heterologous boost regimens could generate antibodies with detectable affinity for core-N276, that eOD→core induced significantly higher affinity antibodies for core-N276 than eOD→eOD→core (week 24 median *K*_D_s for “selected” Abs were 178 nM and 12.2 μM, respectively), and that the affinity drop due to the presence of the glycan was approximately 600-fold to 2,000-fold (eOD→core regimen “selected” Ab median *K*_D_s for core and core-N276 were 0.6 nM and 1249 nM at week 16, respectively, and 0.3 nM and 178 nM at week 24, respectively) (26, 27). The core-N276 SPR data confirmed the superiority of the eOD→core regimen for inducing favorable affinity maturation. The data also demonstrated that at least a subset of the VRC01-class BCRs induced by that regimen at weeks 16 and 24 have acquired the capacity to accommodate the N276 glycan on core-g28v2, an important advance toward bnAb development.

The ultimate goal for the vaccine strategy tested here is to induce VRC01-class bnAbs that bind to diverse native trimers harboring the N276 glycan. This demands that the antibodies can make it past a glycan gauntlet, in which the N276 and other glycans surrounding the CD4bs on the trimer exact a toll on binding affinity and neutralization potency. Knowing that the presence of the N276 glycan alone causes a large drop in affinity (as could be seen in our core-N276 data), we measured binding of the “selected” VRC01-class antibodies from each regimen to diverse native trimers (including from different clades) both with and without the N276 glycan. Although the eOD and core immunogens were not native trimers and did not contain the N276 glycan, we found that subsets (6 to 54%) of VRC01-class antibodies from the eOD→core regimen at weeks 16 and 24 could bind with modest affinities (geomean *K*_D_ values ranged 0.5 to 1.4 μM) to three of the four N276-containing trimers tested (fig. S47). Furthermore, we found that the eOD→core regimen induced VRC01-class antibodies with substantial affinities for all four trimers lacking the N276 glycan (figs. S48 to S51, and table S64). In contrast, non-VRC01-class, core CD4bs-specific BCRs, chosen randomly as one per participant per timepoint and produced for SPR studies, had substantially less capacity to bind the same set of N276-lacking trimers (fig. S52). Thus, we found eOD→core induced VRC01-class responses that could accommodate both the sequence variation within the native-trimer CD4bs and the glycan shield around the native-trimer CD4bs, in some cases including the N276 glycan, another important advance toward bnAb development.

## Neutralization

To determine if trimer binding affinity would translate into neutralizing activity, we evaluated many of the “selected” antibodies from the eOD→eOD (week 16), eOD→core (weeks 16 and 24), and eOD→eOD→core (week 24) immunization regimens for their capacity to neutralize a panel of 14 pseudoviruses derived from five different HIV isolates not represented in the vaccine (26, 27). In accordance with our SPR data, we found that none of the antibodies could neutralize pseudoviruses bearing wild-type (N276-containing) trimers (Table 1 and table S65), and most antibodies from the eOD→eOD and eOD→eOD→core regimens failed to neutralize most N276-lacking pseudoviruses (0% and 32%, respectively, neutralized >1 isolate) (table S65). In contrast, most antibodies from the eOD→core regimen at both weeks 16 and 24 (54% and 66%, respectively) had neutralizing activity (in some cases potent activity) against N276-lacking pseudoviruses from more than one isolate (Table 1). We compared geometric mean IC_50_ for regimens eOD→eOD versus eOD→core at week 16, eOD→core versus eOD→eOD→core at week 24, and eOD→core at wk 16 versus eOD→eOD→core at week 24, and we found significantly lower geomean IC_50_ values in eOD→core in each comparison (4.9-, 5.9-, and 2.8-fold lower; *P* values <0.0001, <0.0001, and 0.0013, respectively; table S66). Thus, in terms of mAb neutralizing activity, we found that heterologous boost was superior to homologous boost, and that the eOD→core regimen was best overall.

For several pseudoviruses for which we had obtained SPR binding data for the corresponding trimer, we noted an apparent quantitative relationship between neutralization IC_50_ and binding *K*_D_, with detectable neutralization arising only for *K*_D_<500 nM, and IC_50_ of 1 μg/ml corresponding approximately to a *K*_D_ of 50 to 100 nM (fig. S53). Neutralizing activity was associated with off-rates slower than 0.004 s^−1^ (fig. S54) but showed no detectable association with on-rate (fig. S55).

We evaluated serum neutralization at weeks 16 and 24 against the same pseudoviruses for which mAb neutralization was detected at those same timepoints. No serum neutralization was detected at serum dilutions as low as 1:10. Thus, the serum frequency of IgGs corresponding to the neutralizing BCRs isolated from memory B cells was too low to generate detectable serum neutralization.

## Prevalence of elite bnAb-precursor responses

**Fig. 7.**
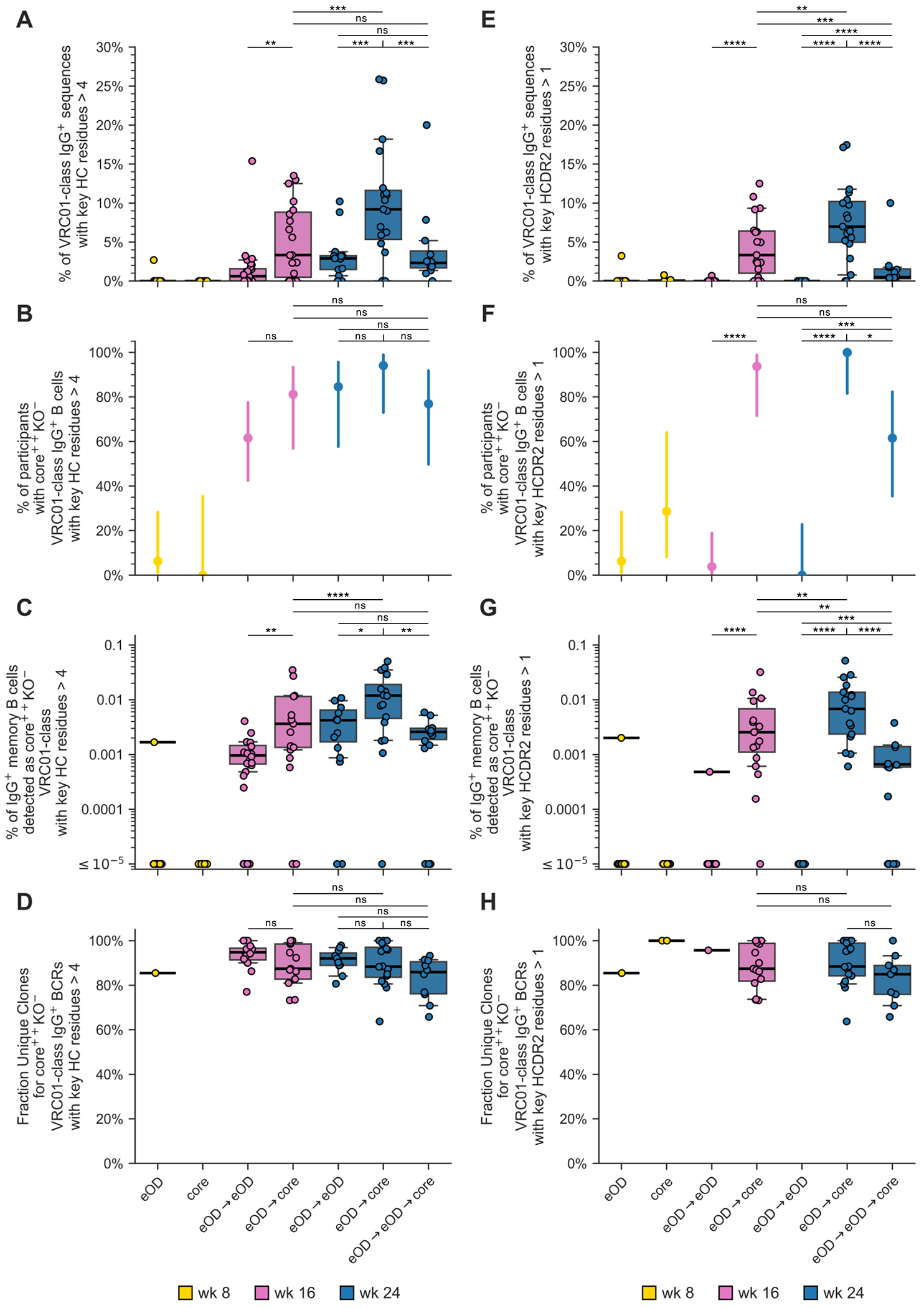
Response rate, frequency, and polyclonality of elite VRC01-class responses. **(A)** Per participant percentage of VRC01-class BCRs with >4 key VRC01-class HC residues **(B)** Percentage of participants with at least one core^++^KO^−^ VRC01-class IgG B cell detected with >4 key VRC01-class HC residues. **(C)** Percentage of core^++^KO^−^ VRC01-class B cells with >4 key VRC01-class HC residues, among IgG memory B cells. **(D)** Polyclonality of core^++^KO^−^ VRC01-class IgG B cells with >4 key VRC01-class HC residues. **(E)** Per participant percentage of VRC01-class BCRs with >1 key VRC01-class HCDR2 residue. **(F)** Percentage of participants with at least one core^++^KO^−^ VRC01-class IgG B cell detected with >1 key VRC01-class HCDR2 residue. **(G)** Percentage of core^++^KO^−^ VRC01-class IgG B cells with >1 key VRC01-class HCDR2 residue, among IgG memory B cells. **(H)** Polyclonality of core^++^KO^−^ VRC01-class IgG B cells with >1 key VRC01-class HCDR2 residue. In (B) and (F), circles indicate median values, and lines indicate 95% CIs computed using the Wilson score method. In (A), (C), (D), (E), (G), and (H), circles represent participants, thick lines indicate median values, boxes indicate 25% and 75% quantiles, and whiskers approximate 90% and 10% quantiles. Testing between groups was done using Barnard’s exact test for the binary outcome in (B) and (F) and the Wilcoxon rank-sum test for the continuous outcomes in the other panels (table S67). Significant differences had FDR Q-value ≤0.2 and P-values of <0.05 (*), <0.01 (**), <0.001 (***), or <0.0001 (****); ns indicated not significant. All B cells in this figure were sorted as core-g28v2 CD4bs-specific. Source data can be found in Data S10.

Our findings that the “selected” mAbs from the eOD→core regimen could bind and neutralize native-like trimers lacking the N276 glycan and bind to N276 glycan-containing trimer were encouraging but raised the question of how common those elite responses were. Noting that the majority of the “selected” mAbs from the heterologous boost regimens had five or more VRC01-class key HC residues, whereas nearly all the “random” mAbs had fewer than five such residues (fig. S40C), we assessed the frequencies among memory B cells and the detection rates across vaccine recipients for VRC01-class responses with five or more VRC01-class key HC residues. We found that such elite VRC01-class responses, which represented a median of 3% or 9% of VRC01-class BCRs per recipient of the eOD→core regimen at week 16 or 24, respectively (Fig. 7A), were detected in more than 80% or 90% of the recipients of the eOD→core regimen at week 16 or 24, respectively (Fig. 7B), had median frequencies among IgG memory B cells of responders of 0.0036% (1 in 28,000) and 0.012% (1 in 8,300), respectively (Fig. 7C), and were highly polyclonal (Fig. 7D). We found similar results when focusing on VRC01-class responses with >1 HCDR2 key residue (Fig. 7, E to G). We also noted that the eOD→core regimen exhibited superiority to eOD→eOD (at week 16 or 24) or eOD→eOD→core (at week 24) in the frequencies of these elite responses among memory B cells (Fig. 7, C and G; table S67). We concluded that the eOD→core regimen produced a diverse pool of VRC01-class IgG B cells that had substantially increased maturation toward bnAb, that occurred with high consistency across vaccine recipients, and that had appreciable frequency among memory B cells.

## Investigating mechanisms for superiority of eOD→core over eOD→eOD→core

The superiority of the eOD→core regimen over the eOD→eOD→core regimen across multiple outputs led us to consider how the conditions of VRC01-class frequency and affinity, and of serum IgG binding, might have differentially impacted the core boost in the two regimens. Evaluating these factors at week 16 after eOD→eOD relative to week 8 after eOD, we found that: (i) the median frequency of core-g28v2-specific VRC01-class IgG B cells was 2.7-fold higher at week 16 (Fig. 4D and table S68); (ii) the percentage of participants with detectable core-g28v2-specific VRC01-class IgG B cells was similar at weeks 8 and 16 (100% and 92%, respectively; Fig. 4E and table S68); and (iii) median VRC01-class affinities for core-g28v2 were 2.5-fold and 1.6-fold higher for the “random” and “selected” mAbs, respectively, at week 16 (Fig. 6A), although the affinity differences were not statistically significant (table S68). Thus, in terms of the frequency and affinity of core-g28v2-specific VRC01-class IgG memory B cells targeted by the core boost, conditions were more favorable for a core boost at week 16 after eOD→eOD. When evaluating serum IgG binding at week 16 after eOD→eOD relative to week 8 after eOD, we found that: (i) the serum IgG binding antibody response rates for core-g28v2 and core-g28v2 CD4bs were significantly higher at week 16 (17% at week 8 and 76% at week 16 for both core-g28v2 and core-g28v2 CD4bs) (fig. S10 and table S69); and (ii) the serum IgG binding antibody median magnitudes were significantly higher at week 16 for core-g28v2 60mer (10.3-fold higher), core-g28v2 monomer (13.9-fold higher), core-g28v2 CD4bs (14.7-fold higher), and lumazine synthase (11% higher) (fig. S10 and table S70). Thus, lower response rates and magnitudes of serum IgG binding antibodies at week 8 favored a core boost at week 8. Consistent with a blunting of the core boost at week 16 due to serum IgG interference, we found that the core-g28v2 60mer boost at week 8 versus at week 16 led to significantly higher fold-increases in core-g28v2-specific IgG B cells (Fig. 4A), core-g28v2 CD4bs-specific IgG B cells (Fig. 4B), and core-g28v2 CD4bs-specific VRC01-class IgG B cells (Fig. 4D) (table S71). We therefore concluded that the higher serum IgG binding to core-g28v2 CD4bs, monomer, and 60mer at week 16 was the dominant factor causing the eOD→eOD→core regimen to be less effective than the eOD→core regimen at driving further maturation of VRC01-class responses.

## Next-stage boost candidates and structural mimicry of VRC01

**Fig. 8.**
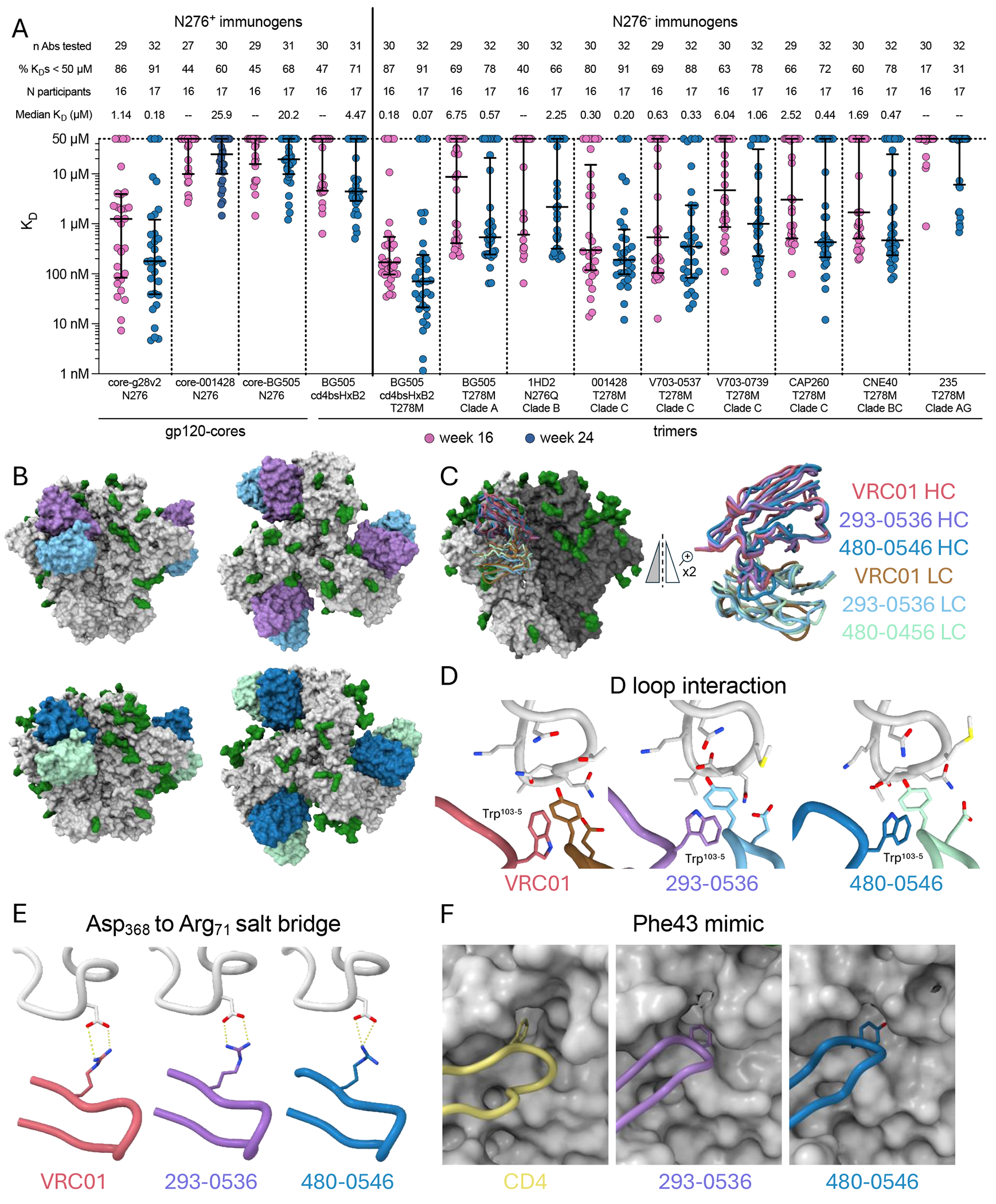
VRC01-class mAbs from eOD→core regimen interacting with candidate next-boost immunogens. (**A**) Apparent *K*_D_ values for “selected” VRC01-class antibodies elicited by the eOD→core immunization regimen (weeks 16 and 24) binding to candidate next-boost immunogens. Horizontal lines represent median and 25% and 75% quantiles. (**B**) Molecular surface representation of atomic models determined by cryo-EM: 293-0563 bound to the 001428 T278M trimer (top) and 480-0546 bound to the V703-0537 T278M trimer (bottom). (**C**) Structural comparison of VRC01 (PDB: 6V8X) to 293-0563 and 480-0546 after aligning on gp120s. (**D**) Molecular contacts of the LCDR3s and Trp^103-5^ to Env D loop residues for VRC01 (left), 293-0563 (center), and 480-0546 (right). (**E**) Arg_71_ to Asp_368_ salt bridges for VRC01 (left), 293-0563 (center), and 480-0546 (right). (**F**) Aromatic residues inserting into the Phe43 pocket for human CD4 (PDB: 5VN3) (left), 293-0563 (center), and 480-0546 (right). Source data for (A) can be found in Data S10.

To identify candidates for the next boost to follow eOD→core, we evaluated “selected” VRC01-class antibodies elicited by the eOD→core immunization regimen (weeks 16 and 24) for their ability to bind an expanded set of heterologous gp120 cores with the N276 glycan or heterologous trimers [including two from recently circulating viruses (2)] modified to lack this glycan (Fig. 8A). The gp120 core sequence alignment is shown in fig. S56, and glycan occupancies are shown in figs. S45 and S57; the trimer sequence alignment is shown in fig. S58, and trimer antigenic profiles are shown in figs. S50 and S59. For comparison, we engineered a native-like Env trimer with an “autologous CD4bs” by grafting the CD4bs from core-g28v2 onto the BG505 MD39.3 Env trimer (BG505 cd4bsHxB2). Binding analyses revealed that over 40% of the week 16 antibodies bound heterologous gp120 cores with the N276 glycan and the “autologous CD4bs” trimer containing the glycan (Fig. 8A). Removing the glycan from the “autologous CD4bs” trimer using the T278M mutation substantially enhanced both binding affinity and percent binders (Fig. 8A). Furthermore, the “selected” VRC01-class antibodies demonstrated robust cross-clade binding to heterologous trimers lacking the N276 glycan, with 8 of 9 trimers tested having median K_D_s ≤ 1 μM (Fig. 8A). Thus, the “selected” VRC01-class antibodies elicited by the eOD→core immunization regimen exhibited substantial affinity for multiple next-stage boost candidates.

To assess the potential for serum interference with next-boost candidates, we assessed serum IgG binding responses from the eOD→core group at weeks 16 and 24 to the native-like trimer with the “autologous CD4bs” (BG505 cd4bsHxB2 T278M), to three next-boost candidates, 001428 T278M, CNE40 T278M, and V703-0537 T278M, and to CD4bs knockout (KO) mutants of each trimer. At week 16, 86% of the samples had detectable binding to the “autologous CD4bs” trimer and to 001428 T278M, whereas 0% and 29% had detectable binding to CNE40 T278M and V703-0537 T278M, respectively (table S72). There were no detectable binders to any of the CD4bs KO trimers, indicating that binding was via the CD4bs. Among positive binders at week 16, binding magnitudes were low, with median area under the titration curve (AUTC) <30 for CNE40 T278M and V703-0537 T278M and <100 for BG505 cd4bsHxB2 T278M and 001428 T278M, compared to >40,000 for eOD-GT8 monomer and >10,000 for core-g28v2 monomer (fig. S60). We tested two serum samples with the highest response rates by cryo-electron microscropy polyclonal epitope mapping (cryoEMPEM) and failed to detect trimer binders in either sample. These data suggested that the risk of serum interference with these next boost candidates is relatively low.

Two next-boost candidate trimers (001428 T278M and V703-0537 T278M) and two week 16 VRC01-class antibodies elicited by the eOD→core regimen (293-0536 and 480-0546) were selected for structural analysis using cryo-EM (Fig. 8B, fig. S61, and table S73). The antibodies were representative of the “selected” VRC01-class elicited by the eOD→core immunization regimen at week 16, featuring 6 and 5 key VRC01-class HC residues, respectively, and utilizing the IGKV1-33 and IGKV3-20 light chains (figs. S41C, S42A, and S62). Both contain a single key VRC01-class light-chain residue in the LCDR1 and share Glu96 in the LCDR3 (fig. S62). Both antibodies bound to Env trimers with a similar angle of approach as VRC01 (Fig. 8C). The heavy-chain C*α* root mean square deviation (RMSD) relative to VRC01 was 1.5 Å for 293-0536 and 2.0 Å for 480-0546 after alignment on gp120 (Fig. 8C). Buried surface area (BSA) analysis revealed that both antibodies bound Env with nearly identical epitope footprints as VRC01 (fig. S63A), with their HCDR2 regions contributing approximately 50% of the BSA (fig. S63B). The two antibodies share many canonical VRC01-class interactions, including contacts with the D loop through short LCDR3s and Trp^103-5^ (Fig. 8D), Asp368-to-Arg71 salt bridges (Fig. 8E), and aromatic residues at position 54 mimicking the Phe43 residue of human CD4 (Fig. 8F). In summary, these “selected” VRC01-class antibodies elicited by the eOD→core immunization regimen bound to two different trimers in a manner nearly identical to that of VRC01, demonstrating that the eOD→core heterologous prime-boost regimen elicited VRC01-class responses with precise structural mimicry of the target bnAb and on a structural pathway consistent with bnAb development.

## Discussion

Germline-targeting vaccine design seeks to induce bnAbs through a stepwise vaccine approach. The conceptual requirements include the design of priming immunogens that consistently induce responses from naive bnAb-precursor B cells, the design of heterologous boost immunogens, or “shepherding” immunogens, that activate partially mature bnAb-precursors from the prior stage and drive maturation further toward bnAb development in the GC and memory B cell compartments, and finally, once bnAbs have developed in those compartments, the design of polishing immunogens that generate high frequencies of long-lived plasma cells secreting bnAbs. The IAVI G001 trial demonstrated proof-of-principle for germline-targeting priming, using adjuvanted protein (15–17). Here, the IAVI G002 and G003 trials showed that efficient priming can also be achieved with mRNA-LNP in both North American and African populations, thus opening the door for potential mRNA-enabled accelerated clinical testing of germline-targeting vaccines and extending clinical proof of principle for the germline-targeting priming concept to African populations in most need of an HIV vaccine. Most important as a conceptual advance, G002 demonstrated clinical proof of principle for germline-targeting heterologous boosting. Showing that a heterologous boost can drive a pre-specified bnAb-precursor response further toward bnAb development at substantial frequencies among memory B cells and with consistency across human vaccine recipients represents a major step forward for the germline-targeting strategy and for HIV vaccine development.

Our finding in G002 that a substantial proportion of the “selected” VRC01-class mAbs from the eOD→core regimen had neutralizing activity to pseudoviruses lacking the N276 glycan demonstrated that the germline-targeting strategy can generate pre-specified BCR classes in the memory compartment with functional neutralizing capacity in humans. The fact that many of those same antibodies could also bind to core-N276 and heterologous core-gp120s with N276, and a subset of the antibodies could bind at least one wild-type trimer containing the 276 glycan, indicated that the antibodies were on productive pathways toward developing the capacity to bind and neutralize trimers containing the N276 glycan, meaning the antibodies were on pathways toward bnAb development. The overall frequencies of VRC01-class IgG B cells with similar numbers of key VRC01-class residues as the “selected” mAbs were substantial (e.g. 1 in 28,000 or 1 in 8,300 among IgG B cells in PBMCs for the eOD→core regimen at week 16 or 24, respectively), showing that appreciable numbers of VRC01-class B cells with partially mature neutralizing capacity developed in the participants in response to heterologous prime-boost vaccination. The polyclonality of this elite population of VRC01-class B cells was high, showing that diverse clones were developing on these desirable trajectories. Although serum neutralization was not detected, at this intermediate stage of the sequential vaccination regimen serum neutralization is not required and is probably not desirable, because substantial frequencies of serum IgG that can bind and neutralize N276-lacking trimers could interfere with the next boost employing an N276-lacking trimer, just as serum IgG likely interfered with the core boost in the eOD→eOD→core regimen. Our cryo-EM studies demonstrated that VRC01-class BCRs elicited by the eOD→core regimen can bind to N276-lacking trimers using the canonical VRC01-class binding mode, which supports the potential for these BCRs to mature further toward bnAb development through additional heterologous boost vaccinations.

Comparison of VRC01-class IgG B cell responses to eOD-GT8 60mer delivered by mRNA-LNP as in IAVI G002 and G003 or delivered as adjuvanted protein as in IAVI G001 indicated potential immunogenicity advantages to mRNA-LNP. With the caveats that the adjuvanted protein and mRNA-LNP immunogens were not compared in the same study, the B cell responses in the three studies were analyzed using different methods, and the dose of folded protein resulting from mRNA-LNP vaccination is not known, our finding of two-fold higher SHM in VRC01-class responses in G002 and G003 compared to G001 was surprising and merits further investigation to understand mechanisms. The higher frequencies of VRC01-class IgG B cells generated by 100 μg mRNA-LNP in G002 compared to adjuvanted protein in G001 (e.g. 6.9-fold higher at week 8) suggest that lower doses of mRNA-LNP might be used to achieve similar responses to those in G001. A lower vaccine dose might in turn result in reduced reactogenicity. Overall, the data supported further studies to test lower doses for the eOD→core regimen.

Engineered self-assembling nanoparticles, which can mimic the valencies and sizes of viral and bacterial particles to which the human immune system has been tuned, represent a promising vaccine technology. However, mRNA-LNP delivery of self-assembling nanoparticle antigens requires design of nanoparticle antigens that express and assemble properly at high yield in vivo without the advantages of purification, and it has remained unknown whether mRNA-LNP delivery of self-assembling nanoparticles could be effective in humans. The potent memory B cell and serum binding antibody responses in G002 and G003, including undiminished serum binding antibody titers to eOD-GT8 monomer at week 24 in G002, provide clinical proof of concept for mRNA-LNP delivery of nanoparticle antigens and support testing delivery of other nanoparticles by mRNA-LNP.

With targeted funding for G003, African research laboratories collaborated with international research partners to establish and execute serum antibody binding measurements and B cell sorting and BCR sequencing. As a result, all G003 serum and PBMC samples subjected to such assays were analyzed in African research laboratories. Thus, the G003 study demonstrates a pathway to expand immunology capacities and support the next generation of African scientists who will likely need to play an important role in developing an HIV vaccine.

Although adverse reactions, including urticaria, could occur with any product, the occurrence of chronic urticaria in 10% of G002 participants was unexpected. However, the urticaria events were assessed as mild and moderate in severity, non-serious, were largely managed with over-the-counter medications, and while persistent, ultimately resolved during the trial. Defined as wheals (hives) or angioedema (swelling) lasting continuously or intermittently for at least 6 weeks, chronic urticaria occurs at a rate of 0.23% in the general United States population (44). In the large phase 3 trial of the Moderna mRNA-LNP COVID-19 vaccine, urticarial reactions of any type were reported at rates of 0.4 and 0.3% in the vaccine and placebo groups, showing no significant increased risk in the vaccine group (49). Both delayed urticaria (beginning from 4 hours to seven days after vaccination) and chronic urticaria have been observed in recipients of the COVID-19 mRNA-LNP vaccines in the post-licensure setting (50), but the estimated incidence rates in response to the Moderna Spikevax booster have also been substantially lower than the rate observed in G002. For example, one study in Switzerland (51) reported a chronic urticaria rate of 0.0308% (95% CI: 0.0284%-0.0334%) from 1,970,971 people receiving a Spikevax booster. To further examine the risk of urticaria associated with the Moderna mRNA vaccine platform we conducted a pooled analysis of urticaria and angioedema adverse events across Moderna-sponsored investigational trials. We found that the rate of chronic urticaria or angioedema across both primary and booster vaccination studies was substantially (>140-fold) lower than what was observed in the G002 study. Notably, in this pooled analysis, the rate of urticaria or angioedema was comparable in the cohorts who received a variety of investigational mRNA vaccines and either placebo or active non-mRNA comparator vaccines. Furthermore, our analysis of the rate of urticaria or angioedema versus the number of prior Moderna COVID-19 vaccinations showed no rate increase for up to six vaccinations. These data suggest that the rate of urticaria in G002 was related to the specific vaccines tested in this trial.

The safety and tolerability profile in G003 exhibited some differences for skin adverse reactions compared to North American participants in the G002 trial who received the same eOD-GT8 60mer mRNA-LNP vaccine regimen. No urticaria or dermatographism was reported in G003. Two G003 participants (2/18; 11.1%) experienced pruritus with delayed onset (13-18 days post second vaccination) that lasted for 8 days in both cases and in G002, eight of the 52 (15.4%) participants who received two vaccinations with eOD-GT8 60mer mRNA-LNP experienced pruritus that was also delayed in onset (median delay of 20 days). However, the pruritus reported in G002 seemed to have different features than in G003, with longer duration (median duration of 68 days), and with half of the cases co-occurring with urticaria or dermatographism and considered part of the same clinical event. Additional studies would be needed to evaluate the statistical significance and potential molecular, cellular, genetic, and environmental causes for the observed differences in skin reactions between African and North American populations receiving eOD-GT8 60mer mRNA-LNP in G002 and G003.

It is also important to consider that the small size of the G002 and G003 trials, open-label study designs, and absence of a placebo or active control are important limitations in determining the risk associated with the investigational vaccines used. In this context, any retrospective comparisons of baseline factors in these studies, such as demographic and medical history between participants who reported urticaria and those who did not, are subject to bias and should be considered exploratory or hypothesis generating. Nevertheless, the increased incidence of urticaria reported by participants in the G002 study compared to the absence of the events with the recombinant antigen combined with adjuvant in the earlier G001 study (15) warrants further characterization.

The higher rate of chronic urticaria observed in G002 compared to the rates observed for the Spikevax primary (49) and booster (51) vaccines suggested a potential contributing role for the HIV antigens expressed in vivo. The fact that most (11/14) of the skin AEs observed in G002 occurred after immunization with eOD-GT8 60mer mRNA-LNP, whereas no skin AEs were observed after immunization with eOD-GT8 60mer protein and AS01_B_ adjuvant in G001 (15), suggested that factors that differ between the two delivery systems might be playing a role. We considered that these factors could include the occupancy and glycoforms of the many N-linked glycosylation sites present on eOD-GT8 60mer. These could differ between the form expressed in vivo from mRNA-LNP and the purified protein, because the protein was produced from HEK293 cells in the presence of the mannosidase I inhibitor kifunensine, which caused the majority (~92%) of expressed glycans to be Man9 (52). Furthermore, several glycosylation sites on the purified protein were completely or substantially underoccupied (52), which can affect glycoforms at adjacent sites (53). T cell responses to eOD-GT8 60mer could also potentially differ, in magnitude, specificity, or kinetics, between protein (16) and mRNA-LNP delivery. Finally, the mRNA-LNP will likely have produced a greater degree of incompletely assembled eOD-GT8 60mer nanoparticles compared to delivery of purified protein.

Chronic urticaria was also observed in a different phase 1 trial (HVTN302, NCT05217641) evaluating mRNA-LNP delivery of different HIV Env-based immunogens (native-like trimers based on the BG505 isolate) (54). We have considered that shared glycan or T cell epitopes between the different immunogens, or aspects of the mRNA-LNP platform, or other uncontrolled covarying factors, could potentially have contributed to the skin reactions. A multi-institutional working group was formed to study the skin adverse events and attempt to discover biological or immunological correlates. Overall, the observed skin adverse events need to be better understood and the implications and potential mitigation strategies better defined.

Our immunological analyses suggest that the responses to the eOD→core regimen represent a promising starting point for additional heterologous boost immunogens to drive the VRC01-class responses to bnAb production. Whether or not the elite population of VRC01-class B cells we identified can be boosted and matured further toward bnAb development should be tested in future trials. Boost immunogens to follow core-g28v2 60mer would be Env trimers lacking the N276 glycan followed by wild-type trimers containing the N276 glycan (26, 27). We believe the mRNA-LNP platform is essential for expediting the development of a germline-targeting HIV vaccine that induces multiple classes of bnAbs, based on the high efficacy and safety of licensed mRNA-LNP vaccines, the speed and adaptability of the mRNA-LNP platform for clinical studies of diverse immunogens, and the ability to deliver membrane-anchored Env trimer immunogens (54–57). The next step for this clinical program is a follow-up study in South Africa (HVTN317/DESIIGN001, NCT06694753) to test lower doses of mRNA-LNP for the eOD→core regimen, to determine if the occurrence of skin AEs can be reduced or eliminated while consistent bnAb-precursor priming and boosting is maintained. The strong VRC01-class responses and maturation induced by 100 μg mRNA-LNP for the eOD→core regimen here in IAVI G002 suggest that lower doses might be capable of inducing the desired elite VRC01-class responses.

## Materials and Methods

### G002 study design

IAVI G002 (clinicaltrials.gov NCT05001373) was a phase 1, randomized, open label study to evaluate the safety and immunogenicity of the eOD-GT8 60mer mRNA-LNP vaccine (mRNA-1644) and the core-g28v2 60mer mRNA-LNP vaccine (mRNA-1644v2) in HIV-uninfected adults in good general health in the United States. The immunological hypothesis was that sequential vaccination with a germline-targeting prime followed by a directional boost immunogen could induce VRC01-class bnAb-precursor B cell responses and guide their early maturation toward bnAb development, using an mRNA-LNP vaccine platform. Sixty participants were immunized with either 100 μg eOD-GT8 60mer mRNA-LNP at weeks 0 and 8 (group 1; n=17), 100 μg eOD-GT8 60mer mRNA-LNP at week 0 followed by 100 μg core-g28v2 60mer mRNA-LNP at week 8 (group 2; n=17), 100 μg eOD-GT8 60mer mRNA-LNP at weeks 0 and 8 followed by 100 μg core-g28v2 60mer mRNA-LNP at week 16 (group 3; n=18), or 100 μg core-g28v2 60mer mRNA-LNP at week 0 (group 4; n=8) (fig. S2A and tables S1 to S3). Vaccines were administered intramuscularly in the deltoid, and each participant received all vaccinations in the same deltoid. The vaccination schedules and PBMC sampling timepoints essential for assessing VRC01-class responses are depicted in Fig. 1. The full schedules of procedures for safety and immunogenicity evaluations are given in tables S7 to S10. The CONSORT diagram is shown in fig. S2A.

The primary objectives of the study were to evaluate the vaccines for safety and tolerability and the capacity to induce Immunoglobulin G (IgG) B cell responses from rare precursors for VRC01-class bnAbs. The primary endpoints were the occurrence of adverse events (AEs), assessed as the proportion of participants with: (1) local or systemic solicited AEs from day 0 to day 7 inclusive after each IP administration; (2) Grade 2 or higher unsolicited AEs, including safety laboratory (biochemical, hematological) parameters from the day of each IP administration through 28 days after each IP administration; (3) SAEs at any time during the study period; (4) MAAEs or AESIs from the first day of IP administration through 24 weeks post final IP administration. AESIs defined by the protocol included thrombocytopenia, anaphylaxis, myocarditis/pericarditis, or a new onset or worsening of the following specific neurologic diseases: Guillain-Barre Syndrome, acute disseminated encephalomyelitis (ADEM), idiopathic peripheral facial nerve palsy (Bell’s palsy), and seizures including but not limited to febrile seizures and/or generalized seizures/convulsions. The secondary endpoints were: (1) the proportion of participants with VRC01-class IgG B cells, and frequency of VRC01-class B cells among IgG B cells in PBMCs or in germinal centers, after each IP administration and at baseline; and (2) the proportion of participants with serum binding antibody responses to eOD-GT8 60mer, eOD-GT8 monomer, core-g28v2 60mer, core-g28v2 monomer, the CD4bs epitope on eOD-GT8, and the CD4bs epitope on core-g28v2, and the magnitude of those responses, after each IP administration and at baseline. Exploratory endpoints included analyses of SHM, affinity, and neutralization capacity to determine if the responses from priming and boosting were conducive to bnAb development; analysis of personalized VH1-2 genotypes for each participant to assist interpretation of VRC01-class response rates; and magnitude and specificity of CD4 T cell responses to the vaccines.

#### G003 study design

IAVI G003 (clinicaltrials.gov NCT05414786) was a phase 1 study to evaluate the safety and immunogenicity of eOD-GT8 60mer mRNA vaccine in HIV-1 uninfected adults in good general health at two sites in Africa; one site in Rwanda and one in South Africa. The immunological hypothesis was that priming with eOD-GT8 60mer mRNA-LNP vaccine in African populations would elicit VRC01-class bnAb precursor responses with similar response characteristics to those observed for the same vaccine regimen evaluated in North American populations in IAVI G002. Eighteen (10 males, 8 females) healthy HIV-1 uninfected adults, aged 21 to 41 years were enrolled from Rwanda (n = 10) and South Africa (n = 8). All participants were of African origin. Each participant received two 100 μg doses of eOD-GT8 60mer mRNA-LNP vaccine administered via intramuscular (IM) injection into the non-dominant arm eight weeks apart. Participants were followed for six months after the last administration of investigational product (IP). The vaccination schedules and PBMC sampling timepoints essential for assessing VRC01-class responses are depicted in Fig. 1. The full schedules of procedures for safety and immunogenicity evaluations are given in tables S11 and S12. The CONSORT diagram is shown in fig. S2B.

The primary objectives of the study were to evaluate the vaccines for safety and tolerability and the capacity to induce Immunoglobulin G (IgG) B cell responses from rare precursors for VRC01-class bnAbs. The primary endpoints were the occurrence of adverse events (AEs), assessed as the proportion of participants with: (1) local or systemic solicited AEs from day 0 to day 7 inclusive after each IP administration; (2) Grade 2 or higher unsolicited AEs, including safety laboratory (biochemical, hematological) parameters from the day of each IP administration through 28 days after each IP administration; (3) Serious AEs (SAEs) at any time during the study period; (4) medically attended AEs (MAAEs) or adverse events of special interest (AESIs) from the first day of IP administration through 24 weeks post final IP administration. AESIs defined by the protocol included thrombocytopenia, anaphylaxis, myocarditis/pericarditis, or a new onset or worsening of the following specific neurologic diseases: Guillain-Barre Syndrome, acute disseminated encephalomyelitis (ADEM), idiopathic peripheral facial nerve palsy (Bell’s palsy), and seizures including but not limited to febrile seizures and/or generalized seizures/convulsions. The secondary endpoints were: (1) the proportion of participants with VRC01-class IgG B cells, and frequency of VRC01-class B cells among IgG B cells in PBMCs after each IP administration and at baseline; and (2) the proportion of participants with serum binding antibody responses to eOD-GT8 60mer, eOD-GT8 monomer, the CD4bs epitope on eOD-GT8, and the magnitude of those responses, at baseline and after each vaccine administration. Exploratory endpoints included (1) analyses of SHM and affinity to determine if the responses from the two immunizations set the elicited B cell responses on a trajectory for bnAb development; (2) analysis of personalized VH1-2 genotypes for each participant to assist interpretation of VRC01-class response rates; and magnitude and specificity of CD4 T cell responses to the vaccines.

##### G002 participants and randomization

Eligible participants were healthy male and female adults 18 through 50 years of age who were willing to undergo HIV testing, use an effective method of contraception, understood the study in the opinion of the investigator or designee, and provided written informed consent. The study design was for a total of 56 participants, with n=16 each in groups 1 to 3, and n=8 in group 4, but the study design also allowed for over-enrollment of up to two participants per group. Accordingly, 60 participants who met all eligibility criteria were included in the study and were randomly assigned to participate in one of four groups (fig. S2A). Participants were enrolled at four clinical sites: George Washington University (GWU; 25 participants enrolled), University of Texas Health Science Center at San Antonio (UTHSA; 17 participants enrolled), Fred Hutchinson Cancer Center (FHCC; 10 participants enrolled), and Emory University (8 participants enrolled) (Data S1). The first six participants were sentinels with a predetermined vaccine allocation of three to eOD-GT8 60mer mRNA-LNP Vaccine (Groups 1, 2, or 3) and three to core-g28v2 60mer mRNA-LNP Vaccine (Group 4). There was no attempt to match the participants for any demographic category among the four study groups or the four clinical sites. Participant demographics are given in table S3. Among enrolled participants, for sex at birth, approximately equal numbers were male (26/60, 43.3%) and female (34/60, 56.7%); the predominant race reported was White (44/60, 73.3%), with Multiracial (8/60, 13.3%) and Asian (5/60, 8.3%) being the next highest race categories reported. The ethnicity of “Hispanic or Latino” was reported for 20% (12/60) of participants, reflective of the U.S. population. The median age and body mass index were 27.9 years and 24.5 kg/m^2^, respectively. The first vaccination for the first volunteer in G002 occurred on January 20, 2022, and the last visit for the last volunteer occurred on June 20, 2023.

##### G003 participants and randomization

Eligible participants were healthy male and female adults 18 through 50 years of age who were willing to undergo HIV testing, use an effective method of contraception, understood the study in the opinion of the investigator or designee, and provided written informed consent. Participants were enrolled at two clinical research sites, Center for Family Health Research (CFHR) (Kigali, Rwanda) and Aurum Tembisa CRC (Johannesburg, South Africa) between July and September 2022. Forty-four participants were screened to enroll 18 participants overall. There was only one group for the study, consisting of 18 participants, and they all received two doses of 100μg eOD-GT8 60mer mRNA-LNP vaccine at week 0 and 8 and follow up to week 32. There was no blinding and no randomization in this open-label study.

A sentinel strategy was applied to the first three participants enrolled and included a minimum of 72 hours between the dosing of each sentinel. The decision to open study enrollment was based on the recommendation of an independent Safety Monitoring Committee (SMC) after review of the Day 8 safety data of the three sentinels. Participant safety was monitored throughout the study by the Investigators, the Sponsor’s Medical Monitor, a Protocol Safety Review Team (PSRT), and the SMC.

Among enrolled participants, for sex at birth, approximately equal numbers were male (10/18 (55.6%)) and female (8/18 (44.4%)); median age was 33.7 years, and median body mass index (BMI) was 61.5kg. The race of all participants was Black African. The first enrolled participant was administered the first IP injection on 15 July 2022. The last two participants enrolled received their first IP injections on 09 September 2022. At the second IP administration, two participants were out of the predetermined visit window. These two participants’ visits were kept in the analyses due to the minimal deviation from the predetermined window. With the exception of two participants (11.1 %) who missed the Day 4 contact visit (which could be conducted in person or via telephone, text, or email), all participants attended all study visits and completed the study by 12 May 2023.

##### G002 oversight

The trial was conducted under an Investigational New Drug (IND) application submitted to the US Food and Drug Administration, and was carried out in compliance with the protocol filed within the IND. The trial adhered to IAVI standard operating procedures in accordance with the guidelines formulated by the International Committee on Harmonization for Good Clinical Practice in clinical trials. Furthermore, the trial complied with applicable local standards and regulatory requirements including review and approval by the institutional review boards at GWU, UHTSA, FHCC and Emory. The G002 IRB approval numbers were as follows: NCR213652 (GWU), 21-339H (UTHSA), STUDY00002864 (Emory), and 10723 (FHCC). The trial was overseen by a Protocol Safety Review Team and independent Safety Monitoring Committee (SMC).

##### G003 oversight

IAVI G003 was approved in South Africa by South African Health Products Regulatory Authority (SAHPRA), with reference number Ref 20210616) and received ethics approval from University of the Witwatersrand’s Human Research Ethics Committee (Wits HREC), with Ethics Reference 210504B. The study was approved in Rwanda by Rwanda Food and Drugs Authority (RFDA), with Clinical Trial Approval Certificate no. 018/CTAC/FDA/2022, and received ethics approval from Rwanda National Ethics Committee (RNEC), with reference number 970/RNEC/2021. Scientific and ethical approval for the BAMA and BCR immunogenicity analyses was obtained from the Kenya Medical Research Institute Scientific and Ethics Review Unit (KEMRI SERU) under protocol KEMRI/SERU 4177. Study oversight was provided by the International AIDS Vaccine Initiative (IAVI).

##### G002 and G003 blinding

Both studies were open label with no blinding. Participants, site staff, and study staff were not blinded to participant IP allocation in either study.

##### G002 safety and tolerability monitoring

Safety and tolerability were monitored during the trial by site Investigators, the IAVI Medical Monitor and the Protocol Safety Review Team (PSRT). The first six participants were sentinel participants. There was a minimum of 72 hours between IP administration for each of the sentinel participants. An independent SMC reviewed the cumulative 8-day safety data, including safety laboratory results, following the first immunization for all sentinel participants, and recommended opening enrollment to the remaining participants in the study. Per the protocol, ad hoc SMC safety reviews could be requested by the Sponsor, Moderna, the Site Principal Investigators, Institutional Review Boards (IRBs), Regulatory Authorities, or by the SMC. There were three ad hoc SMC meetings to review cases of skin reactions, in October, November, and December 2022, respectively, and two PSRT meetings. No pause criteria were met in the study; however, there were two periods in which vaccinations were paused. One pause in vaccinations occurred per SMC recommendation while Investigator Brochures (IBs) and Informed Consent Forms (ICFs) were updated regarding skin reactions and then approved by site IRBs. That pause ranged from 6 to 34 days depending on the site. The second pause in vaccinations was a 6-day pause from November 22 to December 1, 2022 while waiting for SMC review of skin reactions. Participants were followed up to 12 months after the final investigational product administration. AEs were grouped by Medical Dictionary for Regulatory Activities (MedDRA) terminology System Organ Class (SOC) and Preferred Term (PT). All AEs were graded for the entire duration of the study, using the National Institutes of Allergy and Infectious Diseases (NIAID) Division of AIDS (DAIDS) Table for Grading the Severity of Adult and Pediatric Adverse Events, Version 2.1, July 2017.

##### G003 safety and tolerability monitoring

Safety and tolerability were monitored during the trial by site Investigators, the IAVI Medical Monitor and the Protocol Safety Review Team (PSRT). The study employed a sentinel dosing strategy with the first cohort of 3 participants across the 2 participating sites being enrolled in a staggered manner in advance of the full study cohort. There was a minimum of 72 hours between IP administration for each of the sentinel participants. An independent SMC reviewed the cumulative 8-day safety data, including safety laboratory results, following the first immunization for all sentinel participants, and recommended opening enrollment to the remaining participants in the study. Per the protocol, ad hoc SMC safety reviews could be requested by the Sponsor, Moderna, the Site Principal Investigators, Institutional Review Boards (IRBs), Regulatory Authorities, or by the SMC. No pause criteria were met in the study. There were no ad hoc SMC meetings required for the IAVI G003 trial, however the SMC members from IAVI G003 were included in the IAVI G002 SMC meetings for full visibility of the safety events occurring in that trial. All Investigators were informed and updated on events in IAVI G002, and a participant letter was issued to all participants in IAVI G003 to inform them of the events happening in IAVI G002, as a parallel trial. AEs were grouped by Medical Dictionary for Regulatory Activities (MedDRA) terminology System Organ Class (SOC) and Preferred Term (PT). All AEs were graded for the entire duration of the study, using the National Institutes of Allergy and Infectious Diseases (NIAID) Division of AIDS (DAIDS) Table for Grading the Severity of Adult and Pediatric Adverse Events, Version 2.1, July 2017.

##### G002 immunological assays

Frequencies of antigen-specific and CD4bs epitope-specific B cells were assessed by fluorescence-activated cell sorting (FACS). Frequencies of VRC01-class IgG B cells were assessed by CD4bs-specific B cell sorting, B cell receptor (BCR) sequencing by 10x Genomics, and bioinformatic analysis. Polyclonality and genetic diversity of VRC01-class IgG BCR responses were assessed by bioinformatic analysis including hierarchical sequence clustering. Binding affinities for VRC01-class and non-VRC01-class mAbs were assessed by SPR. Neutralization capacities for VRC01-class mAbs were assessed by TZM-bl neutralization assays. Serum antibody binding responses were assessed by Binding Antibody Multiplex Assay (BAMA), and serum antibody neutralization was assessed using TZM-bl neutralization assays.

##### G003 immunological assays

The frequencies of antigen-specific and CD4bs epitope-specific B cells were assessed by FACS. Frequencies of VRC01-class IgG B cells were assessed by CD4bs-specific B cell sorting, BCR sequencing following 10x Genomics library preparation, and bioinformatic analysis. Polyclonality and genetic diversity of VRC01-class IgG BCR responses were assessed by bioinformatic analysis including hierarchical sequence clustering. Binding affinities for a subset of randomly selected VRC01-class and non-VRC01-class mAbs were assessed by SPR, and serum antibody binding responses were assessed by BAMA. Additional details of immunological assays are provided below.

##### G002 and G003 definition of CD4bs-specific responses

In both trials, assessment of serum or B cell binding to the eOD-GT8 CD4bs epitope was determined by differential binding to eOD-GT8 and eOD-GT8-KO11, a variant of eOD-GT8 with three mutations in the CD4bs (N280R, S365L, and F371R in HxB2 numbering) that essentially abrogates binding by VRC01-class precursor Abs and VRC01-class bnAbs (58–62), as previously reported for IAVI G001 (15). In G002, assessment of serum or B cell binding to the core-g28v2 CD4bs epitope was determined by differential binding to core-g28v2 and core-g28v2-KO11b, a variant of core-g28v2 that contains the same CD4bs mutations as eOD-GT8-KO11, but also contains the D368R mutation (26).

##### G002 power analysis and rationale for trial size

Group sizes for Groups 1 to 3 were selected as n=16 to measure the primary hypothesis that the vaccine would induce VRC01-class IgG B cells with a response rate of at least 50% in at least one of the three groups, as well as to satisfy the need to have enough endpoints for further characterization of that response. We powered the study to have high probability of observing at least 5 participants with a vaccine-induced VRC01-class IgG B cell response among participants in Groups 1, 2 or 3 given that the true response rate for this class of B cells was at least 50% or greater among participants in any one of the three groups. In contrast to IAVI G001, in which we assumed a dropout rate of 10%, here we assumed no dropouts. Under those assumptions, we determined that at least 5 positive responders for VRC01-class B cells would be required for the 95% confidence interval (CI) about the observed rate to be consistent with a true rate of 50%, because the 95% CI for an observed rate of 5/16=31.25% is 14.2% to 55.6%. Furthermore, power was 96.2% to detect 5 or more positive responders out of 16 when the true rate of response was 50% (15). For group 4, we selected a group size of n=8 to enable setting a reasonably restrictive upper limit on the 95% CI for the true response rate if only a small number of responders is observed (table S74).

##### G003 power analysis and rationale for trial size

The G003 study was powered to have a high probability of observing at least 5 participants with a vaccine-induced VRC01-class IgG B cell response among participants, with rationale and calculations matching those described for G001 (15).

##### G002 and G003 vaccine composition

The investigational products (IPs), eOD-GT8 60mer and (in G002 only) core-g28v2 60mer, were supplied as mRNA-LNPs at an mRNA concentration of 0.5 mg/mL. LNPs included 4 lipid excipients: the proprietary ionizable amino lipid SM-102 and three commercially available lipids: cholesterol, DSPC, and PEG2000 DMG. mRNA-LNP vaccines were formulated in 20 mM Trometamol (Tris) buffer, 87 g/L sucrose and 10.7 mM sodium acetate at a pH of 7.5. mRNA-LNP vaccines were sterile filtered and filled into 2R Type I glass vials at a mRNA concentration of 0.5 mg/mL.

#### Study procedures

##### Vaccine preparation in G002 and G003

eOD-GT8 60mer mRNA-LNP Vaccine IP (mRNA-1644) and core-g28v2 60mer mRNA-LNP Vaccine IP (mRNA-1644v2) were each diluted with saline just prior to administration. Saline was 0.9% Sodium Chloride Injection, USP, in single-use 5 mL glass ampules. The final diluted IPs were administered IM in a dose volume of 0.5mL. Injections were administered in the deltoid muscle of the non-dominant arm, and all vaccinations per participant were given to the same arm unless medically contraindicated.

##### Schedules of procedures for G002 and G003

The full schedules of procedures are given in tables S7 to S10 for G002 and tables S11 to S12 for G003.

##### G002 safety and tolerability

At each vaccination visit, vital signs (oral temperature, heart rate, respiratory rate, and blood pressure) were measured by study staff prior to vaccination and at least 30 minutes post-vaccination. Solicited local and systemic reactogenicity were collected from day 0 to day 7 after each vaccination (figs. S3 and S4, tables S14, S16, and S17, and Data S2 and S3), which was accomplished by participants recording events using a memory aid. The preferred memory aid was an electronic diary that participants completed in real time using a cell phone app. Unsolicited AEs were collected initially through unprompted self-reporting but later, after initial cases of skin AEs, through open-ended questions. Unsolicited AEs were collected from day 1 to day 28 after each vaccination (tables S14 and S15, Data S4 and S5). SAEs, MAAEs, and AESIs were collected during the entire study period through 6 months after the last IP administration (table S14). Schedules for collecting AEs, SAEs, MAAEs, and AESIs are shown in table S7 (Group 1), table S8 (Group 2), table S9 (Group 3), and table S10 (Group 4).

##### G003 safety and tolerability

At each vaccination visit, vital signs (oral temperature, heart rate, respiratory rate, and blood pressure) were measured by study staff prior to vaccination and at least 30 minutes post-vaccination. Solicited local and systemic reactogenicity were collected from day 0 to day 7 after each vaccination (figs. S6 and S7, and tables S22 to S24). This was recorded in a participant paper diary and reviewed by the Investigator. Unsolicited AEs were collected through open-ended questions. Unsolicited AEs were collected from day 1 to day 28 after each vaccination and SAEs, MAAEs, and AESIs were collected during the entire study period through 6 months after the last IP administration (table S22).

##### G002 skin-related unsolicited adverse events

As described in the main text and summarized in fig. S5 and tables S18 to S21, a subset of participants in G002 experienced skin AEs judged probably or possibly related to administration of study investigational products by site Principal Investigators. These were unsolicited reports, initially received in the 28-day post vaccination period in most cases. During the trial, we developed two case definitions for these events, as follows.

Case definition 1 included Urticaria and/or Angioedema and/or Pruritus and was the broader of the two case definitions. The MedDRA search strategy included: All events with a preferred term (PT) that included the words “Urticaria”, “Idiopathic angioedema”, “Angioedema”, “Rash pruritic”, or any MedDRA PT that contained the word “Pruritus”, excluding cholestatic pruritus, gingival pruritus, neuropathic pruritus, senile pruritus, tumor pruritus and uraemic pruritus. The skin-related AEs in G002 were analyzed using case definition 1. There were no cases of angioedema in G002.

Case definition 2 included Urticaria and/or Angioedema. The MedDRA search strategy included: All events with PT that included the words “Urticaria” (i.e. urticaria chronic, mechanical urticaria, etc), “Idiopathic angioedema”, “Angioedema”. This narrower definition was utilized to provide insight into what were considered to be the more clinically significant skin-related AEs.

Skin-related AEs were considered resolved if there was no reaction present for 14 days without medical treatment. Multiple skin reactions occurring within 14 days were considered part of the same clinical event, and AE, and recorded as such. Any skin AE occurring after 14 days of no symptoms or treatment was considered a new onset AE and recorded as such. Participants were contacted weekly to inquire about changes in symptoms. The characteristics of the reactions were intermittent episodes with waxing and waning symptoms. During the course of any one skin AE, the number of exacerbations (waxing and waning of symptoms during the same clinical event), ranged from 1 to 75. All participants treated the skin AEs with oral antihistamines, and some participants continued to exhibit intermittent clinical symptoms while on antihistamine treatment. A median duration of antihistamine treatment was not calculated or estimated due to the intermittent use.

##### G003 skin-related unsolicited adverse events

As described in the main text, two participants in IAVI G003 experienced pruritus judged probably or possibly related to administration of study investigational products by site Principal Investigators. These were unsolicited reports, initially received in the 28-day post vaccination period in most cases.

##### Communications in G002 and G003 regarding skin-related adverse events

Following the onset of the skin AEs in G002, and meetings of the G002 and G003 Safety Monitoring committees, participants in both trials were informed of the events that had occurred to date and asked during the course of a routine study visit whether they had experienced skin AEs that they may have not reported. This resulted in one additional episode of pruritis being reported retrospectively for a previously reported skin AE. The participants were also reconsented in G002, with an updated ICF that included the information on the skin AEs, and in G003 were given a participant letter with this information, which they signed to acknowledge. There was no formal questionnaire on delayed systemic urticaria and other skin AEs as a potential outcome administered at any point in either study. Three of the 14 skin AEs in G002 were reported to occur at points in time after the reconsenting, one of which was pruritus, and two of which were urticaria or dermatographism that lasted >6 weeks. Hence the communication about skin AEs might have increased the rate of reporting, at most by 3 of 14 for all skin AEs or 2 of 6 for chronic urticaria.

##### Pooled analysis of urticaria and angioedema events occurring in Moderna mRNA vaccine trials

Analyses were performed of all the Moderna-sponsored interventional trials that employ the SM-102 LNP formulated for infectious disease vaccines and had the final clinical study report completed (or interim clinical study report submitted to the health authorities) as of Oct 4, 2024. All mRNA-LNP vaccines in these studies were formulated the same way as the mRNA-LNP vaccines in IAVI G002. Studies included in the analyses are listed in tables S25 and S26. We note that the participants in mRNA-1273 studies who received the placebo in the blinded phase could request to take the mRNA-1273 vaccine in the open-label phase. Per the study design, unsolicited AEs of participants in the open-label phase were not collected in the case report form (CRF). Therefore, the open-label phase of these studies was excluded from our analyses. Similarly, the mRNA-1273-P204 Part 1&2 Booster Phase was also excluded from the analysis, because unsolicited AEs were not collected. In total, 29 studies were included for analysis.

The search criteria for urticaria and/or angioedema events was Urticaria high-level term and preferred terms angioedema (including Angioedema, Idiopathic angioedema, Idiopathic histaminergic angioedema, and Intestinal angioedema), according to the Medicinal Dictionary for Drug Regulatory Activities (MedDRA) version 26.1. The safety data for each study were coded in a different MedDRA version but were re-mapped to version 26.1 for this analysis. The following unsolicited outcomes of urticaria and/or angioedema events were analyzed: any unsolicited AE within 28 days, any unsolicited AE up to the data cutoff date or end of the study, onset day of unsolicited AE, and episode duration of unsolicited AE.

The analyses were conducted at the study level following the recommendation specified in the study statistical analysis plan for the data handling. The study analysis results were then pooled to obtain the overall estimation across the studies where all the mRNA products regardless of dose level were pooled into the “mRNA vaccine” group and all the study comparators were pooled into “Non-mRNA vaccine” group. Rates of urticaria and/or angioedema for the “mRNA vaccine” and “Non-mRNA vaccine” groups were calculated from participants included in the safet set across studies. The safety set consisted of all randomized participants who received at least one study injection. Overall estimations of rates were conducted separately for two different sets of pooled studies, where the first set excluded the COVID19 studies in the booster phase and the second set only included the COVID19 studies in the booster phase. The participants in the booster phase of COVID19 studies were previously administered primary series from the Moderna-sponsored study or under EUA. To avoid double counting of AEs from the same participant, the booster phase of COVID19 studies were excluded from the first pooled set. A separate analysis that only included the COVID19 studies in the booster phase was conducted to better understand the risk of urticaria and/or angioedema for participants who had received COVID19 mRNA vaccine primary series in the past. For all analyses, events of urticaria or angioedema were counted regardless of relationship to study vaccination, meaning the reported rates provide upper bounds on the rates related to study vaccination.

##### Urticaria rate versus number of prior Moderna COVID-19 vaccinations in the NextCOVE trial

The analysis was conducted using data from the NextCOVE trial [mRNA-1283 (COVID-19) P301 in table S26], as this study utilized a version of the WHO Drug Dictionary (version 202303 or later) that enables capture of COVID-19 vaccinations received prior to study enrollment. Additionally, the trial required participants to have received at least one dose of a primary series of an authorized or approved COVID-19 vaccine before enrolling. The purpose of this analysis was to assess whether there is any association or observable trend between the rate of urticaria or angioedema and the number of prior Moderna COVID-19 vaccine doses. The rate of urticaria or angioedema was calculated using the search criteria in table S27, and events were counted regardless of relationship to study vaccination, as in the first and second set analyses.

##### G002 immunological sample collection and storage

Small-scale leukapheresis, referred to simply as leukapheresis, was employed in this study, in which 40-60 ml of plasma containing a high concentration of white blood cells were collected, producing approximately 1-2 billion PBMCs after processing. Leukapheresis was performed at two time points for Groups 1 and 2, once at one to five days prior to the second vaccination, and once at study week 16 (approximately 8 weeks after the second vaccination). Leukapheresis was also performed at two time points for Group 3, once at one to five days prior to the third vaccination, and once at study week 24 (approximately 8 weeks after the third vaccination). Leukapheresis was also performed for Group 4, at week 8 (approximately 8 weeks after the first and only vaccination). PBMCs were collected by venipuncture (whole blood with ACD anticoagulant) at weeks −5, 4, and 24 for Groups 1 and 2; at weeks −5, 4, and 8 for Group 3; and at weeks −5 and 4 for Group 4 (Fig. 1A). PBMCs collected by leukapheresis or venipuncture were isolated by density gradient centrifugation and cryopreserved in liquid nitrogen storage units as aliquots of 20×10^6^, 25×10^6^, or 50×10^6^ cells. Ultrasound-guided fine needle aspirations (FNAs) of axillary lymph node(s) were performed approximately 21 days after each vaccination in all Groups. The procedure was performed by a board-certified radiologist using ultrasound guidance to avoid needle insertion into any adjacent structures. Lymph node (LN) FNA samples were cryopreserved in liquid nitrogen storage units and will be analyzed in the future.

##### G003 immunological sample collection and storage

Serum samples were collected in SST tubes by venipuncture at weeks −5, 4, 8, 10, and 16. PBMCs were collected by venipuncture (whole blood with ACD anticoagulant) at weeks −5, 8, 10, 16 and 21 (Fig. 1B). In addition, leukapheresis was performed at one timepoint, 8 weeks post the second immunization for participants enrolled at the Aurum Institute site in South Africa. PBMCs collected by either leukapheresis or venipuncture were isolated by density gradient centrifugation and cryopreserved in liquid nitrogen storage units as aliquots of 20×10^6^, 50×10^6^ or at 100×10^6^cells per vial. Ultrasound-guided fine needle aspirations (FNAs) of axillary lymph node(s) were performed on weeks 3 and 10 post first immunization. The FNA procedure was performed by a board-certified radiologist using ultrasound guidance to avoid needle insertion into any adjacent structures. Mononuclear cell suspensions were prepared from the FNA and cryopreserved in liquid nitrogen. Few mononuclear cells were recovered in most of the participants (less than 2×10^6^ cells) limiting the BCR analysis from these samples. Gene expression profiles have been generated and will be reported elsewhere.

#### G002 and G003 production of protein reagents for assays

Reagents were produced for B cell sorting, BAMA, and SPR for both studies, and additionally for BLI, nsEM, and neutralization for G002. His-Avi-tagged monomeric antigens were produced at the Scripps Research Antibody Core Facility by transient transfection of HEK-293F cells (Thermo Fisher) and purified by immobilized metal ion affinity chromatography (IMAC) using HisTrap excel columns (Cytiva) followed by size-exclusion chromatography (SEC) using a Superdex 75 10/300 GL column (Cytiva). His-Avi-tagged monomeric antigen lots were split into 2 portions. The first portion was biotinylated using BirA (Avidity) following the manufacturer’s instructions and purified again to remove excess biotin using SEC with either Superdex 75 10/300 GL or Superdex 200 Increase 10/300 GL columns (Cytiva). Biotinylated antigens were used as probes for flow cytometry and cell sorting. The second portion of each His-Avi-tagged monomeric antigen lot was not biotinylated and was used for BAMA and SPR analysis. The eOD-GT8 and (for G002) core-g28v2 60mers used in the BAMA assay were produced at the Scripps Research Antibody Core Facility by transient transfection of HEK-293F cells (Thermo Fisher) and purified by Galanthus nivalis lectin affinity chromatography (Vectorlabs) followed by SEC using a Superose 6 16/600 PG column (Cytiva). The 1HQK naked lumazine synthase nanoparticle used in the BAMA assay was produced by LakePharma Inc. in ArcticExpress E. coli cells (Agilent) and purified by sequential SEC using Superdex 75 (Cytiva) followed by Sephacryl S500 (Cytiva). Monoclonal antibodies used for SPR and (for G002) pseudovirus neutralization assays were produced as human IgG1s by GenScript using the TurboCHO expression service. For G002, His-tagged HIV Env trimers used in BLI, nsEM, and SPR assays were produced by transient transfection of HEK-293F cells (Thermo Fisher) and purified by immobilized metal ion affinity chromatography (IMAC) using HisTrap excel columns (Cytiva) followed SEC on a Superdex 200 Increase 10/300 GL column (Cytiva).

#### G002 biolayer interferometry

His-tagged HIV Env trimers were assessed for native antigenicity using biolayer interferometry (BLI). BLI experiments were conducted using an Octet RED96 instrument at a temperature of 24°C as previously described (63) or by using the following protocol. The BLI assay buffer was composed of 0.1% BSA (Rockland, Cat. No. BSA-30) in HBS-EP+ (Teknova, Cat. No. H8022), while the BLI assay regeneration solution comprised 0.85% Phosphoric acid (Sigma-Aldrich, Cat. No. 345245). Prior to use, Octet ProA biosensors (Sartorius, Cat. No. 18-5012) were pre-hydrated in the BLI assay buffer for 10 minutes. Greiner 96-well black microplates (Cat. No. 655209) were loaded with 200 μL per well. The assay plate setup was as follows: Column 1 contained BLI assay buffer, Columns 2 and 3 held HIV trimers at a concentration of 1 μM in BLI assay buffer, Columns 4-10 contained antibodies at a concentration of 5 μg/mL in BLI assay buffer, Column 11 held the regeneration solution, and Column 12 contained BLI assay buffer. The BLI method was executed as follows: A 30-second baseline (Baseline 1) was established in Column 1, followed by a 90-second loading period in Columns 4-10 for antibody loading. Another 30-second baseline (Baseline 2) was set in Column 1, followed by a 180-second association phase in Columns 2 or 3 for antigen binding. A 30-second dissociation phase was initiated in Column 1, followed by a 30-second regeneration step in Column 11. Raw data in .csv format were exported from the Octet Data Analysis software. The antibody binding value was determined by calculating the ratio of the difference between the association step and the end of the second baseline to the difference between the end of the second baseline and the end of the first baseline. The reported binding value represents the ratio between the “binding level” (increase in signal during the association phase) and the “capture level” (increase in signal during the antibody loading phase). A graph illustrating the antibody binding values was generated using Prism software. Similar results were obtained by considering the “binding level” as the increase in signal from the beginning of the association phase to the end of the dissociation phase, but we reported results for the association phase only considering that trimeric avidity likely slowed dissociation of binders.

### G002 serum binding analysis by BAMA

Serum IgG responses were measured via a multiplex antigen panel (i.e., eOD-GT8.1, eOD-GT8.1_KO11, eOD-GT8 60mer, core-g28v2, g28v2_60mer, core-g28v_KO11b, Lumazine synthase, uncoupled/blank) to determine specificity and magnitude in the same manner as in the G001 study (15) (figs. S8 and S10, and tables S31 to S36, S69, and S70). A binding antibody multiplex assay (BAMA) (15, 64–66) was modified by lengthening the primary antibody incubation period from 30 to 120 min for enhanced detection sensitivity of early bnAb precursors. The assay was validated for accuracy, specificity, precision, robustness, and limit of detection/quantitation (LLOD/LLOQ). The LLOQ for detection of early bnAb precursors was 0.0041 to 0.0866 μg/ml. Antibodies were measured at day 0 (day of first vaccination), week 2 (2 weeks post-first vaccination), week 4 (4 weeks post-first vaccination, week 8 (day of second vaccination), week 10 (2 weeks post-second vaccination), week 16 (8 weeks post-second vaccination for groups 1 and 2, day of third vaccination for group 3 participants), week 18 (10 weeks post-second vaccination for groups 1 and 2; 2 weeks post-third vaccination for group 3 participants), week 20 (12 weeks post-second vaccination for groups 1 and 2; 4 weeks post-third vaccination for group 3 participants), and week 24 (16 weeks post-second vaccination for groups 1 and 2; 8 weeks post-third vaccination for group 3 participants). Samples were serially diluted (1:50, 1:250, 1:1250, 1:6250, 1:31250, and 1:156250) and incubated for 120 min with a mixture of carboxylated fluorescent MagPlex microsphere sets (Luminex) that were each covalently coupled to one of the antigens. For G002 vaccine antigen panel testing, antigen-specific IgG was detected using a biotinylated detection antibody to human IgG Fc (Southern Biotech), followed by washing and incubation with Streptavidin PE (BD Pharmingen). Samples were acquired on a Bio-Plex instrument (Bio-Rad), and antibody levels were measured as median fluorescent intensity (MFI) from two wells and then averaged using the mean. The readout was background-subtracted median fluorescent intensity (MFI), where background refers to the antigen-specific plate-level control (i.e., a blank well containing antigen-conjugated beads run on each plate plus detection antibody). Additionally, a blank or reference bead was included to estimate nonspecific antibody binding. Area under the titration curve (AUTC) was used as the magnitude measure of interest. AUTC was calculated using the trapezoid method with truncation in the case of negative background-adjusted MFI minus background-adjusted blank MFI (MFI*) values or MFI* values > 22000. Samples with blank MFI > 5000 were excluded from the analysis. Several controls were used to confirm bead coupling and antigen conformation: GL-VRC01 (germline VRC01 mAb), VRC01 mAb, anti-68× HIS epitope tag (for His-tagged proteins), CH31 mAb, 2G12 mAb, CH65 mAb (influenza-specific/negative control), KG064-nonVRC01c_099 mAb (eOD-GT8-specific) (67), N6-iGL-IgG mAb (eOD-GT8/CD4bs-specific), and S2102_JN-56_nonVRC01c_m11 mAb (core-g28-specific) (26). GL-VRC01 and VRC01 were titrated in each assay and the area under the titration curve values were tracked over time in Levey Jennings control tracking charts to ensure that antigen binding magnitude was consistent across G001 and G002. Parallel testing and bridging was also done according to Good Clinical Laboratory Practice guidelines for other critical reagents used in the assay to ensure consistent results over the time period of testing samples from both trials.

Data analysis was conducted in a similar manner as G001, where the positivity of a response was defined based on background-adjusted MFI values at the screening dilution level (1:50) except lumazine synthase. Antigen-specific positivity thresholds were computed as the maximum of 100 and the 95th percentile of the baseline MFI* by antigen except lumazine synthase, which was set to 100. Samples from post enrollment visits were declared to have a positive binding antibody response by BAMA if they met three criteria:

1. MFI* values were greater than the antigen specific positivity threshold.
2. MFI* values were greater than three times the baseline MFI* values.
3. Background-adjusted MFI values were greater than three times the baseline background-adjusted MFI values.

For differential binding to the CD4 binding site (CD4bs), defined as the difference in AUTC for binding to eOD-GT8 (AUTC_Ref_) and eOD-GT8-KO11 (AUTC_KO_) or core-g28v2 (AUTC_Ref_) and core-g28v_KO11b (AUTC_KO_), the positivity of response was defined as a positive response to eOD-GT8 plus an additional criterion:

- ΔAUTC > 0, where ΔAUTC was defined as AUTC_Ref_ − AUTC_KO._

Group 2 (eOD→core) samples were also tested by BAMA for binding to potential boost candidate trimer antigens 001428-T278M, BGHxB2-T278M, V703-0537-T278M, CNE40-T278M as well as the CD4bs knockout version of each (fig. S60 and table S72). Samples from weeks 0, 16, and 24 were tested against the boost candidate trimer antigens and, as controls, samples from weeks 0, 8, and 16 were tested against group 2 antigens eOD-GT8 60mer, eOD-GT8 monomer, eOD-GT8 KO11 monomer, core-g28v2 60mer, core-g28v2 monomer, and core-g28v2 KO11b (fig. S60 and table S72). All trimers were biotinylated and bound to beads that were covalently coupled to neutravidin. In this case, BAMA was conducted in the same manner as for G001 (15) and G002 (figs. S8 and S10, and tables S31 to S36, S69, and S70), with the following modifications. The same clone of detection antibody was used to detect antigen-specific IgG, except the detection antibody was directly conjugated to PE (Southern Biotech). Due to differences in sensitivity between the PE-conjugated detection antibody used for the boost candidate trimer antigen panel (fig. S60 and table S72) and the biotin-conjugated detection antibody used for G002 vaccine antigen panel testing (figs. S8 and S10, and tables S31 to S36, S69, and S70), MFI and AUTC values from the two data sets cannot be directly compared. To achieve maximum sensitivity for detection of trimer-specific responses, a relaxed positivity criteria was utilized in which antigen-specific thresholds were not applied, and positive responses were expressed as the percentage of week 8, 16, or 24 samples with MFI at least 3-fold higher than at baseline, both before and after blank bead subtraction.

#### G003 serum binding analysis by BAMA

Serum IgG antibody binding responses to different components of the vaccine in G003 (figs. S8 and S9, and tables S31 to S34) were measured using a standardized BAMA (15) that was transferred from the Center for Human Systems Immunology, Duke University and used for G001 and G002 testing. Briefly, fluorescent magnetic carboxylated beads (MagPlex Microspheres, Luminex) were covalently conjugated to the respective antigens, namely whole eOD-GT8 60mer, eOD-GT8 monomer, eOD-GT8-KO11 and lumazine synthase as per manufacturer’s instructions using EDC (1-ethyl-3-(3-dimethylaminopropyl) carbodiimide hydrochloride) and Sulfo-NHS (N-hydroxysulfosuccinimide). Antigens that were not supplied in phosphate buffered saline (PBS) were dialyzed with PBS prior to conjugation. Antigen-conjugated beads were then incubated with serum samples that had been serially diluted at 1:50, 1:250, 1:1250, 1:6250, 1:31250 and 1:156250 for 120 minutes to enable binding of antigen-specific antibodies, followed by incubation with Biotinylated Mouse anti-Human IgG (Southern Biotech) for 30 minutes and Streptavidin Phycoerythrin (BD Pharmingen) for 30 minutes for detection. Beads were washed after each incubation prior to addition of reagents for the next step. Data were acquired using a Luminex 200 platform.

The assay was qualified for accuracy, specificity, sensitivity, precision and robustness using standard monoclonal antibodies and serum samples from healthy unvaccinated individuals. The transfer of the assay to the KAVI Institute of Clinical Research was also cross-validated against the transferring laboratory at the Center for Human Systems Immunology at Duke University with reproducibility analyses on shared pre-vaccination and postvaccination serum samples from the G001 clinical trial (15) where the immunogen eOD-GT8 60mer had been administered. The lower limit of detection of the assay for detection of VRC01-class antibodies, as determined using germline-reverted VRC01, was <0.0015 for eOD-GT8 60mer beads and 0.0690 for eOD-GT8 monomer beads. In the inter-lab cross-validation, the comparison of G001 response calls for postvaccination samples yielded similar outputs for eOD-GT8 60mer, eOD-GT8 monomer, eOD-GT8.1-KO11 (Cohen’s Kappa =1.00). Likewise, Lin’s concordance correlation coefficient (CCC) was high, at >0.92, for eOD-GT8 60mer, eOD-GT8 monomer, eOD-GT8.1-KO11, suggesting good comparability of the assay across the two laboratories (fig. S9). Concordance for eOD-GT8 CD4bs (CCC=0.3965) and lumazine synthase (CCC=0.8296) was lower between the laboratories (fig. S9), hence we did not make statistical comparisons involving CD4bs or LS between G003 and other trials.

BAMA analyses in G003 were carried out using the same procedures as in G001 (15) and G002. Here we describe these procedures. Net mean fluorescence intensity (MFI), defined as the Log10 background- and blank-subtracted MFI, was used to evaluate response magnitudes in the G003 study participants. Values were truncated at 1 (for values <1) and 22,000 (for values >22,000, because they were considered saturated). Area under the titration curve (AUTC) was calculated using the trapezoid method. Samples with blank MFI>5000 were excluded. Graphical distributions of response magnitudes for the AUTC were plotted on a log10 scale for each timepoint showing distribution of positive responders. Pairwise comparisons of AUTC between visits were done for timepoints that had at least 3 positive responders using the Wilcoxon rank-sum test (two-sided, α = 0.05) on the positive responders only.

Background-adjusted MFI of the screening dilution (1:50) was used for defining the positivity of a response, except for lumazine synthase that had high baseline values at the screening dilution. The threshold for positivity for each antigen was computed as the 95th percentile of the baseline net MFI by antigen. Post-enrollment samples were declared to have a positive response if they met 3 criteria: (1) the net MFI at the screening dilution were greater than the maximum of 100 or the 95th percentile of baseline samples by antigen, (2) the net MFI values were greater than 3 times the baseline net MFI values, and (3) the background-adjusted MFI values were greater than 3 times the baseline background-adjusted MFI values. For the lumazine synthase, the response call was made at the first dilution for which the baseline net MFI was <6500 and the antigen specificity threshold was set at 100. For differential binding to the CD4 binding site, defined as the difference in AUTC for binding to eOD-GT8 monomer and eOD-GT8.1-KO11, the positivity of response was defined as a positive response to eOD-GT8 monomer plus a difference in AUTC that was greater than zero. Response rates (percentage of participants making a positive response) were plotted with accompanying 95% Wilson score confidence intervals for each timepoint and antigen.

Samples were excluded from the analyses if the blank MFI for the corresponding negative control was greater than 5000. Records were excluded at all visits if any of the following occurred at baseline: (1) missing values for MFI, blank MFI, or net MFI (2) high background, i.e., blank MFI for negative control >5000 or (3) high baseline MFI or net MFI>6500. AUTC records were excluded if any of the dilutions were excluded.

#### G002 B cell sorting and BCR sequencing methods

The aim of B cell analyses was to identify B cells responding to eOD-GT8 or core-g28v2 and further subset their specificities based on whether they targeted the CD4bs-epitope region on these molecules or off-target sites on the molecules. We accomplished this by first labelling eOD-GT8, core-g28v2, and their respective CD4bs-knockout protein variants to fluorochromes and fitting them into a flow cytometry B cell phenotyping panel. Fluorescently labeled eOD-GT8 and the eOD-GT8 CD4bs knock out protein (eOD-GT8-KO11) are together referred to as the eOD-GT8 probe-set. Fluorescently labeled core-g28v2 and the core-g28v2 CD4bs knockout protein (core-g28v2-KO11b) are together referred to as the core-g28v2 probe-set.

Using flow cytometry, we detected B cells responding to eOD-GT8 or core-g28v2 (antigen^++^ B cells) and further distinguished those B cells that targeted the CD4bs on these molecules based on differential staining to CD4bs-knockout proteins. Those B cells that were able to bind eOD-GT8 or core-g28v2 but not their respective CD4bs-knockout proteins, were identified as CD4bs-specific B cells (CD4bs^++^ B cells). Those that bound eOD-GT8 or core-g28v2 and their respective CD4bs-knockout proteins were considered off-target specific B cells.

We identified B cells with these different specificities within naïve IgD^+^ B cells, IgD^−^ B cells, IgG^+^ memory B cells, IgA^+^ memory B cells and IgM^+^ memory B cells in PBMC samples. We sorted and sequenced B cells that recognized the CD4bs-epitope region using the eOD-GT8 probe-set or the core-g28v2 probe-set. From PBMC collected at the pre-vaccination timepoint, we sorted CD4bs-specific B cells from both the IgD^+^ and IgD− compartments for sequencing. From PBMC collected at post-vaccination timepoints, we sorted CD4bs-specific B cells from the IgD- compartment for sequencing. The total numbers of PBMCs processed from the different groups and timepoints are indicated in table S37, along with the probesets with which they were processed. Methods were established, optimized and all samples were processed and sequenced at the Vaccine Research Center, NIH.

##### G002 probe preparation

Tetramer probes were prepared at 4:1 molar ratio of streptavidin-fluorochrome to monomeric eOD-GT8, eOD-GT8-KO11, core-g28v2 or core-g28v2-KO11b. The total volume of protein and 1x PBS were added to a microfuge tube. Twenty percent of the total streptavidin volume was added, mixed and incubated in the dark at 4°C for 20 minutes. Incremental addition of streptavidin was repeated 4 times until the total amount of streptavidin had been added to the protein. Tetramers were made and stored at 4°C and used for up to 2 weeks.

##### G002 probe QC

Bead assays were performed on the day samples were processed to confirm functionality of probes by flow cytometry. Two tests were set up for each probe (one experimental and one control). Anti-mouse Ig-kappa beads (50ul) were mixed with an equal volume of R10 media (RPMI 1640 containing 10% fetal bovine serum and 1% penicillin-streptavidin - Thermo Fisher Scientific, Waltham, MA) in polystyrene FACS tubes for each test. 1μg of mouse anti-human IgG (BD Bioscience, La Jolla, CA, USA) was added to each tube and incubated at 4°C for 15 minutes. Beads were washed with R10 and resuspended in 100μl of R10. To the experimental, but not control tubes, 1μg of linker antibody was added. Linker antibodies were germline-VRC01 for eOD-GT8, KG064-nonVRC01c_099 (67) for eOD-GT8-KO11, mature-VRC01 for core-g28v2 and S2102_JN-56_nonVRC01c_m11 (26) for core-g28v2-KO11b. After incubation at 4°C for 15 minutes, beads were washed and resuspended in 100μl of R10. Probes were added to experimental and control tubes and incubated at 4°C for 15 minutes. Beads were washed with R10 and resuspended in 200μl of R10 for collection.

##### G002 sample batching schemes

PBMC samples were prescreened to determine frequencies of CD4bs-specific B cells to optimize sorting and sequencing (figs. S12A and S13). Based on CD4bs-specific B cell numbers observed during prescreening, samples were batched strategically such that optimal CD4bs-specific cell numbers were bulk sorted on experimental days for downstream 10x Genomics sequencing (fig. S12A and table S37). We targeted to collect 800 - 8000 cells per pool with no more than 8 pools of epitope+ cells on a single day sorted from clinical samples. Typically, longitudinal samples from donors were batched together for sorting.

##### G002 sample processing workflow

For prescreening and sorting, PBMC samples were enriched for B cells and stained with a B cell phenotyping panel and fluorescently labeled probes. B cell enrichment was incorporated into the PBMC sample-processing workflow to eliminate non-specific binding and T cell binding to probes (fig. S12A). For sorting, PBMC samples were also barcoded with hashtags to allow for multiplexing.

##### G002 B cell enrichment

Samples were enriched for B cells based on negative-selection using the StemCell pan-B cell enrichment kit (StemCell Technologies; Vancouver, CA) using the manufacturer’s instructions. In brief, cryopreserved samples were thawed using Cryothaw adaptors (Medax USA) into warm R10 containing benzonase (Thermo Fisher Scientific, Waltham, MA). Supernatants were decanted and cells were washed with EasySep buffer. After the wash, cells were resuspended in 1-2ml of EasySep buffer for B cell enrichment according to the manufacturer’s instructions. Volumes of enrichment cocktail and magnetic beads added per sample were adjusted based on cell numbers processed.

##### G002 barcoding with hashtags

For barcoding with hashtags, cells were washed once with PBS containing 0.1% BSA. Following the wash, 10μl of Fc Blocking reagent and 1μl of commercial hashtag product (Biolegend, San Diego, CA) were added to cells and mixed. Cells were immediately placed on ice for 15 minutes and then washed with PBS.

##### G002 flow cytometry staining with phenotyping markers and probes

Cells were incubated for 2 minutes with viability blue fluorescent reactive dye (Thermo Fisher Scientific, Waltham, MA) added at 1:60 dilution at room temperature to stain dead cells. Following incubation, cells were stained with a panel of fluorescently labeled cell markers (table S38) along with CD4bs knock-out probes for 30 minutes in the dark at 4°C, washed once with R10, and stained with eOD-GT8 or core-g28v2 probes for another 30 minutes in the dark at 4°C. After incubation, cells were washed twice with R10, and resuspended in R10 for collection.

##### G002 flow cytometry gating

Standardized gating templates were designed using the BD Diva software for analysis of PBMC samples. These templates were replicated for each prescreen and sort experiment to ensure consistent gating of samples. The M.A.R.I.O gates (15) were implemented to identify eOD-GT8 and core-g28v2 specific B cell subsets (figs. S13 and S14).

##### G002 FACS

CD4bs-specific B cells identified using the eOD-GT8 probe-set or the core probe-set were bulk sorted into collection tubes for downstream encapsulation with 10x Genomics Gel Beads and subsequent sequencing. At the pre-vaccination timepoint, CD4bs-specific B cells from both the IgD^+^ compartment and IgD^−^ compartment (CD19^+^, CD20^+^, IgD^−^ or IgD^+^) were sorted (figs. S13 and S14). For post-vaccination timepoints, CD4bs-specific B cells from the IgD- B cell compartment (CD19^+^, CD20^+^, IgD^−^) were sorted (figs. S13 and S14). Cells were sorted into collection tubes containing R10.

##### G002 positive control

Commercially available Ramos cells (ATCC) were used as a positive control in every pool sorted. Ramos cells were expanded and cryopreserved in 5 million aliquots for use as positive control samples on every sort day. On a sort day, cryopreserved Ramos cells were thawed using Cryothaw adaptors and transferred into warm R10 media containing benzonase (Thermo Fisher Scientific, Waltham, MA). Cells were pelleted, washed once with R10 and once with PBS containing 0.1% BSA and barcoded with hashtags as described above. After the PBS wash, cells were stained with the viability blue fluorescent reactive dye (Thermo Fisher Scientific, Waltham, MA) for 2 minutes at room temperature at 1:60 dilution. Cells were then washed twice with R10 and resuspended in R10 for collection. One hundred viable Ramos cells were sorted into every pool of cells collected from the trial.

##### G002 B cell sequencing

Bulk sorted epitope^+^ B cells were processed using the Chromium Next GEM Single Cell 5’ Reagent Kit v2 with Feature Barcoding technology according to the manufacturer’s instructions. Briefly, DPBS (Gibco 14190-144) with 0.04% BSA (Sigma A7030-10G) was added to the collection tube with sorted cells to adjust the total volume to 38.7ul. The cell suspension was mixed well with 36.7μl of the reverse transcription (RT) master mix before loading onto a Chromium Next GEM Chip K (10X Genomics PN-1000286). Barcoded Single Cell VDJ 5’ Gel Beads (10X Genomics PN-1000264) and partitioning oil were also loaded into the Chip K, which was then run in a Chromium Controller or Chromium X to form single cell partitioning in Gel bead-in-emulsions (GEMs). In the following RT reaction, complimentary DNA (cDNA) from the single cells’ mRNA and hashtag DNA oligo bound to the cell surface were labeled with unique cellular barcodes. The PCR amplified total cDNA mix was separated into cell hashtag DNA oligo derived cDNA and cellular mRNA derived cDNA components. 3μl of cell hashtag cDNA was utilized directly as template for cell hashtag library construction and indexed via Dual Index Kit TN (10X Genomics PN-1000250). Cellular gene transcript cDNA was used as template for V(D)J amplification via the Chromium Single Cell Human BCR Amplification Kit (10X Genomics PN-1000253), and V(D)J libraries were constructed and indexed with Dual Index Plate TT Set A (10X Genomics PN-3000431). The purified libraries were quantified using Qubit^®^ dsDNA HS Assay (Invitrogen, Q32854) and Bioanalyzer High Sensitivity DNA (Agilent, 5067-4626) and normalized to 2nM before combining for sequencing. The pooled libraries were diluted to 800pM by Nextseq 1000/2000 RSB with Tween 20 (Illumina, 20050639) and then paired-end sequenced via Illumina Nextseq2000 on P2 (Illumina 20046811) or P3 (Illumina 20040560) 100 cycles kits with the sequencing read lengths set to 26bp for read 1, 10bp for index 1, 10bp for index 2 and 90bp for read 2. For both VDJ libraries and cell hashtag libraries, we targeted at least 20,000 reads per cell.

#### G003 B cell sorting and BCR sequencing methods

Prevaccination and postvaccination B cells were sorted to identify antigen-specific (eOD-GT8-specific) and epitope-specific (CD4bs-specific) B cells. The flow cytometry and cell sorting were carried out on a FACS Melody instrument, and a B cell phenotyping panel (table S39) was set up to identify B cell subsets, antigen- and epitope-specific memory B cells. As the sorting instrument employed here was different from that used in G002, we considered the following steps to ensure comparability: 1) the baits preparation procedure (conjugation to fluorescent streptavidins) was optimized for maximum signal to noise ratio and assessed for stability using beads coated with control mAbs (VRC01-GL, AB099); 2) the sensitivity, accuracy and precision of target cell detection was determined using random PBMC samples from healthy individuals spiked with a VRC01-cell line; 3) BD Melody sorter Instrument Qualification was conducted by the manufacturer to ensure instrument concordance between the sorter at the KEMRI lab and a reference laboratory at the IAVI Neutralizing Antibody Center (NAC) at Scripps Research; 4) voltages standardization and calibration processes were established using BD FC beads to ensure concordance and stability of fluorescent signals across experiments and across instruments at KEMRI and the NAC, and 5) the M.A.R.I.O. gating strategy developed for G001 (15) and also used for G002 was adopted for G003 to ensure comparability of antigen-specific B cell frequency data throughout the trial.

Cells of interest were identified using fluorophore-conjugated antibodies against B cell subset markers as well as fluorophore-conjugated eOD-GT8 and eOD-GT8 knock-out (eOD-GT8 KO11) baits (15). B cells considered to be CD4bs-specific were those that bound to the eOD-GT8 probe on two separate fluorophores (‘double-positive’ cells) but did not bind the eOD-GT8 KO11 probes. B cells that bound both the eOD-GT8 and eOD-GT8-KO11 probes were considered off-target. By flow cytometry, we assessed epitope-specific cells within naïve (IgD^+^) B cells, and memory (IgD^−^) B cells. For the memory B cells, we specifically identified IgG^+^ memory B cells and IgM^+^ memory B cells and grouped IgA and IgE as IgM^−^/IgG^−^ memory B cells due to limited fluorophore options on the FACS melody. We then sorted and sequenced the IgD^−^ B cells population that recognized the CD4bs-epitope region. In G003, the number of PBMCs analyzed at weeks −5, 8, 10, 16 and 21 were, on average, 300, 200, 100, 200 and 200 million, respectively. The numbers of IgD^−^ B cells, IgG^+^ B cells, eOD-GT8^++^ IgG^+^ B cells, and eOD-GT8 CD4bs-specific IgG^+^ B cells sorted per participant per timepoint are shown in fig. S21.

##### G003 probe preparation and QC

To prepare the eOD-GT8 and eOD-GT8-KO11 probes, biotinylated eOD-GT8 and eOD-GT8-KO11 monomers were combined with fluorescently labeled streptavidin (SA-Alexa fluor647, SA-Alexa fluor488 or SA-Brilliant Violet 421) at a 4:1 molar ratio. First the monomers were mixed with an appropriate volume of 1xPBS in a microcentrifuge tube then one-fifth of the volume of the respective labeled streptavidin was added and the preparation incubated at 4°C for 20 minutes in the dark. This was repeated four times until the entire volume of streptavidin was added. Prepared probes were stored at 4°C for a maximum of two weeks.

 The performance of the probes was then tested in a bead assay. Briefly, 50 μL of Anti-mouse Ig-kappa beads were added to an equal volume of R10 media (RPMI-1640 containing 10% heat-inactivated fetal bovine serum and 1% Penicillin-Streptomycin [Pen-Strep]) in a FACS tube before 1μg of mouse anti-human IgG was added to each tube and incubated at room temperature (RT) for 15 min. The beads were then washed with 3 mL of R10 then resuspended in 100 μL of R10 and 1μg of linker antibody (germline-VRC01 for eOD-GT8 and KG064-099 for eOD-GT8-KO11) added followed by a 20-min incubation at RT. After incubation, the beads were washed as above and resuspended in 500 μL of R10 and stored at 4°C for a maximum of 2 weeks. The mAb-conjugated beads were then stained with baits to test their functionality. First, 50 μL of the conjugated beads were added to a FACS tube then 20 μL of R10 and 10 μL of the eOD-GT8-KO11 probe were added before incubating at 4°C for 15 minutes. Ten microliters of each eOD-GT8 probe (coupled either to AF647 or AF488) was premixed then added to the conjugated beads and incubated for a further 15 minutes. One drop of BD^™^ CompBeads Negative Control was then added to the stained beads and the beads washed with 3 mL of R10. The beads were then resuspended in 300 μL of R10 and acquired on the FACS melody.

##### G003 sample batching scheme

The target number of cells per pool was between 800-8000 and a maximum of 5 pools per sort. To estimate the number of samples needed to meet this sorting criteria, PBMC samples from the week 10 post-vaccination time-point were pre-screened to determine the CD4bs-specific B cell frequencies for each participant. Participants were then batched for sorting based on the expected number of epitope-specific cells (sort pools). This sorting strategy resulted in samples from weeks 10 and 16 being sorted independently while samples from weeks −5, 8 and 21 from one participant were combined for sorting on the same day and when necessary, were pooled into one sort-pool to retain the number of cells per pool between 800-8000 (fig. S12B). The PBMC samples were barcoded with TotalSeq-C anti-human hashtags prior to pooling to allow for multiplexed analysis.

##### G003 B cell enrichment, flow cytometry and sorting of epitope-specific memory B cells

Cryopreserved PBMC samples were rapidly thawed in a pre-warmed water bath set at 37°C then quickly transferred to pre-warmed R50 media (RPMI-1640 containing 50% heat-inactivated fetal bovine serum [HIFBS]). The PBMCs were then spun down, supernatants decanted and the PBMCs resuspended in FACS buffer (2% HIFBS, 25 mM HEPES buffer, 2 mM EDTA and 1xPBS). PBMC counts were then estimated using an automated cell counter (ViCell XR) before concentrations were adjusted to 5 x 10^7^ cells/mL for B cell enrichment. B cell enrichment was done based on a negative selection approach, using the EasySep^™^ Human Pan-B Cell Enrichment Kit according to the manufacturer’s instructions. The enriched B cells were counted to determine the volume of antibody to use for staining. We used a final staining volume of 100 μL for every 10 - 20 million B cells. For the 100 μL staining volume: 5 μL of antibody, 2 μL of hashtag and 10 μL of probe were used. The enriched B cells were first pelleted by centrifugation and the supernatant carefully aspirated before a staining cocktail containing a unique hashtag, fluorescently labeled cell markers (table S39) and the eOD-GT8 KO11 probe was added. The cells were then incubated on ice for 15 minutes in the dark. After 15 minutes, a premix of the eOD-GT8 probes was added, followed by a 30-minute incubation on ice and in the dark. After this a 1:300 dilution of viability dye (Zombie Aqua^™^ amine-reactive fluorescent dye) was then added at a ratio of 1 mL for every 10 – 20 million B cells and the cells incubated on ice for 30 minutes in the dark. Ten milliliters of FACS buffer were then added to wash the cells before resuspending them at a concentration of 15,000 cells/μL in FACS buffer for collection.

##### G003 flow cytometry gating and FACS

PBMC samples from random healthy, HIV-uninfected blood donors were stained with the full B cell phenotyping panel then acquired on the FACS Melody to set population gates to identify the B cell subsets of interest and define the M.A.R.I.O gates (15), to identify eOD-GT8-specific B cells. Owing to the low MFI of the AF488 fluorophore observed, the M.A.R.I.O gate was modified slightly by dropping the AF488 voltage by half a log as this ensured that the ‘double-positive’ CD4bs-positive population was fully captured. The modified M.A.R.I.O gate was maintained throughout the study to ensure consistent gating of the epitope-specific B cells. A positive control ‘bridging sample’ was run to confirm that all populations were clearly identified within the set gates, then the gates and experiment settings were saved in an experiment template used throughout the study. The two bridging samples used in this study were derived from the AURUM week 16 post-vaccination time-point from individuals for whom a large volume of PBMCs were harvested by leukapheresis. The bridging samples were also run: 1) after updating the compensation standards on the FACS Melody (this needs to be updated every 60 days), and 2) when the experiment templates had to be re-created after servicing of the FACS melody sorters either during preventative maintenance or after a machine breakdown.

##### G003 monitoring of inter-day variation

After the initial experiment set up, BD compensation beads and BD FC beads were acquired on the FACS melody and the MFI for each fluorophore determined. The FC beads were run to check the FACS Melody stability on each day while the compensation beads were run as a daily test of the fluorophore-conjugated antibodies used for staining to ensure that they gave reproducible MFIs. To determine reference ranges of MFI for each fluorophore, compensation beads stained with each fluorophore and FC beads were run on 10 consecutive days and MFIs recorded. Upper and lower limits of MFI were defined as the upper quartile (Q3) plus 1.5 x the interquartile range (IQR) and the lower quartile (Q1) minus 1.5 x IQR, respectively. A decision tree was used to determine the course of action to take if an MFI value for a particular fluorophore for either the FC or compensation beads fell outside of the reference ranges. This information was used to guide when to update the compensation settings on the FACs melody, and when to use a new reagent tube of a fluorophore-conjugated antibody. Both the compensation beads and FC beads were run on each sort day prior to staining and sorting, as well as for routine checks when no sorts were planned.

##### G003 FACS

CD4bs-specific B cells from the IgD^−^ B cell compartment were bulk sorted into wells of a 96-well plate containing 20 μL of 100% HIFBS for downstream preparation of single-cell suspensions using the 10x Genomics platform. The flow rate was adjusted during the sort to between 6000 – 8000 events per second to ensure a sort efficiency of ≥ 70%. As a positive control, 100 RAMOS cells (sourced commercially from ATCC) were also sorted into each well containing epitope-specific B cells on every sort day. The RAMOS cells were thawed and counted in a similar manner to the PBMC samples but were only stained with a unique hashtag and viability stain before sorting. Immediately after the sort, 200 μL of sterile 1xPBS was added to the wells containing the sorted cells and the 96-well plate was then spun down briefly at 2500 rpm for 2 minutes. The PBS was then partially aspirated to leave 38.7 μL of suspension in the wells.

##### G003 single-cell library preparation and sequencing

Single-cell suspensions of the epitope-specific B cells were prepared using the Chromium Next GEM Single Cell 5’ Reagent Kit v2 with Feature Barcoding technology according to the manufacturer’s instructions. Briefly, Gel-beads-in emulsion (GEMS) were prepared by adding 36.7 μL of reverse transcription (RT) master mix to the 38.7 μL of suspension containing the epitope-specific cells and mixing gently with a wide-bore tip. The cells were then loaded onto a Chromium Next GEM Chip K along with Barcoded Single Cell VDJ 5’ Gel Beads and partitioning oil and the chip run in a Chromium Controller to form the GEMS. After recovery of the GEMS, an RT reaction was carried out to prepare cDNA. V(D)J genes were amplified from the fraction of cDNA prepared from cellular mRNA and used to prepare V(D)J libraries. On the other hand, cell hashtag libraries were constructed from the cell hashtag oligonucleotide-derived cDNA. The purified libraries were quantified by qPCR using the KAPA Library Quantification Kit for Illumina^®^ Platforms and average library sizes estimated using the Agilent TapeStation platform. Libraries were then pooled targeting at least 5000 reads per cell for both V(D)J libraries and cell hashtag libraries. B cell sorting and 10x library preparation were carried out at Kenya Medical Research Institute (KEMRI). Sequencing of the libraries was outsourced to a service provider (International Livestock Research Institute, ILRI) where the libraries were sequenced on a NextSeq 500 using a 150-cycle high output kit with read lengths set to 26 base pairs (bp) for read 1, 10 bp for index 1, 10 bp for index 2 and 90 bp for read 2.

#### G002 IGHV1-2 genotype analysis using IgDiscover

For G002, individualized IGHV genotypic analysis was performed on two independent IgM libraries prepared for each case, the leader library (L), and upstream library (U), named after the target location of the 5’ multiplex primer sets in either the leader exon (L) or the upstream 5’ UTR region (U). Both sets of libraries were prepared according to the protocol described previously (68) prior to sequencing with the Illumina MiSeq 2 x 300 cycle V3 kit. The sequence data for all IgM libraries was analyzed with the germline inference program IgDiscover (69) and additionally with the Ig genotyping tool, corecount (70), as described in deCamp et al. 2024 (17), to identify the IGHV1-2 genotype, with agreement between all four outputs: L library IgDiscover, L library corecount, U library IgDiscover and U library corecount; being required to call the IGHV1-2 genotype.

#### G003 IGHV1-2 genotype analysis using IgDiscover

The G003 trial participant who did not produce VRC01-class responses was typed for IGHV1-2 as described previously (15). In brief, 200ng total RNA, extracted from PBMCs, was used as template to produce UMI containing IgM libraries. These were subsequently indexed and sequenced on the Ilumina Miseq platform using Illumina V3 2 x 300 cycle kit. The resultant libraries were processed with the germline inference tool IgDiscover (69) and genotyping program corecount (70) to determine the IGHV1-2 allelic content, as previously described in deCamp et al., 2024 (17).

#### G002 bioinformatic BCR sequence processing

To measure the frequencies of VRC01-class B cells and other B cell subsets, and to enable detailed analyses of sequence features for VRC01-class and non-VRC01-class BCRs, a bioinformatic pipeline was needed. Although we previously developed a computational pipeline to analyze single B cell sorting and Sanger sequencing data for IAVI G001 (15), the change in laboratory BCR analysis methodology in IAVI G002 to bulk B cell sorting and droplet-based RNAseq necessitated development of a new computational pipeline. We developed a new pipeline for IAVI G002 bioinformatic processing, termed G00x-G002, that was designed to adaptable for additional trials.

At a high level, the G00x-G002 pipeline: (a) parsed and validated input files from flow sorting and 10x Genomics sequencing; (b) converted raw BCL sequencing data into fully annotated paired BCR heavy and light chains associated with cell count data and other clinical trial meta data such as participant, group, timepoint and sorting probe; (c) calculated frequencies of VRC01-class B cells and other B cell subsets; and (d) combined all analyses into a DataFrame (CSV file) that allowed for data graphical display and additional bioinformatic analyses. Within this pipeline, BCR V gene assignments and SHM were determined using Sequencing Analysis and Data library for Immunoinformatics Exploration (SADIE, https://github.com/jwillis0720/sadie), and paired BCR heavy and light chains and their characteristics were stored in the standardized Adaptive Immune Receptor Repertoire (AIRR) format (71). Mutation analysis for VH1-2 genes was made more accurate by accounting for personalized IGHV1-2 genotypes determined through separate analysis of personalized IgM libraries and provided as additional inputs. The entire bioinformatics pipeline was packaged, scripted and documented in a private, password-protected repository that will be made public upon manuscript publication (https://github.com/schieflab/G00x-G002). For G002, all analysis steps were performed under the “g00x g002” subcommand. Details of individual stages of the pipeline are described in the next sections.

##### G002 flow data

The VRC lab collected flow data and deposited it into folders labelled with sort date and upload date on Box, a secure filesharing service, with the following directory structure:


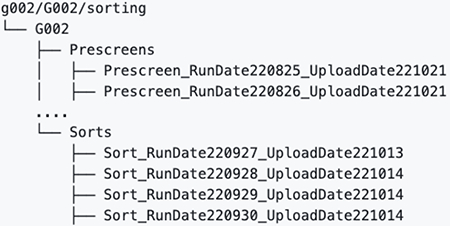


Using RClone, flow data folders stored on Box were mounted on an AWS EC2 instance and copied over locally (fig. S17A). We then used the command “g00x g002 validate” to verify the meta data associated with each folder by looking at the labeled population files that had been generated from Diva. The CSV files with the sorting information had the following naming structure:

<run purpose>_<run date>_<sort id>_<ptid>_<visit id>_<sort probe>_<sample type>_<hashtag>_<sort software>_<sort file type>_<tube id>_<pool id>_<pool subset id>.csv.

The CSV file was programmatically checked for correct sort dates, ptids, visit ids, sort hashtags and tube numbers and ensured that the data entries complied with strict formatting rules set in place by the VRC lab. A spreadsheet showing the flow data manifest architectures used is given in Data S6.

##### G002 sequencing data

For the sequencing data, a folder was uploaded with Globus (fig. S17A), as raw Illumina BCL under the run_id, denoted run####, with a sequencing manifest in a CSV for each individual run. The sequencing data were validated to contain expected data formats in the following sequential folders run0002, run003, … run0032 which corresponded to each sequencing run. The sequencing data had the following structure:


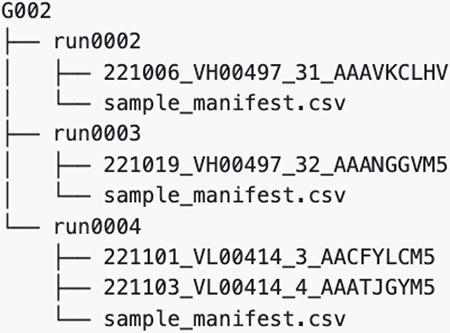


The sample_manifest.csv contained the following fields: pool_number, sorted_date, vdj_index, feature_index, vdj_run_id, cso_run_id. The data was joined with the flow data to get ptid, sorting probe and visit by joining on pool number and sorted date to make a master Pandas (https://github.com/pandas-dev/pandas) DataFrame that contained the locations of all files. This DataFrame was output as merged.csv or merged.feather. An example merged DataFrame is provided in Data S7.

##### G002 demultiplex BCL

For each entry in the merged DataFrame, we demultiplexed the raw Illumina base call files (BCL) using cellranger version 6.1.2 that was wrapped in the command “g00x g002 pipeline demultiplex” (fig. S18A). This command called on “cellranger mkfastq” and output fastq files to a specified directory named demultiplex.feather that was saved in our DataFrame. The specified fastq files were either VDJ libraries or cell-surface oligo (CSO) libraries that were identified by the Illumina index.

##### G002 VDJ and CSO

For the VDJ libraries, the command “g00x g002 pipeline vdj” was used to call on “cellranger vdj” which in turn generated a VDJ output for each sample or mixture of samples to be demultiplexed with the CSO command. The VDJ output was saved in the DataFrame and output as vdj.feather. We next called on “g00x g002 pipeline cso” which in turn called on “cellranger count” to count each hashtag (feature index, the unique oligo associated with each experimental sample, e.g. participant and timepoint) associated with a cellhash (the unique oligo associated with each cell). This feature-index DataFrame had its location saved as cso.feather. The “cellranger count” command was used instead of the “cellranger multi” command to reduce downstream code and DataFrame complexity.

##### G002 AIRR

To fully demultiplex the samples using CSO and VDJ libraries, we invoked the command “g00x g002 pipeline airr” which first ran the VDJ libraries through SADIE (www.github.com/jwillis0720/sadie.git) which saved an AIRR-compliant DataFrame. This resulting intermediate DataFrame was used to pair the IGH locus (heavy chain) sequences with the IGK or IGL locus sequences if they only had one heavy chain-light chain pair. The airr command also obtained the feature index counts of each cell surface oligo and joined them back on to the paired VDJ to determine what sample each cellhash belonged to. The feature index was counted per cellhash and was discarded if there were fewer than 100 counts of the sum of the feature index. For a cellhash to claim a feature index, that feature index had to represent 95% of the feature index counts initially associated with the cellhash. A strict 95% threshold was used to avoid doublets or multiplets and optimize the probability of capturing the correct feature index. The airr command also personalized each participant sample by the IGHV1-2 genotype determined experimentally by IgDiscover. For example, for a participant with an experimentally determined IGHV1-2 genotype of *02/*05, the code would restrict the reference database to IGHV1-2 alleles *02 and *05 only, to prevent miscalls to other IGHV1-2 alleles.

Next, the “g00x g002 airr” command identified somatic mutations and recorded them in the DataFrame in Kabat numbering. A BCR was considered a VRC01-class BCR if the sequence had IGHV1-2 allele *02 or *04 and an LCDR3 length of 5; in this case, is_vrc01_class was marked as True in the DataFrame. The number of key VRC01-class HC residues was then counted. As previously described, key VRC01-class HC residues were counted on a scale ranging from −4 to +16 to allow for all possibilities from losing all four germline-encoded key residues to gaining key residues at all 16 positions not containing a germline-encoded key residue (15, 26). The heavy chain (HC) residues considered key VRC01-class HC residues were identified by the intersection between the Kabat mutations and a list of all key VRC01-class heavy chain residues (15, 26). The number of Kabat mutations in common with the list of all possible key VRC01-class HC residues was counted, and the number of Kabat mutations that mutated away from a germline-encoded key HC residue was subtracted, to give the total count of key VRC01-class HC residues.

Next, the AIRR command clustered all sequences. Sequences were grouped by HCDR3 length and LCDR3 length and the Boolean is_vrc01_class, and within each group the Levenshtein distance across HCDRs 1 to 3 and LCDRs 1 to 3 was measured between each pair of sequences. An average linkage cluster distance of 5 was used to assign clusters of each sequence. The automatic clustering carried out by the airr command was used to provide initial guidance for subsequent more refined clustering strategies.

All the above information was recorded in the sequence DataFrame and saved to a final_df.feather.

##### G002 flow report

A flow DataFrame was also recorded with the command “g00x g002 pipeline flow”. This command parsed the population sort files uploaded from the VRC lab and recorded which gates corresponded to which sort population and save the information as flow.feather DataFrame. Each flow run is represented as their own individual row in the flow.feather DataFrame and includes fields to allow a complete traversal to the original sort run. A list of all gates for G002 is provided in Data S8.

##### G002 analysis Report

The “g00x g002 analysis report” subcommand took in the sequencing (final_df.feather) and flow (flow.feather) DataFrames to make frequencies from the flow and sequencing DataFrame into a parsable CSV file. The flow frequencies were recorded by taking as the numerator the events of a sort-specific population (e.g. epitope-specific cells) and taking as the denominator the events of a B-cell population (e.g. IgG^+^). The resulting counts and frequencies were used as the core metrics for additional analysis and visualizations. A list of frequencies computed for G002 is provided in Data S9.

With this pipeline we analyzed data from 358 flow runs and 342 sequencing runs, and the pipeline output 158,267 BCR sequences of all isotypes with associated AIRR characteristics, including 121,571 IgG BCR sequences.

#### G003 bioinformatic BCR sequence processing

The bioinformatics G00x-G003 pipeline was built on the same codebase as G00x-G002 but with several changes. These changes encompass syncing sequencing and flow data directly to an S3 bucket using the AWS CLI, flow directory names changed, removing hashtags from sorting data file nomenclature, reducing the number of unique field values in flow data, updating CellRanger to version 7.2 for each step of the pipeline, merging sequence data with flow data as a separate step, and allowing the input and output of each pipeline step to be located outside of the main pipeline directory path. In addition, for the analysis of SHM in the VH1-2 gene, whereas in G001 (15) and G002 this SHM was computed using germline VH1-2 alleles inferred from personalized VH1-2 genotypes from each participant, in G003 this SHM was computed using inferred-germline VH1-2 alleles without knowledge of personalized genotype. Of 11831 VH1-2 allele calls for VRC01-class BCRs, 10593 (89.5%) were called as *02 with no ambiguity in scoring, 1007 (8.5%) were called as *04 with no ambiguity in scoring, 225 (1.9%) were called as *02 in cases where *02 and *04 were ranked as equally likely, 5 (0.043%) were called as *02 in cases where *02 and *05 were ranked as equally likely, and 1 (0.0085%) was called *02 in a case where *02, *04, and *05 were ranked as equally likely.

The original G00x-G002 pipeline was developed and designed to be adaptable for additional trials such as G00x-G003. This pipeline parses and validates input files from flow sorting and 10x Genomics sequencing experiments, converts raw BCL sequencing data into fully annotated paired BCR heavy and light chains associated with cell count data and other clinical trial metadata, calculates frequencies of VRC01-class B cells and other B cell subsets, and combines all analyses into a DataFrame (stored as a .CSV file). Within this pipeline, BCR V gene assignments and SHM were determined using Sequencing Analysis and Data library for Immunoinformatics Exploration (SADIE, https://github.com/jwillis0720/sadie), and paired BCR heavy and light chains and their characteristics were stored in the standardized Adaptive Immune Receptor Repertoire (AIRR) format (71). The entire G003 bioinformatics pipeline was packaged and will be made public upon manuscript publication (https://github.com/schieflab/G00x-G003). Details of individual stages of the pipeline are described in the following sections.

##### G003 flow data

Using the AWS client, flow data collected from the trial participants were deposited into folders, labeled with the sort and upload dates, in a private AWS S3 bucket, which offers a secure file storage service. The directory structure used was as follows:


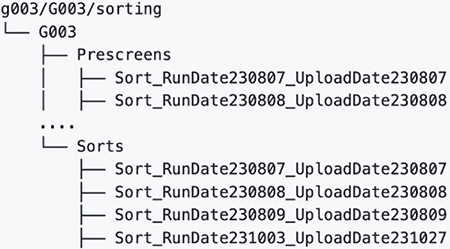


The flow data folders stored on the S3 bucket were synced onto an AWS FSx file system that was then mounted to an AWS EC2 instance (fig. S17B). Once on the EC2 instance, we used the command “g00x g003 validate” to verify the metadata associated with each folder. This verification involved examining the labeled data stats files generated from FlowJo. The Excel files containing the sorting information followed the naming structure below:

<run purpose>_<run date>_<sort id>_<ptid>_<visit id>_<sort probe>_<sample type>_<sort software>_<sort file type>_<tube id> _<pool id><pool subset id>.xlsx

Each Excel file was programmatically checked to ensure compliance with strict formatting rules.

##### G003 sequencing data

The sequencing data used the same file storage pipeline as the flow data (fig. S18B) and were uploaded as raw Illumina BCL files under the run identifier “run_id####”, where “#” represents a digit, with a sequencing manifest in a .CSV for each run. The data was validated for expected formats and organized in sequential folders (run0001, run0002, … run0007) corresponding to each sequencing run.


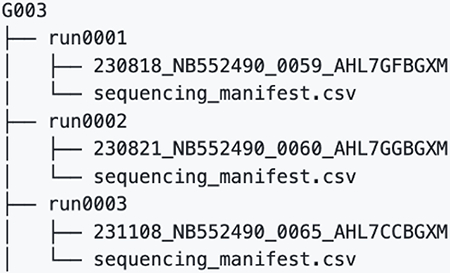


The sample_manifest.csv contained the following fields: ptid, timepoint, sorted_date, cells, hto, vdj_index, cso_index, pool_number, and run_id.

#### G003 demultiplex BCL

The G00x-G003 BCL demultiplexing step, with the command “g00x g003 pipeline demultiplex”, handled the demultiplexing of the BCL files using the same methods as for G00x-G002 except that for G003 we utilized CellRanger version 7.2.0 instead of version 6.1.2.

#### G003 VDJ and CSO

The command “g00x g003 pipeline vdj” was used to call on “cellranger vdj,” which generated a VDJ output for each sample or mixture of samples. This output was then demultiplexed using the CSO command and saved in the DataFrame as vdj.feather. Next, “g00x g003 pipeline cso” was invoked, which in turn called on “cellranger count” to count each hashtag (feature index — the unique oligo associated with each experimental sample, i.e. participant and timepoint) associated with a cellhash (the unique oligo associated with each cell). The location of this feature-index DataFrame was saved as cso.feather. The “cellranger count” command was chosen instead of the “cellranger multi” command to simplify downstream code and DataFrame complexity.

#### G003 AIRR

To complete the final demultiplexing step using CSO and VDJ libraries, we called the “g00x g003 pipeline airr” command. The initial processing of VDJ libraries was run through SADIE (www.github.com/jwillis0720/sadie.git) to generate an AIRR-compliant DataFrame. This intermediate AIRR-compliant DataFrame was then utilized to pair the IGH locus (heavy chain) sequences with the IGK or IGL locus sequences, if they only had one heavy chain-light chain pair. The airr command also collected feature index counts for each CSO and merged them back onto the paired VDJ to determine the corresponding sample for each cellhash. Feature indexes with fewer than 100 counts were discarded, and a 95% threshold was used to avoid doublets or multiplets.

The “g00x g003 pipeline airr” command then identified somatic mutations and recorded them in Kabat numbering format. As noted above, for the analysis of SHM in the VH1-2 gene, whereas in G001 (15) and G002 this SHM was computed using personalized VH1-2 genotypes from each participant, in G003 this SHM was computed using inferred germline VH1-2 alleles. In all cases the inferred germline allele was either *02 or *04. A BCR was classified as a VRC01-class BCR if it contained the IGHV1-2 allele *02 or *04 and had an LCDR3 length of 5, in which case the is_vrc01_class field was marked as True in the DataFrame. We identified key VRC01-class heavy chain (HC) residues using the same procedure as for G001 (15) and G002: we looked at both Kabat mutations and a list of all key VRC01-class HC residues. We counted the number of Kabat mutations also present in the list of key VRC01-class HC residues, then subtracted any Kabat mutations that deviated from a germline-encoded key HC residue, giving us the total count of key VRC01-class HC residues.

The final step from the “g00x g003 pipeline airr” command was to perform an initial clustering across all sequences. Sequences were grouped on the top V heavy call, top V light call, junction of amino acid heavy length, top c call, and the Boolean value of is_vrc01_class. Each resulting group was evaluated using the Levenshtein distance across HCDR3 and LCDR3 between each pair of sequences. An average linkage cluster distance of 3 was assigned to each sequence.

All the above information was recorded in the sequence DataFrame and saved to a final_df.feather file.

#### G003 flow report

The command “g00x g003 pipeline flow” created a flow DataFrame by parsing population sort files from both CFHR and AURUM. It matched gates to sort populations and saved this information as a flow.feather DataFrame. Each flow run is displayed as a separate row in the flow.feather DataFrame and includes fields to help navigate back to the original sort run. The list of all G003 gates is available in table S75.

#### G003 flow and AIRR merged

The data was joined with the flow data to get ptid, sorting probe, and visit by joining on pool number and sorted date to make a master Pandas DataFrame (https://github.com/pandas-dev/pandas) that contained the locations of all files. This DataFrame was stored as merged.feather to be used to create the downstream analysis report.

#### G003 analysis Report

The “g00x g003 analysis report” command took as input the final_df.feather file from the sequencing data and the flow.feather file to compute frequencies. The flow frequencies were recorded by taking as the numerator the events of a sort-specific population and taking as the denominator the events of a B-cell population. The resulting counts and frequencies were the core metrics for additional analysis and visualizations. The list of frequencies reported for G003 is given in table S76.

### G002 and G003 computing VRC01-class frequencies by combining frequencies from FACS and BCR sequencing

The frequency of VRC01-class IgG memory B cells was estimated by multiplying two frequencies: (1) the frequency of CD4bs-specific IgG memory B cells among all IgG memory B cells sorted by FACS; and (2) the frequency of VRC01-class BCRs among all CD4bs-specific IgG Memory B cells with sequenced BCRs. For samples sorted with eOD-GT8 probes, the frequency of eOD-GT8 CD4bs-specific (GT8^++^GT8-KO^−^) IgG B cells was used. For samples sorted with core-g28v2 probes, the frequency of core-g28v2 CD4bs-specific (core^++^core-KO^−^) IgG B cells was used. When the frequency of CD4bs-specific IgG memory B cells was zero, the estimate for the VRC01-class frequency was also set to zero. In the case where the frequency of CD4bs-specific IgG memory B cells was positive but no CD4bs-specific IgG memory B cells were successfully sequenced, the estimate for the VRC01-class frequency was set to zero for postvaccination samples.

#### Cross-calibration of B cell workflows from G001, G002, and G003

Retesting of 10 previously analyzed G001 samples using the G002 workflow produced significantly higher frequencies of eOD-GT8-specific (2.3-fold), eOD-GT8 CD4bs-specific (1.9-fold), and VRC01-class IgG B cells (1.7-fold), and similar degrees of V_H_ SHM (0.88-fold) and V_K/L_ SHM (0.89-fold) among VRC01-class IgG B cells (fig. S19 and table S40). Retesting a subset of those samples with the G003 workflow produced significantly higher frequencies of eOD-GT8-specific and eOD-GT8 CD4bs-specific IgG B cells than in either G001 or G002; however, due to technical difficulties we recovered VRC01-class B cells in only three samples, which we judged insufficient to capture methodological differences for the frequency of VRC01-class IgG B cells or the SHM in VRC01-class BCRs (fig. S19 and table S40). Given the observed methodological differences, we did not make quantitative inter-trial comparisons, except for comparisons between G002 and G001 for the frequency of VRC01-class IgG B cells and the degree of SHM in those cells. Those comparisons showed the smallest methodological differences and were of high interest. We developed a statistical approach to evaluate the significance of differences in VRC01-class IgG B cell frequencies and SHM between G001 and G002, using the retesting results to account for methodological differences quantitatively (see Inter-trial comparisons of outcomes involving trial-specific B cell workflows). For these comparisons, we pooled data from both G001 dose groups, because differences between G001 dose groups were modest and largely attributable to differences in VH1-2 genotype unrelated to dose (15, 17).

#### G002 and G003 VRC01-class response calls

A VRC01-class vaccine-induced response call was evaluated after one or two vaccinations with eOD-GT8 60mer. Post-vaccination responses were considered positive for a VRC01-class response if the frequency of VRC01-class B cells among IgG B cells at that timepoint was greater than at baseline. For participants with no sequences of CD4bs-specific memory B cells available at baseline, the baseline VRC01-class frequency used for the response call was estimated as the frequency of CD4bs-specific IgG memory B cells, which was a conservative approach. For participants with no sequences of CD4bs-specific memory B cells available at non-baseline timepoints, which occurred in a few cases in G003 due to technical failures in the Gel Bead in Emulsion process, the samples were dropped from response call analysis.

#### G002 and G003 BCR sequence hierarchical clustering

Clustering of post eOD-GT8 60mer BCR sequences for comparison between G001, G002 and G003 (fig. S29 and S30) was performed on VRC01-class IgG sequences from samples sorted as eOD-GT8 CD4bs-specific at weeks 4, 8, 10 and 16 (G001), weeks 4, 8, 16, and 24 (G002), or weeks 8, 10, 16, and 21 (G003) using the SADIE cluster module with average-linkage clustering and a distance cutoff of 3. Clustering was performed across BCRs from all participants from one trial together to facilitate identification of public clonotypes. Sequences within a cluster were required to share the same heavy and light V-gene calls and the same length CDR3s. The distance between two antibodies was the sum of Levenshtein distances between the HCDR3s and LCDR3s.

Clustering of BCRs from G002 prime-boost regimens (fig. S35) was performed on VRC01-class IgG sequences from samples sorted as core-g28v2 CD4bs-specific at weeks 8, 16, and 24. Individual sequences within a cluster were required to come from the same participant and to share the same heavy and light V gene calls and the same CDR3 lengths. The distance between two antibodies was the sum of Levenshtein distances between the HCDR3s and LCDR3s. Average-linkage clustering with a distance cutoff of 5 was used to account for the higher levels of SHM observed after boosting.

A third round of G002 BCR clustering was performed for all VRC01-class IgG sequences sorted as either eOD-GT8 CD4bs-specific or core-g28v2 CD4bs-specific across all timepoints to identify expanded clonal lineages for additional analysis. Individual sequences within a cluster were required to come from the same participant and to share the same heavy and light V gene calls and the same CDR3 lengths. The distance between two antibodies was the sum of Levenshtein distances between the HCDR3s and LCDR3s. Average-linkage clustering with a distance cutoff of 5 was used to account for the higher levels of SHM observed after boosting.

In all three cases of clustering above, identifying appropriate clustering parameters was an iterative process. We manually inspected the results of the clustering algorithm to identify incorrectly clustered sequences at the extremes of HCDR3 length and adjusted the clustering parameters until no such misclustered sequences were found.

#### Lineage analysis

The three largest clusters (figs. S37 and S38) were extracted from the SADIE data frame, and the corresponding HCs were formatted for use in the Immcantation software suite. Maximum likelihood-based lineage trees were built for each cluster with Dowser (72) using the IgPhyML method in the getTrees function with separate partitions for the CDRs and FRs (73). Divergence was calculated using Dowser as the sum of branch lengths leading from the root of the tree to each tip. Divergence versus timepoint graphs were plotted in Prism and single linear regression analysis was performed. To detect B cell evolution over time, a phylogenetic date randomization test was performed using the correlationTest function in Dowser (74, 75). Trees were plotted and annotated using iTOL (https://itol.embl.de/). IgGs were synthesized and produced as described below for a subset of BCRs from each lineage tree and assessed for binding to core-g28v2 using SPR.

#### G002 counting key VRC01-class light chain mutations

Key VRC01-class light chain mutations for VRC01-class antibodies with kappa light chains were defined as deletions or glycine mutations in the LCDR1 (Kabat residues 26 to 32 inclusive) that could potentially accommodate the N276 glycan or V gene-specific mutations determined from structural analysis of multiple VRC01-class bnAbs with HIV Env trimer or gp120. All alleles of a given V gene were included in the analysis. The list of key VRC01-class light chain mutations is as follows:

VK1-33, VK1-5, VK3-15, or VK3-20: deletion or mutation to glycine at any position within 26 to 32.

VK1-33: Y32D, Y32S, N34H, N34Q, Y49H, D50H, D50G, S67F, G68H, D70S, D70T, D70P, F73L

VK1-5: Y49H, Y49F, D50H, D50K, S67F, S67H, S67Y, S67W, G68H, G68F, G68Y, G68W

VK3-15: N32D, L33V, S67F, G68H

VK3-20: S30Y, Y32S, G66R, G66K, S67W, S67F, S67Y, F71Y

#### G002 and G003 bioinformatic BCR sequence analyses other than VRC01-calls or clustering

Statistical quantile analyses throughout the manuscript were carried out using Pandas (https://github.com/pandas-dev/pandas). Confidence intervals for response rates were computed using the Wilson score method (76). Analyses of VRC01-class BCR features were carried out by custom python functions available as a package in the data repository for G002 (https://github.com/SchiefLab/G00x-G002). When referring to control distributions from OAS (77, 78), we restricted to human, non-vaccinated, no disease state data from OAS.

#### G002 and G003 SPR

Kinetics and affinity of antibody–antigen interactions were measured on a Carterra LSA using the methods previously described (26). A typical SPR run tested six different analyte concentrations using a dilution factor of four. The top concentration for each analyte varied from run to run and between analytes. Core-g28v2 was run with top concentrations between 15.4 μM and 59.7 μM. Core-N276 was run with top concentrations between 33.5 μM and 56 μM. Trimers were run with top concentrations between between 11.7 μM and 30.3 μM. For trimer analytes, low density IgG capture was used to ensure that *K*_D_s approximated monovalent interactions to the greatest degree possible. For interactions measured more than once, the measurement with the lowest chi-squared fit value was chosen as the representative measurement. If no measurement resulted in a kinetic-fit *K*_D_ from the Carterra Kinetics software analysis, the *K*_D_ value was set to ≥100 μM for monomeric analytes or ≥50 μM for trimeric analytes. If the Carterra Kinetics software analysis reported a *K*_D_ value of >100 μM for a monomeric analyte or >50 μM for a trimeric analyte, the *K*_D_ was reset to ≥100 μM or ≥50 μM, respectively.

##### G002 glycan occupancy analysis

Glycan occupancy analysis of core-N276 was performed as previously described (79).

##### G002 negative stain EM analysis

Negative stain EM analysis of N276-lacking trimers was performed as previously described (80).

##### G002 pseudovirus production and mAb neutralization assays

Pseudovirus neutralization assays for mAbs were performed as previously described (26).

#### G002 serum neutralization assays

Neutralizing activity was measured as a function of an ability to reduce the infectivity of HIV Env-pseudotyped viruses in TZM-bl cells (81). A pre-titrated dose of virus was incubated with serial 5-fold dilutions of monoclonal antibodies or heat-inactivated (56°C, 30 minutes) serum samples in duplicate for 1 hr before adding TZM-bl cells. Neutralization was assessed after a 48 to 52 hour incubation period. Neutralization titers are the serum dilution or mAb concentration at which relative luminescence units (RLU) were reduced by 50% (ID50/IC50) or 80% (ID80/IC80) compared to virus control wells after subtraction of background RLUs. Assay stocks of molecularly cloned Env-pseudotyped viruses were prepared by transfection in 293T/17 cells (American Type Culture Collection) and titrated in TZM-bl cells (81). This assay has been formally optimized and validated (82) and was performed in compliance with Good Clinical Laboratory Practices, including participation in a formal proficiency testing program (83).

#### G002 structure determination using cryo-electron microscopy

The G002-293-0536 Fab complex was prepared by incubating 0.2 mg 001428_T278M_L14 SOSIP, 0.4 mg G002-293-0536 Fab and 0.3 mg RM20A3 Fab (an NHP antibody that binds the base of SOSIP and increases particle tumbling in EM) overnight at room temperature, followed by purification in Tris-buffered saline over a HiLoad 16/600 Superdex 200 pg (Cytiva) gel filtration column and concentrating to 8.2 mg/mL using a MilliporeSigma Amicon 100 kDa MWCO centrifugal filter. For G002-480-0546, 0.1 mg of V703-0537_T278M_L14 SOSIP was incubated with 0.1 mg G002-480-0546 Fab and 0.1 mg BG18 Fab (non-competing V3-glycan epitope) for 2 hours and concentrated to 6.4 mg/mL without the size-exclusion chromatography step. Cryo grids were prepared using a Vitrobot Mark IV (Thermo Fisher). The temperature was set to 4 °C and humidity was maintained at 100% during the freezing process. The blotting force was set to 1 and wait time was set to 10 s. Blotting time was varied from 3 to 6 s. Detergent lauryl maltose neopentyl glycol (LMNG; Anatrace) at a final concentration of 0.005 mM was used for freezing. UltrAufoil R 1.2/1.3 (gold, 300-mesh; Quantifoil Micro Tools GmbH) grids were glow discharged before sample application. 0.5 μL of detergent was mixed with 3.5 μL of samples and 3 μL of the mixture was immediately loaded onto the grid. Following blotting, the grids were plunge-frozen into liquid nitrogen-cooled liquid ethane.

Cryo grids were loaded into a Thermo Fisher Scientific Glacios electron microscope operating at 200 kV. Exposure magnification was set to 190,000x with a pixel size at the specimen plane of 0.718 Å. EPU software (Thermo Fisher) was used for automated data collection. Micrograph movie frames were motion and CTF corrected using cryoSPARC Live (84). The remaining data processing was performed in cryoSPARC. Particle picking was performed using blob picker initially followed by template picker. During extraction particles were downscaled to 1.0052 Å/pixel (G002-293-0536 dataset) or 1.0339 Å/pixel (G002-480-0546) to reduce box size and increase speed of downstream jobs. Multiple rounds of 2D classification and 3D ab-initio reconstruction were performed prior to 3D non-uniform refinement with global CTF refinement. Final data collection and processing stats are summarized in fig. S58 and table S73. Model building was performed by docking homology models of trimer (generated by AlphaFold 3 (85)) and Fab Fv (generated by AbodyBuilder2 (86)) in UCSF ChimeraX (87), manually building and refining in Coot 0.9.8 (88) and real space refinement using Phenix (89). Final models were validated using MolProbity and EMRinger in the Phenix suite, and statistics are summarized in table S73. All maps and models have been deposited to the Electron Microscopy Data Bank and Protein Data Bank, respectively with accession codes summarized in table S73.

#### G002 structural Analysis

Visualization, structural analysis, and figure generation was performed using UCSF Chimera and ChimeraX (87, 90). In the reference structure of VRC01 bound to BG505 F14 SOSIP (PDB: 6V8X) (91) we corrected the D368 rotamer in gp120 to the most probable Dunbrack rotamer (92) using UCSF Chimera (90). For the angle of approach analysis, G002 structures were aligned to 6V8X in UCSF ChimeraX based on gp120 and the C*α* RMSD values between the G002 heavy chains and the VRC01 heavy chain (residues 3 to 92) were calculated. The buried surface area (BSA) of the Env trimers in complex with the G002 antibodies and VRC01 was determined using PISA (93). Contact residues identified during the BSA analysis were used to generate epitope footprints. For the Phe43 interaction, the structure of B41 SOSIP in complex with soluble human CD4 (PDB: 5VN3) (94) was aligned to the 6V8X reference in UCSF ChimeraX based on gp120.

#### G002 and G003 statistical analyses

##### Safety and reactogenicity analyses

Safety analyses used the safety analysis set defined as ever receiving at least one dose of investigational product. This safety set is the same as the intent-to-treat analysis set as all participants enrolled received at least one dose of investigational product. Basic descriptive statistics were used to summarize categorical and continuous variables. Ninety-five percent confidence intervals for proportions were constructed using the exact Clopper-Pearson methods (95) (tables S14, S15, S19, S21, and S22). Analyses were conducted via SAS version 9.4 (SAS Institute, Cary, North Carolina).

##### BAMA assay concordance between G001 and G003

Lin’s concordance correlation coefficient (CCC) (96) was computed to measure the concordance of log10 binding responses after truncating values at 100, (AUTC for individual antigens and ΔAUTC for differential binding), for several outcomes measured using BAMA by the Duke and KAVI labs (fig. S9). The CCC was computed using samples from 7 G001 vaccine recipients at study weeks 0 (baseline), 2, 8, 10, and 16 (baseline included) or at the 4 post-vaccination study weeks (excluding baseline). The CCC is the product of a bias correction factor (a measure of accuracy) and Pearson’s correlation coefficient (a measure of precision). Confidence intervals (CIs) for CCC are computed based on asymptotic normality of a Z-transformation of the estimator.

##### General statistical testing

For all tests, a minimum of three participants per group was required to conduct the test. The Wilcoxon rank-sum test was used for independent two-sample comparisons of the magnitude of responses across trials (Figs. 2E and 3, A to B, figs. S25 and S30, and table S54) or pseudo-groups (Figs. 4, A to D and F to H; 5, A, B, E, and F; 7, A, C, D, E, G, and H; fig. S40, tables S57 to S61, S62, S67, and S68), while comparisons of response rates between independent groups were conducted using a two-sided Barnard test (Figs. 4E and 7, B and F, and tables S57, S67, and S68). For paired comparisons, the Wilcoxon signed-rank test was applied to assess changes in the magnitude of responses across time (Figs. 2, A to C and E to F; 3, A, B, and E; 5, A, B, E, and F; tables S46, S47, S55, S56, S58, S61, and S70), and McNemar’s test was used to evaluate changes in response rates over time (tables S33, S57, and S69). For calculating the 90^th^ percentiles per sample in Fig. 3E and Fig. 5 E and F, the nearest method (97) implemented in NumPy (https://numpy.org/doc/stable/reference/generated/numpy.quantile.html) was used; similar results were obtained using the midpoint and median-unbiased methods. Comparisons between G001 and G002 in Fig. 3E were not conducted owing to the large difference in the number of sequences per participant in the two trials.

To evaluate the effect of regimen on the neutralizing IC_50_ (Table 1 and tables S65 and S66), we employed a marginal mean model fit with generalized estimating equations (GEE) (98). The model estimated the geometric mean ratio IC_50_ between regimens or time points using all antibodies and N276-lacking pseudoviruses, accounting for within-individual correlation among multiple IC_50_ values. The model specified an independence correlation structure and a gaussian error distribution and was fit using the gee package in R (version 4.13-29). Robust standard errors, computed using a sandwich estimator, were used to calculate a 95% confidence interval and two-sided P-values. A similar approach was used to estimate the geometric mean ratio of *K*_D_ (Fig. 6, figs. S47 and S48, and tables S63, S64, and S68) for comparison across regimens and time points, and to estimate the geometric mean ratio of *K*_D_ (Fig. 8A and table S78) across time points for different antigens, while accounting for within-individual correlation of *K*_D_ across multiple antibodies.

##### Inter-trial comparisons of outcomes involving trial-specific B cell workflows

For comparisons of outcomes from trial-specific B-cell sorting and sequencing (fig. S19 and table S40) conducted on G001 samples, a paired t-test was used. For comparisons between trial results where outcomes were measured using trial-specific methodologies (Figs. 2E, 3A and 3B, and tables S48 and S53), a simulation-based approach was employed.

For analyses, data were either evaluated on the original scale or following log-transformation, depending on how the data are shown in the figures. When a figure was displayed using a log-transformed scale, the data were analyzed on the log-transformed scale. Differences between groups were represented as the mean difference for analyses on the original scale or as the geometric mean ratio for analyses on the log-transformed scale. Additionally, log-transformed analyses were done conditionally on the set of non-zero values.

At each time point for a given outcome, the mean and standard deviation were calculated for both trials being compared. These parameters were used to define normal distributions for the trial-specific outcomes. The method difference was represented by a mean-shifted and standard-deviation-scaled t-distribution, derived from the paired data of the trial-specific methodology assessment. A parametric bootstrap with 10,000 simulations was performed to estimate the 95% confidence interval for the methodology-adjusted difference (net difference) in group means. For each simulation, the net difference was computed as the mean of the group 2 data minus the mean of the group 1 data, adjusted by subtracting the methodology-induced mean difference between groups. The 95% confidence interval for the net difference was determined as the 2.5th and 97.5th percentiles of the simulated net differences. The P-value was calculated as twice the smaller of the proportions of simulated net differences either above or below zero, with an additional 0.5 included in the numerator, divided by the total number of simulations plus one.

##### Multiplicity adjustment

A multiplicity adjustment was applied to control the false discovery rate (FDR) at 0.20 across the set of P-values within each figure panel, except where otherwise noted in the table caption. A result was considered statistically significant if both the unadjusted P-value was less than 0.05 and the FDR-adjusted P-value (or Q-value) was less than 0.2 (45, 46).

##### Analysis of the dependence of neutralization IC_50_ on binding kinetics parameters

Segmented linear regression was used to analyze the dependence of neutralization IC_50_ on SPR-measured binding parameters *K*_D_, *k*_off_, and *k*_on_ in figs. S53, S54, and S55, respectively. The IC_50_ log_10_ transformation was individually plotted against the log_10_ transformations of *K*_D_, *k*_off_, and *k*_on_. Segmented linear regression was performed in Prism, with slope2 constrained to 0 while allowing the remaining variables to be fitted. X0, the intercept of the two lines, was the *K*_D_, *k*_off_, or *k*_on_ threshold needed to detect neutralization activity. Segmented linear regression did not result in converged fits for the IC_50_ versus *k*_on_ plots.

#### Figure generation

Most figures (Figs. 2 to 7, and figs. S19 to S35, S40 to S43, S46 to S48, and S52) were generated using Python with either Matplotlib (99) or a custom port of the Seaborn package that incorporates Wilson confidence intervals into the statistical analysis [ (100); https://github.com/tmsincomb/seaborn-fork]. Figure 8A and figs. 53 to 55 were made using Prism. Figures S8 to S10 were produced using the ggplot package in R. Python code for figure generation can be found in the accompanying data analysis repository (https://github.com/SchiefLab/G002-and-G003-figures). Figure 1A and 1B and figs. S16 and S17 were produced using BioRender (BioRender.com), and the publishing license can be found in the data repository.

## Supplementary Material

Data S1

Data S2

Data S3

Data S4

Data S5

Data S7

Data S8

Data S9

Data S6

Supp

Data S10

## Figures and Tables

**Table 1.** Neutralization IC_50_s for the indicated VRC01-class mAbs against the indicated pseudoviruses The data are representative of two independent assays in each case. All mAbs from IAVI G002 in this figure correspond to B cells sorted with core-g28v2 probes. Source data can be found in Data S10.

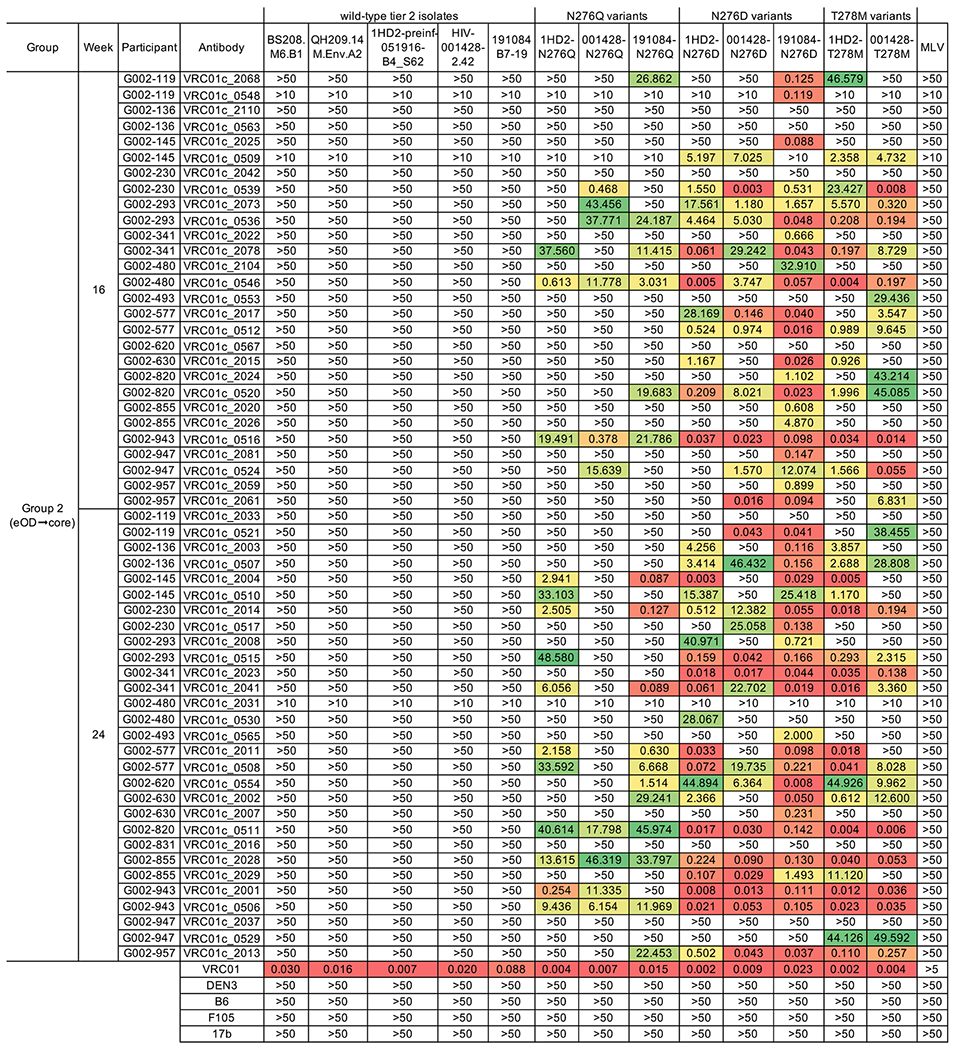

## Data Availability

All BCR sequences and FACS analysis files produced in these studies are available in the public data repositories https://github.com/SchiefLab/G002-and-G003 permanently archived at (this will be a Zenodo link). All cryo-EM maps are available in the public repository Electron Microscopy Data Bank (EMDB) under accession codes EMD-48575 and EMD-48591, and cryo-EM atomic models in the public repository Protein Data Bank (PDB) under accession codes 9MSD and 9MSY. All other data are available in the main text or supplementary materials.

## References

[1] Pegu A , A Meta-analysis of Passive Immunization Studies Shows that Serum-Neutralizing Antibody Titer Associates with Protection against SHIV Challenge. Cell Host Microbe 26, 336–346 e333 (2019). 31513771 10.1016/j.chom.2019.08.014 PMC6755677

[2] Corey L , Two Randomized Trials of Neutralizing Antibodies to Prevent HIV-1 Acquisition. N Engl J Med 384, 1003–1014 (2021). 33730454 10.1056/NEJMoa2031738 PMC8189692

[3] Gilbert PB , Neutralization titer biomarker for antibody-mediated prevention of HIV-1 acquisition. Nature Medicine 28, 1924–1932 (2022). 10.1038/s41591-022-01953-6 PMC9499869 35995954

[4] Pegu A , Antibodies targeting the fusion peptide on the HIV envelope provide protection to rhesus macaques against mucosal SHIV challenge. Sci Transl Med 16, eadh9039 (2024). 38232141 10.1126/scitranslmed.adh9039 PMC13310453

[5] Jardine J , Rational HIV immunogen design to target specific germline B cell receptors. Science 340, 711–716 (2013). 23539181 10.1126/science.1234150 PMC3689846

[6] McGuire AT , Engineering HIV envelope protein to activate germline B cell receptors of broadly neutralizing anti-CD4 binding site antibodies. J Exp Med 210, 655–663 (2013). 23530120 10.1084/jem.20122824 PMC3620356

[7] Escolano A , Sequential Immunization Elicits Broadly Neutralizing Anti-HIV-1 Antibodies in Ig Knockin Mice. Cell 166, 1445–1458 e1412 (2016). 27610569 10.1016/j.cell.2016.07.030 PMC5019122

[8] Jardine JG , HIV-1 broadly neutralizing antibody precursor B cells revealed by germline-targeting immunogen. Science 351, 1458–1463 (2016). 27013733 10.1126/science.aad9195 PMC4872700

[9] Steichen JM , HIV Vaccine Design to Target Germline Precursors of Glycan-Dependent Broadly Neutralizing Antibodies. Immunity 45, 483–496 (2016). 27617678 10.1016/j.immuni.2016.08.016 PMC5040827

[10] Tian M , Induction of HIV Neutralizing Antibody Lineages in Mice with Diverse Precursor Repertoires. Cell 166, 1471–1484 e1418 (2016). 27610571 10.1016/j.cell.2016.07.029 PMC5103708

[11] Medina-Ramirez M , Design and crystal structure of a native-like HIV-1 envelope trimer that engages multiple broadly neutralizing antibody precursors in vivo. J Exp Med 214, 2573–2590 (2017). 28847869 10.1084/jem.20161160 PMC5584115

[12] Parks KR , Overcoming Steric Restrictions of VRC01 HIV-1 Neutralizing Antibodies through Immunization. Cell Rep 29, 3060–3072 e3067 (2019). 31801073 10.1016/j.celrep.2019.10.071 PMC6936959

[13] Steichen JM , A generalized HIV vaccine design strategy for priming of broadly neutralizing antibody responses. Science 366, (2019). 10.1126/science.aax4380 PMC7092357 31672916

[14] Chen X , Vaccination induces maturation in a mouse model of diverse unmutated VRC01-class precursors to HIV-neutralizing antibodies with >50% breadth. Immunity 54, 324–339 e328 (2021). 33453152 10.1016/j.immuni.2020.12.014 PMC8020832

[15] Leggat DJ , Vaccination induces HIV broadly neutralizing antibody precursors in humans. Science 378, eadd6502 (2022). 36454825 10.1126/science.add6502 PMC11103259

[16] Cohen KW , A first-in-human germline-targeting HIV nanoparticle vaccine induced broad and publicly targeted helper T cell responses. Sci Transl Med 15, eadf3309 (2023). 37224227 10.1126/scitranslmed.adf3309 PMC11036875

[17] deCamp AC , Human immunoglobulin gene allelic variation impacts germline-targeting vaccine priming. NPJ Vaccines 9, 58 (2024). 38467663 10.1038/s41541-024-00811-5 PMC11384754

[18] Sprumont A, Rodrigues A, McGowan SJ, Bannard C, Bannard O, Germinal centers output clonally diverse plasma cell populations expressing high- and low-affinity antibodies. Cell 186, 5486–5499 e5413 (2023). 37951212 10.1016/j.cell.2023.10.022 PMC7617393

[19] ElTanbouly MA , Role of affinity in plasma cell development in the germinal center light zone. J Exp Med 221, (2024). 10.1084/jem.20231838 PMC10631489 37938344

[20] Sutton HJ , Lack of affinity signature for germinal center cells that have initiated plasma cell differentiation. Immunity 57, 245–255 e245 (2024). 38228150 10.1016/j.immuni.2023.12.010 PMC10922795

[21] McNamara HA , Antibody Feedback Limits the Expansion of B Cell Responses to Malaria Vaccination but Drives Diversification of the Humoral Response. Cell Host Microbe 28, 572–585 e577 (2020). 32697938 10.1016/j.chom.2020.07.001

[22] Tas JMJ , Antibodies from primary humoral responses modulate the recruitment of naive B cells during secondary responses. Immunity 55, 1856–1871 e1856 (2022). 35987201 10.1016/j.immuni.2022.07.020 PMC9350677

[23] Schaefer-Babajew D , Antibody feedback regulates immune memory after SARS-CoV-2 mRNA vaccination. Nature 613, 735–742 (2023). 36473496 10.1038/s41586-022-05609-w PMC9876794

[24] Mesin L , Restricted Clonality and Limited Germinal Center Reentry Characterize Memory B Cell Reactivation by Boosting. Cell 180, 92–106 e111 (2020). 31866068 10.1016/j.cell.2019.11.032 PMC6958527

[25] Viant C , Antibody Affinity Shapes the Choice between Memory and Germinal Center B Cell Fates. Cell 183, 1298–1311 e1211 (2020). 33125897 10.1016/j.cell.2020.09.063 PMC7722471

[26] C. A. Cottrell , Heterologous prime-boost vaccination drives early maturation of HIV broadly neutralizing antibody precursors in humanized mice. Sci Transl Med 16, eadn0223 (2024). 38753806 10.1126/scitranslmed.adn0223 PMC11233128

[27] Wang X , mRNA-LNP prime boost evolves precursors toward VRC01-like broadly neutralizing antibodies in preclinical humanized mouse models. Sci Immunol 9, eadn0622 (2024). 38753808 10.1126/sciimmunol.adn0622 PMC11488661

[28] West AP Jr., Diskin R, Nussenzweig MC, Bjorkman PJ, Structural basis for germ-line gene usage of a potent class of antibodies targeting the CD4-binding site of HIV-1 gp120. Proceedings of the National Academy of Sciences of the United States of America 109, E2083–2090 (2012). 22745174 10.1073/pnas.1208984109 PMC3409792

[29] Zhou T , Multidonor analysis reveals structural elements, genetic determinants, and maturation pathway for HIV-1 neutralization by VRC01-class antibodies. Immunity 39, 245–258 (2013). 23911655 10.1016/j.immuni.2013.04.012 PMC3985390

[30] Baden LR , Efficacy and Safety of the mRNA-1273 SARS-CoV-2 Vaccine. N Engl J Med 384, 403–416 (2021). 33378609 10.1056/NEJMoa2035389 PMC7787219

[31] Wilson E , Efficacy and Safety of an mRNA-Based RSV PreF Vaccine in Older Adults. N Engl J Med 389, 2233–2244 (2023). 38091530 10.1056/NEJMoa2307079

[32] https://www.unaids.org/en/resources/fact-sheet. (2024).

[33] Campbell MC, Tishkoff SA, African genetic diversity: implications for human demographic history, modern human origins, and complex disease mapping. Annu Rev Genomics Hum Genet 9, 403–433 (2008). 18593304 10.1146/annurev.genom.9.081307.164258 PMC2953791

[34] Watson CT , Complete haplotype sequence of the human immunoglobulin heavy-chain variable, diversity, and joining genes and characterization of allelic and copy-number variation. Am J Hum Genet 92, 530–546 (2013). 23541343 10.1016/j.ajhg.2013.03.004 PMC3617388

[35] Scheepers C , Ability to develop broadly neutralizing HIV-1 antibodies is not restricted by the germline Ig gene repertoire. Journal of immunology 194, 4371–4378 (2015). 10.4049/jimmunol.1500118 PMC4513073 25825450

[36] Rodriguez OL , Genetic variation in the immunoglobulin heavy chain locus shapes the human antibody repertoire. Nat Commun 14, 4419 (2023). 37479682 10.1038/s41467-023-40070-x PMC10362067

[37] Petrova VN , Incomplete genetic reconstitution of B cell pools contributes to prolonged immunosuppression after measles. Sci Immunol 4, (2019). 10.1126/sciimmunol.aay6125 31672862

[38] Stelekati E, Wherry EJ, Chronic bystander infections and immunity to unrelated antigens. Cell host & microbe 12, 458–469 (2012). 23084915 10.1016/j.chom.2012.10.001 PMC3617576

[39] Borkow G , Chronic immune activation associated with intestinal helminth infections results in impaired signal transduction and anergy. The Journal of clinical investigation 106, 1053–1060 (2000). 11032865 10.1172/JCI10182 PMC314342

[40] Clerici M , Immune activation in africa is environmentally-driven and is associated with upregulation of CCR5. Italian-Ugandan AIDS Project. Aids 14, 2083–2092 (2000). 11061648 10.1097/00002030-200009290-00003

[41] Matassoli F , High frequency of HIV precursor-target-specific B cells in sub-Saharan populations. Cell Rep 42, 113450 (2023). 38019653 10.1016/j.celrep.2023.113450 PMC10886445

[42] Stewart-Jones GBE , Domain-based mRNA vaccines encoding spike protein N-terminal and receptor binding domains confer protection against SARS-CoV-2. Sci Transl Med 15, eadf4100 (2023). 37703353 10.1126/scitranslmed.adf4100

[43] Chalkias S , Efficacy, immunogenicity, and safety of a next-generation mRNA-1283 COVID-19 vaccine compared with the mRNA-1273 vaccine: results from NextCOVE, a phase 3, randomised, observer-blind, active-controlled trial. Lancet Infectious Diseases, (in-press). 10.1016/S1473-3099(25)00236-1 40639387

[44] Lang DM, Chronic Urticaria. N Engl J Med 387, 824–831 (2022). 36053507 10.1056/NEJMra2120166

[45] Juraska M , Prevention efficacy of the broadly neutralizing antibody VRC01 depends on HIV-1 envelope sequence features. Proceedings of the National Academy of Sciences of the United States of America 121, e2308942121 (2024). 38241441 10.1073/pnas.2308942121 PMC10823214

[46] Benjamini Y, Hochberg Y, Controlling the False Discovery Rate: A Practical and Powerful Approach to Multiple Testing. Journal of the Royal Statistical Society: Series B (Methodological) 57, 289–300 (2018).

[47] Jardine JG , Minimally Mutated HIV-1 Broadly Neutralizing Antibodies to Guide Reductionist Vaccine Design. PLoS pathogens 12, e1005815 (2016). 27560183 10.1371/journal.ppat.1005815 PMC4999182

[48] Diskin R , Restricting HIV-1 pathways for escape using rationally designed anti-HIV-1 antibodies. J Exp Med 210, 1235–1249 (2013). 23712429 10.1084/jem.20130221 PMC3674693

[49] El Sahly HM , Efficacy of the mRNA-1273 SARS-CoV-2 Vaccine at Completion of Blinded Phase. N Engl J Med 385, 1774–1785 (2021). 34551225 10.1056/NEJMoa2113017 PMC8482810

[50] Anvari S , Urticaria and/or angioedema secondary to mRNA COVID-19 vaccines: Updates from a United States case registry. Allergy 78, 283–286 (2023). 35842747 10.1111/all.15447 PMC9349391

[51] Duperrex O, Tommasini F, Muller YD, Incidence of Chronic Spontaneous Urticaria Following Receipt of the COVID-19 Vaccine Booster in Switzerland. JAMA Netw Open 6, e2254298 (2023). 36723944 10.1001/jamanetworkopen.2022.54298 PMC9892951

[52] Sharma VK , Use of Transient Transfection for cGMP Manufacturing of eOD-GT8 60mer, a Self-Assembling Nanoparticle Germline-Targeting HIV-1 Vaccine Candidate. bioRxiv, 2022.2009.2030.510310 (2022). 10.3390/pharmaceutics16060742 PMC11206926 38931864

[53] Behrens AJ , Integrity of Glycosylation Processing of a Glycan-Depleted Trimeric HIV-1 Immunogen Targeting Key B-Cell Lineages. J Proteome Res 17, 987–999 (2018). 29420040 10.1021/acs.jproteome.7b00639 PMC5846105

[54] Parks R , mRNA vaccination with HIV envelope trimer elicits neutralizing antibodies in humans. Science, (submitted).

[55] Willis JR , Human immunoglobulin repertoire analysis guides design of vaccine priming immunogens targeting HIV V2-apex broadly neutralizing antibody precursors. Immunity 55, 2149–2167 e2149 (2022). 36179689 10.1016/j.immuni.2022.09.001 PMC9671094

[56] Melzi E , Membrane-bound mRNA immunogens lower the threshold to activate HIV Env V2 apex-directed broadly neutralizing B cell precursors in humanized mice. Immunity 55, 2168–2186 e2166 (2022). 36179690 10.1016/j.immuni.2022.09.003 PMC9671093

[57] Xie Z , mRNA-LNP HIV-1 trimer boosters elicit precursors to broad neutralizing antibodies. Science 384, eadk0582 (2024). 38753770 10.1126/science.adk0582 PMC11488660

[58] Sok D , Priming HIV-1 broadly neutralizing antibody precursors in human Ig loci transgenic mice. Science 353, 1557–1560 (2016). 27608668 10.1126/science.aah3945 PMC5404394

[59] Abbott RK , Precursor Frequency and Affinity Determine B Cell Competitive Fitness in Germinal Centers, Tested with Germline-Targeting HIV Vaccine Immunogens. Immunity 48, 133–146 e136 (2018). 29287996 10.1016/j.immuni.2017.11.023 PMC5773359

[60] Havenar-Daughton C , The human naive B cell repertoire contains distinct subclasses for a germline-targeting HIV-1 vaccine immunogen. Sci Transl Med 10, (2018). 10.1126/scitranslmed.aat0381 PMC6145074 29973404

[61] Huang D , B cells expressing authentic naive human VRC01-class BCRs can be recruited to germinal centers and affinity mature in multiple independent mouse models. Proceedings of the National Academy of Sciences of the United States of America 117, 22920–22931 (2020). 32873644 10.1073/pnas.2004489117 PMC7502816

[62] Wang X , Multiplexed CRISPR/CAS9-mediated engineering of pre-clinical mouse models bearing native human B cell receptors. EMBO J 40, e105926 (2021). 33258500 10.15252/embj.2020105926 PMC7809789

[63] Cottrell CA , Priming antibody responses to the fusion peptide in rhesus macaques. NPJ Vaccines 9, 126 (2024). 38997302 10.1038/s41541-024-00918-9 PMC11245479

[64] Tomaras GD , Initial B-cell responses to transmitted human immunodeficiency virus type 1: virion-binding immunoglobulin M (IgM) and IgG antibodies followed by plasma anti-gp41 antibodies with ineffective control of initial viremia. Journal of virology 82, 12449–12463 (2008). 18842730 10.1128/JVI.01708-08 PMC2593361

[65] Yates NL , Vaccine-induced Env V1-V2 IgG3 correlates with lower HIV-1 infection risk and declines soon after vaccination. Sci Transl Med 6, 228ra239 (2014). 10.1126/scitranslmed.3007730 PMC4116665 24648342

[66] Yates NL , HIV-1 Envelope Glycoproteins from Diverse Clades Differentiate Antibody Responses and Durability among Vaccinees. Journal of virology 92, (2018). 10.1128/JVI.01843-17 PMC5874409 29386288

[67] Schiffner T , Diverse competitor B cell responses to a germline-targeting priming immunogen in human Ig loci transgenic mice. bioRxiv, 2024.2001.2022.575410 (2024).

[68] Vazquez Bernat N , High-Quality Library Preparation for NGS-Based Immunoglobulin Germline Gene Inference and Repertoire Expression Analysis. Front Immunol 10, 660 (2019). 31024532 10.3389/fimmu.2019.00660 PMC6459949

[69] Corcoran MM , Production of individualized V gene databases reveals high levels of immunoglobulin genetic diversity. Nat Commun 7, 13642 (2016). 27995928 10.1038/ncomms13642 PMC5187446

[70] Narang S, Kaduk M, Chernyshev M, Karlsson Hedestam GB, Corcoran MM, Adaptive immune receptor genotyping using the corecount program. Front Immunol 14, 1125884 (2023). 37114042 10.3389/fimmu.2023.1125884 PMC10126697

[71] Breden F , Reproducibility and Reuse of Adaptive Immune Receptor Repertoire Data. Front Immunol 8, 1418 (2017). 29163494 10.3389/fimmu.2017.01418 PMC5671925

[72] Hoehn KB, Pybus OG, Kleinstein SH, Phylogenetic analysis of migration, differentiation, and class switching in B cells. PLoS Comput Biol 18, e1009885 (2022). 35468128 10.1371/journal.pcbi.1009885 PMC9037912

[73] Hoehn KB , Repertoire-wide phylogenetic models of B cell molecular evolution reveal evolutionary signatures of aging and vaccination. Proceedings of the National Academy of Sciences of the United States of America 116, 22664–22672 (2019). 31636219 10.1073/pnas.1906020116 PMC6842591

[74] Jensen CG, Sumner JA, Kleinstein SH, Hoehn KB, Inferring B cell phylogenies from paired heavy and light chain BCR sequences with Dowser. bioRxiv, (2023). 10.4049/jimmunol.2300851 PMC11073909 38557795

[75] Hoehn KB , Human B cell lineages associated with germinal centers following influenza vaccination are measurably evolving. eLife 10, (2021). 10.7554/eLife.70873 PMC8741214 34787567

[76] Agresti A, Coull BA, Approximate is better than “exact” for interval estimation of binomial proportions. Am Stat 52, 119–126 (1998).

[77] Kovaltsuk A , Observed Antibody Space: A Resource for Data Mining Next-Generation Sequencing of Antibody Repertoires. Journal of immunology 201, 2502–2509 (2018). 10.4049/jimmunol.1800708 30217829

[78] Olsen TH, Boyles F, Deane CM, Observed Antibody Space: A diverse database of cleaned, annotated, and translated unpaired and paired antibody sequences. Protein Sci 31, 141–146 (2022). 34655133 10.1002/pro.4205 PMC8740823

[79] Baboo S , DeGlyPHER: An Ultrasensitive Method for the Analysis of Viral Spike N-Glycoforms. Anal Chem 93, 13651–13657 (2021). 34597027 10.1021/acs.analchem.1c03059 PMC8848675

[80] Ozorowski G , Effects of Adjuvants on HIV-1 Envelope Glycoprotein SOSIP Trimers In Vitro. Journal of virology 92, (2018). 10.1128/JVI.00381-18 PMC6002727 29669838

[81] Montefiori DC, Measuring HIV neutralization in a luciferase reporter gene assay. Methods in molecular biology 485, 395–405 (2009). 19020839 10.1007/978-1-59745-170-3_26

[82] Sarzotti-Kelsoe M , Optimization and validation of the TZM-bl assay for standardized assessments of neutralizing antibodies against HIV-1. J Immunol Methods 409, 131–146 (2014). 24291345 10.1016/j.jim.2013.11.022 PMC4040342

[83] C. A. Todd , Development and implementation of an international proficiency testing program for a neutralizing antibody assay for HIV-1 in TZM-bl cells. J Immunol Methods 375, 57–67 (2012). 21968254 10.1016/j.jim.2011.09.007 PMC3332116

[84] Punjani A, Rubinstein JL, Fleet DJ, Brubaker MA, cryoSPARC: algorithms for rapid unsupervised cryo-EM structure determination. Nature methods 14, 290–296 (2017). 28165473 10.1038/nmeth.4169

[85] Abramson J , Accurate structure prediction of biomolecular interactions with AlphaFold 3. Nature 630, 493–500 (2024). 38718835 10.1038/s41586-024-07487-w PMC11168924

[86] Abanades B , ImmuneBuilder: Deep-Learning models for predicting the structures of immune proteins. Commun Biol 6, 575 (2023). 37248282 10.1038/s42003-023-04927-7 PMC10227038

[87] Meng EC , UCSF ChimeraX: Tools for structure building and analysis. Protein science : a publication of the Protein Society 32, e4792 (2023). 37774136 10.1002/pro.4792 PMC10588335

[88] Casanal A, Lohkamp B, Emsley P, Current Developments in Coot for Macromolecular Model Building of Electron Cryo-microscopy and Crystallographic Data. Protein science : a publication of the Protein Society, (2019). 10.1002/pro.3791 PMC7096722 31730249

[89] Afonine PV , Real-space refinement in PHENIX for cryo-EM and crystallography. Acta Crystallogr D Struct Biol 74, 531–544 (2018). 29872004 10.1107/S2059798318006551 PMC6096492

[90] Pettersen EF , UCSF Chimera--a visualization system for exploratory research and analysis. J Comput Chem 25, 1605–1612 (2004). 15264254 10.1002/jcc.20084

[91] Henderson R , Disruption of the HIV-1 Envelope allosteric network blocks CD4-induced rearrangements. Nat Commun 11, 520 (2020). 31980614 10.1038/s41467-019-14196-w PMC6981184

[92] Shapovalov MV, Dunbrack RL Jr., A smoothed backbone-dependent rotamer library for proteins derived from adaptive kernel density estimates and regressions. Structure 19, 844–858 (2011). 21645855 10.1016/j.str.2011.03.019 PMC3118414

[93] Krissinel E, Stock-based detection of protein oligomeric states in jsPISA. Nucleic Acids Res 43, W314–319 (2015). 25908787 10.1093/nar/gkv314 PMC4489313

[94] Ozorowski G , Open and closed structures reveal allostery and pliability in the HIV-1 envelope spike. Nature 547, 360–363 (2017). 28700571 10.1038/nature23010 PMC5538736

[95] Clopper CJ, Pearson ES, The Use of Confidence or Fiducial Limits Illustrated in the Case of the Binomial. Biometrika 26, 404–413 (1934).

[96] Lin LI, A concordance correlation coefficient to evaluate reproducibility. Biometrics 45, 255–268 (1989). 2720055

[97] Hyndman RJ, Fan Y, Sample Quantiles in Statistical Packages. The American Statistician 50, 361–365 (1996).

[98] Liang KY, Zeger SL, Longitudinal data analysis using generalized linear models. Biometrika 73, 13–22 (1986).

[99] Hunter JD, Matplotlib: A 2D Graphics Environment. Computing in Science & Engineering 9, 90–95 (2007).

[100] Waskom ML, seaborn: statistical data visualization. Journal of Open Source Software 6, 3021 (2021).

[99] DeKosky BJ , In-depth determination and analysis of the human paired heavy- and light-chain antibody repertoire. Nat Med 21, 86–91 (2015). 25501908 10.1038/nm.3743

[100] Lee JH , Vaccine genetics of IGHV1-2 VRC01-class broadly neutralizing antibody precursor naive human B cells. NPJ Vaccines 6, 113 (2021). 34489473 10.1038/s41541-021-00376-7 PMC8421370

[101] Kulp DW , Structure-based design of native-like HIV-1 envelope trimers to silence non-neutralizing epitopes and eliminate CD4 binding. Nat Commun 8, 1655 (2017). 29162799 10.1038/s41467-017-01549-6 PMC5698488

[102] Kabat EA, Wu TT, Bilofsky H, Unusual distributions of amino acids in complementarity-determining (hypervariable) segments of heavy and light chains of immunoglobulins and their possible roles in specificity of antibody-combining sites. The Journal of biological chemistry 252, 6609–6616 (1977). 408353

[103] Kabat EA, Sequences of proteins of immunological interest. NIH publication (U.S. Dept. of Health and Human Services, Public Health Service, National Institutes of Health, Bethesda, Md. (Bethesda, 20892), ed. 5th, 1991).

