# Supplementary material for: Vaccination with mRNA-encoded nanoparticles drives early maturation of HIV bnAb precursors in humans
Source: Science. Author manuscript; Available in PMC 2026 May 12. (PMC13164876; doi:10.1126/science.adr8382)
Supplement: Supp [file NIHMS2164451-supplement-Supp.docx]

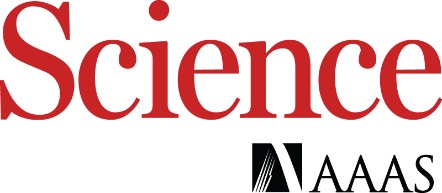


Supplementary Materials for

**Vaccination with mRNA-encoded nanoparticles drives early maturation of HIV bnAb precursors in humans**

Jordan R. Willis, Madhu Prabhakaran, Michelle Muthui, Ansuya Naidoo, Troy Sincomb, Weiwei Wu, Christopher A. Cottrell, Elise Landais, Allan C. deCamp, Nahid R. Keshavarzi, Oleksandr Kalyuzhniy, Jeong Hyun Lee, Linda M. Murungi, Wilfrida A Ogonda, Nicole L. Yates, Martin M. Corcoran, Swastik Phulera, Joel Musando, Amanda Tsai, Gabrielle Lemire, Yiakon Sein, Michael Muteti, Praveen Alamuri, Jennifer A. Bohl, Drienna Holman, Sunny Himansu, Brett Leav, Caroline Reuter, Li-An Lin, Baoyu Ding, Chunla He, Walter L. Straus, Kellie J. MacPhee, Isabel Regadas, Diana V. Nyabundi, Ruth ChirChir, Omu Anzala, John N. Kimotho, Caleb Kibet, Kelli Greene, Hongmei Gao, Erica Beatman, Kiara Benson, Dominick Laddy, David M. Brown, Rhianna Bronson, Jalen Jean-Baptiste, Suprabhath Gajjala, Zahra Rikhtegaran-Tehrani, Alison Benner, Mukundhan Ramaswami, Danny Lu, Nushin Alavi, Sonya Amirzehni, Michael Kubitz, Ryan Tingle, Erik Georgeson, Nicole Phelps, Yumiko Adachi, Alessia Liguori, Claudia Flynn, Katherine McKenney, Xiaoya Zhou, David C. Owour, Sharon A. Owuor, Soo-Young Kim, Michael Duff, Ju Yeong Kim, Grace Gibson, Sabyasachi Baboo, Jolene Diedrich, Torben Schiffner, Marisa Shields, Mabela Matsoso, Jennifer Santos, Kristen Syvertsen, Allison Kennedy, Melissa Schroeter, Johan Vekemans, John R. Yates III, James C. Paulson, Ollivier Hyrien, Adrian B. McDermott, Pholo Maenetje, Julien Nyombayire, Etienne Karita, Rosine Ingabire, Vinodh Edward, Vincent Muturi-Kioi, Janine Maenza, Adrienne E. Shapiro, M. Juliana McElrath, Srilatha Edupuganti, Barbara S. Taylor, David Diemert, Gabriel Ozorowski, Richard A. Koup, David Montefiori, Andrew B. Ward, Gunilla B. Karlsson Hedestam, Georgia Tomaras, Devin J. Hunt, Daniel Muema, Devin Sok, Dagna S. Laufer, Sarah F. Andrews, Eunice W. Nduati, William R. Schief

Corresponding authors: [daniel.muema@ahri.org](mailto:daniel.muema@ahri.org) (D.Mu.) or [dsok@ghicfunds.org](mailto:dsok@ghicfunds.org) (D.S.) or [dlaufer@iavi.org](http://dlaufer@iavi.org) (D.S.L.) or [sarah.andrews2@nih.gov](http://sarah.andrews2@nih.gov) (S.F.A) or [ENduati@kemri-wellcome.org](mailto:ENduati@kemri-wellcome.org) (E.W.N.) or [schief@scripps.edu](http://schief@scripps.edu) (W.R.S).

**The PDF file includes:**

Supplementary Text

Figs. S1 to S63

Tables S1 to S78

**Other Supplementary Materials for this manuscript include the following:**

Data S1 to S10

**Supplementary Text**

**G002 detailed findings regarding safety and tolerability**

***Adverse events*.** Sixty of 60 participants (100%) reported solicited AEs, and 45 of 60 participants (75%) reported unsolicited AEs (table S14). Fifty-nine of 60 participants (98.3%) reported local administration site reactogenicity, the most common being pain at the injection site (59/60; 98.3%) and axillary swelling (28/60; 46.7%) (fig. S3 and Data S2). Fifty-seven of 60 participants reported systemic reactogenicity (95%). Fatigue (55/60; 91.7%) and headache (42/60; 70%) were the most common systemic reactogenicity events reported (fig. S4 and Data S3). Overall reactogenicity was transient, generally mild or moderate in severity and resolved in most cases within 1-2 days after onset. Local and systemic reactions were similar between the groups for number and grade of events. Local and systemic reactogenicity were similar between the different doses of IP, for number and grade of events (table S14).

***Local reactogenicity*.** Local injection-site reactogenicity was collected through 7 days post vaccination, as shown in fig. S3, tables S16 and S17, and Data S2. Most local reactogenicity was graded as mild or moderate. Overall local reactogenicity events were experienced in 59/60 participants (98.3%). The percentages of participants with any local reactogenicity within 7 days after injection were 100% (17/17) in Group 1, 100% (17/17) in Group 2, 100% (18/18) in Group 3, and 87.5% (7/8) in Group 4. For all groups, the most common participant-reported Grade 2 local reactogenicity event was pain at injection site (3/17, 17.6 % for Group 1; 8/17, 47.1 % for Group 2; 6/18, 33.3 % for Group 3; and 3/8, 37.5 % for Group 4). Seven participants reported Grade 3 local reactogenicity in the study: 3/17 (17.6%) participants in Group 2 (erythema, pain at the injection site [2 participants]); 2/17 (11.1%) participants in Group 3 (injection site pain in both participants); and 2/8 (25%) participants in Group 4 (injection site pain in both participants).

***Systemic reactogenicity*.**  Systemic reactogenicity was collected through 7 days post vaccination and is shown in fig. S4, tables S16 and S17, and Data S3. Most systemic reactogenicity events were graded as mild or moderate. The percentages of participants with any systemic reactogenicity within 7 days after injection were 100% (17/17) in Group 1, 100% (17/17) in Group 2¸ 100% (18/18) in Group 3 and 62.5% (5/8) in Group 4. The most common Grade 2 events reported for all groups was fatigue (7/17, 41.2% for Group 1; 8/17, 47.1% for Group 2; 9/18, 50% for Group 3; and 3/8, 37.5% for Group 4). Grade 3 systemic reactogenicity events were reported in Group 1 by 4 participants (4/17, 23.5%) with MedDRA preferred terms (PTs) of fatigue, fever, headache and myalgia, in Group 2 by 3 participants (3/17, 17.6%) with PTs of chills, fatigue, headache and myalgia, in Group 3 by 3 participants (4/18, 22.2%) with PTs of chills, fatigue and myalgia, and in Group 4 by 2 participants (2/8, 25%) with PTs of fatigue and nausea.

***Unsolicited adverse events*.** Unsolicited AEs are shown in Data S4 and S5. Unsolicited adverse events were reported through 28 days after each vaccination. COVID-19 was the most common unsolicited adverse event, reported by 23.5% in Group 1, 11.8% in Group 2, 27.8% in Group 3, and 12.5% in Group 4. Two Grade 3 events were reported. Grade 3 urticaria reported in participant G002-758, in Group 1, lasted for 4 days and was then downgraded to Grade 2. Urticaria onset was 27 days after IP and lasted a total of 34 days and was considered related to IP. The second Grade 3 AE was in participant G002-733, also in Group 1, and was COVID-19 infection, with an onset 7 days post IP administration and duration of 12 days; this AE was considered unrelated to IP.

***Unsolicited adverse events related to study procedures***. Unsolicited AEs related to study procedures are shown in table S15. The most common unsolicited AEs related to study procedures were ecchymosis, oral parasthesia, presyncope and haematomas. The reported AEs were associated with the procedures of large-volume phlebotomies, leukapheresis, and fine needle aspiration (FNA) of the axillary lymph nodes.

***Unsolicited skin adverse events*.** Unsolicited skin AEs, judged by site PIs as possibly or probably related to study IP, are summarized in fig. S5 and tables S18 and S19. As described in the main text, there were 14 clinical events of skin AEs with the preferred terms of urticaria, dermatographism (mechanical urticaria) and/or pruritus reported in 11 of 60 participants. Some participants reported a combination of the three above-defined skin AEs.

The breakdown of the skin AEs was as follows:

- pruritus only: 7 events
- urticaria and dermatographism: 4 events
- pruritus and dermatographism: 1 event
- urticaria and pruritus: 1 event
- dermatographism only: 1 event

Seven of 60 participants (12%) reported urticaria and/or dermatographism, of which 6 participants (10% of 60) had symptoms that lasted 6 weeks or more. Three of these 7 also reported pruritus. Four other participants (4 of 60, 6.7%) reported pruritus without reporting urticaria or dermatographism.

Overall, the skin AEs were characterized by intermittent episodes with symptoms that waxed and waned. As described in the Materials and Methods section, skin AEs were considered to be resolved if there were no symptoms present for 14 days continuous days without treatment. Multiple skin adverse events occurring within the same 14-day period were considered part of the same clinical event, and AE, and recorded as such. Any skin AE occurring after 14 days of no symptoms or treatment was recorded as a new onset AE.

All participants took oral antihistamine medications for symptom management, and some participants continued to experience breakthrough intermittent clinical symptoms even while taking a daily antihistamine. One participant, who experienced Grade 3 (severe) symptoms, received a short course of high-dose oral corticosteroid treatment that resulted in significant improvement of symptoms. There were two events (pruritus [n=1] and dermatographism [n=1]) ongoing in two participants after their last in-person study visit (6 months after the last study vaccination). All events ultimately resolved (fig. S5 and table S18).

**G003 detailed findings regarding safety and tolerability**

A total of 44 individuals were assessed for eligibility, and 18 of these enrolled in Rwanda (n=10) and South Africa (n=8) (fig. S2B and table S4). Eight (44.4%) of the participants were females; all participants were of black African origin, and their mean age was 32.4 years (SD 5.96) (table S5). All tested negative for HIV, syphilis, and hepatitis B and C serology at baseline, and none reported behavior that placed them at risk for HIV infection. None presented with medical history or concomitant medications that were considered exclusionary in terms of the study eligibility criteria. All participants were included in the enrolled and safety analysis populations and completed per protocol study follow up visits (tables S4 and S5).

Solicited adverse events (AEs) were reported in all participants (figs. S6 and S7). The majority of local solicited AEs (reported in 15 [83.3 %] participants after each IP administration; most commonly pain and axillary swelling) were grade 1 in intensity, and no grade 3 or higher events were reported (fig. S6 and tables S22 to S24). Systemic solicited AEs (most commonly headache and fatigue) tended to increase in incidence following the second IP administration compared to the first (17 events in nine [50.0 %] participants and 30 events in 13 [72.2 %] participants after the first and second IP administrations, respectively) (fig. S7 and tables S22 to S24). One headache after the second IP administration was grade 3 in intensity for one day of its 4-day duration. Unsolicited AEs were reported in eight (44.4 %) participants during the 28 days following each IP administration (table S22). These were most frequently related to miscellaneous infections. Three events (2 pruritus events and 1 fatigue event) in three (16.7 %) participants were considered related to the IP. The two dermatological AEs of pruritus started 13 and 18 days after the second administration of IP, respectively, and both resolved after eight days and treatment with desloratadine. No grade 3 or higher unsolicited AEs were reported. Fourteen grade 1 or 2 unsolicited AEs were reported in eight (44.4%) participants, including three judged related to IP (table S22). Ten (10) medically attended adverse events (MAAEs) were reported in eight (44.4%) participants from the time of first IP administration until the end of study 24 weeks after the second dose of IP (table S22). All except one MAAE (grade 2 fatigue) were considered unrelated to IP (table S22). No serious adverse events (SAEs) or adverse events of special interest (AESIs) were reported during the study, and no participant discontinued IP or was withdrawn from the study due to an AE. There were no clinically significant safety laboratory findings, and no post-dose safety laboratory trends observed in the study cohort after either dose of IP administered (table S22).

**G002 summary narratives of skin AEs for each affected participant**

***Group 1: G002-254***

Participant has a history of previous COVID-19 infection, seasonal allergies, and eczema with a baseline use of antihistamines (loratadine, cetirizine, famotidine), and triamcinolone topical cream. COVID-19 vaccination: Johnson & Johnson x1, Moderna booster x2 (mild arm pain).

Participant G002-254 developed Grade 1 generalized pruritus 40 days post-Dose 1 of IP (eOD: Group 1). Started diphenhydramine which improved symptoms. Participant experienced ongoing intermittent symptoms of generalised itching with a decreasing frequency. Itching resolved with use of the antihistamine, but resumed when doses were missed, and lasted approximately 30 minutes each episode. Participant reduced the use of diphenhydramine but continued with baseline antihistamines. Itching worsened if a dose of baseline antihistamines was missed. The event was considered resolved after 165 days, when participant had more than 14 days without symptoms while on baseline medication.

***Group 1: G002-479***

Medical history of childhood eczema, and COVID-19 infection. COVID-19 vaccination prior to study: Moderna x3.

Participant G002-479 experienced event of urticaria with dermatographism, 19 days post-Dose 1 of IP (eOD: Group 1). Symptoms included generalized urticaria, itching and dermatographism. Started on diphenhydramine on their own, which improved symptoms. If medication was stopped, symptoms would return within same day of missed dose. A clinical decision was taken by the site PI, in agreement with the participant, that the participant would not receive the Dose 2 of IP. Frequency and duration of episodes decreased with time, and participant was able to stop taking the antihistamine. The urticaria resolved after 45 days duration, and the dermatographism resolved after 57 days duration.

***Group 1: G002-516***

The participant has a history of seasonal allergies, for which he used loratadine as needed. COVID-19 vaccination prior to study: Moderna x2 (injection-site pain, fever, body aches), Pfizer x1 (body aches).

Participant G002-516 developed Grade 2 generalized urticaria (arms, back, chest, face) and associated dermatographism 10 days (about 1 and a half weeks) post-Dose 2 of IP (eOD: Group 1). He began taking diphenhydramine, cetirizine and topical corticosteroid cream, as directed by the study team, which improved but did not resolve symptoms. Participant was seen in clinic and directed by site PI to start famotidine, 4 days after onset, and stop the cetirizine and topical corticosteroid. The symptoms improved significantly after a week of adding famotidine. Participant elected to stop all treatment 5 weeks after the onset of the event, however continued having intermittent mild Grade 1 episodes about 2-3 times per week, which were self-limited and did not require the use of medication. The urticaria resolved after 121 days (about 4 months) of intermittent symptoms. The dermatographism continued after the last study visit; symptoms were intermittent and decreased in frequency and severity as the event progressed and did not require medication. The event of dermatographism resolved after 369 days (about 1 year).

***Group 1: G002-758***

No relevant medical history. COVID-19 vaccinations: Moderna x3.

Participant G002-758 experienced Grade 2 urticaria and dermatographism which started 27 days post-Dose 1 of IP (eOD: Group 1). Episodes were self-limited and transient, affecting one part of the body at a time. Treated with fexofenadine which improved symptoms. The participant went on to receive Dose 2 of IP (eOD: Group 1), 57 days after Dose 1. 7 days post-Dose 2 of IP, the symptoms worsened and became generalized with erythematous plaques on hands, legs and trunk. The event was upgraded to Grade 3 for 4 days. He was treated with a 6-day tapering course of oral prednisone and famotidine, for worsening lesions on one hand. Symptoms improved progressively, and participant was able to discontinue antihistamine. Urticaria was considered resolved 34 days after onset, and dermatographism resolved 84 days after onset.

***Group 1: G002-834***

Medical history included symptomatic COVID-19 and exercise-induced asthma. COVID-19 vaccination: Pfizer x2 (injection-site pain, arthralgia), Moderna x1 (arthralgia, myalgia, fatigue, injection-site pain).

Participant G002-834 experienced an event of mild pruritus with dermatographism, with onset 12 days post-Dose 2 of IP (eOD, eOD: Group 1). Symptoms occurred daily, lasting a few hours each episode, and worsening at night. Started on daily oral cetirizine, which improved symptoms. Attempted to stop antihistamine after 6 weeks, but symptoms recurred 8 days after stopping. Restarted cetirizine for an additional 6 weeks and had mild intermittent clinical symptoms. Was then able to stop antihistamine and remained symptom free. The event was considered resolved after 107 days.

***Group 1: G002-969***

Participant has a medical history of seasonal allergies and eczema, which he treats with cetirizine regularly and diphenhydramine as needed. Previous COVID-19 vaccination and reactogenicity prior to study: Pfizer x3 doses (body aches, dizziness, shaky legs) prior to study, and Moderna half dose booster (mild fever and fatigue).

Participant G002-969 developed chills and Grade 2 generalized urticaria with pruritus, affecting multiple body sites, 11 days post-Dose 2 of IP (eOD, eOD: Group 1). Symptoms were worse at night and seemed exacerbated by ingestion of alcohol and sugary foods, and hot showers. Urticaria and pruritus symptoms were controlled with cetirizine (as needed, was not daily) and gradually improved to Grade 1 before eventually resolving. The participant returned to baseline antihistamines, and the event was considered resolved after a total event duration of 69 days.

***Group 2: G002-577***

Medical history of urticaria to environmental allergen from 2009-2012. COVID-19 vaccination: Pfizer x2 (injection-site pain, fatigue), Moderna x1 (injection-site pain).

Participant G002-577 experienced an AE of Grade 2 pruritus, 13 days post-Dose 2 of IP (eOD, Core: Group 2). Generalized itchiness with erythema, without rash. Affecting different parts of the body sporadically, including gluteal area, chest, legs, and hands. Participant started on diphenhydramine, which provided relief. Symptoms progressively improved, and participant was able to stop antihistamines, with the event resolving after 27 days duration.

***Group 2: G002-943***

Participant has a medical history of childhood eczema, and contact dermatitis to perfumes, about once a year. COVID-19 vaccinations prior to study: Moderna x2, Pfizer x1. (fever, general discomfort, chills, dizziness, bilateral lymph node swelling for 1 week).

Participant G002-943 developed a Grade 2 event of Pruritus over the left breast 39 days (about 1 and a half months) post-Dose 1 of IP (eOD: Group 2), which improved over the course of 2-3 weeks with the use of diphenhydramine as needed. Participant opted to continue with IP and received Dose 2 (core-g28v2). Two days post-Dose 2 of IP, participant began itching and noted a rash on her chest that worsened throughout the day requiring a visit to urgent care, where she was prescribed 1% triamcinolone cream for possible contact dermatitis. The rash spread to other parts of the body, and the participant added loratadine to the diphenhydramine. The symptoms continued intermittently, which allowed the participant to reduce antihistamine use. The event was considered resolved after 68 days (about 2 months 1 week), and the participant did not require antihistamines. Approximately 30 days (about 4 and a half weeks) after resolution of the previous event, the participant experienced another event of pruritic rash, with intermittent symptoms and a maximum of Grade 2 severity, on various body areas, including the back, legs, and bra line. Participant resumed antihistamine and topical corticosteroid use for symptoms, which were waxing and waning over the course of 16 weeks (about 3 and a half months). Symptoms gradually improved; the participant returned to baseline antihistamine usage; and the second event was considered resolved after 116 days (about 4 months).

***Group 3: G002-462***

Participant had no relevant medical history. COVID-19 vaccination prior to study: Moderna x2, Pfizer x1.

Participant G002-462 developed Grade 1 pruritus 38 days after Dose 1 of IP (eOD: Group 3). Transient episodes of itching localized to various parts of body, including arms, leg, and back. Did not require treatment and did not affect participant daily activities at the time. Participant agreed to proceed with Dose 2 of IP (eOD: Group 3). Developed new onset Grade 2 urticaria, with associated pruritus and dermatographism, involving the neck, chest, back, upper limbs, and lower limbs, 7 days post-Dose 2 of IP. Treated with cetirizine, diphenhydramine, and famotidine. The urticaria resolved with treatment after 3 days, however pruritus and dermatographism remained ongoing. Dermatographism symptoms were intermittent and resolved after 70 days, while on daily treatment. Multiple flare-ups of pruritus occurred when participant attempted to discontinue cetirizine; flare-ups resolved after restarting treatment. Participant also developed an intermittent, Grade 1 macular rash on forearms bilaterally, 12 days (1 week and 5 days) post-Dose 2 of IP, which lasted 41 days (about 1 and a half months) prior to resolution. Participant also experienced 2 episodes of Grade 2 urticaria, 101- and 159-days post-Dose 2 of IP, respectively. Both episodes lasted 1 day, and participant was on as-needed antihistamine treatment for pruritus as above, at the time.

***Group 3: G002-595***

No relevant medical history. Previous COVID-19 vaccination and reactogenicity: AstraZeneca x2 doses, Pfizer booster (mild injection-site pain, fatigue) prior to study enrolment.

Participant G002-595 developed Grade 1 pruritus on the back and lower limbs, 28 days post Dose 3 of IP (eOD, eOD, Core: Group 3). Episodes were intermittent, occurring approximately every other day and lasting approximately 1 hour each time. Experienced one episode of dermatographism, lasting 1 day, during the pruritus event. Treated with antihistamines for 1 day but stopped voluntarily. Total duration of pruritus event was 48 days, and symptoms resolved without further medical intervention.

A decision was made by the Safety Monitoring Committee (SMC) to not proceed with Dose 3 (core-g28v2 60mer mRNA-LNP) for this participant.

Participant reduced antihistamine treatment gradually, to daily cetirizine only, and continued to have episodes of Grade 1 pruritus which improved slowly and gradually from 2-3x per week to once every 2-3 weeks over the course of 9 months. Participant eventually reduced cetirizine to as needed, and the event of pruritus resolved completely after 295 days (61 days post-last study visit).

***Group 3: G002-632***

Medical history of gastroesophageal reflux disease, treated with famotidine. COVID-19 vaccination: Moderna x2 (injection-site pain), Pfizer x1 (injection-site pain and “brain fog”).

Participant G002-632 experienced an event of Grade 1 pruritus 20 days post-Dose 1 of IP (eOD: Group 3). Described as localized pruritus on the extremities and trunk lasting up to 1 minute, at least once per day. The event resolved without treatment after a duration of 22 days.

The participant went on to receive Dose 2 of IP (eOD: Group 3) and then experienced another event of pruritus 9 days post-IP. Itching on random areas of body which lasted up to 1 minute per episode before resolving. There was no associated rash. Participant started loratadine, as directed by the site PI, which improved symptoms. Pruritus continued, becoming less frequent, and improving when treated with antihistamines. The event resolved after a total duration of 66 days.

The SMC advised that the participant should not receive Dose 3 of IP (Core: Group 3).

**G002 durability of serum antibody binding responses**

Considering eOD-GT8 60mer as a model vaccine antigen, we found it notable that the serum IgG binding responses to the eOD-GT8 monomer and 60mer showed no significant reduction between week 8 (the day of the second and last vaccination) and week 24 (the last timepoint tested) (table S77; for both antigens the AUC values were numerically but not significantly higher at week 24). Furthermore, serum IgG responses to the eOD-GT8 CD4bs epitope declined by only 1.8-fold over that time period (table S77). Although high serum antibody binding responses are not necessarily desirable at the early or intermediate stages of our sequential vaccination strategy due to the potential for epitope masking and interference with heterologous boosting, high and durable serum antibody binding and neutralizing responses are required at the end stage of the regimen once bnAbs have developed (fig. S1). The fact that mRNA-LNP delivery of the eOD-GT8 60mer at weeks 0 and 8 provided highly durable responses at least to week 24 indicates that mRNA-LNP delivery of nanoparticle antigens should be evaluated for the ability to generate long-lived serum antibody responses in general, whether at the end of a germline-targeting sequential vaccination regimen to induce bnAbs to HIV or as part of a simpler regimen with a single antigen to induce protective responses to a pathogen less challenging than HIV.

**Timing of the next boost**

The data from IAVI G002 on responses to the core-g28v2 60mer mRNA-LNP boost at week 8 following a single eOD-GT8 60mer mRNA-LNP prime (eOD→core regimen) has implications for the optimal timing of the next boost to follow that regimen. The next boost is envisaged to be an N276-lacking native-like trimer. Several factors considered together suggest that delivering the next boost at week 24 or later rather than at week 16 would be advantageous. Those factors include:

1. the higher frequency of VRC01-class IgG memory B cells at week 24 compared to week 16 (2.8-fold higher; *P*=0.0003) (Fig. 4D and table S57)
2. the higher degree of SHM in VRC01-class IgG memory B cells at week 24 (e.g. median SHM in VH1-2 gene of 9.2% at week 24 compared to 7.7% at week 16; *P*<0.0001) (Fig. 5, A and B, and table S61)
3. the higher 90th percentile number of key VRC01-class HC residues at week 24 (5.0 versus 4.0 at week 16; *P*<0.001) (Fig. 5E and table S61)
4. the higher percentage of “elite” VRC01-class IgG B cells with >4 key VRC01-class HC residues among all VRC01-class IgG B cells (median of 9% at week 24 compared to 3% at week 16; *P*=0.0004) (Fig. 7A and table S67)
5. the higher frequency of “elite” VRC01-class IgG B cells with >4 key VRC01-class HC residues among all IgG B cells at week 24 (1 in 8,400 compared to 1 in 27,000 at week 16; *P*<0.0001) (Fig. 7C and table S67)
6. the similar or higher affinities of “selected” mAbs (top-ranked mAbs per participant by number of key HC residues) for N276-lacking trimer boost candidates at week 24 compared to week 16 (Fig. 8A and table S78)
7. the maintenance of low serum IgG reactivity to N276-lacking trimers at week 24, indicating low potential for serum IgG interference with the next boost (fig. S60 and table S72).

**Affinity associated with effective boosting**

Having determined that eOD®core was an effective heterologous prime-boost regimen VRC01-class responses, we considered that the binding characteristics of core-g28v2 with the VRC01-class BCRs isolated after eOD-GT8 60mer priming would provide insight into the monovalent interactions associated with a successful boost by a multivalent nanoparticle antigen in humans. Among IgG B cells sorted as specific for the core-g28v2 CD4bs at week 8 after priming with eOD-GT8 60mer mRNA-LNP, the "random" and "selected" VRC01-class mAb populations both had 100% detectable binders to core-g28v2 and had similar median *K*_D_s of 4.6 µM and 3.2 µM, respectively (Fig. 6A). Median *k*_off_ values were 0.15 s^-1^ in both cases, and median *k*_on_ values were 3.8×10^4^ M^-1^s^-1^ and 3.7×10^4^ M^-1^s^-1^, respectively (fig. S43). These data provided population-level monovalent binding affinity and kinetics parameters associated with an effective boost by a highly multivalent nanoparticle in the context of low epitope-specific serum IgG binding to the boost immunogen in humans (evidence for low serum IgG interference at week 8 was discussed in the main text). Whether the overall or elite VRC01-class responses at weeks 16 and 24 derived from VRC01-class IgG B cells with binding characteristics of the overall week 8 population or high- or low-affinity subsets of that population is not known.

These population-level binding characteristics associated with a successful heterologous boost targeting a specific class of B cells in humans may provide useful benchmarks for development of sequential vaccination regimens. Notably, VRC01-class BCR populations with median *K*_D_ values for core-g28v2 in the single-digit micromolar range and relatively fast kinetics were sufficient for effective boosting by core-g28v2 60mer mRNA-LNP in the context described. Similar median affinities were found among BCRs with detectable affinity for core-g28v2 whether those BCRs were isolated by sorting with the priming antigen eOD-GT8 (fig. S33) or the boosting antigen core-g28v2 (fig. S43); however, the percentage of detectable binders was substantially lower among the BCRs sorted with eOD-GT8 probes, which represented a typical scenario encountered when trying to identify candidates for the next immunogen in a germline-targeting vaccine sequential vaccine.

Low percentages of binders and modest affinities for core-g28v2 were also observed for eOD-GT8-probe-sorted VRC01-class BCRs either induced in the IAVI G001 trial by eOD-GT8 60mer protein and AS01_B_ (20% binders with binder geomean *K*_D_ value of 3.5 µM after a single vaccination with eOD-GT8 60mer (*26*)), or induced in stringent mouse models by eOD-GT8 60mer mRNA-LNP (*26, 27*) or eOD-GT8 60mer protein and Sigma adjuvant (*26*) (e.g. in the SE09 mouse 6 weeks after a single eOD-GT8 60mer mRNA-LNP priming immunization, 28% of eOD-GT8-sorted VRC01-class BCRs had detectable affinity for core-g28v2, with binder geomean *K*_D_ value of 7.6 µM (*26*)). In both mouse models, core-g28v2 60mer boosting after eOD-GT8 60mer priming was effective for driving further maturation of VRC01-class responses in conditions of low epitope-specific serum IgG binding to the boost (*26, 27*).

In sum, our findings here in IAVI G002 and in mouse studies indicate that populations of previously primed bnAb-precursor B cell populations with median monovalent affinities in the single-digit micromolar range for a boost antigen can be boosted effectively when that antigen is arrayed on a highly multivalent nanoparticle and epitope-specific serum IgG binding to the boost is low. The fact that week 8 affinities for core-N276 did not meet this benchmark indicated that boosting with core-N276 60mer mRNA-LNP in place of core-g28v2 60mer mRNA-LNP likely would not have been effective in humans.

**Fig. S1. Conceptual schematic for germline-targeting vaccine design.** **(A)** The induction of protective bnAbs in serum requires: (i) stimulating rare bnAb-precursor naive B cells that have specific genetic features shared for each bnAb class but nevertheless have diverse sequences and genetic properties within and across vaccine recipients and are typically difficult to stimulate with wild-type HIV proteins; (ii) selecting for substantial SHM to gain affinity for a relatively conserved surface patch (epitope) on HIV Env; and (iii) generating long-lived plasma cells that secrete bnAbs into serum. **(B)** In the germline-targeting vaccine design strategy, the priming immunogen stimulates bnAb-precursor naïve B cells and produces a first pool of bnAb-precursor germinal center (GC) and memory B cells; successive immunizations with heterologous boost immunogens having structures increasing more similar to wild-type HIV Env (shepherding immunizations) each stimulate the diverse pool of bnAb-precursor GC and memory B cells produced by the prior immunization and further increase SHM toward bnAb development in the GC and memory compartments; lastly, one or more polishing immunizations drives mature-bnAb GC and memory B cells to differentiate into long-lived plasma cells that secrete bnAbs into serum. Plasma cells that secrete antigen-specific IgG will also be generated at each stage. Although high titers of serum bnAbs are the end goal, at the beginning of each stage, high or even modest serum titers of antibodies that bind to the epitope of interest on the new immunogen are not desirable because such antibodies could mask the boost immunogen (*21-23*).

**Fig. S2. Consort diagrams for IAVI G002 (A) and IAVI G003 (B).**

**Fig. S3. IAVI G002 local solicited adverse events by group, last vaccine received, and maximum severity.**

**Fig. S4. IAVI G002 systemic solicited adverse events by group, last vaccine received, and maximum severity.**

**
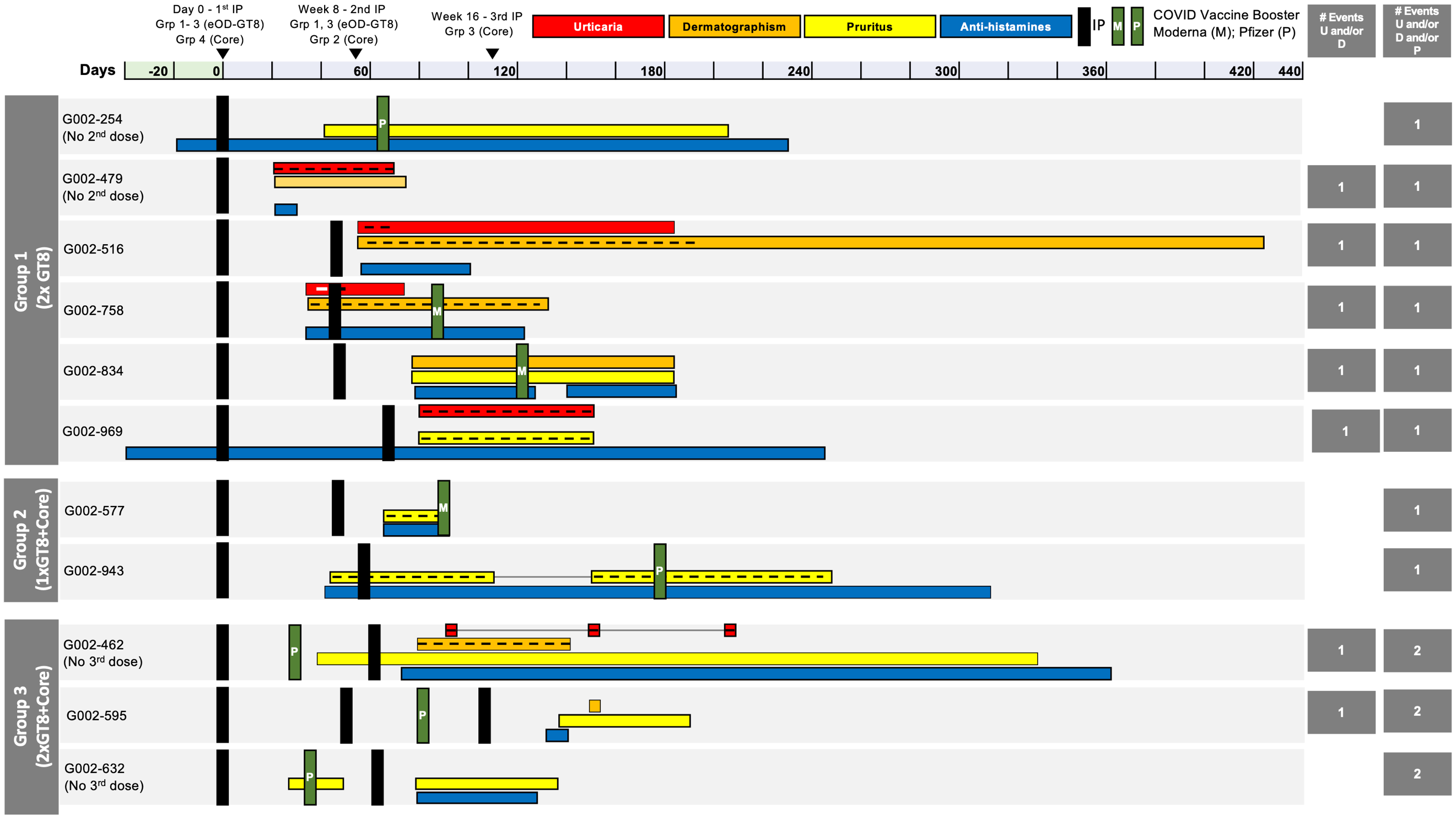
**

**Fig. S5. IAVI G002 overview of skin adverse events.** Skin adverse event type (urticaria, red; dermatographism, orange; pruritus, yellow), duration, and severity (grade 1, solid color rectangle; grade 2, dashed black line inside colored rectangle; grade 3, white line for G002-758 shows the single grade 3 skin AE in the trial) for each of the 14 events experienced by 11 trial participants, with antihistamine use (blue). G002 vaccinations are indicated by vertical black bars, and COVID-19 mRNA-LNP vaccinations during study participation are indicated by vertical green bars for either Moderna (M) or Pfizer/BioNtech (P). COVID-19 vaccination histories prior to enrollment are provided in tables S18 and S21. The number of events per participant are shown at far right, for case definition 1 (urticaria and/or dermatographism and/or pruritus) or case definition 2 (urticaria and/or dermatographism). For G002-943 and G002-462, the gray lines linking periods of pruritus (G002-943) or urticaria (G002-462) indicate that different episodes were considered a single clinical event due to ongoing treatment, per our definition of a clinical event.

**Fig. S6. Summary of local solicited adverse events from the last vaccine received and maximum severity after eOD-GT8 60mer mRNA-LNP vaccination in IAVI G003 (African populations) and IAVI G002 (North American populations).** Data from IAVI G002 only includes participants from group 1, who received the same regimen as IAVI G003 participants, that is, two vaccinations with 100 µg eOD-GT8 60mer mRNA-LNP, 8 weeks apart.

**Fig. S7. Summary of systemic solicited adverse events from the last vaccine received and maximum severity after eOD-GT8 60mer mRNA-LNP vaccination in IAVI G003 (African populations) and IAVI G002 (North American populations).** Data from IAVI G002 only includes participants from group 1, who received the same regimen as IAVI G003 participants, that is, two vaccinations with 100 µg eOD-GT8 60mer mRNA-LNP, 8 weeks apart.

**Fig. S8. Serum IgG antibody binding responses to eOD-GT8 60mer and related probes, for recipients of eOD-GT8 60mer mRNA-LNP in G002 and G003 or eOD-GT8 60mer protein and AS01_B_ in G001.** Response rates (left) and magnitudes (right) are shown for eOD-GT8 60mer [(A) and (B)], eOD-GT8 monomer [(C) and (D)], eOD-GT8-KO11 monomer [(E) and (F)], eOD-GT8 CD4bs [(G) and (H)], and lumazine synthase [(I) and (J)]. Response magnitudes are expressed as area under the titration curve (AUTC) in (B), (D), (F), (J). For the eOD-GT8 CD4bs (H), response magnitude is expressed as ΔAUTC = AUTC_eOD-GT8_ minus AUTC_eOD-GT8-KO11_. On the left, in (A), (C), (E), (G), and (I), circles represent the response rate, and lines indicate the accompanying 95% Wilson confidence intervals. On the right, in (B), (D), (F), (H), and (J), each symbol represents a single participant at one timepoint, and the horizontal lines denote the median, 25% and 75% quantiles. For G001, data from low (20 µg) and high (100 µg) dose groups, which had similar serum IgG responses (*15*), were pooled. For G002, data from groups receiving identical treatment at earlier time points were pooled and analyzed together until their vaccine regimens diverged at later time points. Statistical analyses are presented in tables S31 to S34.

**Fig. S9. BAMA assay concordance analyses between KAVI Institute of Clinical Research and the transferring laboratory at the Center for Human Systems Immunology in Duke University on shared samples from the IAVI G001 clinical trial.** Data shown exclude baseline values. Statistical analyses used Lin’s concordance correlation coefficient (CCC) and its components Accuracy and Precision. BAMA experiments for IAVI G003 were performed at the KAVI laboratory.

**Fig. S10. Serum IgG antibody binding responses to core-g28v2 60mer and related probes, for recipients of all regimens in G002.** Response rates (left) and magnitudes (right) are shown for core-g28v2 60mer [(A) and (B)], core-g28v2 monomer [(C) and (D)], core-g28v2-KO11b monomer [(E) and (F)], core-g28v2 CD4bs [(G) and (H)], and lumazine synthase [(I) and (J)]. Response magnitudes are expressed as area under the titration curve (AUTC) in (B), (D), (F), (J). For the core-g28v2 CD4bs (H), response magnitude is expressed as ΔAUTC = AUTC_core-g28v2_ minus AUTC_core-g28v2-KO11b_. On the left, in (A), (C), (E), (G), and (I), circles represent the response rate, and lines indicate the accompanying 95% Wilson confidence intervals. On the right, in (B), (D), (F), (H), and (J), each symbol represents a single participant at one timepoint, and the horizontal lines denote the median, 25% and 75% quantiles. For G002, data from groups receiving identical treatment at earlier time points were pooled and analyzed together until their vaccine regimens diverged at later time points. Statistical analyses are presented in tables S35 and S36.

**A**

**
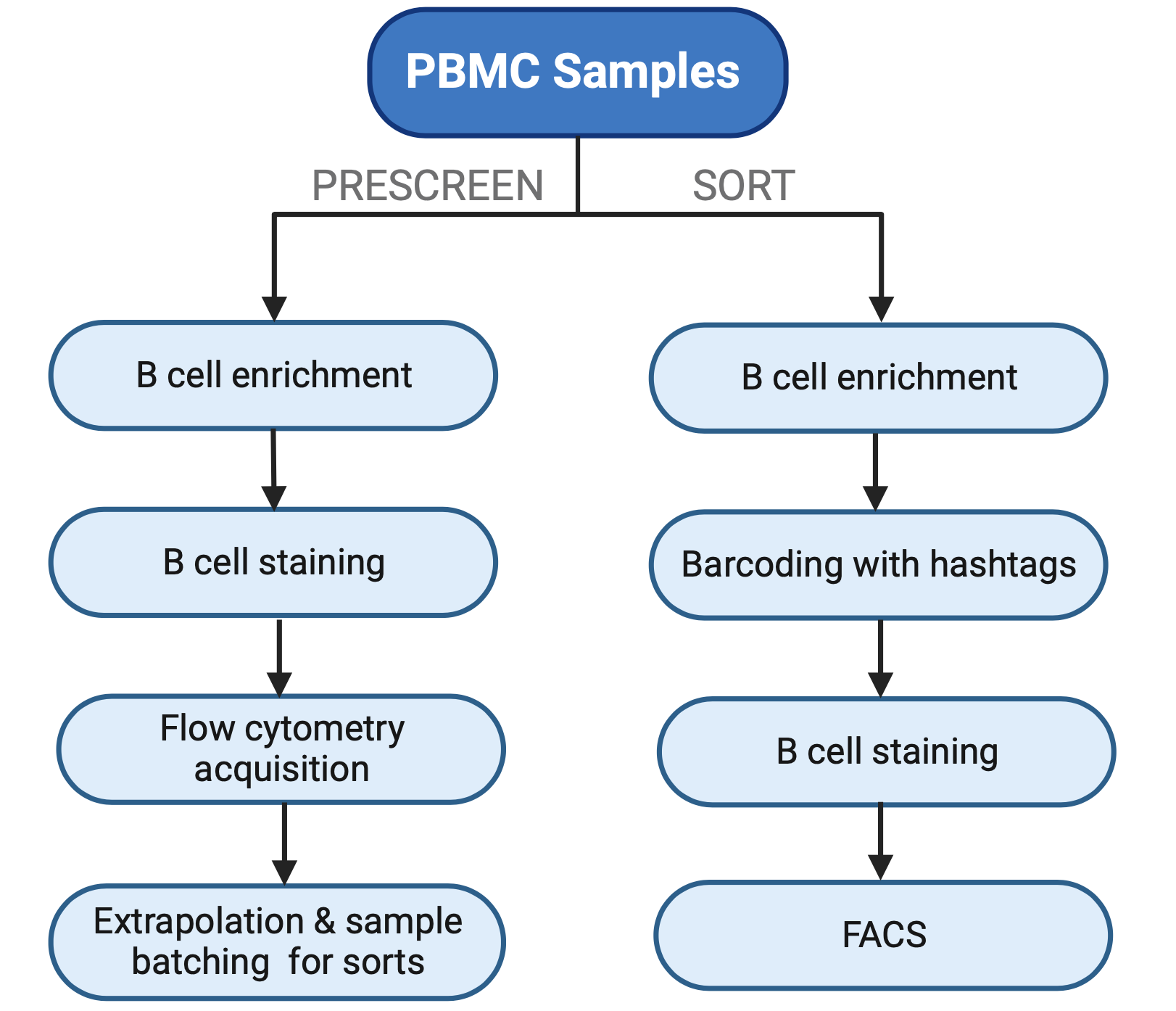
**

**B**

**
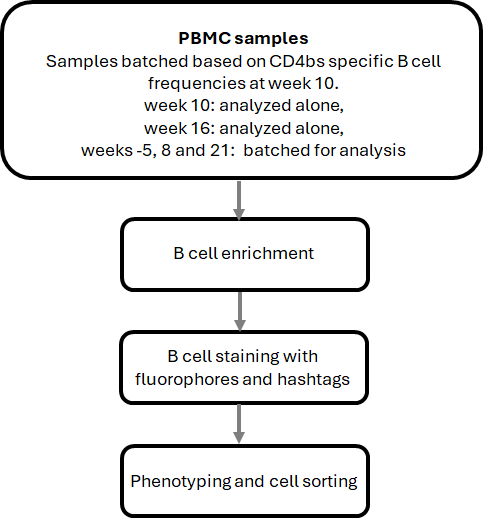
**

**Fig. S11. Sample processing workflows for G002 and G003. (A)** G002. Sample processing and analytical steps involved with prescreens and sorts are shown. **(B)** G003. PBMC samples from the week 10 post-vaccination time-point were pre-screened to determine the CD4bs-specific B cell frequencies for each participant. Participants were then batched for sorting based on the expected number of epitope-specific cells (sort pools). B cells were enriched and stained before phenotyping and sorting.

**Fig. S12. G002 correlation between CD4bs^++^ cells extrapolated and predicted to be sorted from prescreen data and actual CD4bs^++^ cells sorted.** Each dot represents a sample processed with either the eOD-GT8 probeset or the core-g28v2 probeset. Non-parametric Spearman correlation was computed using Prism version 9.3.1 and P and r values are indicated.

**Fig. S13. G002 flow cytometry gating scheme.** Gating strategy to subset different B cell populations and specifically identify antigen-specific and CD4bs-specific (CD4bs^++^) B cells within these populations is shown. The CD4bs^++^ subset indicated with a * was sorted from all post-vaccination timepoints for sequencing. CD4bs^++^ subsets indicated with * and # were sorted from the pre-vaccination timepoint for sequencing.

**Fig. S14. G002 representative flow cytometry plots for one participant for each vaccination group.** Flow cytometry plots showing longitudinal CD4bs-specific IgG memory responses are provided for one representative individual from each of the different vaccination groups (Group 1, G1; Group 2, G2; Group 3, G3; Group 4, G4). Samples were processed with either the eOD-GT8 probeset or Core-g28v2 probeset or both probesets.

**Fig. S15. G003 flow cytometry gating scheme.** The gating strategy to subset different B cell populations and specifically identify eOD-GT8-specific (GT8^++^) and CD4bs-specific (CD4bs^++^) B cells within these populations is shown. The CD4bs^++^ subset indicated with the label “Sorting gate” was sorted from all prevaccination and postvaccination timepoints for sequencing.


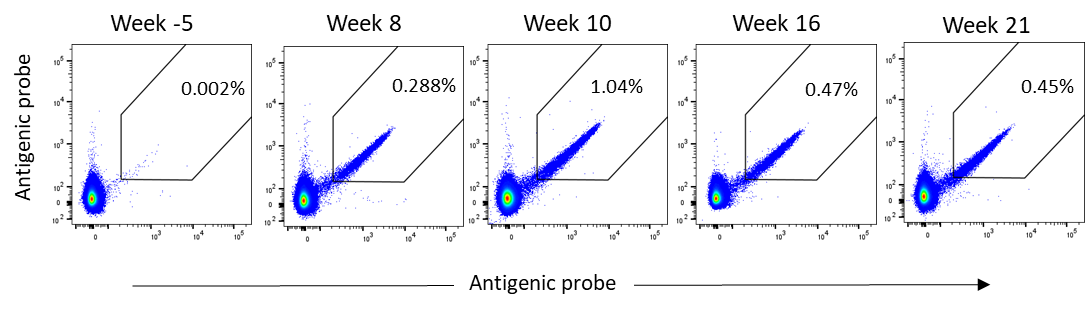


**Fig. S16. G003 flow cytometry plot examples.** These examples show CD4bs-specific IgG memory responses over time post vaccination with eOD-GT8 60mer mRNA-LNP. The KO^-^ gate (not shown) was imposed prior to these eOD-GT8^++^ gates.

**A**

**
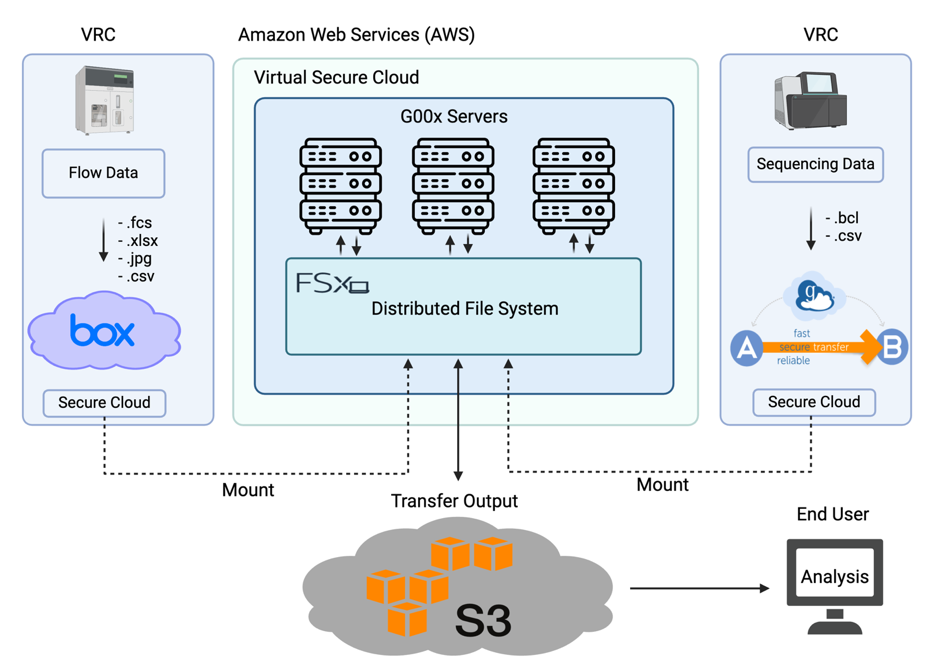
**

**B**


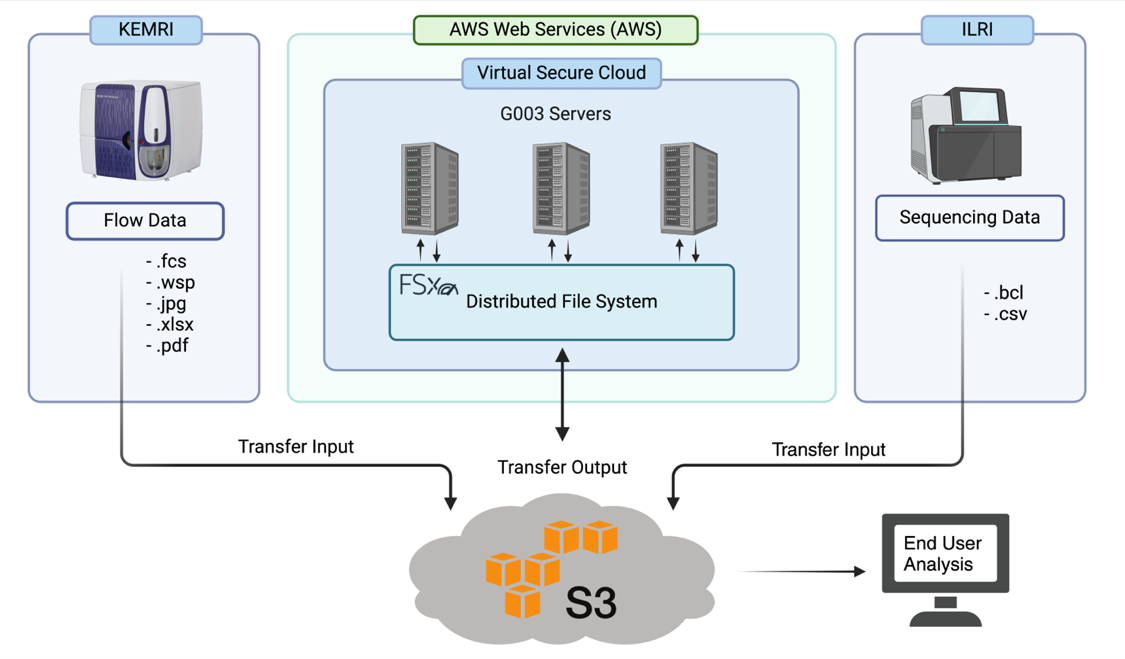


**Fig. S17. Compute infrastructures for G002 and G003. (A)** G002. Flow data (top left) and sequencing data (top right) were mounted to our distributed file system FSx (top center) using Rclone for the flow data and Globus for the sequencing data. All output was copied to an AWS S3 bucket for further analysis. **(B)** G003. The flow data (left), sorted at KEMRI, and the sequencing data (right), sequenced at ILRI, were synced to an AWS S3 bucket. The AWS S3 bucket was synced to the distributed file system FSx (center) mounted to an AWS EC2 instance for the computational analysis. All output was synced to an additional AWS S3 bucket.

**A**

**
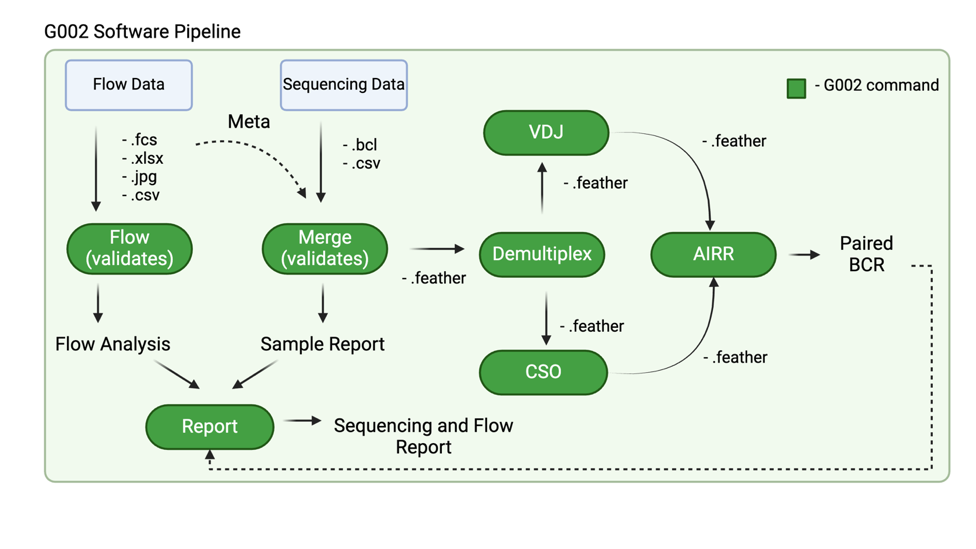
**

**B**


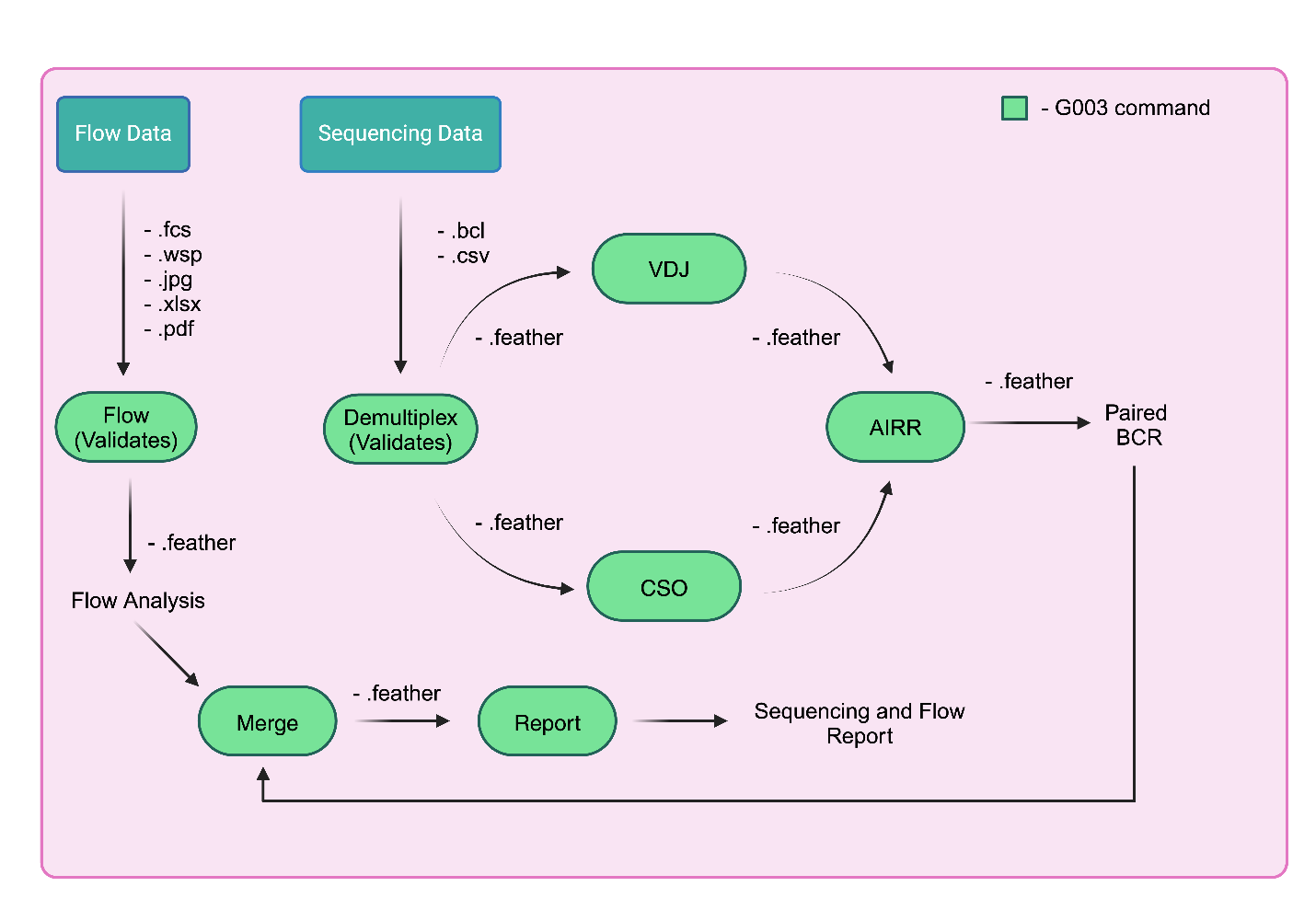


**Fig. S18. Software infrastructures for G002 and G003. (A)** G002. Flow and Sequencing data previously mounted using Rclone or Globus, respectively, were validated for metadata. Sequencing data, which had incomplete metadata, were merged into flow meta data to give complete sequencing dataframe (merge.feather). The merged feather file was demultiplexed with the Demultiplex subcommand according to VDJ or CSO index and was further run through the VDJ and CSO subcommand for VDJ and CSO libraries respectively. The AIRR subcommand joined the CSO and VDJ data to get paired BCR sequences. The Report subcommand took the sequencing and flow data and computed frequencies of VRC01-class B cells and other B cell subsets. **(B)** G003. Flow and sequencing were validated for metadata independently. The sequencing data was validated and demultiplexed with the Demultiplex subcommand according to the VDJ or CSO index. Then, the VDJ and CSO subcommands were run for both VDJ and CSO libraries. The AIRR subcommand joined the VDJ and CSO data and paired the BCR sequences. The validated Flow data was merged with the AIRR paired BCR sequences with the Merge subcommand. This merged.feather was then used to generate the report file with computed frequencies of VRC01-class B cells and B cell subsets.

**
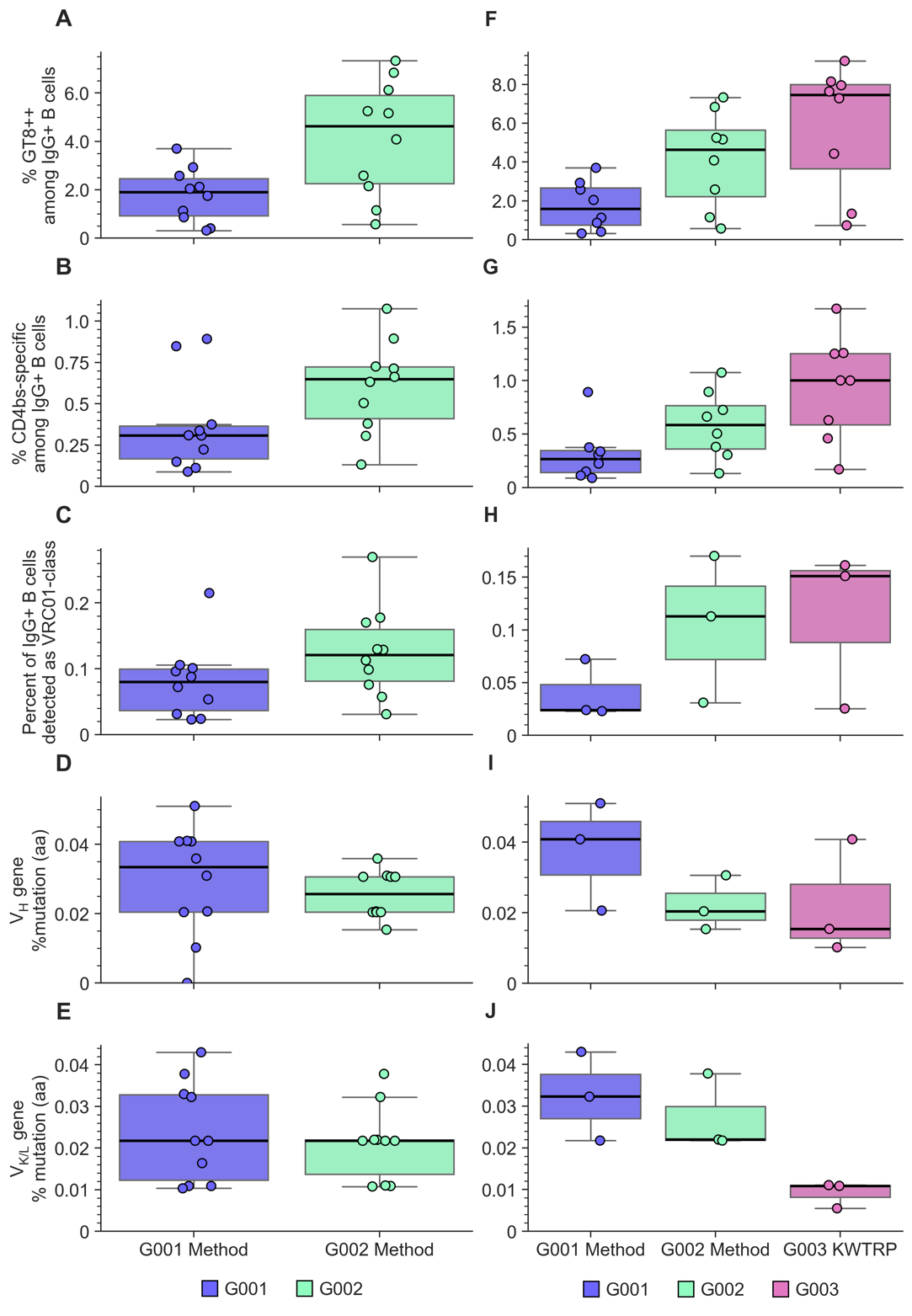
**

**Fig. S19. Analysis of samples from G001 week 10 (two weeks post-boost) using the G002 and G003 B cell workflows and comparing to the published G001 data. (A to E)** Analysis of 10 G001 samples using the G002 workflow with results compared to published results for G001. **(A and B)** Percentages of IgG memory B cells that are eOD-GT8–specific (GT8^++^) (A) or eOD-GT8 CD4bs-specific (B); **(C)** Percentage of VRC01-class IgG B cells among IgG memory B cells; **(D)** VRC01-class BCR V_H_ percent amino acid (aa) mutation, with symbols representing the median per participant per timepoint. **(E)** VRC01-class BCR V_K/L_ percent amino acid (aa) mutation, with symbols representing the median per participant per timepoint. **(F to J)** Analysis of a subset of 8 of the 10 G001 samples from (A) to (E) using the G003 workflow, with results compared to those for G002 and published results for G001. In each row of the figure, the quantities analyzed in the panels on the right [(F) to (J)] match the quantities on the left [(A) to (E)]. Due to technical difficulties with BCR sequencing, VRC01-class responses were only recovered for 3 of 8 samples tested by the G003 workflow; hence panels (H) to (J) show data for only three samples. In (A) through (J), thick lines are medians, box plots show 25% and 75% quantiles, and whiskers approximate the 10% and 90% quantiles. Statistical analysis of the differences between workflows in this figure are provided in table S40. Source data can be found in Data S10.

**Fig. S20. Number of cells sorted per participant in G002 using eOD-GT8 probes. (A)** B cells. **(B)** IgG B cells. **(C)** eOD-GT8-specific (GT8^++^) IgG B cells. **(D)** eOD-GT8 CD4bs-specific (GT8^++^GT8-KO^-^) IgG B cells. Each symbol represents a single participant. Thick lines indicate median values, box plots show 25% and 75% quantiles, and whiskers approximate the 10% and 90% quantiles. Source data can be found in Data S10.


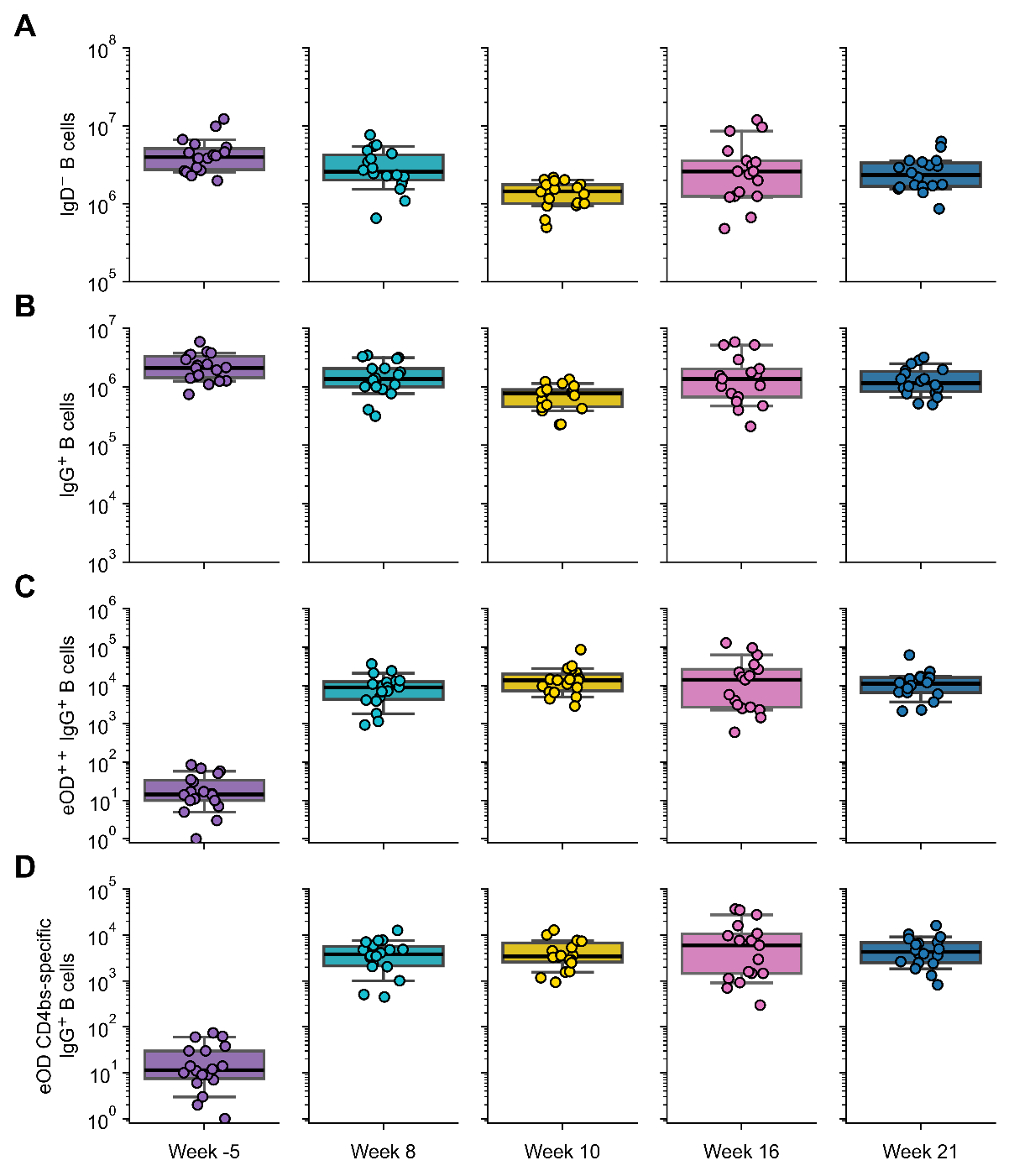


**Fig. S21. Number of cells sorted per participant in G003 using eOD-GT8 probes.** **(A)** IgD- B cells; **(B)** IgG+ B cells; **(C)** eOD-GT8-specific (GT8++) IgG+ B cells; **(D)** eOD-GT8 CD4bs-specific (GT8++GT8-KO-) IgG+ B cells. Each symbol represents a single participant. Thick lines indicate median values, box plots show 25% and 75% quantiles, and whiskers approximate the 10% and 90% quantiles. Source data can be found in Data S10.

**Fig. S22. Isotype and IgG subclass distributions in BCRs sorted by eOD-GT8 probes before and after eOD-GT8 60mer mRNA-LNP vaccination in G002. (A)** Isotype for all BCRs. **(B)** IgG subclass for all BCRs. **(C)** Isotype for VRC01-class BCRs. **(D)** IgG subclass for VRC01-class BCRs. Isotype and subclass were determined by sequence analysis of BCRs from IgD^-^ eOD-GT8 CD4bs-specific cells at postvaccination timepoints and both IgD^-^ and IgD^+^ types of eOD-GT8 CD4bs-specific cells at the prevaccination timepoint (fig. S13). eOD-GT8 60mer mRNA-LNP vaccinations occurred at weeks 0 and 8. Source data can be found in Data S10.

**
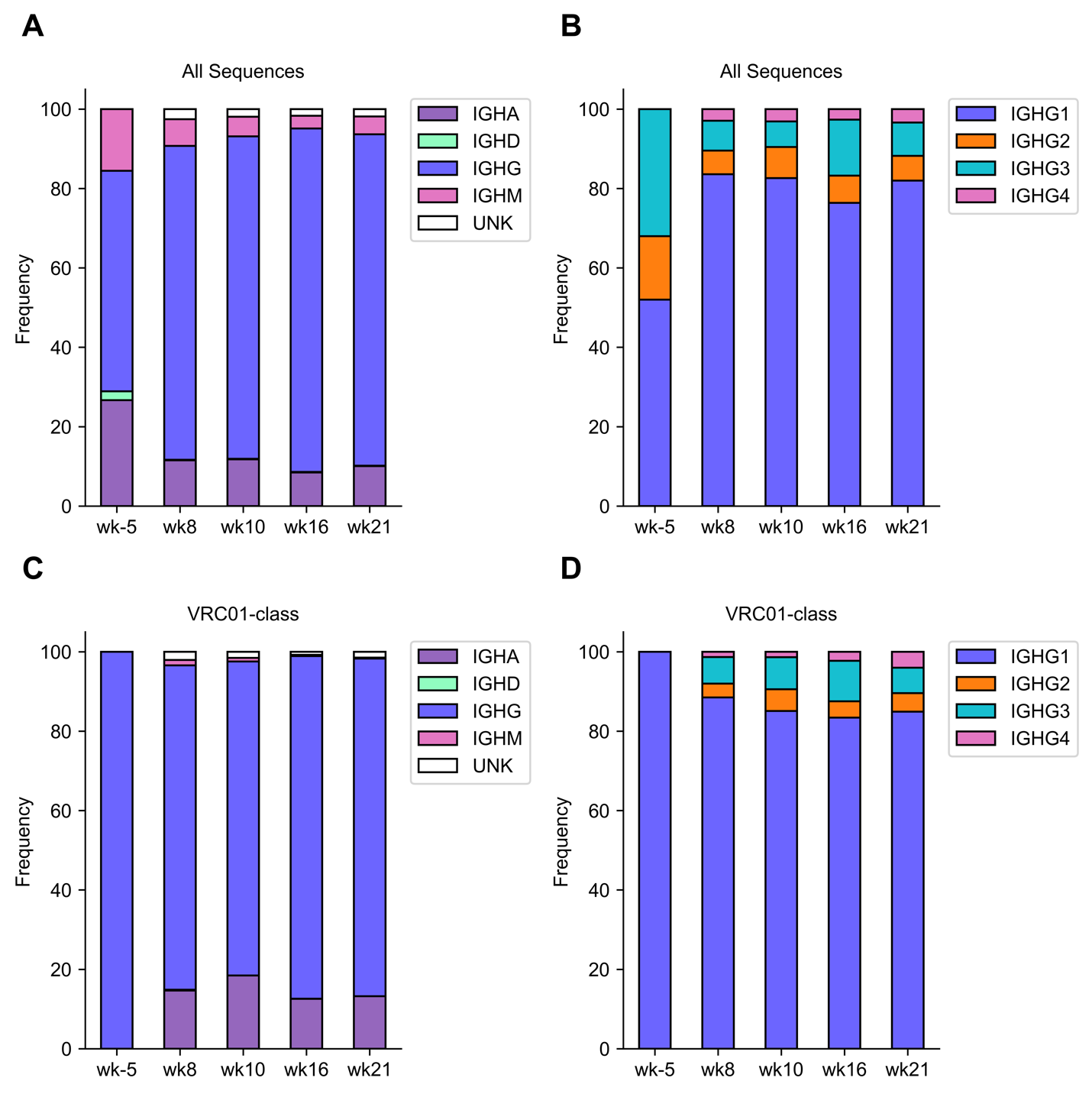
**

**Fig. S23. Isotype and IgG subclass distributions in BCRs sorted by eOD-GT8 probes before and after eOD-GT8 60mer mRNA vaccination in G003. (A)** Isotype for all BCRs; **(B)** IgG subclass for all BCRs; **(C)** Isotype for VRC01-class BCRs; **(D)** IgG subclass for VRC01-class BCRs. Isotype and subclass were determined by sequence analysis of BCRs from IgD^-^ eOD-GT8 CD4bs-specific cells at all timepoints (fig. S15). eOD-GT8 60mer mRNA-LNP vaccinations occurred at weeks 0 and 8. Source data can be found in Data S10.

**Fig. S24. VRC01-class response rates and VH1-2 genotypes for recipients of eOD-GT8 60mer mRNA-LNP in G002 and G003 or eOD-GT8 60mer protein and AS01_B_ in G001. (A)** Percentage of participants with a VRC01-class IgG B cell response (defined as having a VRC01-class IgG B cell frequency higher than at baseline), after one or two vaccinations. (B) Number of each VH1-2 genotype detected in G001 and G002, with VRC01-class response status for each genotype. In G001 and G002, all participants with non-permissive VH1-2 genotypes (05/05, 05/05, and 06/06) failed to produce a VRC01-class response (bottom three rows), and all participants with permissive VH1-2 genotypes (any genotype with at least one copy of 02 or 04) produced a VRC01-class response. In G003, we genotyped the one participant who did not produce VRC01-class responses and found that person to have a VH1-2 genotype of *06/*06. VH1-2 alleles *02 or *04 are utilized by VRC01-class antibodies, but alleles *05 and *06 are not (*15, 17*). Source data can be found in Data S10.

**Fig. S25.** Nucleotide percent mutation in heavy and light chain V genes for VRC01-class

BCRs isolated after eOD-GT8 60mer immunization from G001 (adjuvanted protein) and G002 and G003 (mRNA-LNP). (**A** and **B**) VRC01-class BCR V_H_ (A) and V_K/L_ (B) percentage nucleotide mutation, with symbols representing the median per participant per time point. Thick lines indicate median values, box plots show 25% and 75% quantiles, and whiskers approximate the 10% and 90% quantiles. (**C** and **D**) VRC01-class BCR V_H_ (C) and V_K/L_ (D) percentage nucleotide mutation, with violin plots representing the distribution of all BCRs per timepoint. Solid lines indicate median values, and dashed lines show 25% and 75% quantiles. Data from G001 are from both 100 µg and 20 µg dose groups combined. Comparisons over time within one trial were tested using Wilcoxon signed-rank test for paired data (table S56). Testing between G001 and G002 was done using the Wilcoxon rank-sum test for unpaired data (table S54). Note that these tests of differences in nucleotide mutation between G001 and G002 did not use the B cell workflow-adjusted bootstrap, whereas the analogous tests on amino acid mutation in Fig. 3 did use the bootstrap method (table S53). Significant differences had FDR Q-value ≤0.2 and P-values of <0.05 (*), <0.01 (**), <0.001 (***), or <0.0001 (****); ns indicated not significant. All B cells in this figure were sorted as eOD-GT8 CD4bs-specific. Source data can be found in Data S10.

**Fig. S26. Characteristics of post-eOD-GT8 60mer vaccination BCRs shared with VRC01-class bnAbs, for G001, G002, and G003. (A)** Percentage of BCRs using VRC01-class bnAb V_K/L_ genes, for VRC01-class and VH1-2-using non–VRC01-class BCRs, and for control VH1-2 BCRs from HIV-unexposed individuals from DeKosky et al. (*102*). VRC01-class bnAb V_K/L_ are indicated in the color key. **(B)** Percentage of BCRs with LCDR3 matching a VRC01-class bnAb sequence, for five–amino acid LCDR3s from VRC01-class BCRs, non–VRC01-class BCRs, and OAS control data LCs (*78, 79*), distinguishing IGK and IGL LCs. **(C)** Percentage of BCRs using Glu or Gln at LC position 96, for five–amino acid LCDR3s from VRC01-class BCRs, non–VRC01-class BCRs, and OAS control data LCs (*78, 79*). **(D)** Sequence logos for five–amino acid LCDR3s from VRC01-class BCRs for bnAbs (top row), G001 (second row), G002 (third row), G003 (fourth row), and human naïve precursors from prior studies (*8, 61, 103*) (bottom row), distinguishing kappa (left) and lambda (right) LCs. **(E)** Sequence logos for five–amino acid LCDR3s from non–VRC01-class BCRs from G001, G002, G003, and control data human LCs from HIV-unexposed individuals from the Observed Antibody Space (OAS) (*78, 79*), distinguishing kappa (left) and lambda (right) LCs. **(F)** Percentage of BCRs with Trp_103-5_, for VRC01-class and VH1-2-using non–VRC01-class BCRs and for OAS (*78, 79*) control data VH1-2 HCs. In (B), (C), and (F), symbols represent individual participants; thick lines indicate median values, boxes show 25 and 75% quantiles, and whiskers approximate 10 and 90% quantiles. The analysis includes all VRC01-class BCRs sorted as eOD-GT8 CD4bs-specific (GT8^++^GT8-KO^-^) at weeks 4, 8, 10, and 16 (G001), weeks 4, 8, 16, and 24 (G002), or at weeks 8, 10, 16, and 21 (G003) from all participants vaccinated with eOD-GT8 60mer adjuvanted protein (G001) or eOD-GT8 60mer mRNA-LNP (G002 and G003). G001 data includes both 20 µg and 100 µg dose groups. G002 data includes all participants in groups 1, 2, and 3 vaccinated once or twice with eOD-GT8 60mer mRNA-LNP but not after vaccination with core-g28v2 60mer. Source data can be found in Data S10.

**
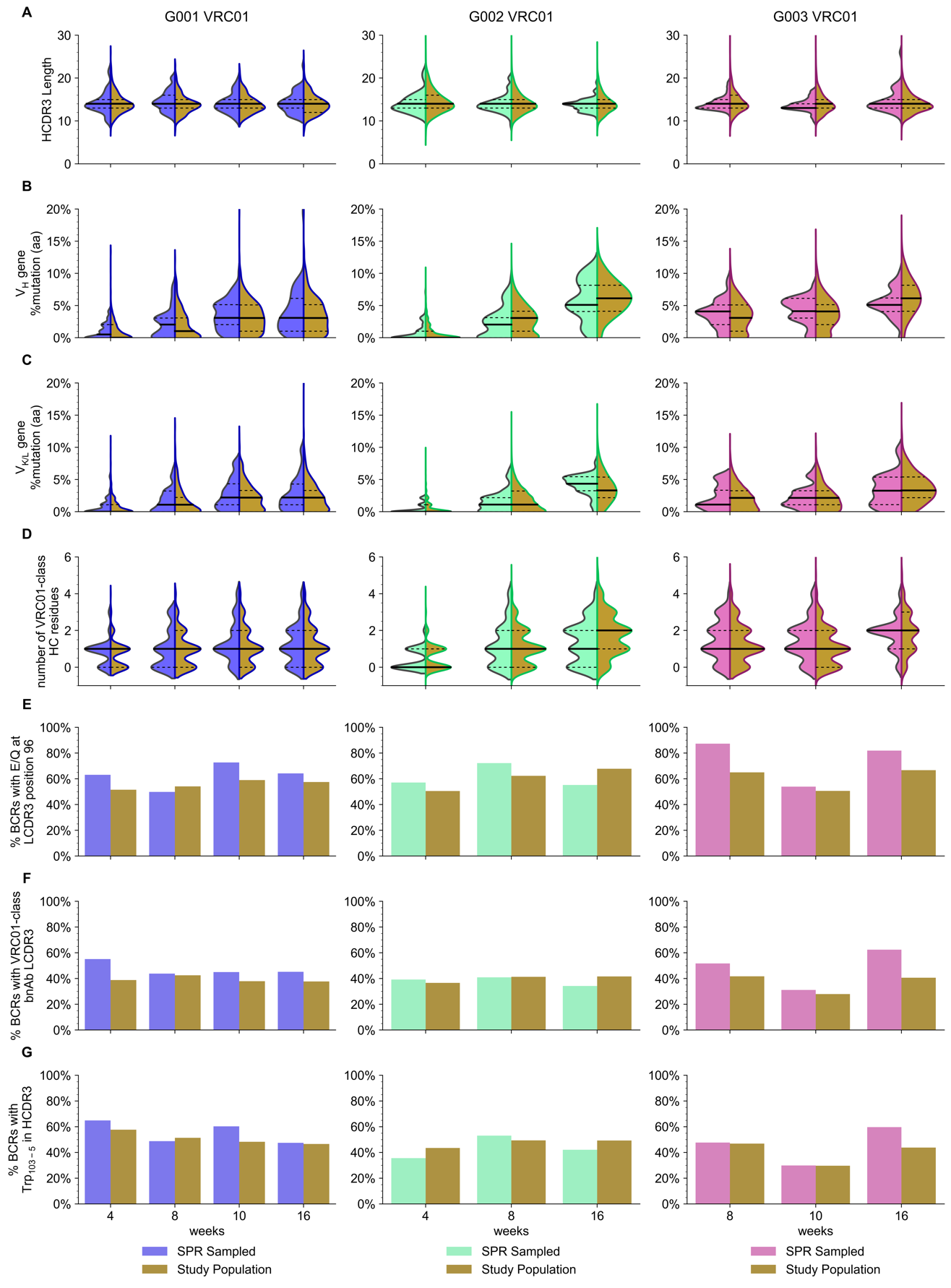
**

**Fig. S27. Comparisons of general genetic features and specific features shared with bnAbs, for VRC01-class mAbs tested by SPR compared to all VRC01-class BCRs isolated from the groups from which the mAbs were selected, for G001, G002, and G003.** Source data can be found in Data S10.

**
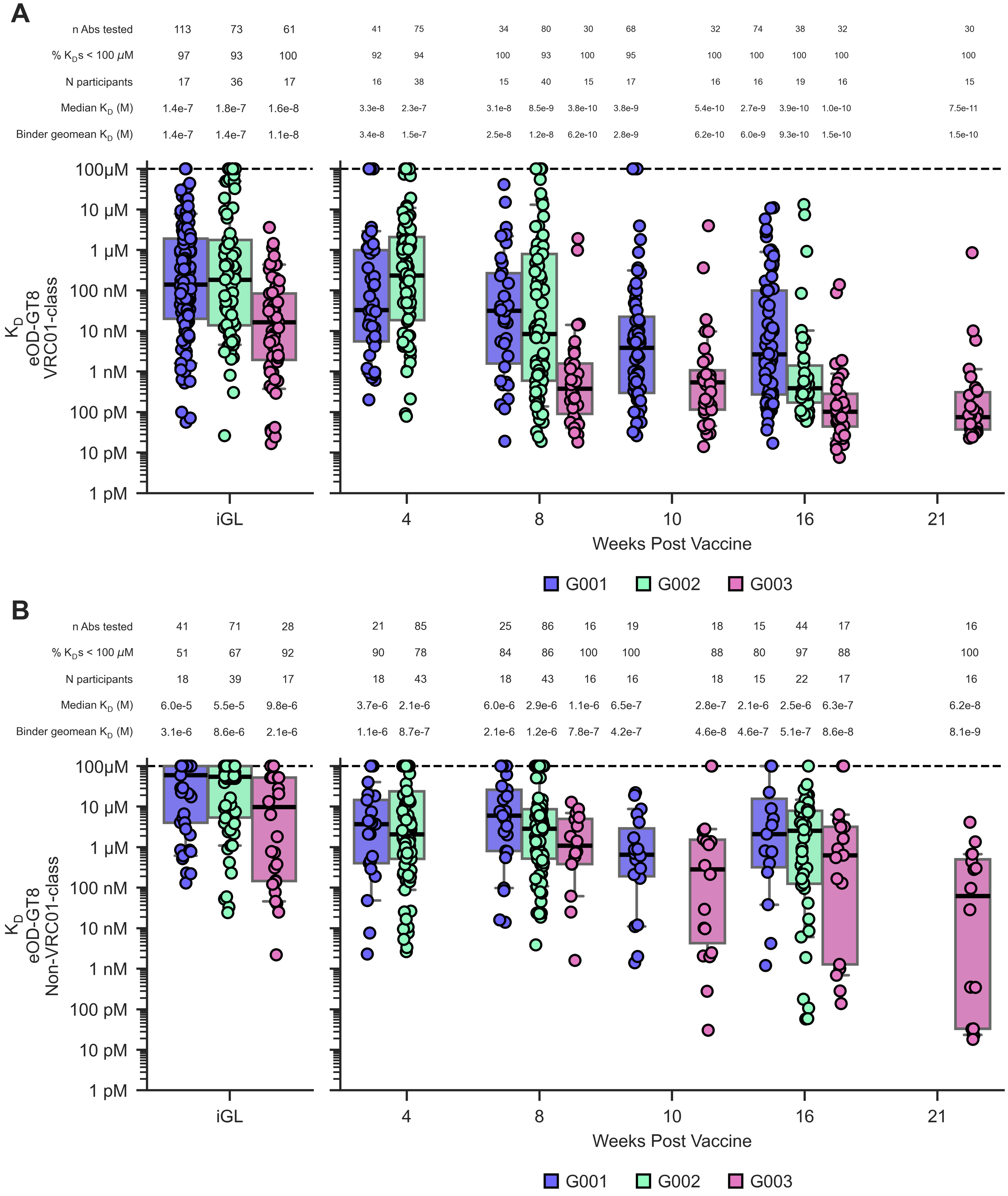
**

**Fig. S28. SPR-measured K_D_s for eOD-GT8 binding to VRC01-class and non-VRC01-class BCRs isolated post-eOD-GT8 60mer vaccination and their inferred germlines, for G001, G002, and G003. (A)** VRC01-class. **(B)** non-VRC01-class. All BCRs were sorted as eOD-GT8 CD4bs-specific and were selected randomly for expression and affinity testing for each participant and timepoint indicated. Antibodies from G002 are from participants in groups 1, 2, and 3 vaccinated once or twice with eOD-GT8 60mer mRNA-LNP who had not received a vaccination with core-g28v2 60mer at the time of sorting. Antibodies from G001 are from both low and high dose groups. The iGL antibodies represent inferred-germline variants of the post-vaccination antibodies shown. Source data can be found in Data S10.

**
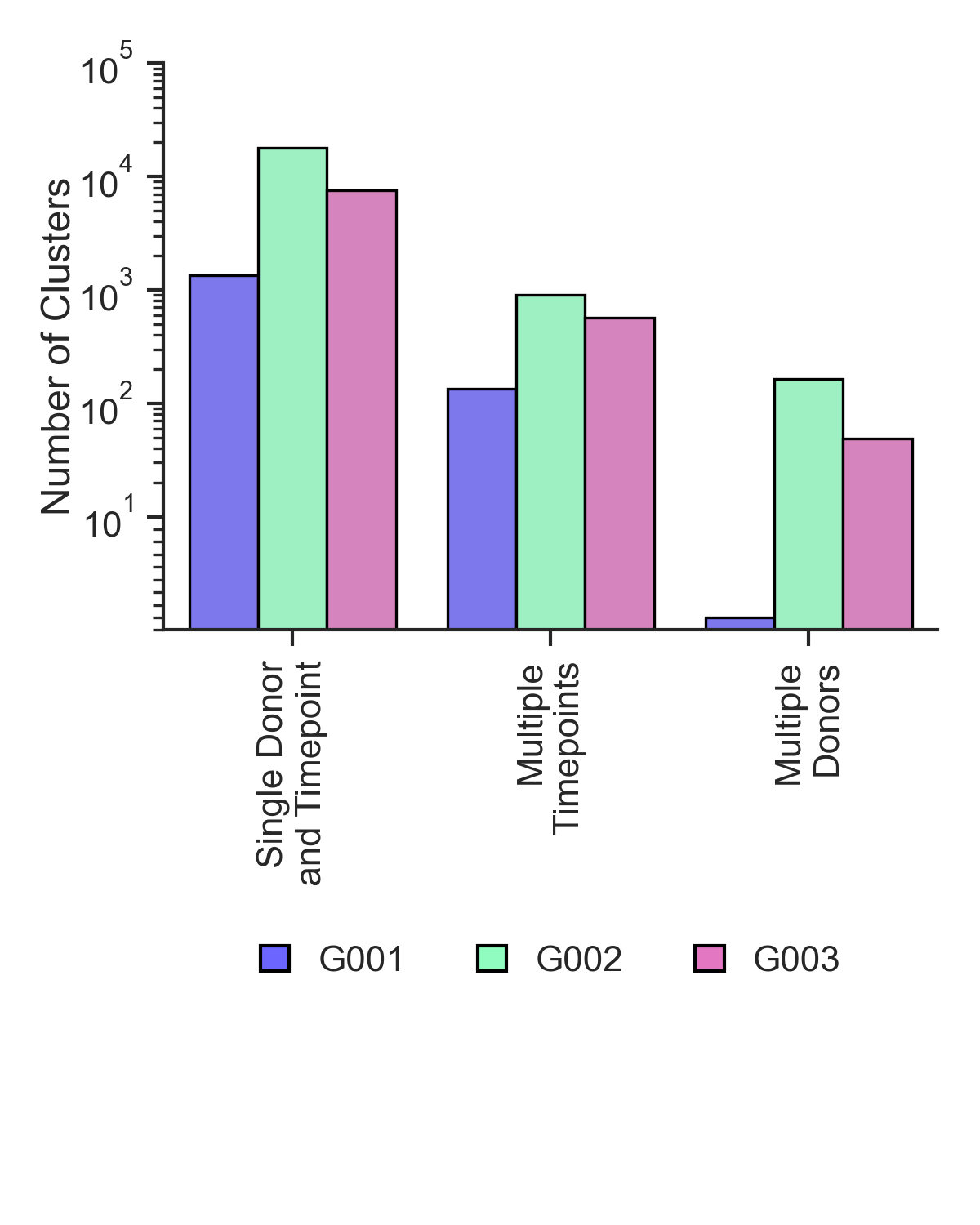
**

**Fig. S29. Number of BCR clusters (lineages) within one participant at one or multiple timepoints, or detected in more than one participant, for VRC01-class BCRs isolated after eOD-GT8 60mer vaccination by mRNA-LNP in G002 and G003 or by adjuvanted protein in G001.**  The analysis includes all VRC01-class BCRs sorted as eOD-GT8 CD4bs-specific (GT8^++^GT8-KO^-^) at weeks 4, 8, 10, and 16 (G001), weeks 4, 8, 16, and 24 (G002), or at weeks 8, 10, 16, and 21 (G003) from all participants vaccinated with eOD-GT8 60mer adjuvanted protein (G001) or eOD-GT8 60mer mRNA-LNP (G002 and G003). G001 data includes both 20 µg and 100 µg dose groups. G002 data includes all participants in groups 1, 2, and 3 vaccinated once or twice with eOD-GT8 60mer mRNA-LNP but not after vaccination with core-g28v2 60mer. BCR sequences from G001, G002, and G003 were clustered using the same method, but the method was different than previously described for G001 (*15*). Source data can be found in Data S10.

**
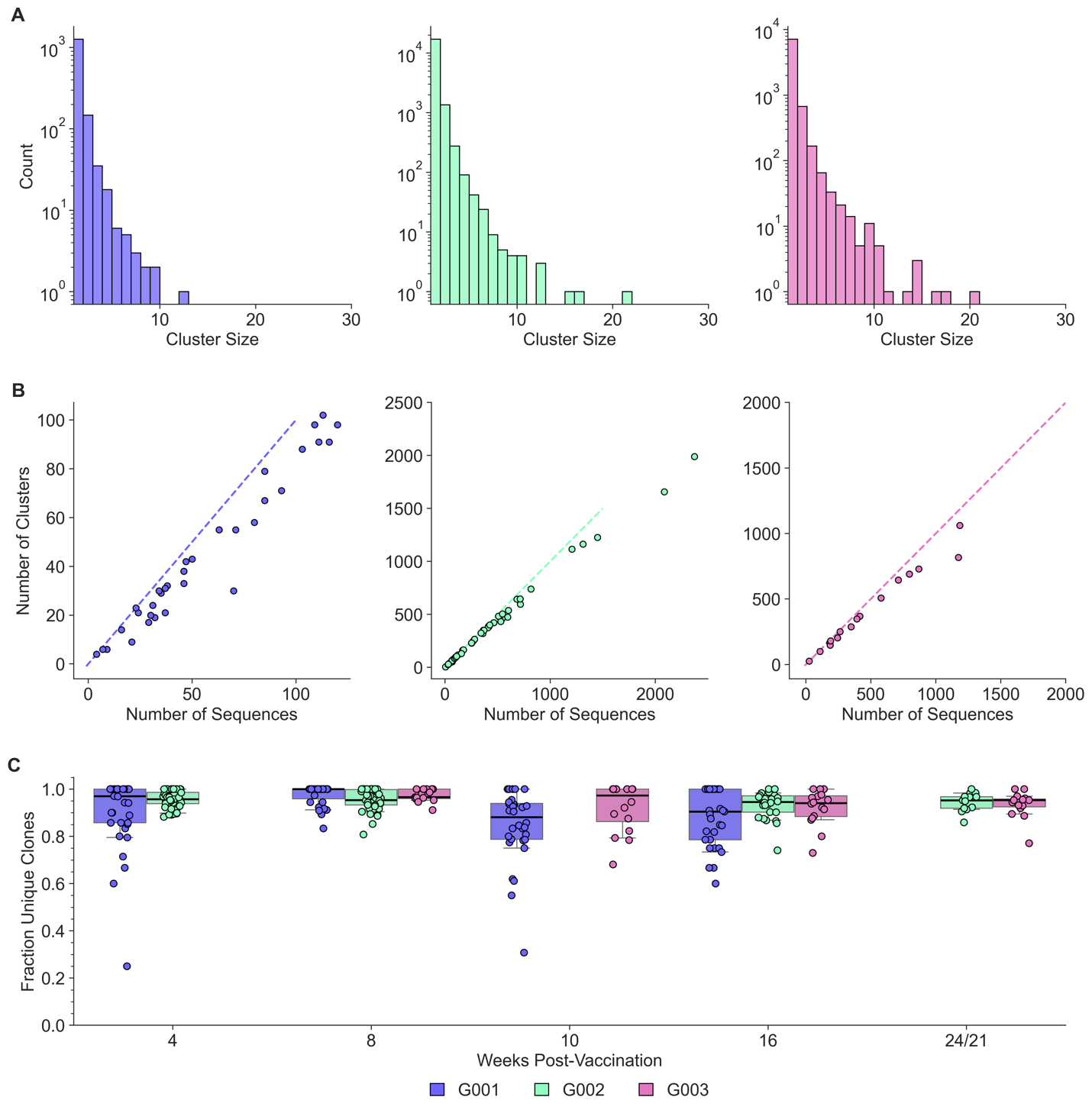
**

**Fig. S30. Hierarchical clustering and polyclonality for VRC01-class BCRs isolated after eOD-GT8 60mer vaccination, for G001, G002, and G003. (A)** Histogram of cluster size for G001 (left), G002 (middle), and G003 (right). **(B)** Number of clusters versus number of VRC01-class BCR sequences, for each participant, for G001 (left), G002 (middle), and G003 (right). **(C)** VRC01-class BCR polyclonality over time for G001 (blue), G002 (green), and G003 (salmon). Each symbol reports the fraction of BCR sequences that cluster as unique clones within a single donor at a single timepoint. Thick lines represent median values; boxes indicate 25% and 75% quantiles; whiskers approximate 10% and 90% quantiles. In (A) through (C), the analysis includes all VRC01-class BCRs sorted as eOD-GT8 CD4bs-specific (GT8^++^GT8-KO^-^) at weeks 4, 8, 10, and 16 (G001), weeks 4, 8, 16, and 24 (G002), or at weeks 8, 10, 16, and 21 (G003) from all participants vaccinated once (week 4 and 8 data) or twice (week 10, 16, 21, 24 data) with eOD-GT8 60mer adjuvanted protein (G001) or eOD-GT8 60mer mRNA-LNP (G002 and G003). G001 data includes both 20 µg and 100 µg dose groups. G002 data includes all participants in groups 1, 2, and 3 vaccinated once or twice with eOD-GT8 60mer mRNA-LNP but not after vaccination with core-g28v2 60mer. BCR sequences from G001, G002, and G003 were clustered using the same method, but the method was different than previously described for G001 (*15*). In (C), statistical testing between trials at each timepoint was done using the Wilcoxon rank-sum test. The only significant difference was G001 (mean=1.0; range=0.8333, 1.000; N=31) vs. G002 (mean=0.9529; range=08077,1.000; N=46) at week 8, with *P*=0.0036 and *Q*=0.0252. All B cells in this figure were sorted as eOD-GT8 CD4bs-specific. Source data can be found in Data S10.

**Fig. S31. Numbers of key cell populations sorted using core-g28v2 probes per participant in G002. (A)** Number of B cells. **(B)** Number of IgG B cells. **(C)** Number of core-g28v2-specific IgG B cells. **(D)** Number of core-g28v2 CD4bs-specific IgG B cells. Each symbol represents a participant at a given timepoint. Thick lines represent median values; boxes indicate 25% and 75% quantiles; whiskers approximate 10% and 90% quantiles. Source data can be found in Data S10.

**
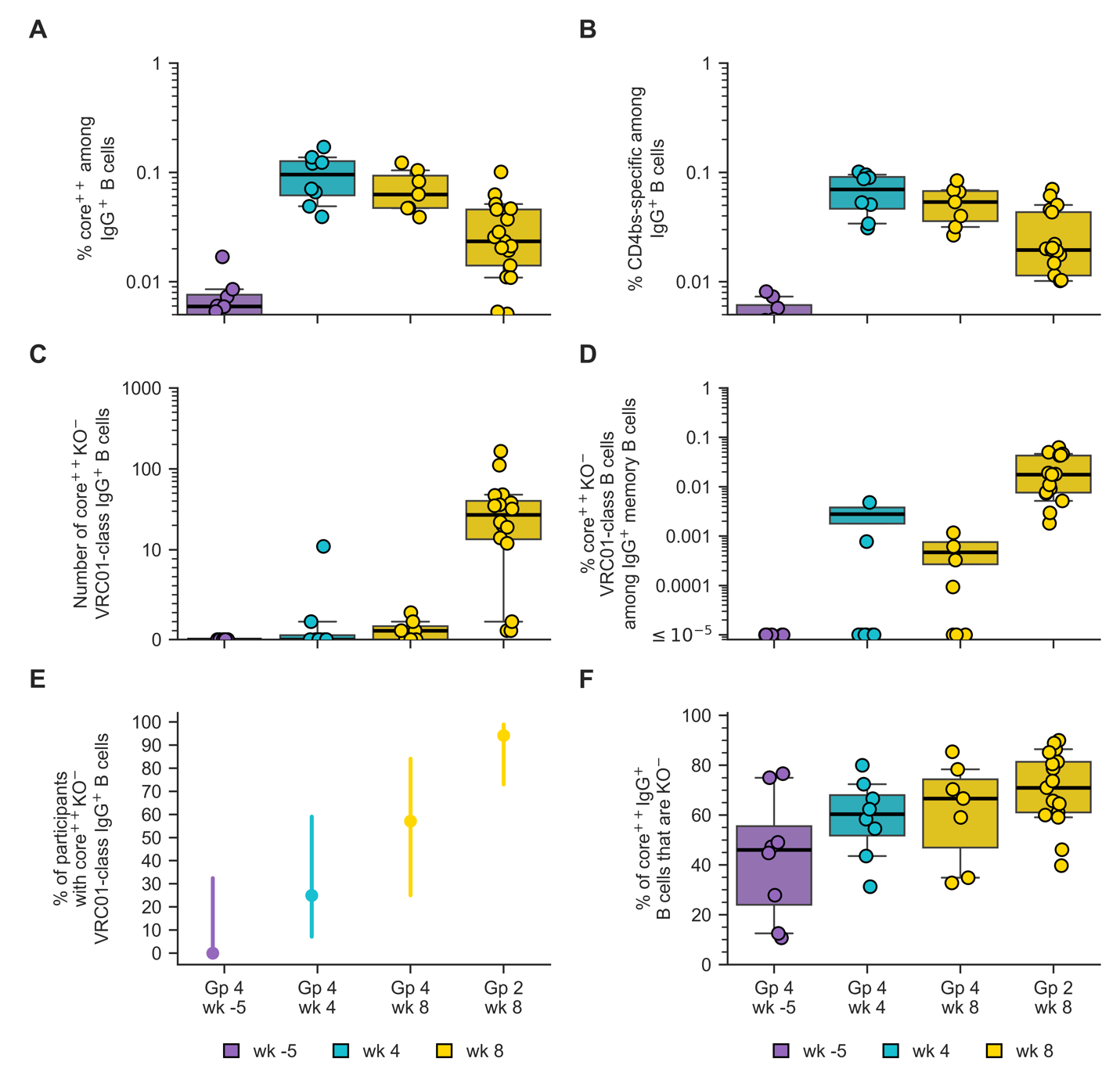
**

**Fig. S32. Frequency analysis of B cell responses in G002 group 4, with a comparison to week 8 for G002 group 2, with all samples sorted using core-g28v2 probes. (A and B)** Percentages of IgG memory B cells that are core-g28v2–specific (core^++^) (A) or core-g28v2 CD4bs-specific (B). **(C)** Number of core^++^KO^-^ VRC01-class IgG B cells detected. **(D)** Percentage of core^++^KO^-^ VRC01-class B cells among IgG memory B cells. **(E)** Percentage of participants with core^++^KO^-^ VRC01-class B cells detected at each timepoint. **(F)** Percentage of core^++^ IgG memory B cells that are CD4bs-specific (KO^-^). In (A) through (D) and (F), circles represent participants, thick lines indicate median values, and boxes indicate 25 and 75% quantiles. In (D), medians and quantiles were computed over nonzero values only because nonresponders are accounted for in (E). In (E), circles indicate median values, and lines indicate 95% CIs computed using the Wilson score method. Source data can be found in Data S10.


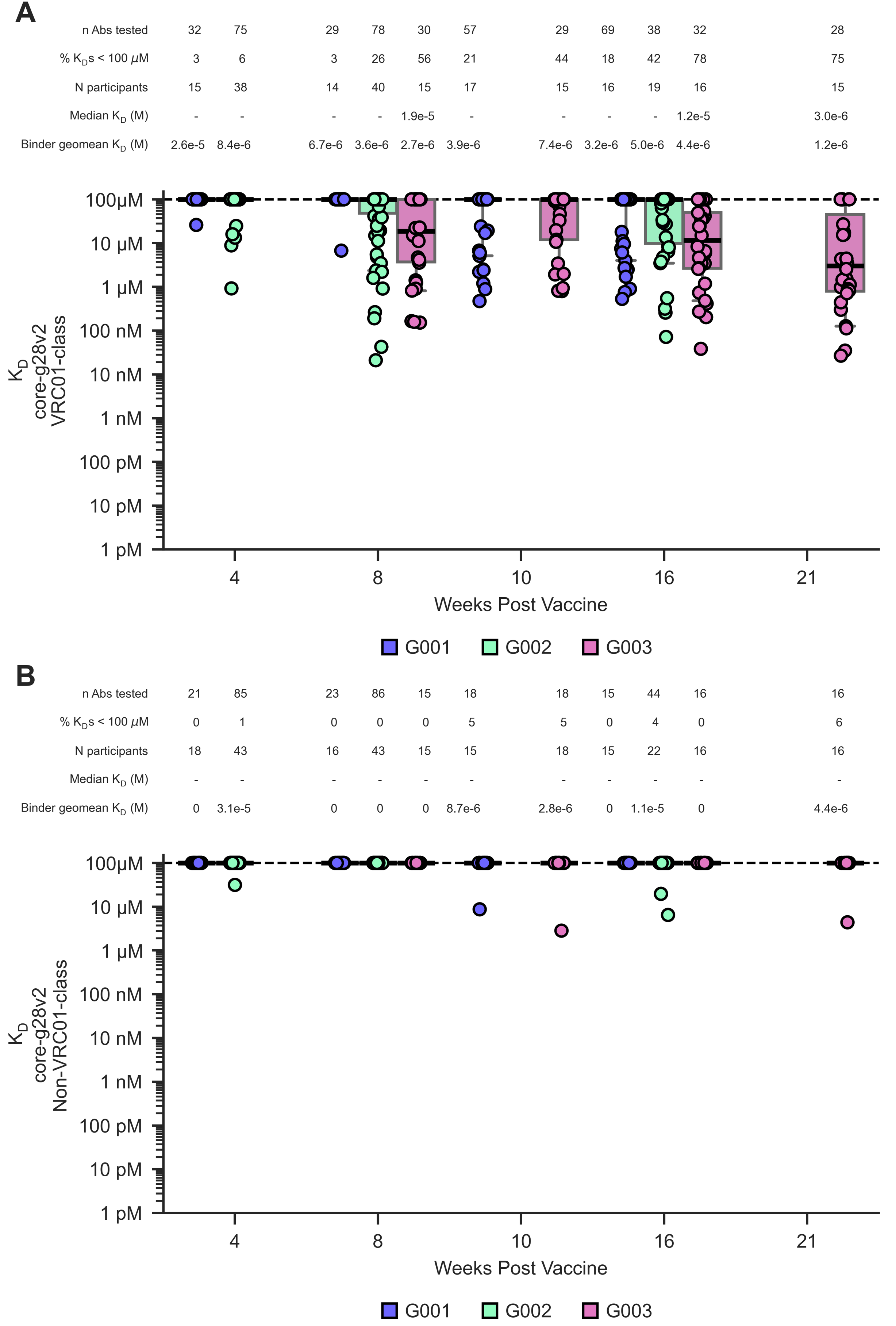


**Fig. S33. SPR-measured K_D_s for core-g28v2 binding to VRC01-class and non-VRC01-class BCRs isolated post-eOD-GT8 60mer vaccination, for G001, G002, and G003.** All BCRs were sorted as eOD-GT8 CD4bs-specific and selected randomly for expression and affinity testing for each participant and timepoint indicated. Antibodies from G002 are from participants in groups 1, 2, and 3 vaccinated once or twice with eOD-GT8 60mer mRNA-LNP who had not received a vaccination with core-g28v2 60mer at the time of sorting. Antibodies from G001 are from both low and high dose groups. Source data can be found in Data S10.

**Fig. S34. Ig isotype and IgG1 subclass distributions in VRC01-class BCRs isolated for the indicated prime-boost regimens and timepoints in G002. (A)** Isotype distribution. **(B)** IgG1 subclass distribution. All BCRs were sorted as core-g28v2 CD4bs-specific (core^++^core-KO^-^). Isotype and subclass were determined by sequence analysis. Source data can be found in Data S10.

**Fig. S35. VRC01-class BCR polyclonality for prime-boost regimens. (A)** Fraction unique clones, also referred to as polyclonality. Each symbol reports the fraction of core^++^KO^-^ VRC01-class IgG BCR sequences that cluster as unique clones within a single donor at a single timepoint. **(B)** Total number of unique clones. Each symbol reports the total number of core^++^KO^-^ VRC01-class IgG BCR sequences that cluster as unique clones within a single donor at a single timepoint. Thick lines represent median values; boxes indicate 25% and 75% quantiles; whiskers approximate 10% and 90% quantiles. Testing between groups at matched timepoints was done using the Wilcoxon rank-sum test for unpaired data or a signed rank test for paired data (table S59). Testing between groups at different timepoints was done using the Wilcoxon rank-sum test (table S60). Significant differences had FDR Q-value ≤0.2 and P-values of <0.05 (*), <0.01 (**), <0.001 (***), or <0.0001 (****); ns indicated not significant. All B cells in this figure were sorted as core-g28v2 CD4bs-specific. Source data can be found in Data S10.

**Fig. S36. Histograms of clone size by group in G002.** (A) Group 1 (eOD®eOD; 10,196 total clones) (B) Group 1 (eOD®core; 8,905 total clones) (C) Group 3 (eOD®eOD®core; 9,438 total clones) (D) Group 4 (core; 10 total clones). Clustering included all VRC01-class IgG BCRs in G002 whether sorted as eOD-GT8 CD4bs-specific or core-g28v2 CD4bs-specific. Histograms show percentages of the total number of clones per group, and the total numbers of clones are provided for each group. Histograms had bin size of 1; all bins are shown.

**Fig. S37. Properties of the two largest VRC01-class memory B cell lineages detected for the eOD→core regimen. (A and B)** Maximum likelihood-based BCR lineage trees for (A) G002-145-clone99 and (B) G002-577-clone46. Tips are labeled with the sorting probe used to isolate the memory B cells and color-coded based on timepoint. Branch lengths correspond to nucleotide substitutions per site. Monovalent *K*_D_ values for binding to core-g28v2 monomer for representative members of the lineage trees are also included in tip labels. **(C and D)** Divergence versus time plots and B cell evolution over time correlation analysis for (C) G002-145-clone99 and (D) G002-577-clone46. Linear regression was performed in Prism (slope), and correlation analysis (correlation and p value) was performed in Dowser (*76*). **(E and F)** Affinity versus time plots for binding to core-g28v2 monomer for (E) G002-145-clone99 and (F) G002-577-clone46. Linear regression was performed in Prism.

**Fig. S38. Properties of the largest VRC01-class memory B cell lineages detected for the eOD→eOD regimen. (A)** Maximum likelihood-based BCR lineage tree for G002-852-clone8. As in fig. S37, tips are labeled with the sorting probe used to isolate the memory B cells and color-coded based on timepoint. Branch lengths correspond to nucleotide substitutions per site. Monovalent *K*_D_ values for binding to core-g28v2 monomer for representative members of the lineage trees are also included in tip labels. **(B)** Divergence versus time plots and B cell evolution over time correlation analysis. As in fig. S37, linear regression was performed in Prism (slope), and correlation analysis (correlation and p value) was performed in Dowser (*76*). **(C)** Affinity versus time plot for binding to core-g28v2 monomer for G002-852-clone83.

**Fig. S39. Key VRC01-class heavy chain residues documented for VRC01-class antibodies in G002.** Each row corresponds to a VRC01-class BCR. However, it was not practical to display all VRC01-class BCRs, therefore we used downsampling to select random sets of BCRs to display for each group and timepoint. Dark blue indicates the presence of a key VRC01-class HC residue, whereas light blue indicates the absence of a key VRC01-class HC residue. Positions of key VRC01-class HC residues (*15, 26*) are indicated at the bottom. Trp_103-5_ is included in the key VRC01-class HC residues but is not shown here.

**Fig. S40. BnAb properties of core^++^KO^-^** **VRC01-class IgG BCRs induced by prime-boost regimens in G002.** **(A)** Percentage of BCRs using VRC01-class bnAb V_K/L_ genes, for VRC01-class BCRs from the indicated regimens, and for control VH1-2 BCRs from HIV-unexposed individuals from DeKosky et al. (*102*). VRC01-class bnAb V_K/L_ are indicated in the color key. **(B)** Percentage of VRC01-class BCRs with LCDR3 matching a VRC01-class bnAb sequence, distinguishing kappa and lambda BCRs. **(C)** Percentage of BCRs using Glu or Gln at LC position 96, for five–amino acid LCDR3s from VRC01-class BCRs and OAS control data LCs (*78, 79*). **(D)** Sequence logos for five–amino acid LCDR3s from VRC01-class BCRs for bnAbs (top row), each G002 prime-boost pseudogroup (rows 2 to 8), and human naïve precursors from prior studies (*8, 61, 103*) (bottom row), distinguishing kappa (left) and lambda (right) LCs. **(E)** Percentage of BCRs with Trp_103-5_, for VRC01-class BCRs and for OAS (*78, 79*) control data VH1-2 HCs. In (B), (C), and (E), symbols represent individual participants; thick lines indicate median values, boxes show 25 and 75% quantiles, and whiskers approximate 10 and 90% quantiles. Statistical testing between groups in (B), (C), and (E) was done using the Wilcoxon rank-sum test (table S62). Significant differences had FDR Q-value ≤0.2 and P-values of <0.05 (*), <0.01 (**), <0.001 (***), or <0.0001 (****). Non-significant differences are shown in table S62 but not indicated in the figure. All B cells in this figure were sorted as core-g28v2 CD4bs-specific. Source data can be found in Data S10.

**Fig. S41. SHM and key VRC01-class residues in "random" and "selected" VRC01-class mAbs elicited by the indicated prime-boost regimens in G002. (A** and **B)** VRC01-class BCR V_H_ (A) and V_K/L_ (B) percent amino acid (aa) mutation, with symbols representing individual mAbs. **(C** and **D)** Number of key VRC01-class HC (C) and HCDR2 (D) residues, with symbols representing individual mAbs. In (A) to (D), thick lines indicate median values, box plots show 25% and 75% quantiles, and whiskers approximate 10% and 90% quantiles, for the indicated populations of mAbs. All mAbs in this figure correspond to B cells sorted as core-g28v2 CD4bs-specific (core^++^KO^-^). Source data can be found in Data S10.

**Fig. S42. Properties of "random" and "selected" VRC01-class mAbs elicited by the indicated prime-boost regimens in G002, that are shared with VRC01-class bnAbs. (A)** Percentage of BCRs using VRC01-class bnAb V_K/L_ genes, for "random" (left) "selected" (middle) mAbs, and for control VH1-2 BCRs from HIV-unexposed individuals from DeKosky et al. (*102*). VRC01-class bnAb V_K/L_ are indicated in the color key. **(B)** Percentage of mAbs with LCDR3 matching a VRC01-class bnAb sequence. **(C)** Percentage of mAbs using Glu or Gln at LC position 96. **(D)** Percentage of mAbs with Trp_105-3_. Bars indicate the percentage of mAbs having the property of interest among the "random" or "selected" mAbs elicited by the indicated prime-boost regimen. All mAbs in this figure correspond to B cells sorted as core-g28v2 CD4bs-specific (core^++^KO^-^). Source data can be found in Data S10.

**Fig. S43. On- and off-rates for monovalent interactions between core-g28v2 and VRC01-class BCRs isolated after different prime-boost regimens, corresponding to *K*_D_s in Fig. 6A.** *k*_on_ (A) and *k*_off_ (B) values for "random" and "selected" antibody ligands binding to core-g28v2 monomer analyte. Thick lines indicate median values, boxes show 25% and 75% quantiles, and whiskers approximate 10% and 90% quantiles. Data are shown for all VRC01-class BCRs in Fig. 6A for which core-g28v2 *K*_D_s of <100 μM were obtained by kinetic fits. All mAbs in this figure correspond to B cells sorted as core-g28v2 CD4bs-specific. Source data can be found in Data S10.

**Fig. S44. Key VRC01-class light chain residues.** Violin plots show the distribution of the number of key VRC01-class light chain residues in VRC01-class BCRs sorted by core-g28v2 probes with light chain V genes of V_K_1-33 (A), V_K_3-20 (B), V_K_1-5 (C), V_K_3-15 (D). Numbers of key VRC01-class light chain residues in VRC01-class bnAbs with the indicated light chain V genes are indicated in black circles. Dashed lines represent the mean number of key VRC01-class light chain residues in VRC01-class bnAbs with the indicated V genes.


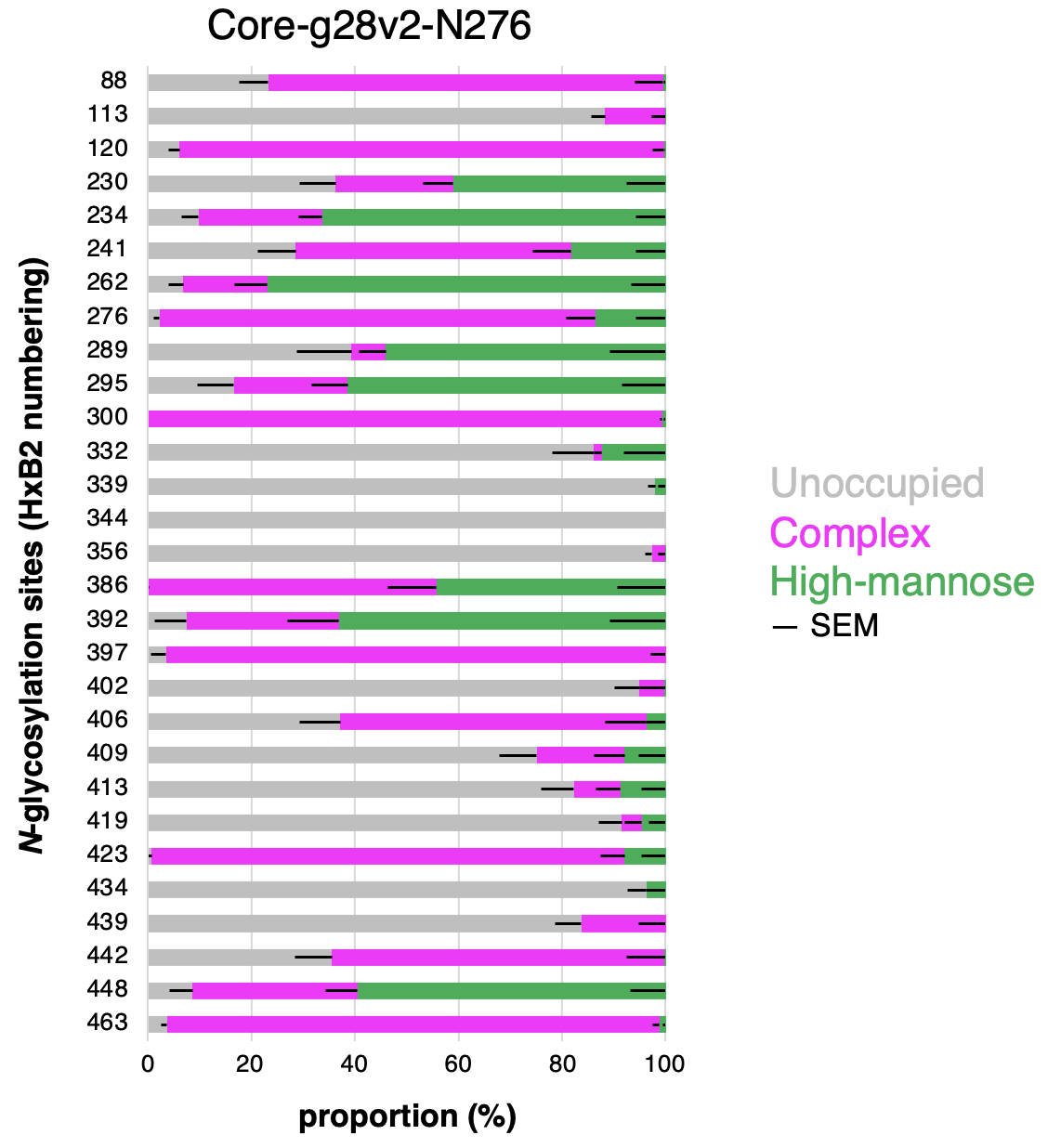


**Fig. S45.** **N-linked glycan occupancy and glycoform for core-N276, the core-g28v2 variant containing the N276 glycosylation site.** The N276 glycan was 97.7% occupied. Glycan analyses were performed as described in Baboo et al. 2021 (*80*).

**Fig. 46. On- and off-rates for monovalent interactions between core-N276 and VRC01-class BCRs isolated after different prime-boost regimens in G002, corresponding to *K*_D_s in Fig. 6B.** *k*_on_ (A) and *k*_off_ (B) values for "random" and "selected" antibody ligands binding to core-N276 monomer analyte. Thick lines indicate median values, boxes show 25% and 75% quantiles, and whiskers approximate 10% and 90% quantiles. Data are shown for all VRC01-class BCRs in Fig. 6B for which core-N276 *K*_D_s of <100 μM were obtained by kinetic fits. All mAbs in this figure correspond to B cells sorted as core-g28v2 CD4bs-specific. Source data can be found in Data S10.

**
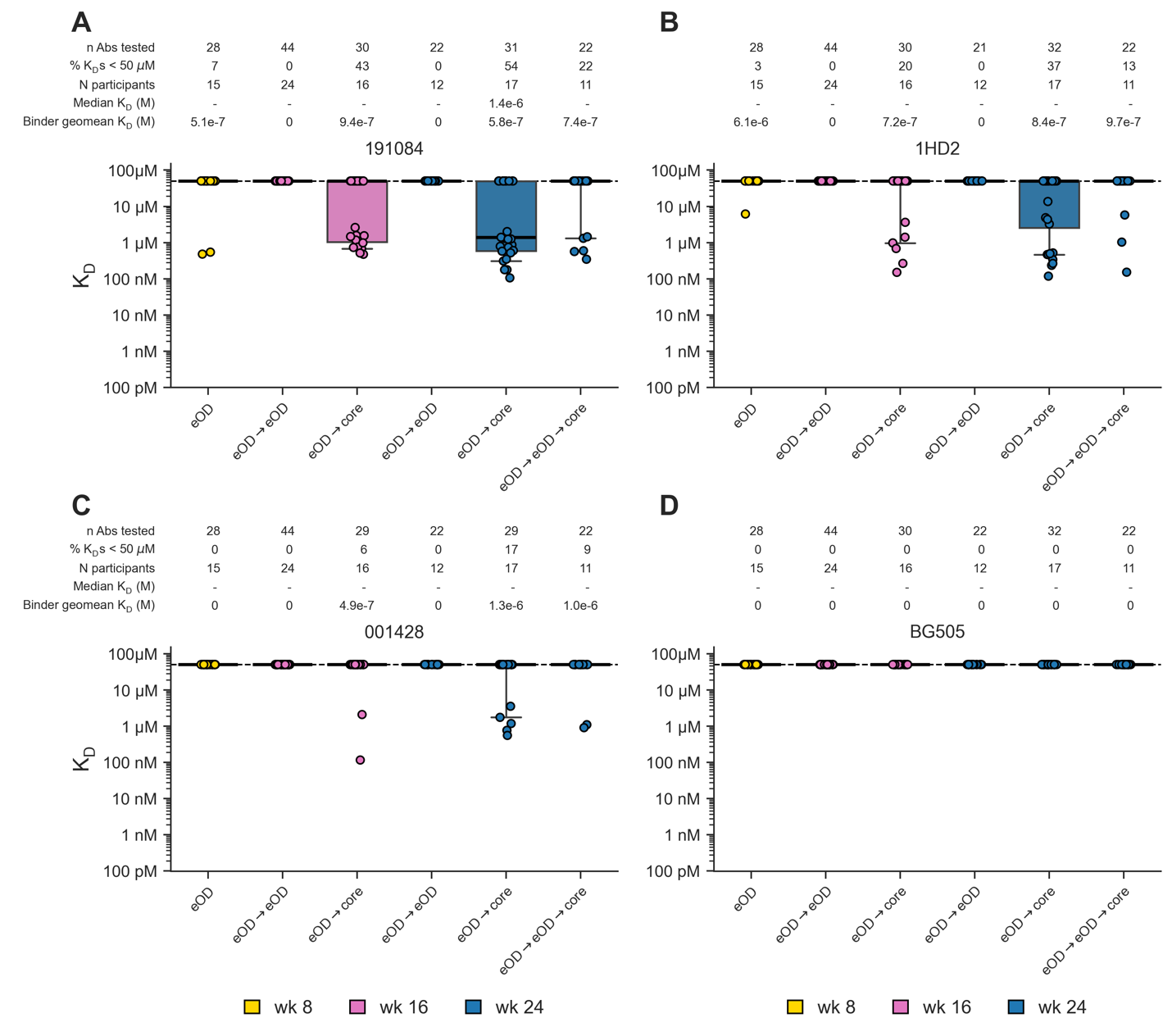
**

**Fig. S47.** **SPR analysis of VRC01-class BCR affinities for wild-type HIV Env trimers of different isolates containing the N276 glycan.** Apparent K_D_ values for "selected" antibody ligands binding to stabilized trimer analytes containing the N276 glycan from the clade A isolate 191084 (A), the clade B isolate 1HD2 (B), clade C isolate 001428 (C), and the clade A isolate BG505 the (D). Low density IgG ligand capture was used to ensure that K_D_s approximate monovalent interactions to the greatest degree possible. Thick lines are medians; boxes show 25% and 75% quantiles; whiskers approximate 10% and 90% quantiles. For median *K*_D_, "-" indicates median ≥ 50 µM. For binder geomean *K*_D_, "-" indicates the geomean was undefined because there were no binders with *K*_D_<50 µM. The K_D_s in this figure were generally measured once. All mAbs in this figure correspond to B cells sorted as core-g28v2 CD4bs-specific (core^++^KO^-^). Testing between populations of binders (*K*_D_<50μM) in different groups was done using GEE, but very few comparisons could be made due to the low number of binders per group. The only significant difference identified was in (A) for eOD®core comparing week 16 to week 24, with geomean ratio of 0.6203, P value of 0.0187, and FDR Q value of 0.0562. Source data can be found in Data S10.

**Fig. S48.** **SPR analysis of VRC01-class BCR affinities for HIV Env trimers of different isolates modified to lack the N276 glycan. (A** to **D)** Apparent *K*_D_ values for "selected" antibody ligands binding to stabilized trimer analytes lacking the N276 glycan from the 191084 clade A isolate (191084-N276D) (A), the clade B isolate 1HD2 (1HD2-N276Q) (B), the clade C isolate 001428 (001428-T278M) (C), and the clade A isolate BG505 (BG505-N276Q) (D). Low density IgG ligand capture was used to ensure that *K*_D_s approximate monovalent interactions to the greatest degree possible. Thick lines indicate median values, boxes show 25% and 75% quantiles, and whiskers approximate 10% and 90% quantiles. For median *K*_D_, "-" indicates median ≥ 50 µM. For binder geomean *K*_D_, "-" indicates the geomean was undefined because there were no binders with *K*_D_<50 µM. Testing between populations of binders (*K*_D_<50μM) in different groups was done using GEE (table S64). Significant differences had FDR Q-value ≤0.2 and P-values of <0.05 (*), <0.01 (**), <0.001 (***), or <0.0001 (****); ns indicated not significant. Comparisons resulting in NA did not have any values <50μM in one of the comparator groups. Most *K*_D_ values in this figure were measured once; a subset was measured twice. All mAbs in this figure correspond to B cells sorted as core-g28v2 CD4bs-specific. Source data can be found in Data S10.

**Fig. S49. Sequence alignment for stabilized trimers with mutations to remove the N276 glycan employed for SPR studies in fig. S48.** The sequences are aligned to the HxB2 Env sequence, and the HxB2 residue numbering is indicated.

**Fig. S50. Antigenic properties of N276-lacking HIV Env trimers used for SPR studies in fig. S48.** Biolayer interferometry (BLI) was used to assess antigenic profiles for the indicated trimers binding to IgGs for bnAbs (quaternary, PGT151 and PGT145; CD4bs, VRC01; and V3-glycan, PGT121 and PGT128) and non-nAbs (V3, 19B and 3074; CD4bs, B6 and F105). The trimers 191084 N276D, BG505 N276Q, 1HD2 N276Q, and 001428 T278M were the same trimers tested by SPR in fig. S48; their amino acid sequences are given in fig. S49. BG505 MD39 congly was a positive control stabilized trimer (*9*) with two additional glycosylation sites to fill the "glycan hole" in the BG505 trimer (*104*). The 1HD2 isolate is not neutralized by PGT145, and hence PGT145 is not expected to bind well to 1HD2 stabilized trimers.

**Fig. S51. Negative stain EM assessment of N276-lacking HIV Env trimers used for SPR studies in fig. S48.** Negative stain 2D class averages are shown for (A) 191084-N276D; (B) 1HD2-N276Q; (C) 001428-T287M; (D) BG505-N276Q. The percentage native-like trimer in the sample and the number of particles analyzed are shown in each panel. The amino acid sequences of the trimers are given in fig. S48.

**Fig. S52.** **SPR analysis of binding of non-VRC01-class, core^++^KO^-^ BCRs to HIV Env trimers of different isolates modified to lack the N276 glycan.** Apparent K_D_ values for non-VRC01-class antibody ligands binding to stabilized trimer analytes lacking the N276 glycan from the 191084 clade A isolate (191084-N276D) (A), the clade B isolate 1HD2 (1HD2-N276Q) (B), the clade C isolate 001428 (001428-T278M) (C), and the clade A isolate BG505 (BG505-N276Q) (D). The same trimers were tested against VRC01-class mAbs in fig. S48. Low density IgG ligand capture was used to ensure that K_D_s approximate monovalent interactions to the greatest degree possible. Thick lines indicate median values, boxes show 25% and 75% quantiles, and whiskers approximate 10% and 90% quantiles. For median K_D_, "-" indicates median ≥ 100 µM. The K_D_s in this figure were generally measured once. All mAbs in this figure correspond to B cells sorted as core-g28v2 CD4bs-specific (core^++^KO^-^). Source data can be found in Data S10.

**Fig. S53. Neutralization IC_50_ versus SPR *K*_D_ for "selected" VRC01-class mAbs from group 2 and the indicated pseudoviruses and matched soluble trimers.** The limit of detection for the neutralization assay was 50 µg/ml, as indicated by the dotted horizontal line. Fitting to a two-segment linear function was done on log-transformed IC_50_ and *K*_D_ data. Solid lines show the fit, and dashed lines show the 95% confidence range for the fit. X0 corresponds to the fit value for the maximum *K*_D_ for neutralization IC_50_<50 µg/ml. The median *K*_D_ threshold for detectable neutralization across all 6 pseudoviruses was 329 nM. Source data can be found in Data S10.

**Fig. S54. Neutralization IC_50_ versus SPR off-rate (*k*_off_) for "selected" VRC01-class mAbs from group 2 and the indicated pseudoviruses and matched soluble trimers.** The limit of detection for the neutralization assay was 50 µg/ml, as indicated by the dotted horizontal line. Fitting to a two-segment linear function was done on log-transformed IC_50_ and *k*_off_ data. Solid lines show the fit, and dashed lines show the 95% confidence range for the fit. X0 corresponds to the fit value for the maximum *k*_off_ for neutralization IC_50_<50 µg/ml. The median *k*_off_ threshold for detectable neutralization across all 6 pseudoviruses was 0.001935 s^-1^. Source data can be found in Data S10.

**Fig. S55. Neutralization IC_50_ versus SPR on-rate (*k*_on_) for "selected" VRC01-class mAbs from group 2 and the indicated pseudoviruses and matched soluble trimers.** The limit of detection for the neutralization assay was 50 µg/ml, as indicated by the dotted horizontal line. Fitting was attempted as in figs. S53 and S54, but none of the fits converged. Source data can be found in Data S10.

**Fig. S56. Sequence alignment for N276+ gp120 cores tested by SPR in Fig. 8.**


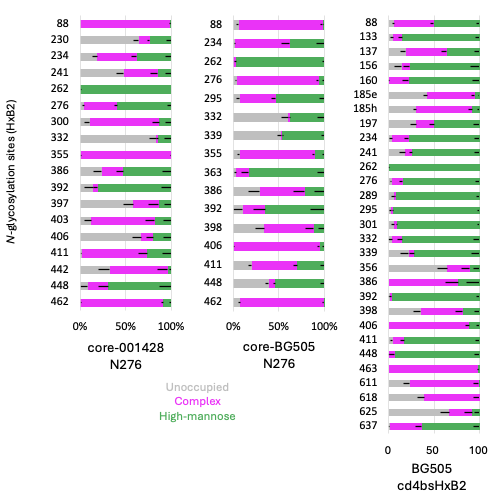


**Fig. S57. N-linked glycan occupancy and glycoform analysis of heterologous cores and BG505 cd4bsHxB2 trimer used for SPR analysis in Fig. 8.** The N276 glycan was >95% occupied in all three constructs. Glycan analyses were performed as described in Baboo et al. 2021 (*80*).

**Fig. S58. Amino acid sequence alignment for N276-lacking trimer next-boost candidates to follow core-g28v2 60mer, used for SPR and structural studies in** **Fig. 8.**

**Fig. S59. Antigenic profiles for additional N276-lacking trimer next-boost candidates to follow core-g28v2 60mer, used for SPR and structural studies in** **Fig. 8.** BLI was used to assess antigenic profiles for the indicated trimers binding to IgGs for bnAbs (quaternary, PGT151 and PGT145; CD4bs, VRC01; and V3-glycan, PGT121 and PGT128) and non-nAbs (V3, 19B and 3074; CD4bs, B6 and F105). Antigenic profiles for other trimers tested in Fig. 8 are shown in fig. S50.

**Fig. S60. Serum IgG antibody binding magnitudes to HIV Env trimers lacking the N276 glycan and controls. (A)** Response magnitudes (AUTC) are shown for serum binding response from Group 2 weeks 0, 16 and 24 to BG505 cd4bsHxB2-T278M and three next-stage booster candidates (001428-T278M, CNE40-T278M, and V703-0537-T278M). (B) Response magnitudes (AUTC) are shown for serum binding response from Group 2 weeks 0, 8, and 16 to controls eOD-GT8 60mer, eOD-GT8, eOD-GT8 KO11, core-g28v2 60mer, core-g28v2, and core-g28v2 KO11b.

**Fig. S61. CryoEM reconstruction statistics.** Representative 2D class averages, Fourier shell correlation, EM density map, local resolution estimate, angular distribution of observations, and map to atomic model Fourier shell correlation for **(A)** G002-293-0536 Fab in complex with 001428_T278M_L14 SOSIP and RM20A3 Fab and **(B)** G002-480-0546 Fab in complex with V703-0537_T278M_L14 SOSIP and BG18 Fab.

**Fig. S62. Sequence comparison of mAbs 293-0563 and 480-0546 to VRC01.** Analyses of heavy and light chain amino acid sequences of 293-0563 and 480-0546. Framework regions (FRs) and CDRs were determined using Kabat’s definition (*105*). Residue numbering based on the Kabat numbering scheme (*106*). Key VRC01-class germline residues highlighted in blue, key VRC01-class mutations highlighted in pink, and LCDR3 residues matching VRC01-class bnAbs highlighted in blue.

**Fig. S63. Structural comparison of Fabs 293-0563 and 480-0546 to VRC01.** (**A**) Epitope footprints of VRC01, 293-0563, and 480-0546 colored based on contact residues determined from BSA analysis. (**B**) Analyses of binding interface for VRC01, 293-0563, and 480-0546 bound to Env trimers. Buried surface areas were calculated with PISA. BSA contributed by HCDR2s lighted in red and account for approximately 50% of the protein BSA. Framework regions (FRs) and CDRs were determined using Kabat’s definition (*105*). Residue numbering based on the Kabat numbering scheme (*106*).

**Table S1. IAVI G002 study schema with participant group allocations**

| **Group** | **N** | **Week 0** | **Week 8** | **Week 16** |
| --- | --- | --- | --- | --- |
| **1** | 17 | eOD-GT8 60mer mRNA-LNP Vaccine (100µg) | eOD-GT8 60mer mRNA-LNP Vaccine (100µg) | − |
| **2** | 17 | eOD-GT8 60mer mRNA-LNP Vaccine (100µg) | Core-g28v2 60mer mRNA-LNP Vaccine (100µg) | − |
| **3** | 18 | eOD-GT8 60mer mRNA-LNP Vaccine (100µg) | eOD-GT8 60mer mRNA-LNP Vaccine (100µg) | Core-g28v2 60mer mRNA-LNP Vaccine (100µg) |
| **4** | 8 | Core-g28v2 60mer mRNA-LNP Vaccine (100µg) |  | − |
| **TOTAL** | **60*** |  |  |  |

*An over-enrollment of up to 2 participants per group was permitted to facilitate rapid enrollment. A total of 60 participants were enrolled.

**Table S2. IAVI G002 participant enrollment and disposition**

|  | Group 1 (eOD/eOD) (N=17) | Group 2 (eOD/CORE) (N=17) | Group 3 (eOD/eOD/CORE) (N=18) | Group 4 (CORE) (N=8) | Overall (N=60) |
| --- | --- | --- | --- | --- | --- |
| Informed consent signed |  |  |  |  | 78 |
| Eligible |  |  |  |  | 60 |
| Randomized | 17 | 17 | 18 | 8 | 60 |
| Participants who completed the study | 17 (100%) | 17 (100%) | 17 (94.4%) | 7 (87.5%) | 58 (96.7%) |
| Received study product at D1 who discontinued early | 0 (0.0%) | 0 (0.0%) | 1 (5.6%) | 1 (12.5%) | 2 (3.3%) |
| Lost to follow-up* | 0 (0.0%) | 0 (0.0%) | 1 (5.6%) | 1 (12.5%) | 2 (3.3%) |
| Moved from area | 0 (0.0%) | 0 (0.0%) | 0 (0.0%) | 0 (0.0%) | 0 (0.0%) |
| Participant chose to withdraw | 0 (0.0%) | 0 (0.0%) | 0 (0.0%) | 0 (0.0%) | 0 (0.0%) |
| Reactogenicity Event | 0 (0.0%) | 0 (0.0%) | 0 (0.0%) | 0 (0.0%) | 0 (0.0%) |
| Adverse Event/Intercurrent Illness | 0 (0.0%) | 0 (0.0%) | 0 (0.0%) | 0 (0.0%) | 0 (0.0%) |
| Protocol Deviation | 0 (0.0%) | 0 (0.0%) | 0 (0.0%) | 0 (0.0%) | 0 (0.0%) |
| Investigator/Study Decision | 0 (0.0%) | 0 (0.0%) | 0 (0.0%) | 0 (0.0%) | 0 (0.0%) |
| Death | 0 (0.0%) | 0 (0.0%) | 0 (0.0%) | 0 (0.0%) | 0 (0.0%) |
| Other | 0 (0.0%) | 0 (0.0%) | 0 (0.0%) | 0 (0.0%) | 0 (0.0%) |
| Safety analysis population | 17 (100%) | 17 (100%) | 18 (100%) | 8 (100%) | 60 (100%) |
| Stopped IP but remained in the study | 3 (17.6%) | 0 (0.0%) | 4 (22.2%) | 0 (0.0%) | 7 (11.7%) |

*One participant from group 3 was lost to follow-up at the second vaccination visit, and one participant from group 4 was lost to follow up at the Leukapheresis Visit, Day 57 visit. Neither experienced skin AEs prior to being lost to follow up.

**Table S3. IAVI G002 summary of demographics by treatment (randomized population)**

| Demographics and Baseline Characteristics | Group 1 (eOD/eOD) (N=17) | Group 2 (eOD/CORE) (N=17) | Group 3 (eOD/eOD/CORE) (N=18) | Group 4 (CORE) (N=8) | Overall (N=60) |
| --- | --- | --- | --- | --- | --- |
| **Age (yrs)** |  |  |  |  |  |
| n | 17 | 17 | 18 | 8 | 60 |
| Mean (SD) | 29.6 (5.86) | 27.2 (5.00) | 30.0 (6.85) | 27.9 (7.52) | 28.8 (6.15) |
| Median | 28.7 | 25.2 | 30.0 | 25.4 | 27.9 |
| Q1, Q3 | 25.1, 34.3 | 23.9, 30.4 | 23.8, 32.6 | 22.3, 33.4 | 23.9, 31.8 |
| Min, Max | 20.0, 42.8 | 22.0, 42.1 | 20.3, 46.5 | 21.0, 40.4 | 20.0, 46.5 |
| **Sex at Birth, n (%)** |  |  |  |  |  |
| Female | 10 (58.8%) | 10 (58.8%) | 9 (50.0%) | 5 (62.5%) | 34 (56.7%) |
| Male | 7 (41.2%) | 7 (41.2%) | 9 (50.0%) | 3 (37.5%) | 26 (43.3%) |
| **Gender, n (%)** |  |  |  |  |  |
| Male | 6 (35.3%) | 6 (35.3%) | 8 (44.4%) | 3 (37.5%) | 23 (38.3%) |
| Female | 9 (52.9%) | 7 (41.2%) | 8 (44.4%) | 5 (62.5%) | 29 (48.3%) |
| Transgender male (female to male) | 0 (0.0%) | 3 (17.6%) | 1 (5.6%) | 0 (0.0%) | 4 (6.7%) |
| Transgender female (male to female) | 1 (5.9%) | 0 (0.0%) | 1 (5.6%) | 0 (0.0%) | 2 (3.3%) |
| Gender queer | 0 (0.0%) | 0 (0.0%) | 0 (0.0%) | 0 (0.0%) | 0 (0.0%) |
| Gender variant or gender non-conforming | 1 (5.9%) | 1 (5.9%) | 0 (0.0%) | 0 (0.0%) | 2 (3.3%) |
| Self-identify | 0 (0.0%) | 0 (0.0%) | 0 (0.0%) | 0 (0.0%) | 0 (0.0%) |
| Prefer not to answer | 0 (0.0%) | 0 (0.0%) | 0 (0.0%) | 0 (0.0%) | 0 (0.0%) |
| **Ethnicity, n (%)** |  |  |  |  |  |
| Hispanic or Latino | 6 (35.3%) | 1 (5.9%) | 3 (16.7%) | 2 (25.0%) | 12 (20.0%) |
| Not Hispanic or Latino | 11 (64.7%) | 16 (94.1%) | 15 (83.3%) | 6 (75.0%) | 48 (80.0%) |
| Not reported | 0 (0.0%) | 0 (0.0%) | 0 (0.0%) | 0 (0.0%) | 0 (0.0%) |
| Unknown | 0 (0.0%) | 0 (0.0%) | 0 (0.0%) | 0 (0.0%) | 0 (0.0%) |
| **Race, n (%)** |  |  |  |  |  |
| American Indian or Alaskan Native | 1 (5.9%) | 0 (0.0%) | 0 (0.0%) | 0 (0.0%) | 1 (1.7%) |
| Asian | 0 (0.0%) | 1 (5.9%) | 2 (11.1%) | 2 (25.0%) | 5 (8.3%) |
| Black or African American | 0 (0.0%) | 1 (5.9%) | 0 (0.0%) | 0 (0.0%) | 1 (1.7%) |
| Native Hawaiian or Other Pacific Islander | 0 (0.0%) | 0 (0.0%) | 0 (0.0%) | 1 (12.5%) | 1 (1.7%) |
| White | 13 (76.5%) | 13 (76.5%) | 14 (77.8%) | 4 (50.0%) | 44 (73.3%) |
| Multiracial^1^ | 3 (17.6%) | 2 (11.8%) | 2 (11.1%) | 1 (12.5%) | 8 (13.3%) |
| Unknown | 0 (0.0%) | 0 (0.0%) | 0 (0.0%) | 0 (0.0%) | 0 (0.0%) |
| Other | 0 (0.0%) | 0 (0.0%) | 0 (0.0%) | 0 (0.0%) | 0 (0.0%) |
| **Height (cm)** |  |  |  |  |  |
| n | 17 | 17 | 18 | 8 | 60 |
| Mean (SD) | 168.5 (6.93) | 176.0 (9.21) | 172.7 (7.93) | 170.9 (11.48) | 172.2 (8.85) |
| Median | 170.0 | 175.0 | 174.0 | 170.0 | 171.0 |
| Q1, Q3 | 164.0, 171.0 | 170.0, 183.0 | 167.0, 179.0 | 163.5, 178.5 | 166.5, 179.0 |
| Min, Max | 155.0, 183.0 | 156.0, 191.0 | 156.0, 183.0 | 154.0, 189.0 | 154.0, 191.0 |
| **Weight (kg)** |  |  |  |  |  |
| n | 17 | 17 | 18 | 8 | 60 |
| Mean (SD) | 70.6 (13.65) | 79.3 (15.16) | 74.3 (11.55) | 74.0 (22.35) | 74.6 (14.88) |
| Median | 69.0 | 76.0 | 73.5 | 69.5 | 73.5 |
| Q1, Q3 | 59.0, 78.0 | 71.0, 90.0 | 65.0, 79.0 | 59.0, 78.5 | 64.5, 83.0 |
| Min, Max | 52.0, 98.0 | 52.0, 111.0 | 59.0, 104.0 | 54.0, 124.0 | 52.0, 124.0 |
| **BMI (kg/m^2^)** |  |  |  |  |  |
| n | 17 | 17 | 18 | 8 | 60 |
| Mean (SD) | 24.9 (4.43) | 25.5 (4.31) | 24.9 (3.52) | 25.0 (4.70) | 25.1 (4.08) |
| Median | 24.6 | 24.5 | 24.1 | 24.3 | 24.5 |
| Q1, Q3 | 21.2, 27.0 | 22.1, 29.4 | 22.0, 26.5 | 22.2, 26.6 | 21.9, 27.1 |
| Min, Max | 18.2, 33.9 | 19.8, 34.1 | 21.1, 34.7 | 18.9, 34.7 | 18.2, 34.7 |

**Table S4. IAVI G003 study participant enrollment and recruitment.**

|  | eOD-GT8 60mer mRNA-LNP (100µg)  (eOD/eOD)  (N=18) |
| --- | --- |
| Informed consent signed | 44 |
| Eligible | 18 |
| Participants who completed study | 18 (100%) |
| Received study product at Day 1 who discontinued early | 0 (0.0%) |
| Lost to follow-up | 0 (0.0%) |
| Moved from area | 0 (0.0%) |
| Participant chose to withdraw | 0 (0.0%) |
| Reactogenicity Event | 0 (0.0%) |
| Adverse Event/Intercurrent Illness | 0 (0.0%) |
| Protocol Deviation | 0 (0.0%) |
| Investigator/Study Decision | 0 (0.0%) |
| Death | 0 (0.0%) |
| Other | 0 (0.0%) |
| Safety analysis population | 18 (100%) |
| Stopped IP but remained in the study | 0 (0.0%) |

| N = Number of participants who were enrolled |
| --- |
| The denominator of percentage is the number of participants enrolled. |
| n = Number of participants in each category |
| % = Percentage of participants in each category relative to the total number, i.e., 100 x n/N |

**Table S5. IAVI G003 summary of the Demographics of enrolled participants.**

| Demographics and Baseline Characteristics | eOD-GT8 60mer (100ug)  (eOD/eOD)  (N=18) |
| --- | --- |
| **Age (yrs.)** |  |
| Mean (SD) | 32.4 (5.96) |
| Median | 33.7 |
| Q1, Q3 | 28.5, 37.5 |
| Min, Max | 21.6, 41.5 |
| **Sex at Birth, n (%)** |  |
| Female | 8 (44.4%) |
| Male | 10 (55.6%) |
| **Gender, n (%)** |  |
| Male | 10 (55.6%) |
| Female | 8 (44.4%) |
| Transgender male (female to male) | 0 (0.0%) |
| Transgender female (male to female) | 0 (0.0%) |
| Gender queer | 0 (0.0%) |
| Gender variant or gender non-conforming | 0 (0.0%) |
| Self-identify | 0 (0.0%) |
| Prefer not to answer | 0 (0.0%) |
| **Ethnicity, n (%)** |  |
| Not Hispanic or Latino | 18 (100%) |
| **Race, n (%)** |  |
| Black | 18 (100%) |
| **Height (cm)** |  |
| Mean (SD) | 164.9 (7.32) |
| Median | 166.5 |
| Q1, Q3 | 159.0, 170.0 |
| Min, Max | 151.0, 176.0 |
| **Weight (kg)** |  |
| Mean (SD) | 62.5 (8.55) |
| Median | 61.5 |
| Q1, Q3 | 57.0, 65.0 |
| Min, Max | 50.0, 84.0 |
| **BMI** |  |
| Mean (SD) | 23.2 (4.32) |
| Median | 22.3 |
| Q1, Q3 | 19.8, 25.0 |
| Min, Max | 18.4, 35.0 |

**Table S6. IAVI G003 visit dates for enrolled participants.**

| **Table s7. G002 SCHEDULE OF Activities, GROUP 1** | | | | | | | | | | | \| IP administration visit \| FNA visit \| Leukapheresis visit \| \| --- \| --- \| --- \| | | | | | | | | | | |
| --- | --- | --- | --- | --- | --- | --- | --- | --- | --- | --- | --- | --- | --- | --- | --- | --- | --- | --- | --- | --- | --- | --- | --- | --- |
| **Month** | **-2** |  | **0** |  |  |  |  | **1** |  | **2** |  |  |  |  | **3** | **4** |  |  |  | **6** | **8^^[[1]](#endnote-2)^^** |
| **Week** | **-8** | **-5** | **0** |  | **1** | **2** | **3** | **4** |  | **8** |  | **9** | **10** | **11** | **12** | **16** | **18** | **19** | **20** | **24** | **32** |
| **Day** | **-56** | **-35** | **1** | **4** | **8** | **15** | **22** | **29** | **1-5 days prior to Day 57/2^nd^ IP** | **57** | **60** | **64** | **71** | **78** | **85** | **113** | **127** | **134** | **141** | **169** | **225** |
| **Visit Window (Days)** |  | -20^^[[2]](#endnote-3)^^ | 0 | ±1 | +3 | ±3 | ±7 | ±3 |  | ±7^^[[3]](#endnote-4)^^ | ±1 | +3 | ±3 | ±7 | ±4 | ±7 | ±4 | ±7 | -3 to +7 | ±14 | ±14 |
| **Weeks Since Most Recent IP administration** |  |  | 0 |  | 1 | 2 | 3 | 4 |  | 8/0 |  | 1 | 2 | 3 | 4 | 8 | 10 | 11 | 12 | 16 | 24 |
| **Days Since Most Recent IP administration** |  |  | 0 | 3 | 7 | 14 | 21 | 28 |  | 56/0 | 3 | 7 | 14 | 21 | 28 | 56 | 70 | 77 | 84 | 112 | 168 |
| Investigational Product |  |  | X |  |  |  |  |  |  | X |  |  |  |  |  |  |  |  |  |  |  |
| Telephone, text or email contact |  |  |  | X |  |  |  |  |  |  | X |  |  |  |  |  |  |  |  |  |  |
| **CONSENT/ASSESSMENTS/**  **COUNSELING** |  |  |  |  |  |  |  |  |  |  |  |  |  |  |  |  |  |  |  |  |  |
| Informed consent | X |  |  |  |  |  |  |  |  |  |  |  |  |  |  |  |  |  |  |  |  |
| Assessment of understanding | X |  |  |  |  |  |  |  |  |  |  |  |  |  |  |  |  |  |  |  |  |
| HIV risk assessment | X |  |  |  |  |  |  |  |  |  |  |  |  |  |  |  |  |  |  |  | X |
| HIV risk reduction counseling | X |  |  |  |  |  |  |  |  |  |  |  |  |  |  |  |  |  |  |  | X |
| HIV test counseling | X |  |  |  |  |  |  |  |  |  |  |  |  |  |  |  |  |  |  |  | X |
| Family planning counseling | X |  | X |  |  |  |  |  |  | X |  |  |  |  |  | X |  |  |  |  |  |
| Social impact assessment |  |  |  |  |  |  |  |  |  |  |  |  |  |  |  |  |  |  |  |  | X |
| **CLINICAL SAFETY ASSESSMENTS** |  |  |  |  |  |  |  |  |  |  |  |  |  |  |  |  |  |  |  |  |  |
| Comprehensive medical history | X |  |  |  |  |  |  |  |  |  |  |  |  |  |  |  |  |  |  |  |  |
| Interim medical history |  | X | X | X | X | X | X | X | X | X | X | X | X | X | X | X | X | X | X | X | X |
| Concomitant medications^^[[4]](#endnote-5)^^ | X | X | X | X | X | X | X | X |  | X | X | X | X | X | X |  |  |  |  |  |  |
| General physical examination | X |  |  |  |  |  |  |  |  |  |  |  |  |  |  |  |  |  |  |  |  |
| Directed physical examination |  | X | X |  | X | X | X | X | X | X |  | X | X | X | X | X | X | X | X | X | X |
| Height | X |  |  |  |  |  |  |  |  |  |  |  |  |  |  |  |  |  |  |  |  |
| Weight | X |  |  |  |  |  |  |  | X |  |  |  |  |  |  | X |  |  |  |  | X |
| Vital signs | X | X | X |  | X | X | X | X | X | X |  | X | X | X | X | X | X | X | X | X | X |
| (Cervical and) axillary lymph nodes | X |  | X |  | X | X | X |  |  | X |  | X | X | X |  |  |  | X |  |  |  |
| Local and systemic reactogenicity^^[[5]](#endnote-6)^^ |  |  | X | X | X |  |  |  |  | X | X | X |  |  |  |  |  |  |  |  |  |
| Adverse events^^[[6]](#endnote-7)^^ |  |  | X | X | X | X | X | X |  | X | X | X | X | X | X |  |  |  |  |  |  |
| SAEs (and MAAEs and AESIs)^^[[7]](#endnote-8)^^ |  | X | X | X | X | X | X | X | X | X | X | X | X | X | X | X | X | X | X | X | X |
| **CLINICAL LABORATORY TESTING** |  |  |  |  |  |  |  |  |  |  |  |  |  |  |  |  |  |  |  |  |  |
| Screening labs:  Syphilis, hepatitis B & C | X |  |  |  |  |  |  |  |  |  |  |  |  |  |  |  |  |  |  |  |  |
| Urine dipstick | X |  | X |  |  |  |  |  |  | X |  |  |  |  |  |  |  |  |  |  | X |
| Urine pregnancy test | X |  | X |  |  |  | X |  | X | X |  |  |  | X |  | X |  | X |  | X | X |
| Hematology | X |  | X |  | X |  |  |  |  | X |  | X |  |  |  |  |  |  |  |  | X |
| Chemistry | X |  | X |  | X |  |  |  |  | X |  | X |  |  |  |  |  |  |  |  | X |
| HIV testing | X |  |  |  |  |  |  |  |  |  |  |  |  |  |  |  |  |  |  |  | X |
| Stored samples |  |  | X |  | X |  |  |  |  | X |  | X |  |  |  |  |  |  |  |  |  |
| **SAMPLES FOR RESEARCH IMMUNOLOGY TESTING^^[[8]](#endnote-9)^^** |  |  |  |  |  |  |  |  |  |  |  |  |  |  |  |  |  |  |  |  |  |
| Serum binding antibody |  |  | X |  |  | X |  | X |  | X |  |  | X |  |  | X | X |  | X | X |  |
| Serum neutralization |  |  | X |  |  |  |  |  |  |  |  |  | X |  |  |  | X |  |  |  |  |
| B-cell sorting (whole blood) |  | X |  |  |  |  |  | X |  |  |  |  |  |  |  |  |  |  |  | X |  |
| IgDiscover (whole blood) |  | X |  |  |  |  |  |  |  |  |  |  |  |  |  |  |  |  |  |  |  |
| Leukapheresis (B-cell sorting and Ag-specific CD4 T-cells) |  |  |  |  |  |  |  |  | X |  |  |  |  |  |  | X |  |  |  |  |  |
| Fine needle aspirates (B-cell sorting) |  |  |  |  |  |  | X |  |  |  |  |  |  | X |  |  |  | X |  |  |  |
| Whole blood volume (mL) per visit | 27 | 354 | 24 |  | 19 | 10 | 0 | 359 | 45 | 24 |  | 19 | 10 | 0 |  | 55 | 10 | 0 | 10 | 359 | 20 |
| 8-wk running volume (mL) | 27 | 381 | 405 |  | 397 | 407 | 407 | 412 | 457 | 481 |  | 476 | 467 | 457 |  | 108 | 75 | 65 | 75 | 434 | 379 |
| TOTAL running blood volume (mL) | 27 | 381 | 405 |  | 424 | 434 | 434 | 793 | 838 | 862 |  | 881 | 891 | 891 |  | 946 | 956 | 956 | 966 | 1325 | 1345 |

Abbreviations: HIV=Human Immunodeficiency Virus; IP=investigational product; SAEs=serious adverse event; MAAEs=medically attended adverse events; AESIs=adverse events of special interest.

If early termination is required, perform activities for Day 225 visit.

^2^ The window for the Day -35 visit is from determination of eligibility to 35 days prior to the first vaccination.

^3^ Occurs 1-5 days after leukapheresis.

^4^ Concomitant medication taken at any point in the study for the treatment of SAEs, MAAEs, AESIs, Skin AEs or AEs resulting from study procedures will be recorded. All non-study vaccinations received at any point during the study will be recorded.

^5^ Collect reactogenicity data Day 1 through Day 7.

^6^ Clinical events resulting from study procedures such as leukapheresis, FNAs or large blood draws should be reported on the AE CRF throughout the study from screening to final study visit.

^7^ MAAEs and AESIs will be collected from first IP administration up to 24 weeks post last IP administration.

^8^ Serum binding, serum neutralization, B-cell sorting and IgDiscover (whole blood) are from venous blood collection. Refer to the Lab Operations Manual for details on tube types and volume.

| **table s8. G002 SCHEDULE OF Activities, GROUP 2** | | | | | | | | | | \| IP administration visit \| FNA visit \| Leukapheresis visit \| \| --- \| --- \| --- \| | | | | | | | | | | |
| --- | --- | --- | --- | --- | --- | --- | --- | --- | --- | --- | --- | --- | --- | --- | --- | --- | --- | --- | --- | --- | --- | --- | --- |
| **Month** | **-2** |  | **0** |  |  |  |  | **1** |  | **2** |  |  |  |  | **3** | **4** |  |  | **6** | **8^^[[9]](#endnote-10)^^** |
| **Week** | **-8** | **-5** | **0** |  | **1** | **2** | **3** | **4** |  | **8** |  | **9** | **10** | **11** | **12** | **16** | **18** | **20** | **24** | **32** |
| **Day** | **-56** | **-35** | **1** | **4** | **8** | **15** | **22** | **29** | **1-5 days prior to Day 57/2^nd^ IP** | **57** | **60** | **64** | **71** | **78** | **85** | **113** | **127** | **141** | **169** | **225** |
| **Visit Window (Days)** |  | -20^^[[10]](#endnote-11)^^ | 0 | ±1 | +3 | ±3 | ±7 | ±3 |  | ±7^^[[11]](#endnote-12)^^ | ±1 | +3 | ±3 | ±7 | ±4 | ±7 | ±4 | -3 to +7 | ±14 | ±14 |
| **Weeks Since Most Recent IP administration** |  |  | 0 |  | 1 | 2 | 3 | 4 |  | 8/0 |  | 1 | 2 | 3 | 4 | 8 | 10 | 12 | 16 | 24 |
| **Days Since Most Recent IP administration** |  |  | 0 | 3 | 7 | 14 | 21 | 28 |  | 56/0 | 3 | 7 | 14 | 21 | 28 | 56 | 70 | 84 | 112 | 168 |
| Investigational Product |  |  | X |  |  |  |  |  |  | X |  |  |  |  |  |  |  |  |  |  |
| Telephone, text or email contact |  |  |  | X |  |  |  |  |  |  | X |  |  |  |  |  |  |  |  |  |
| **CONSENT/ASSESSMENTS/**  **COUNSELING** |  |  |  |  |  |  |  |  |  |  |  |  |  |  |  |  |  |  |  |  |
| Informed consent | X |  |  |  |  |  |  |  |  |  |  |  |  |  |  |  |  |  |  |  |
| Assessment of understanding | X |  |  |  |  |  |  |  |  |  |  |  |  |  |  |  |  |  |  |  |
| HIV risk assessment | X |  |  |  |  |  |  |  |  |  |  |  |  |  |  |  |  |  |  | X |
| HIV risk reduction counseling | X |  |  |  |  |  |  |  |  |  |  |  |  |  |  |  |  |  |  | X |
| HIV test counseling | X |  |  |  |  |  |  |  |  |  |  |  |  |  |  |  |  |  |  | X |
| Family planning counseling | X |  | X |  |  |  |  |  |  | X |  |  |  |  |  | X |  |  |  |  |
| Social impact assessment |  |  |  |  |  |  |  |  |  |  |  |  |  |  |  |  |  |  |  | X |
| **CLINICAL SAFETY ASSESSMENTS** |  |  |  |  |  |  |  |  |  |  |  |  |  |  |  |  |  |  |  |  |
| Comprehensive medical history | X |  |  |  |  |  |  |  |  |  |  |  |  |  |  |  |  |  |  |  |
| Interim medical history |  | X | X | X | X | X | X | X | X | X | X | X | X | X | X | X | X | X | X | X |
| Concomitant medications^^[[12]](#endnote-13)^^ | X | X | X | X | X | X | X | X |  | X | X | X | X | X | X |  |  |  |  |  |
| General physical examination | X |  |  |  |  |  |  |  |  |  |  |  |  |  |  |  |  |  |  |  |
| Directed physical examination |  | X | X |  | X | X | X | X | X | X |  | X | X | X | X | X | X | X | X | X |
| Height | X |  |  |  |  |  |  |  |  |  |  |  |  |  |  |  |  |  |  |  |
| Weight | X |  |  |  |  |  |  |  | X |  |  |  |  |  |  | X |  |  |  | X |
| Vital signs | X | X | X |  | X | X | X | X | X | X |  | X | X | X | X | X | X | X | X | X |
| (Cervical and) axillary lymph nodes | X |  | X |  | X | X | X |  |  | X |  | X | X | X |  |  |  |  |  |  |
| Local and systemic reactogenicity^^[[13]](#endnote-14)^^ |  |  | X | X | X |  |  |  |  | X | X | X |  |  |  |  |  |  |  |  |
| Adverse events^^[[14]](#endnote-15)^^ |  |  | X | X | X | X | X | X |  | X | X | X | X | X | X |  |  |  |  |  |
| SAEs (and MAAEs and AESIs)^^[[15]](#endnote-16)^^ |  | X | X | X | X | X | X | X | X | X | X | X | X | X | X | X | X | X | X | X |
| **CLINICAL LABORATORY TESTING** |  |  |  |  |  |  |  |  |  |  |  |  |  |  |  |  |  |  |  |  |
| Screening labs:  Syphilis, hepatitis B & C | X |  |  |  |  |  |  |  |  |  |  |  |  |  |  |  |  |  |  |  |
| Urine dipstick | X |  | X |  |  |  |  |  |  | X |  |  |  |  |  |  |  |  |  | X |
| Urine pregnancy test | X |  | X |  |  |  | X |  | X | X |  |  |  | X |  | X |  |  | X | X |
| Hematology | X |  | X |  | X |  |  |  |  | X |  | X |  |  |  |  |  |  |  | X |
| Chemistry | X |  | X |  | X |  |  |  |  | X |  | X |  |  |  |  |  |  |  | X |
| HIV testing | X |  |  |  |  |  |  |  |  |  |  |  |  |  |  |  |  |  |  | X |
| Stored samples |  |  | X |  | X |  |  |  |  | X |  | X |  |  |  |  |  |  |  |  |
| **SAMPLES FOR RESEARCH IMMUNOLOGY TESTING^^[[16]](#endnote-17)^^** |  |  |  |  |  |  |  |  |  |  |  |  |  |  |  |  |  |  |  |  |
| Serum binding antibody |  |  | X |  |  | X |  | X |  | X |  |  | X |  |  | X | X | X | X |  |
| Serum neutralization |  |  | X |  |  |  |  |  |  |  |  |  | X |  |  |  | X |  |  |  |
| B-cell sorting (whole blood) |  | X |  |  |  |  |  | X |  |  |  |  |  |  |  |  |  |  | X |  |
| IgDiscover (whole blood) |  | X |  |  |  |  |  |  |  |  |  |  |  |  |  |  |  |  |  |  |
| Leukapheresis (B-cell sorting and Ag-specific CD4 T-cells) |  |  |  |  |  |  |  |  | X |  |  |  |  |  |  | X |  |  |  |  |
| Fine needle aspirates (B-cell sorting) |  |  |  |  |  |  | X |  |  |  |  |  |  | X |  |  |  |  |  |  |
| Whole blood volume (mL) per visit | 27 | 354 | 24 |  | 19 | 10 | 0 | 359 | 45 | 24 |  | 19 | 10 | 0 |  | 55 | 10 | 10 | 359 | 20 |
| 8-wk running volume (mL) | 27 | 381 | 405 |  | 397 | 407 | 407 | 412 | 457 | 481 |  | 476 | 467 | 457 |  | 108 | 75 | 75 | 434 | 379 |
| TOTAL running blood volume (mL) | 27 | 381 | 405 |  | 424 | 434 | 434 | 793 | 838 | 862 |  | 881 | 891 | 891 |  | 944 | 956 | 966 | 1325 | 1345 |

Abbreviations: HIV=Human Immunodeficiency Virus; IP=investigational product; SAEs=serious adverse events; MAAEs=medically attended adverse events; AESIs=adverse events of special interest.

If early termination is required, perform activities for Day 225 visit.

^2^ The window for the Day -35 visit is from determination of eligibility to 35 days prior to the first vaccination.

^3^ Occurs 1-5 days after leukapheresis.

^4^ Concomitant medication taken at any point in the study for the treatment of SAEs, MAAEs, AESIs, skin AEs or AEs resulting from study procedures will be recorded.

All non-study vaccinations received at any point during the study will be recorded.

^5^ Collect reactogenicity data Day 1 through Day 7.

^6^ Clinical events resulting from study procedures such as leukapheresis, FNAs or large blood draws should be reported on the AE CRF throughout the study from screening to final study visit.

^7^ MAAEs and AESIs will be collected from first IP administration up to 24 weeks post last IP administration.

^8^ Serum binding, serum neutralization, B-cell sorting and IgDiscover (whole blood) are from venous blood collection. Refer to the Lab Operations Manual for details on tube types and volume.

| **table s9. G002 SCHEDULE OF Activities, GROUP 3** | | | | | | | | | | | | | \| IP administration visit \| FNA visit \| Leukapheresis visit \| \| --- \| --- \| --- \| | | | | | | | | | | |
| --- | --- | --- | --- | --- | --- | --- | --- | --- | --- | --- | --- | --- | --- | --- | --- | --- | --- | --- | --- | --- | --- | --- | --- | --- | --- | --- |
| **Month** | **-2** |  | **0** |  |  |  |  | **1** | **2** |  |  |  |  | **3** |  | **4** |  | **5** |  |  |  | **6** | **10^^[[17]](#endnote-18)^^** |
| **Week** | **-8** | **-5** | **0** |  | **1** | **2** | **3** | **4** | **8** |  | **9** | **10** | **11** | **12** |  | **16** |  | **17** | **18** | **19** | **20** | **24** | **40** |
| **Day** | **-56** | **-35** | **1** | **4** | **8** | **15** | **22** | **29** | **57** | **60** | **64** | **71** | **78** | **85** | **1-5 days prior to Day 113/**  **3^rd^ IP** | **113** | **116** | **120** | **127** | **134** | **141** | **169** | **281** |
| **Visit Window** |  | -20^^[[18]](#endnote-19)^^ | 0 | ±1 | +3 | ±3 | ±7 | ±3 | ±7 | ±1 | +3 | ±3 | ±3 | ±3 |  | ±7^^[[19]](#endnote-20)^^ | ±1 | +3 | ±3 | ±7 | -3 to +7 | ±14 | ±14 |
| **Weeks Since Most Recent IP administrations** |  |  | 0 |  | 1 | 2 | 3 | 4 | 8/0 |  | 1 | 2 | 3 | 4 |  | 8/0 |  | 1 | 2 | 3 | 4 | 8 | 24 |
| **Days Since Most Recent IP administration** |  |  | 0 | 3 | 7 | 14 | 21 | 28 | 56/0 | 3 | 7 | 14 | 21 | 28 |  | 56/0 | 3 | 7 | 14 | 21 | 28 | 56 | 168 |
| Investigational Product |  |  | X |  |  |  |  |  | X |  |  |  |  |  |  | X |  |  |  |  |  |  |  |
| Telephone, text or email contact |  |  |  | X |  |  |  |  |  | X |  |  |  |  |  |  | X |  |  |  |  |  |  |
| **CONSENT/ASSESSMENT/**  **COUNSELING** |  |  |  |  |  |  |  |  |  |  |  |  |  |  |  |  |  |  |  |  |  |  |  |
| Informed consent | X |  |  |  |  |  |  |  |  |  |  |  |  |  |  |  |  |  |  |  |  |  |  |
| Assessment of Understanding | X |  |  |  |  |  |  |  |  |  |  |  |  |  |  |  |  |  |  |  |  |  |  |
| HIV risk assessment | X |  |  |  |  |  |  |  |  |  |  |  |  |  |  |  |  |  |  |  |  |  | X |
| HIV risk reduction counseling | X |  |  |  |  |  |  |  |  |  |  |  |  |  |  |  |  |  |  |  |  |  | X |
| HIV test counseling | X |  |  |  |  |  |  |  |  |  |  |  |  |  |  |  |  |  |  |  |  |  | X |
| Family planning counseling | X |  | X |  |  |  |  |  | X |  |  |  |  |  |  | X |  |  |  |  |  | X |  |
| Social impact assessment |  |  |  |  |  |  |  |  |  |  |  |  |  |  |  |  |  |  |  |  |  |  | X |
| **CLINICAL SAFETY ASSESSMENTS** |  |  |  |  |  |  |  |  |  |  |  |  |  |  |  |  |  |  |  |  |  |  |  |
| Comprehensive medical history | X |  |  |  |  |  |  |  |  |  |  |  |  |  |  |  |  |  |  |  |  |  |  |
| Interim medical history |  | X | X | X | X | X | X | X | X | X | X | X | X | X | X | X | X | X | X | X | X | X | X |
| Concomitant medications^^[[20]](#endnote-21)^^ | X | X | X | X | X | X | X | X | X | X | X | X | X | X |  | X | X | X | X | X | X |  |  |
| General physical examination | X |  |  |  |  |  |  |  |  |  |  |  |  |  |  |  |  |  |  |  |  |  |  |
| Directed physical examination |  | X | X |  | X | X | X | X | X |  | X | X | X | X | X | X |  | X | X | X | X | X | X |
| Height | X |  |  |  |  |  |  |  |  |  |  |  |  |  |  |  |  |  |  |  |  |  |  |
| Weight | X |  |  |  |  |  |  |  |  |  |  |  |  |  | X |  |  |  |  |  |  | X | X |
| Vital signs | X | X | X |  | X | X | X | X | X |  | X | X | X | X | X | X |  | X | X | X |  | X | X |
| (Cervical and) axillary lymph nodes | X |  | X |  | X | X | X |  | X |  | X | X |  |  |  | X |  | X | X | X |  |  |  |
| Local and systemic reactogenicity^^[[21]](#endnote-22)^^ |  |  | X | X | X |  |  |  | X | X | X |  |  |  |  | X | X | X |  |  |  |  |  |
| Adverse events^^[[22]](#endnote-23)^^ |  |  | X | X | X | X | X | X | X | X | X | X | X | X |  | X | X | X | X | X | X |  |  |
| SAEs (and MAAEs and AESIs)^^[[23]](#endnote-24)^^ |  | X | X | X | X | X | X | X | X | X | X | X | X | X | X | X | X | X | X | X | X | X | X |
| **CLINICAL LABORATORY TESTING** |  |  |  |  |  |  |  |  |  |  |  |  |  |  |  |  |  |  |  |  |  |  |  |
| Screening labs; syphilis, hepatitis B & C | X |  |  |  |  |  |  |  |  |  |  |  |  |  |  |  |  |  |  |  |  |  |  |
| Urine dipstick | X |  | X |  |  |  |  |  | X |  |  |  |  |  |  | X |  |  |  |  |  |  | X |
| Urine pregnancy test | X |  | X |  |  |  | X |  | X |  |  |  |  |  | X | X |  |  |  | X |  | X | X |
| Hematology | X |  | X |  | X |  |  |  | X |  | X |  |  |  |  | X |  | X |  |  |  |  | X |
| Chemistry | X |  | X |  | X |  |  |  | X |  | X |  |  |  |  | X |  | X |  |  |  |  | X |
| HIV testing | X |  |  |  |  |  |  |  |  |  |  |  |  |  |  |  |  |  |  |  |  |  | X |
| Stored samples |  |  | X |  | X |  |  |  | X |  | X |  |  |  |  | X |  | X |  |  |  |  |  |
| **SAMPLES FOR RESEARCH IMMUNOLOGY TESTING^^[[24]](#endnote-25)^^** |  |  |  |  |  |  |  |  |  |  |  |  |  |  |  |  |  |  |  |  |  |  |  |
| Serum binding antibody |  |  | X |  |  | X |  | X | X |  |  | X |  |  |  | X |  |  | X |  | X | X |  |
| Serum neutralization |  |  | X |  |  |  |  |  |  |  |  | X |  |  |  |  |  |  | X |  |  |  |  |
| B-cell sorting (whole blood) |  | X |  |  |  |  |  | X | X |  |  |  |  |  |  |  |  |  |  |  |  |  |  |
| IgDiscover (whole blood) |  | X |  |  |  |  |  |  |  |  |  |  |  |  |  |  |  |  |  |  |  |  |  |
| Leukapheresis (B-cell sorting, Ag‑specific CD4 T-cells) |  |  |  |  |  |  |  |  |  |  |  |  |  |  | X |  |  |  |  |  |  | X |  |
| Fine need aspirates (B-cell sorting) |  |  |  |  |  |  | X |  |  |  |  |  |  |  |  |  |  |  |  | X |  |  |  |
| Whole blood volume (mL) per visit | 27 | 354 | 24 |  | 19 | 10 | 0 | 223 | 237 |  | 19 | 10 |  |  | 45 | 24 |  | 19 | 10 | 0 | 10 | 55 | 20 |
| 8-wk running volume (mL) | 27 | 381 | 405 |  | 397 | 407 | 407 | 276 | 512 |  | 507 | 498 |  |  | 311 | 335 |  | 117 | 108 | 98 | 108 | 118 | 20 |
| TOTAL running blood volume (mL) | 27 | 381 | 405 |  | 424 | 434 | 434 | 657 | 894 |  | 913 | 923 |  |  | 968 | 992 |  | 1011 | 1021 | 1021 | 1031 | 1086 | 1106 |

Abbreviations: HIV=Human Immunodeficiency Virus; IP=investigational product; SAEs=serious adverse events; MAAEs=medically attended adverse events; AESIs=adverse events of special interest.

If early termination is required, perform activities for Day 281 visit.

^2^ The window for the Day -35 visit is from determination of eligibility to 35 days prior to the first vaccination.

^3^ Occurs 1-5 days after leukapheresis.

^4^ Concomitant medication taken at any point in the study for the treatment of SAEs, MAAEs, AESIs, skin AEs or AEs resulting from study procedures will be recorded. All non-study vaccinations received

at any point during the study will be recorded.

^5^ Collect reactogenicity data Day 1 through Day 7.

^6^ Clinical events resulting from study procedures such as leukapheresis, FNAs or large blood draws should be reported on the AE CRF throughout the study from screening to final study visit.

^7^ MAAEs and AESIs will be collected from first IP administration up to 24 weeks post last IP administration.

^8^ Serum binding, serum neutralization, B-cell sorting and IgDiscover (whole blood) are from venous blood collection. Refer to the Lab Operations Manual for details on tube types and volume.

| **table s10. G002 SCHEDULE OF ACTIVITIES, GROUP 4** | | | | \| IP administration visit \| FNA visit \| Leukapheresis visit \| \| --- \| --- \| --- \| | | | | | | | |
| --- | --- | --- | --- | --- | --- | --- | --- | --- | --- | --- | --- | --- | --- | --- |
| **Month** | **-2** |  | **0** |  |  |  |  | **1** | **2** | **4** | **6^^[[25]](#endnote-26)^^** |
| **Week** | **-8** | **-5** | **0** |  | **1** | **2** | **3** | **4** | **8** | **16** | **24** |
| **Day** | **-56** | **-35** | **1** | **4** | **8** | **15** | **22** | **29** | **57** | **113** | **169** |
| **Visit Window (Days)** |  | -20^^[[26]](#endnote-27)^^ | 0 | ±1 | +3 | ±3 | ±7 | ±3 | ±7 | ±7 | ±14 |
| **Weeks Since Most Recent IP administration** |  |  | 0 |  | 1 | 2 | 3 | 4 | 8 | 16 | 24 |
| **Days Since Most Recent IP administration** |  |  | 0 | 3 | 7 | 14 | 21 | 28 | 56 | 112 | 168 |
| Investigational Product |  |  | X |  |  |  |  |  |  |  |  |
| Telephone, text or email contact |  |  |  | X |  |  |  |  |  |  |  |
| **CONSENT/ASSESSMENTS/COUNSELING** |  |  |  |  |  |  |  |  |  |  |  |
| Informed consent | X |  |  |  |  |  |  |  |  |  |  |
| Assessment of understanding | X |  |  |  |  |  |  |  |  |  |  |
| HIV risk assessment | X |  |  |  |  |  |  |  |  |  | X |
| HIV risk reduction counseling | X |  |  |  |  |  |  |  |  |  | X |
| HIV test counseling | X |  |  |  |  |  |  |  |  |  | X |
| Family planning counseling | X |  | X |  |  |  |  |  | X |  |  |
| Social impact assessment |  |  |  |  |  |  |  |  |  |  | X |
| **CLINICAL SAFETY ASSESSMENTS** |  |  |  |  |  |  |  |  |  |  |  |
| Comprehensive medical history | X |  |  |  |  |  |  |  |  |  |  |
| Interim medical history |  | X | X | X | X | X | X | X | X | X | X |
| Concomitant medications^^[[27]](#endnote-28)^^ | X | X | X | X | X | X | X | X |  |  |  |
| General physical examination | X |  |  |  |  |  |  |  |  |  |  |
| Directed physical examination |  | X | X |  | X | X | X | X | X | X | X |
| Height | X |  |  |  |  |  |  |  |  |  |  |
| Weight | X |  |  |  |  |  |  |  | X |  | X |
| Vital signs | X | X | X |  | X | X | X | X | X | X | X |
| (Cervical and) axillary lymph nodes | X |  | X |  | X | X | X |  |  |  |  |
| Local and systemic reactogenicity^^[[28]](#endnote-29)^^ |  |  | X | X | X |  |  |  |  |  |  |
| Adverse events^^[[29]](#endnote-30)^^ |  |  | X | X | X | X | X | X |  |  |  |
| SAEs (and MAAEs and AESIs)^^[[30]](#endnote-31)^^ |  | X | X | X | X | X | X | X | X | X | X |
| **CLINICAL LABORATORY TESTING** |  |  |  |  |  |  |  |  |  |  |  |
| Screening labs: Syphilis, hepatitis B & C | X |  |  |  |  |  |  |  |  |  |  |
| Urine dipstick | X |  | X |  |  |  |  |  |  |  | X |
| Urine pregnancy test | X |  | X |  |  |  | X |  | X | X | X |
| Hematology | X |  | X |  | X |  |  |  |  |  | X |
| Chemistry | X |  | X |  | X |  |  |  |  |  | X |
| HIV testing | X |  |  |  |  |  |  |  |  |  | X |
| Stored samples |  |  | X |  | X |  |  |  |  |  |  |
| **SAMPLES FOR RESEARCH IMMUNOLOGY TESTING^^[[31]](#endnote-32)^^** |  |  |  |  |  |  |  |  |  |  |  |
| Serum binding antibody |  |  | X |  |  | X |  | X | X |  |  |
| Serum neutralizing |  |  | X |  |  | X |  |  | X |  |  |
| B-cell sorting (whole blood) |  | X |  |  |  |  |  | X |  |  |  |
| IgDiscover (whole blood) |  | X |  |  |  |  |  |  |  |  |  |
| Leukapheresis (B-cell sorting and Ag-specific CD4 T-cells) |  |  |  |  |  |  |  |  | X |  |  |
| Fine needle aspirates (B-cell sorting) |  |  |  |  |  |  | X |  |  |  |  |
| Whole blood volume (mL) per visit | 27 | 354 | 24 |  | 19 | 10 | 0 | 359 | 55 | 0 | 20 |
| 8-wk running volume (mL) | 27 | 381 | 405 |  | 397 | 407 | 407 | 412 | 467 | 55 | 20 |
| TOTAL running blood volume (mL) | 27 | 381 | 405 |  | 424 | 434 | 434 | 793 | 848 | 848 | 868 |

Abbreviations: HIV=Human Immunodeficiency Virus; IP=investigational product; SAEs=serious adverse events; MAAEs=medically attended adverse events; AESIs=adverse events of special interest.

If early termination is required, perform activities for Day 169 visit.

^2^ The window for the Day -35 visit is from determination of eligibility to 35 days prior to the first vaccination.

^3^ Concomitant medication taken at any point in the study for the treatment of SAEs, MAAEs, AESIs, skin AEs or AEs resulting from study procedures will be recorded. All non-study vaccinations received

at any point during the study will be recorded.

^4^ Collect reactogenicity data Day 1 through Day 7.

^5^ Clinical events resulting from study procedures such as leukapheresis, FNAs or large blood draws should be reported on the AE CRF throughout the study from screening to final study visit.

^6^ MAAEs and AESIs will be collected from first IP administration up to 24 weeks post last IP administration.

^7^ Serum binding, serum neutralization, B-cell sorting and IgDiscover (whole blood) are from venous blood collection. Refer to the Lab Operations Manual for details on tube types and volu

**Table S11. G003 schedule of activities (CFHR, Rwanda)**

|  | | | | | | | | | | | **IP administration visit** | | | | **FNA visit** | | | |
| --- | --- | --- | --- | --- | --- | --- | --- | --- | --- | --- | --- | --- | --- | --- | --- | --- | --- | --- |
| **Month** | **-2** |  | **0** |  |  |  |  | **1** | **2** |  |  |  |  | **3** | **4** |  |  | **8^1^** |
| **Week** | **-8** | **-5** | **0** |  | **1** | **2** | **3** | **4** | **8** |  | **9** | **10** | **11** | **12** | **16** | **20** | **21** | **32** |
| **Day** | **-56** | **-35** | **1** | **4** | **8** | **15** | **22** | **29** | **57** | **60** | **64** | **71** | **78** | **85** | **113** | **141** | **148** | **225** |
| **Visit window (Days)** |  | **-20^2^** | **0** | **±1** | **+3** | **±3** | **±3** | **±3** | **±7** | **±1** | **±3** | **±3** | **±3** | **±4** | **±7** | **-3 to +7** | **±3** | **±14** |
| **Weeks since most recent IP admin.** |  |  | **0** |  | **1** | **2** | **3** | **4** | **8/0** |  | **1** | **2** | **3** | **4** | **8** | **12** | **13** | **24** |
| **Days since most recent IP admin.** |  |  | **0** | **3** | **7** | **14** | **21** | **28** | **56/0** | **3** | **7** | **14** | **21** | **28** | **56** | **84** | **91** | **168** |
| IP ADMINISTRATION |  |  | X |  |  |  |  |  | X |  |  |  |  |  |  |  |  |  |
| CONTACT VISIT/CALL^3^ |  |  |  | X |  |  |  |  |  | X |  |  |  |  |  |  |  |  |
| CONSENT/ASSESSMENTS/ COUNSELING |  |  |  |  |  |  |  |  |  |  |  |  |  |  |  |  |  |  |
| Informed consent | X |  |  |  |  |  |  |  |  |  |  |  |  |  |  |  |  |  |
| Assessment of understanding | X |  |  |  |  |  |  |  |  |  |  |  |  |  |  |  |  |  |
| HIV risk assessment | X |  |  |  |  |  |  |  |  |  |  |  |  |  |  |  |  | X |
| HIV risk reduction counseling | X |  |  |  |  |  |  |  |  |  |  |  |  | X |  |  |  | X |
| HIV test counseling | X |  |  |  |  |  |  |  |  |  |  |  |  | X |  |  |  | X |
| Family planning counseling | X |  | X |  |  |  |  |  | X |  |  |  |  |  | X |  |  |  |
| Social impact assessment |  |  |  |  |  |  |  |  |  |  |  |  |  |  |  |  |  | X |
| Structured interview |  | X |  |  |  |  |  |  |  |  |  |  |  |  |  | X |  | X |
| CLINICAL SAFETY ASSESSMENTS |  |  |  |  |  |  |  |  |  |  |  |  |  |  |  |  |  |  |
| Comprehensive medical history | X |  |  |  |  |  |  |  |  |  |  |  |  |  |  |  |  |  |
| Interim medical history |  | X | X | X | X | X | X | X | X | X | X | X | X | X | X | X | X | X |
| Concomitant medications^4^ | X | X | X | X | X | X | X | X | X | X | X | X | X | X | X | X | X | X |
| General physical examination | X |  |  |  |  |  |  |  |  |  |  |  |  |  |  |  |  |  |
| Directed physical examination |  | X | X |  | X | X | X | X | X |  | X | X | X | X | X | X | X | X |
| Height | X |  |  |  |  |  |  |  |  |  |  |  |  |  |  |  |  |  |
| Weight | X |  |  |  |  |  |  |  |  |  |  |  |  |  |  |  |  | X |
| Vital signs | X | X | X |  | X | X | X | X | X |  | X | X | X | X | X | X | X | X |
| Cervical & axillary lymph nodes | X |  | X |  | X | X | X |  | X |  | X | X | X |  | X |  | X |  |
| Local & systemic reactogenicity^5^ |  |  | X | X | X |  |  |  | X | X | X |  |  |  |  |  |  |  |
| Adverse events^6^ |  |  | X | X | X | X | X | X | X | X | X | X | X | X |  |  |  |  |
| SAEs (and MAAEs and AESIs)^7^ |  | X | X | X | X | X | X | X | X | X | X | X | X | X | X | X | X | X |
| CLINICAL LABORATORY TESTING |  |  |  |  |  |  |  |  |  |  |  |  |  |  |  |  |  |  |
| Syphilis, hepatitis B & C | X |  |  |  |  |  |  |  |  |  |  |  |  |  |  |  |  |  |
| Urine dipstick | X |  | X |  |  |  |  |  | X |  |  |  |  |  |  |  |  | X |
| Urine pregnancy test | X |  | X |  |  |  | X |  | X |  |  |  | X |  | X |  |  | X |
| Hematology | X |  | X |  | X |  |  |  | X |  | X |  |  |  |  |  |  | X |
| Chemistry | X |  | X |  | X |  |  |  | X |  | X |  |  |  |  |  |  | X |
| HIV testing | X |  | X |  |  |  |  |  |  |  |  |  |  | X |  |  |  | X |
| Sample storage |  |  | X |  | X |  |  |  | X |  | X |  |  |  |  |  |  |  |
| IMMUNOLOGY ASSESSMENTS^8^ |  |  |  |  |  |  |  |  |  |  |  |  |  |  |  |  |  |  |
| Serum binding antibody |  | X |  |  |  | X |  |  | X |  |  | X |  |  | X |  |  |  |
| B-cell sorting (whole blood) |  | X |  |  |  |  |  |  | X |  |  | X |  |  | X |  | X |  |
| IgDiscover (whole blood) |  | X |  |  |  |  |  |  |  |  |  |  |  |  |  |  |  |  |
| FNA (B-cell sorting) |  |  |  |  |  |  | X |  |  |  |  |  | X |  |  |  |  |  |

IP = investigational product, FNA = fine needle aspiration, admin.=administration, HIV = Human Immunodeficiency Virus, SAE = serious adverse event; MAAE = medically attended adverse event, AESI = adverse event of special interest

^1^If early termination was required, activities for the Day 225 visit were to be performed.

^2^The window for the Day -35 visit was from determination of eligibility to 35 days prior to the first IP administration

^3^Physical, telephone, text, or email contact

^4^Concomitant medication taken at any point in the study for the treatment of SAEs, MAAEs, AESIs or AEs resulting from study procedures was recorded

^5^Reactogenicity data (solicited AEs) were collected from Day 1 through Day 7

^6^Clinical events resulting from study procedures such as FNAs or large blood draws were reported on the AE CRF throughout the study from screening to final study visit

^7^MAAEs and AESIs were required to be collected from first IP administration up to 24 weeks post last IP administration

^8^Serum binding, B-cell sorting and IgDiscover (whole blood) were from venous blood collection

**Table S12. G003 schedule of activities (Aurum Tembisa CRC, South Africa)**

|  | | | | | | | | IP administration visit | | | | FNA visit | | | Leukapheresis visit | | | |
| --- | --- | --- | --- | --- | --- | --- | --- | --- | --- | --- | --- | --- | --- | --- | --- | --- | --- | --- |
| Month | -2 |  | 0 |  |  |  |  | 1 | 2 |  |  |  |  | 3 | 4 |  |  | 8^1^ |
| Week | -8 | -5 | 0 |  | 1 | 2 | 3 | 4 | 8 |  | 9 | 10 | 11 | 12 | 16 | 20 | 21 | 32 |
| Day | -56 | -35 | 1 | 4 | 8 | 15 | 22 | 29 | 57 | 60 | 64 | 71 | 78 | 85 | 113 | 141 | 148 | 225 |
| Visit window (Days) |  | -20^2^ | 0 | ±1 | +3 | ±3 | ±3 | ±3 | ±7 | ±1 | ±3 | ±3 | ±3 | ±4 | ±7 | -3 to +7 | ±3 | ±14 |
| Weeks since most recent IP admin. |  |  | 0 |  | 1 | 2 | 3 | 4 | 8/0 |  | 1 | 2 | 3 | 4 | 8 | 12 | 13 | 24 |
| Days since most recent IP admin. |  |  | 0 | 3 | 7 | 14 | 21 | 28 | 56/0 | 3 | 7 | 14 | 21 | 28 | 56 | 84 | 91 | 168 |
| IP ADMINISTRATION |  |  | X |  |  |  |  |  | X |  |  |  |  |  |  |  |  |  |
| CONTACT VISIT/CALL^3^ |  |  |  | X |  |  |  |  |  | X |  |  |  |  |  |  |  |  |
| CONSENT/ASSESSMENTS/  COUNSELING |  |  |  |  |  |  |  |  |  |  |  |  |  |  |  |  |  |  |
| Informed consent | X |  |  |  |  |  |  |  |  |  |  |  |  |  |  |  |  |  |
| Assessment of understanding | X |  |  |  |  |  |  |  |  |  |  |  |  |  |  |  |  |  |
| HIV risk assessment | X |  |  |  |  |  |  |  |  |  |  |  |  |  |  |  |  | X |
| HIV risk reduction counseling | X |  |  |  |  |  |  |  |  |  |  |  |  | X |  |  |  | X |
| HIV test counseling | X |  |  |  |  |  |  |  |  |  |  |  |  | X |  |  |  | X |
| Family planning counseling | X |  | X |  |  |  |  |  | X |  |  |  |  |  | X |  |  |  |
| Social impact assessment |  |  |  |  |  |  |  |  |  |  |  |  |  |  |  |  |  | X |
| Structured interview |  | X |  |  |  |  |  |  |  |  |  |  |  |  |  | X |  | X |
| CLINICAL SAFETY ASSESSMENTS |  |  |  |  |  |  |  |  |  |  |  |  |  |  |  |  |  |  |
| Comprehensive medical history | X |  |  |  |  |  |  |  |  |  |  |  |  |  |  |  |  |  |
| Interim medical history |  | X | X | X | X | X | X | X | X | X | X | X | X | X | X | X | X | X |
| Concomitant medications^4^ | X | X | X | X | X | X | X | X | X | X | X | X | X | X | X | X | X | X |
| General physical examination | X |  |  |  |  |  |  |  |  |  |  |  |  |  |  |  |  |  |
| Directed physical examination |  | X | X |  | X | X | X | X | X |  | X | X | X | X | X | X | X | X |
| Height | X |  |  |  |  |  |  |  |  |  |  |  |  |  |  |  |  |  |
| Weight | X |  |  |  |  |  |  |  |  |  |  |  |  |  | X |  |  | X |
| Vital signs | X | X | X |  | X | X | X | X | X |  | X | X | X | X | X | X | X | X |
| Cervical & axillary lymph nodes | X |  | X |  | X | X | X |  | X |  | X | X | X |  | X |  | X |  |
| Local and systemic reactogenicity^5^ |  |  | X | X | X |  |  |  | X | X | X |  |  |  |  |  |  |  |
| Adverse events^6^ |  |  | X | X | X | X | X | X | X | X | X | X | X | X |  |  |  |  |
| SAEs (and MAAEs and AESIs)^7^ |  | X | X | X | X | X | X | X | X | X | X | X | X | X | X | X | X | X |
| CLINICAL LABORATORY TESTS |  |  |  |  |  |  |  |  |  |  |  |  |  |  |  |  |  |  |
| Syphilis, hepatitis B & C | X |  |  |  |  |  |  |  |  |  |  |  |  |  |  |  |  |  |
| Urine dipstick | X |  | X |  |  |  |  |  | X |  |  |  |  |  |  |  |  | X |
| Urine pregnancy test | X |  | X |  |  |  | X |  | X |  |  |  | X |  | X |  |  | X |
| Hematology | X |  | X |  | X |  |  |  | X |  | X |  |  |  |  |  |  | X |
| Chemistry | X |  | X |  | X |  |  |  | X |  | X |  |  |  |  |  |  | X |
| HIV testing | X |  | X |  |  |  |  |  |  |  |  |  |  | X |  |  |  | X |
| Sample storage |  |  | X |  | X |  |  |  | X |  | X |  |  |  |  |  |  |  |
| IMMUNOLOGY ASSESSMENTS^8^ |  |  |  |  |  |  |  |  |  |  |  |  |  |  |  |  |  |  |
| Serum binding antibody |  | X |  |  |  | X |  |  | X |  |  | X |  |  | X |  |  |  |
| B-cell sorting (whole blood) |  | X |  |  |  |  |  |  | X |  |  | X |  |  |  |  | X |  |
| IgDiscover (whole blood) |  | X |  |  |  |  |  |  |  |  |  |  |  |  |  |  |  |  |
| Leukapheresis (B-cell sorting and Ag-specific CD4 T-cells) |  |  |  |  |  |  |  |  |  |  |  |  |  |  | X |  |  |  |
| FNA (B-cell sorting) |  |  |  |  |  |  | X |  |  |  |  |  | X |  |  |  |  |  |

IP = investigational product, FNA = fine needle aspiration, admin.=administration, HIV = Human Immunodeficiency Virus, SAE = serious adverse event; MAAE = medically attended adverse event, AESI = adverse event of special interest

^1^If early termination was required, activities for the Day 225 visit were to be performed.

^2^The window for the Day -35 visit was from determination of eligibility to 35 days prior to the first IP administration

^3^Physical, telephone, text, or email contact

^4^Concomitant medication taken at any point in the study for the treatment of SAEs, MAAEs, AESIs or AEs resulting from study procedures was recorded

^5^Reactogenicity data (solicited AEs) were collected from Day 1 through Day 7

^6^Clinical events resulting from study procedures such as FNAs or large blood draws were reported on the AE CRF throughout the study from screening to final study visit

^7^MAAEs and AESIs were required to be collected from first IP administration up to 24 weeks post last IP administration

^8^Serum binding, B-cell sorting and IgDiscover (whole blood) were from venous blood collection

**Table S13. G002 listing of missed vaccinations.** Immunological assays carried out after the missed vaccinations were excluded from the final analysis.

| **PubID** | **Study Group** | **Dose #** | **Was vaccination administered?** | **Immunological assay data was excluded from final analysis after this timepoint:** |
| --- | --- | --- | --- | --- |
| G002-254 | Group 1 | 2 (eOD) | No | week 8 |
| G002-479 | Group 1 | 2 (eOD) | No | week 8 |
| G002-733 | Group 1 | 2 (eOD) | No | week 8 |
| G002-462 | Group 3 | 3 (Core) | No | week 16 |
| G002-477 | Group 3 | 3 (Core) | No | week 16 |
| G002-632 | Group 3 | 3 (Core) | No | week 16 |
| G002-689 | Group 3 | 2 (eOD) & 3 (Core) | No | week 8 |
| G002-810 | Group 3 | 3 (Core) | No | week 16 |

**Table S14. IAVI G002 overall summary of adverse events by treatment group.**

| n (%) (95% CI) [events] | Group 1 (eOD/eOD) (N=17) | Group 2 (eOD/CORE) (N=17) | Group 3 (eOD/eOD/CORE) (N=18) | Group 4 (CORE) (N=8) | Overall (N=60) |
| --- | --- | --- | --- | --- | --- |
| Any Adverse Events^1^ | 17 (100%) | 17 (100%) | 18 (100%) | 8 (100%) | 60 (100%) |
|  | (80.5, 100) | (80.5, 100) | (81.5, 100) | (63.1, 100) | (94.0, 100) |
|  | [172] | [168] | [240] | [30] | [610] |
| Any Solicited Adverse Events^2^ | 17 (100%) | 17 (100%) | 18 (100%) | 8 (100%) | 60 (100%) |
|  | (80.5, 100) | (80.5, 100) | (81.5, 100) | (63.1, 100) | (94.0, 100) |
|  | [124] | [127] | [196] | [28] | [475] |
| Local Solicited Adverse Events | 17 (100%) | 17 (100%) | 18 (100%) | 7 (87.5%) | 59 (98.3%) |
|  | (80.5, 100) | (80.5, 100) | (81.5, 100) | (47.3, 99.7) | (91.1, 100) |
|  | [45] | [59] | [67] | [11] | [182] |
| Grade 3 or Higher | 0 (0.0%) | 3 (17.6%) | 2 (11.1%) | 2 (25.0%) | 7 (11.7%) |
|  | (0.0, 19.5) | (3.8, 43.4) | (1.4, 34.7) | (3.2, 65.1) | (4.8, 22.6) |
|  | [0] | [3] | [2] | [2] | [7] |
| Systemic Solicited Adverse Events | 17 (100%) | 17 (100%) | 18 (100%) | 5 (62.5%) | 57 (95.0%) |
|  | (80.5, 100) | (80.5, 100) | (81.5, 100) | (24.5, 91.5) | (86.1, 99.0) |
|  | [79] | [68] | [129] | [17] | [293] |
| Grade 3 or Higher | 4 (23.5%) | 3 (17.6%) | 4 (22.2%) | 2 (25.0%) | 13 (21.7%) |
|  | (6.8, 49.9) | (3.8, 43.4) | (6.4, 47.6) | (3.2, 65.1) | (12.1, 34.2) |
|  | [8] | [6] | [6] | [2] | [22] |
| Any Unsolicited Adverse Events^3^ | 14 (82.4%) | 14 (82.4%) | 15 (83.3%) | 2 (25.0%) | 45 (75.0%) |
|  | (56.6, 96.2) | (56.6, 96.2) | (58.6, 96.4) | (3.2, 65.1) | (62.1, 85.3) |
|  | [48] | [41] | [44] | [2] | [135] |
| Related^4^ | 6 (35.3%) | 6 (35.3%) | 8 (44.4%) | 0 (0.0%) | 20 (33.3%) |
|  | (14.2, 61.7) | (14.2, 61.7) | (21.5, 69.2) | (0.0, 36.9) | (21.7, 46.7) |
|  | [12] | [10] | [15] | [0] | [37] |
| Grade 3 or Higher | 2 (11.8%) | 0 (0.0%) | 0 (0.0%) | 0 (0.0%) | 2 (3.3%) |
|  | (1.5, 36.4) | (0.0, 19.5) | (0.0, 18.5) | (0.0, 36.9) | (0.4, 11.5) |
|  | [2] | [0] | [0] | [0] | [2] |
| Related^4^ and Grade 3 or Higher | 1 (5.9%) | 0 (0.0%) | 0 (0.0%) | 0 (0.0%) | 1 (1.7%) |
|  | (0.1, 28.7) | (0.0, 19.5) | (0.0, 18.5) | (0.0, 36.9) | (0.0, 8.9) |
|  | [1] | [0] | [0] | [0] | [1] |
| Serious Adverse Events (SAEs)^5^ | 0 (0.0%) | 0 (0.0%) | 0 (0.0%) | 0 (0.0%) | 0 (0.0%) |
|  | (0.0, 19.5) | (0.0, 19.5) | (0.0, 18.5) | (0.0, 36.9) | (0.0, 6.0) |
|  | [0] | [0] | [0] | [0] | [0] |
| Related^4^ | 0 (0.0%) | 0 (0.0%) | 0 (0.0%) | 0 (0.0%) | 0 (0.0%) |
|  | (0.0, 19.5) | (0.0, 19.5) | (0.0, 18.5) | (0.0, 36.9) | (0.0, 6.0) |
|  | [0] | [0] | [0] | [0] | [0] |
| Medically Attended Adverse Events (MAAEs)^6^ | 11 (64.7%) | 9 (52.9%) | 7 (38.9%) | 2 (25.0%) | 29 (48.3%) |
|  | (38.3, 85.8) | (27.8, 77.0) | (17.3, 64.3) | (3.2, 65.1) | (35.2, 61.6) |
|  | [21] | [15] | [11] | [2] | [49] |
| Related^4^ | 2 (11.8%) | 1 (5.9%) | 0 (0.0%) | 0 (0.0%) | 3 (5.0%) |
|  | (1.5, 36.4) | (0.1, 28.7) | (0.0, 18.5) | (0.0, 36.9) | (1.0, 13.9) |
|  | [4] | [3] | [0] | [0] | [7] |
| Adverse Events of Special Interest (AESI)^6^ | 0 (0.0%) | 0 (0.0%) | 0 (0.0%) | 0 (0.0%) | 0 (0.0%) |
|  | (0.0, 19.5) | (0.0, 19.5) | (0.0, 18.5) | (0.0, 36.9) | (0.0, 6.0) |
|  | [0] | [0] | [0] | [0] | [0] |
| Related^4^ | 0 (0.0%) | 0 (0.0%) | 0 (0.0%) | 0 (0.0%) | 0 (0.0%) |
|  | (0.0, 19.5) | (0.0, 19.5) | (0.0, 18.5) | (0.0, 36.9) | (0.0, 6.0) |
|  | [0] | [0] | [0] | [0] | [0] |
| Adverse Events Leading to Treatment Discontinuation | 2 (11.8%) | 0 (0.0%) | 1 (5.6%) | 0 (0.0%) | 3 (5.0%) |
|  | (1.5, 36.4) | (0.0, 19.5) | (0.1, 27.3) | (0.0, 36.9) | (1.0, 13.9) |
|  | [3] | [0] | [1] | [0] | [4] |
| Any Grade 2 or Higher Laboratory Result^7^ | 3 (17.6%) | 3 (17.6%) | 1 (5.6%) | 0 (0.0%) | 7 (11.7%) |
|  | (3.8, 43.4) | (3.8, 43.4) | (0.1, 27.3) | (0.0, 36.9) | (4.8, 22.6) |
|  | [5] | [4] | [1] | [0] | [10] |
| Any Grade 3 or Higher Laboratory Result^7^ | 1 (5.9%) | 0 (0.0%) | 0 (0.0%) | 0 (0.0%) | 1 (1.7%) |
|  | (0.1, 28.7) | (0.0, 19.5) | (0.0, 18.5) | (0.0, 36.9) | (0.0, 8.9) |
|  | [1] | [0] | [0] | [0] | [1] |

Database lock date: 26SEP2023

N = Number of participants in the safety analysis population within each group and overall

n = Number of participants who experience at least one event (participants with >1 reported event are only counted once in each category).

% = Percentage of participants in each category relative to the total number within each group and overall, i.e., 100 x n/N

All confidence intervals are two-sided 95% via Clopper-Pearson method.

1. Any Adverse Events include both solicited and unsolicited AEs

2. Solicited symptoms are reported from the day of vaccination through 7 days post-vaccination (i.e., 8-day follow-up period).

In the event that the investigator disagrees with the participant's severity assesment, the investigator assessment is reported.

3. Unsolicited AEs are reported from the day of vaccination through 28 days post-vaccination.

4. 'Related' AEs include those reported as possibly, probably, or definitely related to vaccination.

5. SAEs are reported from screening through 24 weeks post final vaccination.

6. MAAEs, and AESIs are reported from the day of the first vaccination through 24 weeks post final vaccination.

7. Chemistry or hematology laboratory results are reported from post-IP administration through the final study visit.

**Table S15. IAVI G002 summary of unsolicited adverse events related to study procedure by MedDRA SOC, PT and treatment group (safety population).**

| **System Organ Class (SOC) /** Preferred Term (PT) | **Overall (N=60 participants)** |
| --- | --- |
|  | **n(%) (95% CI) [events]** |
| **All Systems** | 16 (26.7%) |
|  | (16.1, 39.7) |
|  | [23] |
| **Gastrointestinal disorders** |  |
| **Any Preferred Term** | 4 (6.7%) |
|  | (1.8, 16.2) |
|  | [5] |
| Nausea | 1 (1.7%) |
|  | (0.0, 8.9) |
|  | [1] |
| Paraesthesia oral | 3 (5.0%) |
|  | (1.0, 13.9) |
|  | [3] |
| Vomiting | 1 (1.7%) |
|  | (0.0, 8.9) |
|  | [1] |
| **General disorders and administration site conditions** |  |
| **Any Preferred Term** | 1 (1.7%) |
|  | (0.0, 8.9) |
|  | [1] |
| Axillary pain | 1 (1.7%) |
|  | (0.0, 8.9) |
|  | [1] |
|  | |
| **Investigations** |  |
| **Any Preferred Term** | 2 (3.3%) |
|  | (0.4, 11.5) |
|  | [2] |
| Haematocrit decreased | 1 (1.7%) |
|  | (0.0, 8.9) |
|  | [1] |
| Haemoglobin decreased | 1 (1.7%) |
|  | (0.0, 8.9) |
|  | [1] |
| **Metabolism and nutrition disorders** |  |
| **Any Preferred Term** | 1 (1.7%) |
|  | (0.0, 8.9) |
|  | [1] |
| Iron deficiency | 1 (1.7%) |
|  | (0.0, 8.9) |
|  | [1] |
| **Nervous system disorders** |  |
| **Any Preferred Term** | 3 (5.0%) |
|  | (1.0, 13.9) |
|  | [3] |
| Paraesthesia | 1 (1.7%) |
|  | (0.0, 8.9) |
|  | [1] |
| Presyncope | 2 (3.3%) |
|  | (0.4, 11.5) |
| **Skin and subcutaneous tissue disorders** |  |
| **Any Preferred Term** | 7 (11.7%) |
|  | (4.8, 22.6) |
|  | [9] |
| Ecchymosis | 7 (11.7%) |
|  | (4.8, 22.6) |
|  | [9] |
| **Vascular disorders** |  |
| **Any Preferred Term** | 2 (3.3%) |
|  | (0.4, 11.5) |
|  | [2] |
| Haematoma | 2 (3.3%) |
|  | (0.4, 11.5) |
|  | [2] |
|  | |
| Database lock date: 26SEP2023 N = Number of participants in the safety analysis population within each group and overall n = Number of participants within each SOC and PT % = Percentage of participants with events relative to the total number within each group and overall, i.e., 100 x n/N All confidence intervals are two-sided 95% via Clopper-Pearson method. Unsolicited AEs are reported in this table as per protocol, from the day of vaccination through 28 days post-vaccination. SAEs are reported in this table as per protocol, from screening through 24 weeks post final vaccination. MAAEs, and AESIs are reported in this table as per protocol, from the day of the first vaccination through 24  weeks post final vaccination. | |

**Table S16. IAVI G002 summary of solicited AE duration and days between the most recent vaccination and AE onset**

| **Solicited AE Type** | **Statistics** | **AE Duration (days)** | **Days Between Most Recent Vaccination and AE (days)** |
| --- | --- | --- | --- |
| **Local** | **Max** | 193.0 | 7.0 |
|  | **90% Percentile** | 4.0 | 2.0 |
|  | **75% Percentile** | 3.0 | 1.0 |
|  | **Median** | 2.0 | 1.0 |
|  | **25% Percentile** | 1.0 | 1.0 |
|  | **10% Percentile** | 1.0 | 1.0 |
|  | **Min** | 1.0 | 1.0 |
|  | **# of Events** | 182 | 182 |
| **Systemic** | **Max** | 23.0 | 7.0 |
|  | **90% Percentile** | 4.0 | 2.0 |
|  | **75% Percentile** | 2.0 | 2.0 |
|  | **Median** | 2.0 | 2.0 |
|  | **25% Percentile** | 1.0 | 1.0 |
|  | **10% Percentile** | 1.0 | 1.0 |
|  | **Min** | 1.0 | 1.0 |
|  | **# of Events** | 293 | 293 |
| **All** | **Max** | 193.0 | 7.0 |
|  | **90% Percentile** | 4.0 | 2.0 |
|  | **75% Percentile** | 3.0 | 2.0 |
|  | **Median** | 2.0 | 1.0 |
|  | **25% Percentile** | 1.0 | 1.0 |
|  | **10% Percentile** | 1.0 | 1.0 |
|  | **Min** | 1.0 | 1.0 |
|  | **# of Events** | 475 | 475 |

**Table S17. IAVI G002 detailed summary of solicited AE duration and days between the most recent vaccination and AE onset, with statistics for each type of AE**

| **Solicited AE Type** | **AE Term** | **Statistics** | **AE Duration (days)** | **Days Between Most Recent Vaccination and AE (days)** |
| --- | --- | --- | --- | --- |
| **Local** | **Axillary** **Swelling** | **Max** | 6.0 | 7.0 |
|  |  | **90% Percentile** | 4.0 | 5.0 |
|  |  | **75% Percentile** | 2.0 | 3.0 |
|  |  | **Median** | 1.0 | 2.0 |
|  |  | **25% Percentile** | 1.0 | 1.0 |
|  |  | **10% Percentile** | 1.0 | 1.0 |
|  |  | **Min** | 1.0 | 1.0 |
|  |  | **#** **of** **Events** | 37 | 37 |
|  | **Erythema** **(Redness)** | **Max** | 193.0 | 6.0 |
|  |  | **90% Percentile** | 193.0 | 6.0 |
|  |  | **75% Percentile** | 3.0 | 3.0 |
|  |  | **Median** | 2.0 | 2.0 |
|  |  | **25% Percentile** | 1.0 | 2.0 |
|  |  | **10% Percentile** | 1.0 | 1.0 |
|  |  | **Min** | 1.0 | 1.0 |
|  |  | **#** **of** **Events** | 9 | 9 |
|  | **Induration** **(Hardness)** | **Max** | 3.0 | 2.0 |
|  |  | **90% Percentile** | 3.0 | 2.0 |
|  |  | **75% Percentile** | 2.5 | 2.0 |
|  |  | **Median** | 1.5 | 2.0 |
|  |  | **25% Percentile** | 1.0 | 1.5 |
|  |  | **10% Percentile** | 1.0 | 1.0 |
|  |  | **Min** | 1.0 | 1.0 |
|  |  | **#** **of** **Events** | 8 | 8 |
|  | **Injection** **Site** **Swelling** | **Max** | 6.0 | 2.0 |
|  |  | **90% Percentile** | 4.0 | 2.0 |
|  |  | **75% Percentile** | 3.0 | 2.0 |
|  |  | **Median** | 2.0 | 1.0 |
|  |  | **25% Percentile** | 1.0 | 1.0 |
|  |  | **10% Percentile** | 1.0 | 1.0 |
|  |  | **Min** | 1.0 | 1.0 |
|  |  | **#** **of** **Events** | 14 | 14 |
|  | **Pain** | **Max** | 6.0 | 2.0 |
|  |  | **90% Percentile** | 4.0 | 1.0 |
|  |  | **75% Percentile** | 3.0 | 1.0 |
|  |  | **Median** | 2.0 | 1.0 |
|  |  | **25% Percentile** | 2.0 | 1.0 |
|  |  | **10% Percentile** | 1.0 | 1.0 |
|  |  | **Min** | 1.0 | 1.0 |
|  |  | **#** **of** **Events** | 114 | 114 |
| **Systemic** | **Arthralgia** | **Max** | 5.0 | 2.0 |
|  |  | **90% Percentile** | 3.0 | 2.0 |
|  |  | **75% Percentile** | 2.0 | 2.0 |
|  |  | **Median** | 1.0 | 2.0 |
|  |  | **25% Percentile** | 1.0 | 1.0 |
|  |  | **10% Percentile** | 1.0 | 1.0 |
|  |  | **Min** | 1.0 | 1.0 |
|  |  | **# of Events** | 29 | 29 |
|  | **Chills** | **Max** | 4.0 | 4.0 |
|  |  | **90% Percentile** | 2.0 | 2.0 |
|  |  | **75% Percentile** | 2.0 | 2.0 |
|  |  | **Median** | 1.0 | 2.0 |
|  |  | **25% Percentile** | 1.0 | 1.0 |
|  |  | **10% Percentile** | 1.0 | 1.0 |
|  |  | **Min** | 1.0 | 1.0 |
|  |  | **#** **of** **Events** | 31 | 31 |
|  | **Fatigue** | **Max** | 23.0 | 6.0 |
|  |  | **90% Percentile** | 6.0 | 2.0 |
|  |  | **75% Percentile** | 3.0 | 2.0 |
|  |  | **Median** | 2.0 | 1.0 |
|  |  | **25% Percentile** | 1.0 | 1.0 |
|  |  | **10% Percentile** | 1.0 | 1.0 |
|  |  | **Min** | 1.0 | 1.0 |
|  |  | **#** **of** **Events** | 86 | 86 |
|  | **Fever** | **Max** | 6.0 | 3.0 |
|  |  | **90% Percentile** | 2.0 | 3.0 |
|  |  | **75% Percentile** | 2.0 | 2.0 |
|  |  | **Median** | 1.0 | 2.0 |
|  |  | **25% Percentile** | 1.0 | 1.0 |
|  |  | **10% Percentile** | 1.0 | 1.0 |
|  |  | **Min** | 1.0 | 1.0 |
|  |  | **#** **of** **Events** | 15 | 15 |
|  | **Headache** | **Max** | 6.0 | 7.0 |
|  |  | **90% Percentile** | 4.0 | 3.0 |
|  |  | **75% Percentile** | 3.0 | 2.0 |
|  |  | **Median** | 2.0 | 2.0 |
|  |  | **25% Percentile** | 1.0 | 1.0 |
|  |  | **10% Percentile** | 1.0 | 1.0 |
|  |  | **Min** | 1.0 | 1.0 |
|  |  | **#** **of** **Events** | 63 | 63 |
|  | **Myalgia** | **Max** | 5.0 | 7.0 |
|  |  | **90% Percentile** | 3.0 | 2.0 |
|  |  | **75% Percentile** | 2.0 | 2.0 |
|  |  | **Median** | 1.0 | 1.0 |
|  |  | **25% Percentile** | 1.0 | 1.0 |
|  |  | **10% Percentile** | 1.0 | 1.0 |
|  |  | **Min** | 1.0 | 1.0 |
|  |  | **#** **of** **Events** | 54 | 54 |
|  | **Nausea** | **Max** | 3.0 | 6.0 |
|  |  | **90% Percentile** | 3.0 | 4.0 |
|  |  | **75% Percentile** | 3.0 | 3.0 |
|  |  | **Median** | 1.0 | 2.0 |
|  |  | **25% Percentile** | 1.0 | 2.0 |
|  |  | **10% Percentile** | 1.0 | 1.0 |
|  |  | **Min** | 1.0 | 1.0 |
|  |  | **#** **of** **Events** | 15 | 15 |
| **All** | | **Max** | 193.0 | 7.0 |
|  |  | **90% Percentile** | 4.0 | 2.0 |
|  |  | **75% Percentile** | 3.0 | 2.0 |
|  |  | **Median** | 2.0 | 1.0 |
|  |  | **25% Percentile** | 1.0 | 1.0 |
|  |  | **10% Percentile** | 1.0 | 1.0 |
|  |  | **Min** | 1.0 | 1.0 |
|  |  | **#** **of** **Events** | 475 | 475 |

**Table S18. IAVI G002 skin adverse events assessed as IP-related.**

| **Ppt** | **Reaction PT/PTs** | **Gp** | **Most Recent IP** | **Time to Onset (days post-IP)** | **Duration (max days)** | **Grade**  **(max severity)** | **Medical History** | **COVID Vaccination prior to trial** | **COVID Vaccination while on study** | **Outcome** |
| --- | --- | --- | --- | --- | --- | --- | --- | --- | --- | --- |
| G002-254 | Pruritus | 1 | 1 (eOD) | 40 | 165 | 1 | Seasonal allergies, Eczema, COVID-19 infection | 1x J&J,  1x Moderna | 1 x Pfizer  (25 days post AE onset) | Resolved |
| G002-479 | Urticaria &  Dermatographism | 1 | 1 (eOD) | 19 | 57 | 2 | Eczema,  Depression,  Anxiety,  ADHD,  COVID-19 infection,  Dysmenorrhea | 3x Moderna | N/A | Resolved |
| G002-516 | Urticaria & Dermatographism | 1 | 2 (eOD) | 10 | 151* | 2 | Seasonal allergies | 2x Moderna,  1x Pfizer | N/A | Resolved |
| G002-758 | Urticaria &  Dermatographism | 1 | 1 (eOD) | 27 | 84 | 3*** | IBS, Hyperlipidemia, Anxiety, Depression | 3x Moderna | 1 x Moderna  (57 days post AE onset) | Resolved |
| G002-834 | Pruritus &  Dermatographism | 1 | 2 (eOD) | 12 | 107 | 1 | Acne, Exercise-induced asthma | 2x Pfizer,  1x Moderna | 1 x Moderna  (53 days post AE onset) | Resolved |
| G002-969 | Urticaria & Pruritus | 1 | 2 (eOD) | 12 | 69 | 2 | Seasonal allergies, Eczema | 3x Pfizer; 1x Moderna | N/A | Resolved |
| G002-577 | Pruritus | 2 | 2 (core) | 13 | 27 | 2 | Environmental allergy, Anxiety, Depression | 2x Pfizer,  1x Moderna | 1 x Moderna (18 days post AE onset) | Resolved |
| G002-943 | Pruritus | 2 | 1 (eOD) | 39 | 68 | 2 | Acne, Seasonal allergies, Anxiety, Eczema | 2x Moderna,  1x Pfizer | 1 x Pfizer  (post AE resolution) | Resolved |
| G002-462 | Pruritus | 3 | 1 (eOD) | 38 | 234** | 2 | Anxiety, Vitamin D deficiency | 2x Moderna,  1x Pfizer | 1 x Pfizer  (prior to AE) | Resolved |
| G002-462 | Urticaria &  Dermatographism | 3 | 2 (eOD) | 7 | 70 | 2 | Anxiety, Vitamin D deficiency | 2x Moderna,  1x Pfizer | 1 x Pfizer  (prior to AE) | Resolved |
| G002-595 | Pruritus | 3 | 3 (core) | 28 | 48 | 1 | Anxiety, HPV infection | 2x Astrazeneca,  1x Pfizer | 1 x Pfizer  (prior to AE) | Resolved |
| G002-595 | Dermatographism | 3 | 3 (core) | 36 | 1 | 1 | Anxiety, HPV infection | 2x Astrazeneca,  1x Pfizer | 1 x Pfizer  (prior to AE) | Resolved |
| G002-632 | Pruritus | 3 | 1 (eOD) | 20 | 22 | 1 | ADHD, GERD, Scalp rosacea, Acne, Anxiety | 2x Moderna,  1x Pfizer | 1 x Pfizer  (10 days post AE onset) | Resolved |
| G002-632 | Pruritus | 3 | 2 (eOD) | 9 | 66 | 1 | ADHD, GERD, Scalp rosacea, Acne, Anxiety | 2x Moderna,  1x Pfizer | 1 x Pfizer  (prior to AE) | Resolved |

*AE ended after final study visit. Duration was 151 days while on study; total duration of event until resolution was 369 days.

**AE ended after final study visit. Duration was 234 days while on study; total duration of event until resolution was 295 days.

***AE lasted 4 days at a Grade 3, after which it was reduced to Grade 2 severity.

**Table S19. IAVI G002 frequencies of skin adverse events by dose number and study group.**

| **Group** | **Dose 1** | **# of events** | **n/N (%)**  **[95% CI]** | **Dose 2** | **# of events** | **n/N (%)**  **[95% CI]** | **Dose 3** | **# of events** | **n/N (%)**  **[95% CI]** |
| --- | --- | --- | --- | --- | --- | --- | --- | --- | --- |
| Group 1* (N=17) | eOD | 3 | 3/17 (17.6%)  [3.8%-43.4%] | eOD | 3 | 3/14 (21.4%)  [4.7%-50.8%] | - | - | - |
| Group 2 (N=17) | eOD | 1 | 1/17 (5.9%) [0.1%-28.7%] | Core | 1 | 1/17 (5.9%) [0.1%-28.7%] | - | - | - |
| Group 3* (N=18) | eOD | 2 | 2/18 (11.1.%) [1.4%-34.7%] | eOD | 2 | 2/18 (11.1.%) [1.4%-34.7%] | Core | 2 | 1/13 (0.2%) [0.2-36.0%] |
| Group 4 (N=8) | Core | 0 | 0/8 (0.0%)  [ 0.0%-36.9%] | - | - | - | - | - | - |

*Study groups with discontinued IP administrations

n = number of participants who experienced skin adverse events

N = number of participants who received vaccine

**Table S20. IAVI G002 demographics by skin reaction Case Definition 1 (related urticaria or pruritus AEs).**

| **Participant Characteristics** | **Urticaria /Pruritus** | **No Urticaria /Pruritus** | **Overall** |  |
| --- | --- | --- | --- | --- |
|  |  |  |  |  |
|  | **N=11** | **N=49** | **N=60** |  |
| **Age (years)** |  |  |  |  |
| n | 11 | 49 | 60 |  |
| Mean (SD) | 27.3 (3.43) | 29.2 (6.58) | 28.8 (6.15) |  |
| Median | 27.5 | 28.2 | 27.9 |  |
| Q1, Q3 | 24.9, 30.5 | 23.9, 32.6 | 23.9, 31.8 |  |
| Min, Max | 21.2, 32.1 | 20.0, 46.5 | 20.0, 46.5 |  |
| **Sex at Birth, n (%)** |  |  |  |  |
| Female | 6 (54.5%) | 28 (57.1%) | 34 (56.7%) |  |
| Male | 5 (45.5%) | 21 (42.9%) | 26 (43.3%) |  |
| **Gender, n (%)** |  |  |  |  |
| Male | 5 (45.5%) | 18 (36.7%) | 23 (38.3%) |  |
| Female | 4 (36.4%) | 25 (51.0%) | 29 (48.3%) |  |
| Transgender male (female to male) | 1 (9.1%) | 3 (6.1%) | 4 (6.7%) |  |
| Transgender female (male to female) | 0 (0.0%) | 2 (4.1%) | 2 (3.3%) |  |
| Gender queer | 0 (0.0%) | 0 (0.0%) | 0 (0.0%) |  |
| Gender variant or gender non-conforming | 1 (9.1%) | 1 (2.0%) | 2 (3.3%) |  |
| Self-identify | 0 (0.0%) | 0 (0.0%) | 0 (0.0%) |  |
| Prefer not to answer | 0 (0.0%) | 0 (0.0%) | 0 (0.0%) |  |
| **Ethnicity, n (%)** |  |  |  |  |
| Hispanic or Latino | 1 (9.1%) | 11 (22.4%) | 12 (20.0%) |  |
| Not Hispanic or Latino | 10 (90.9%) | 38 (77.6%) | 48 (80.0%) |  |
| Not reported | 0 (0.0%) | 0 (0.0%) | 0 (0.0%) |  |
| Unknown | 0 (0.0%) | 0 (0.0%) | 0 (0.0%) |  |
| **Race, n (%)** |  |  |  |  |
| American Indian or Alaskan Native | 0 (0.0%) | 0 (0.0%) | 1 (1.7%) |  |
| Asian | 0 (0.0%) | 5 (10.2%) | 5 (8.3%) |  |
| Black or African American | 0 (0.0%) | 1 (2.0%) | 1 (1.7%) |  |
| Native Hawaiian or Other Pacific Islander | 0 (0.0%) | 1 (2.0%) | 1 (1.7%) |  |
| White | 9 (81.8%) | 35 (71.4%) | 44 (73.3%) |  |
| Multiracial | 1 (9.1%) | 7 (14.3%) | 8 (13.3%) |  |
| Unknown | 0 (0.0%) | 0 (0.0%) | 0 (0.0%) |  |
| Other | 0 (0.0%) | 0 (0.0%) | 0 (0.0%) |  |
| **Height (cm)** |  |  |  |  |
| n | 11 | 49 | 60 |  |
| Mean (SD) | 171.8 (8.02) | 172.3 (9.10) | 172.2 (8.85) |  |
| Median | 170 | 171 | 171 |  |
| Q1, Q3 | 164.0, 179.0 | 168.0, 179.0 | 166.5, 179.0 |  |
| Min, Max | 161.0, 183.0 | 154.0, 191.0 | 154.0, 191.0 |  |
| **Weight (kg)** |  |  |  |  |
| n | 11 | 49 | 60 |  |
| Mean (SD) | 73.5 (12.29) | 74.9 (15.51) | 74.6 (14.88) |  |
| Median | 71 | 74 | 73.5 |  |
| Q1, Q3 | 64.0, 86.0 | 65.0, 80.0 | 64.5, 83.0 |  |
| Min, Max | 54.0, 89.0 | 52.0, 124.0 | 52.0, 124.0 |  |
| **BMI** |  |  |  |  |
| n | 11 | 49 | 60 |  |
| Mean (SD) | 24.8 (3.39) | 25.1 (4.25) | 25.1 (4.08) |  |
| Median | 24.7 | 24.5 | 24.5 |  |
| Q1, Q3 | 21.3, 27.2 | 22.0, 27.1 | 21.9, 27.1 |  |
| Min, Max | 20.8, 30.4 | 18.2, 34.7 | 18.2, 34.7 |  |

**Table S21. IAVI G002 medical history and antihistamine use at baseline by skin reaction Case Definition 1 (related urticaria or pruritus AEs).**

| **History of:** | | | **Urticaria^1^ /Pruritus^2^** | **No Urticaria / Pruritus** | **Overall** |
| --- | --- | --- | --- | --- | --- |
|  | | | **(N=11)** | **(N=49)** | **(N=60)** |
|  | | | **n (%)**  **[95% CI]** | **n (%)**  **[95% CI]** | **n (%)**  **[95% CI]** |
| COVID-19 Infection | | | 2 (18.2%)  [2.3% – 51.8%] | 4 (8.2%)  [2.3% - 19.6%] | 6 (10.0%) |
| Medical History Events of Interest (if any): | | | 6 (54.5%)  [23.4% – 83.3%] | 23 (46.9%)  [32.5% – 61.7%] | 29 (48.3%)  [35.2% – 61.6%] |
| History of Eczema | | | 4 (36.4%)  [10.9% - 69.2%] | 4 (8.2%)  [2.3% - 19.6%] | 8 (13.3%)  [5.9% - 24.6%] |
| Seasonal and Environmental Allergies | | | 5 (45.5%)  [16.7% - 76.6%] | 20 (40.8%)  [27.0% - 55.8%] | 25 (41.7%)  [24.6% - 50.1%] |
| History of Food Allergies | | | 1 (9.1%)  [0.2% – 41.3%] | 1 (2.0%)  [0.05% –10.9%] | 2 (3.3%)  [0.4% – 11.5%] |
| History of Drug Hypersensitivity | | | 0 (0.0%)  [0.0% - 28.5%] | 2 (4.1%)  [0.5%– 14.0%] | 2 (3.3%)  [0.4% – 11.5%] |
| History of Other and Non-Specified Allergies | | | 0 (0.0%)  [0.0% - 28.5%] | 1 (2.0%)  [0.1% - 10.9%] | 0 (0.0%)  [0.0% - 6.0%] |
| History of Asthma | | | 0 (0.0%)  [0.0% - 28.5 %] | 5 (10.2%)  [3.4% – 22.2%] | 5 (8.3%)  [2.8% – 18.4%] |
| History of Urticaria | | | 1 (9.1%)  [0.2% – 41.3%] | 3 (6.1%)  [1.3% – 16.9%] | 4 (6.7%)  [1.8% – 16.2%] |
| History of Anxiety | | | 7 (63.6%)  [30.8% - 89.1%] | 10 (20.4%)  [10.2% - 34.3%] | 17 (28.3%)  [17.5% - 41.4%] |
| Baseline Antihistamine, Asthma Inhaler, or Steroid Medications Use | | | 4 (36.4%)  [10.9% - 69.2%] | 12 (24.5%)  [13.3% – 38.9%] | 16 (26.7%)  [16.1% – 39.7%] |
| mRNA-LNP COVID-19 Vaccination | | | 11 (100%)  [71.5% - 100%] | 49 (100%)  [92.7% - 100%] | 60 (100%)  [94.0% - 100%] |
| Types of all COVID-19 vaccinations prior to enrollment | Moderna Only | | 2 (18.2%)  [2.3% - 51.8%] | 12 (24.5%) [13.3% - 38.9%] | 14 (23.3%) [13.4% - 36.0%] |
|  | Moderna and Johnson & Johnson | | 1 (9.0%)  [0.2% - 41.3%] | 2 (4.1%)  [0.4% - 14.0%] | 3 (5.0%)  [1.0% - 13.9%] |
|  | Pfizer Only | | 0 (0.0%)  [0.0% - 28.5%] | 22 (44.9%) [30.7% - 59.8%] | 22 (36.7%) [24.6% - 50.1%] |
|  | Pfizer and Johnson & Johnson | | 0 (0.0%)  [0.0% - 28.5%] | 3 (6.1%)  [1.3% - 16.9%] | 3 (5.0%)  [1.0% - 13.9%] |
|  | Pfizer and Moderna | | 7 (63.6%)  [30.8% - 89.1%] | 9 (19.4%)  [8.8% - 32.0%] | 16 (26.7%) [16.1% - 39.7%] |
|  | Pfizer and Other | | 1 (9.0%)  [0.2% - 41.3%] | 1 (2.0%)  [0.1% - 10.9%] | 2 (3.3%)  [0.4% - 11.5%] |
| Type of the last COVID-19 vaccination prior to enrollment | | Moderna | 6 (54.6%) [23.4% - 83.3%] | 21 (42.9%)  [28.8% - 57.8%] | 27 (45.0%) [32.1% - 58.4%] |
|  |  | Pfizer | 5 (45.5%) [16.7% - 76.6%] | 28 (57.1%)  [42.2% - 71.2%] | 33 (55.0%) [41.6% - 67.9%] |
| Ever received COVID-19 vaccination prior to enrollment | | Moderna | 10 (90.9%) [58.7% - 99.8%] | 23 (46.9%)  [32.5% - 61.7%] | 33 (55.0%)  [41.6% - 67.9%] |
|  |  | Pfizer | 8 (72.7%)  [39.0% - 94.0%] | 35 (71.4%)  [56.7% - 83.4%] | 43 (71.7%)  [58.6% - 82.5%] |

^1^Reported urticaria includes the following terms: mechanical urticaria or dermatographism.

^2^Reported pruritus includes both rash pruritic and pruritus.

**Table S22. Summary of adverse events after eOD-GT8 60mer mRNA-LNP vaccination in IAVI G003 (African populations) and IAVI G002 (North American populations).**

| Adverse Events  /Severity  /Relationship to IP Administration | **G003** | | | | **G002 Group 1** | | | |
| --- | --- | --- | --- | --- | --- | --- | --- | --- |
|  | **[N=18]** | | | | **[N=17]** | | | |
|  | **n** | **(%)** | **E** | **95 % CI** | **n** | **(%)** | **E** | **95 % CI** |
| Any Adverse Events^1^ | 18 | (100) | 105 | (81.5, 100) | 17 | (100) | 172 | (80.5, 100) |
| Any Solicited Adverse Events^2^ | 18 | (100) | 91 | (81.5, 100) | 17 | (100) | 124 | (80.5, 100) |
| Local Solicited Adverse Events | 18 | (100) | 44 | (81.5, 100) | 17 | (100) | 45 | (80.5, 100) |
| Grade 3 or Higher^3^ | 0 | (0.0) | 0 | (0.0, 18.5) | 0 | (0.0) | 0 | (0.0, 19.5) |
| Systemic Solicited Adverse Events | 14 | (77.8) | 47 | (81.5, 100) | 17 | (100) | 79 | (80.5, 100) |
| Grade 3 or Higher^3^ | 1 | (5.6) | 1 | (0.1, 27.3) | 4 | (23.5) | 8 | (6.8, 49.9) |
| Any Unsolicited Adverse Events^4^ | 8 | (44.4) | 14 | (21.5, 69.2) | 14 | (82.4) | 48 | (56.6, 96.2) |
| Grade 1 or 2^3^ | 8 | (44.4) | 14 | (21.5, 69.2) | 12 | (70.6) | 46 | (44.0, 89.7) |
| Grade 3 or Higher^3^ | 0 | (0.0) | 0 | (0.0, 18.5) | 2 | (11.8) | 2 | (1.5, 36.4) |
| Related^5^ to IP | 3 | (16.7) | 3 | (3.6, 41.4) | 6 | (35.3) | 12 | (14.2,61.7) |
| Related^5^ and Grade 3 or Higher | 0 | (0.0) | 0 | (0.0, 18.5) | 1 | (5.9) | 1 | (0.1, 28.7) |
| Serious Adverse Events (SAEs)^6^ | 0 | (0.0) | 0 | (0.0, 18.5) | 0 | (0.0) | 0 | (0.0, 19.5) |
| Medically Attended AEs (MAAEs)^7^ | 8 | (44.4) | 10 | (21.5, 69.2) | 11 | (64.7) | 21 | (38.8, 85.8) |
| MAAEs^7^ Related^5^ | 1 | (5.6) | 1 | (0.1, 27.3) | 2 | (11.8) | 4 | (1.5, 36.4) |
| Adverse Events of Special Interest (AESI)^7^ | 0 | (0.0) | 0 | (0.0, 18.5) | 0 | (0.0) | 0 | (0.0, 19.5) |
| AEs leading to treatment discontinuation | 0 | (0.0) | 0 | (0.0, 18.5) | 2 | (11.8) | 3 | (1.5, 36.4) |
| Any Grade 2 or Higher^3^ Laboratory Result^8^ | 2 | (11.2) | 5 | (1.4, 34.7) | 3 | (17.6) | 5 | (3.8, 43.4) |
| Any Grade 3 or Higher^3^ Laboratory Result^8^ | 0 | (0.0) | 0 | (0.0, 18.5) | 1 | (5.9) | 1 | (0.1, 28.7) |

N = number of participants; n = Number of participants who experienced at least one event (participants with >1 reported events are only counted once in each category); % = Percentage of participants in each category relative to the total number, i.e., 100 x n/N E = number of events; CI = Confidence Interval , all confidence intervals are two-sided, 95% via the Clopper-Pearson method.

1. Any Adverse Events include both solicited and unsolicited AEs

2. Solicited symptoms are reported from the day of vaccination through 7 days post-vaccination (i.e., 8-day follow-up period).

In the event that the investigator disagrees with the participant's severity assessment, the investigator's assessment is reported.

3. In accordance with the DAIDS Table for Grading the Severity of Adult and Pediatric Adverse Events, Version 2.1, July 2017

4. Unsolicited AEs are reported from the day of vaccination through 28 days post-vaccination.

5. 'Related' AEs include those reported as possibly, probably, or definitely related to vaccination.

6. SAEs are reported from screening through 24 weeks post final vaccination.

7. MAAEs, and AESIs are reported from the day of the first vaccination through 24 weeks post final vaccination.

8. Chemistry or hematology laboratory results are reported from post-IP administration through the final study visit.

**Table S23. Solicited adverse event duration and days between the most recent vaccination and adverse event onset, after eOD-GT8 60mer mRNA-LNP vaccination in IAVI G003 (African populations) and IAVI G002 (North American populations).**

**
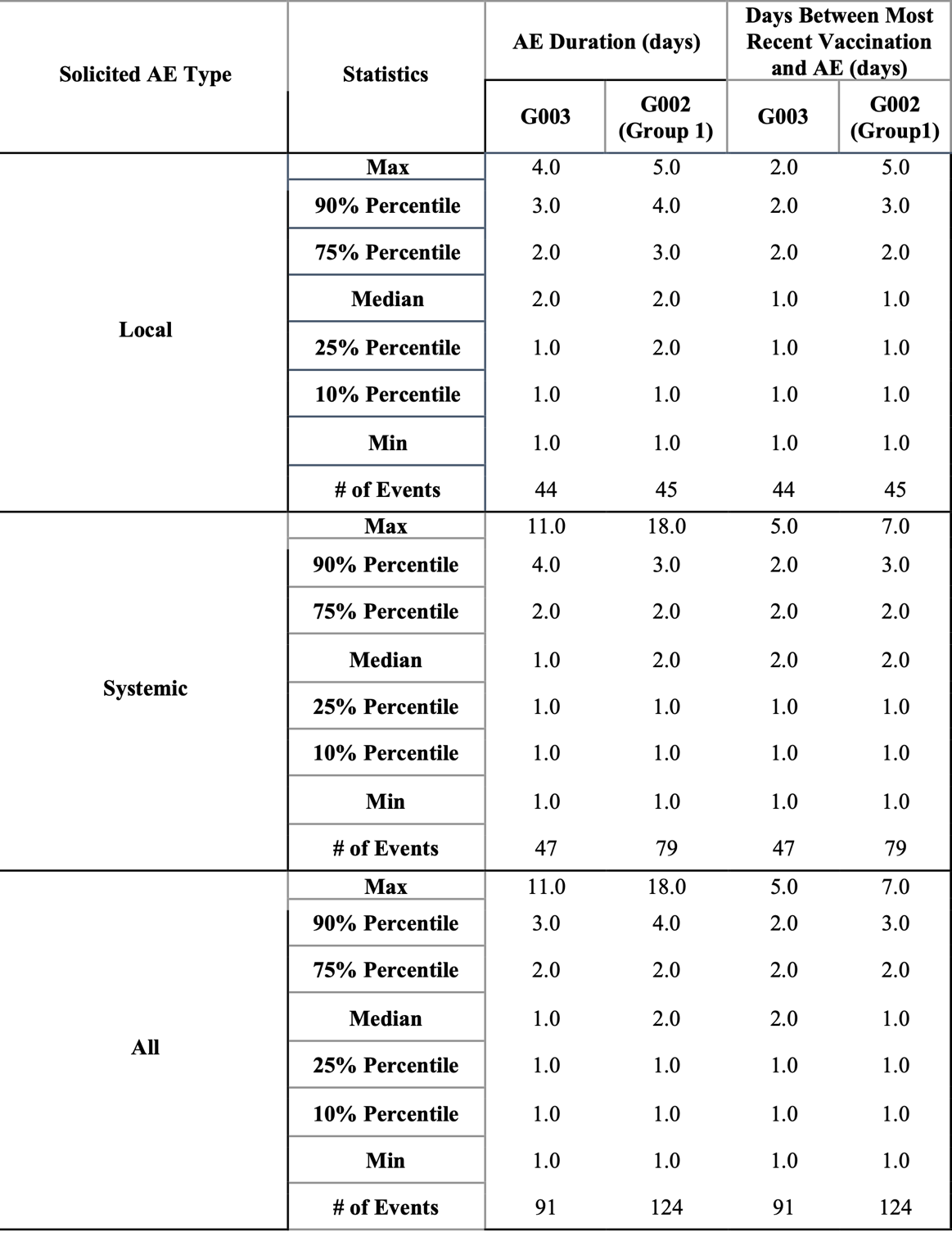
**

**Table S24. Detailed summary of solicited adverse event duration and days between the most recent vaccination and adverse event onset, after eOD-GT8 60mer mRNA-LNP vaccination in IAVI G003 (African populations) and IAVI G002 (North American populations).**

| **Solicited AE Type** | **AE Term** | **Statistics** | **AE Duration (days)** | | **Days Between Most Recent Vaccination and AE (days)** | | |
| --- | --- | --- | --- | --- | --- | --- | --- |
|  |  |  | **G003** | **G002**  **(Group 1)** | **G003** | | **G002 (Group1)** |
| **Local** | **Axillary** **Swelling** | **Max** | 3.0 | 4.0 | 2.0 | 5.0 | |
|  |  | **90% Percentile** | 3.0 | 4.0 | 2.0 | 5.0 | |
|  |  | **75% Percentile** | 2.5 | 3.0 | 2.0 | 3.0 | |
|  |  | **Median** | 1.5 | 1.5 | 2.0 | 2.5 | |
|  |  | **25% Percentile** | 1.0 | 1.0 | 1.0 | 1.0 | |
|  |  | **10% Percentile** | 1.0 | 1.0 | 1.0 | 1.0 | |
|  |  | **Min** | 1.0 | 1.0 | 1.0 | 1.0 | |
|  |  | **#** **of** **Events** | 12 | 10 | 12 | 10 | |
|  | **Erythema** **(Redness)** | **Max** | . | 2.0 | . | 3.0 | |
|  |  | **90% Percentile** | . | 2.0 | . | 3.0 | |
|  |  | **75% Percentile** | . | 2.0 | . | 3.0 | |
|  |  | **Median** | . | 1.0 | . | 2.0 | |
|  |  | **25% Percentile** | . | 1.0 | . | 2.0 | |
|  |  | **10% Percentile** | . | 1.0 | . | 2.0 | |
|  |  | **Min** | . | 1.0 | . | 2.0 | |
|  |  | **#** **of** **Events** | . | 3 | . | 3 | |
|  | **Induration** **(Hardness)** | **Max** | 1.0 | 2.0 | 2.0 | 2.0 | |
|  |  | **90% Percentile** | 1.0 | 2.0 | 2.0 | 2.0 | |
|  |  | **75% Percentile** | 1.0 | 2.0 | 2.0 | 2.0 | |
|  |  | **Median** | 1.0 | 2.0 | 2.0 | 2.0 | |
|  |  | **25% Percentile** | 1.0 | 2.0 | 2.0 | 2.0 | |
|  |  | **10% Percentile** | 1.0 | 2.0 | 2.0 | 2.0 | |
|  |  | **Min** | 1.0 | 2.0 | 2.0 | 2.0 | |
|  |  | **#** **of** **Events** | 1 | 1 | 1 | 1 | |
|  | **Injection** **Site** **Swelling** | **Max** | 1.0 | 2.0 | 2.0 | 2.0 | |
|  |  | **90% Percentile** | 1.0 | 2.0 | 2.0 | 2.0 | |
|  |  | **75% Percentile** | 1.0 | 2.0 | 2.0 | 2.0 | |
|  |  | **Median** | 1.0 | 1.5 | 2.0 | 1.5 | |
|  |  | **25% Percentile** | 1.0 | 1.0 | 2.0 | 1.0 | |
|  |  | **10% Percentile** | 1.0 | 1.0 | 2.0 | 1.0 | |
|  |  | **Min** | 1.0 | 1.0 | 2.0 | 1.0 | |
|  |  | **#** **of** **Events** | 2 | 2 | 2 | 2 | |
|  | **Pain** | **Max** | 4.0 | 5.0 | 2.0 | 2.0 | |
|  |  | **90% Percentile** | 3.0 | 4.0 | 2.0 | 1.0 | |
|  |  | **75% Percentile** | 2.0 | 3.0 | 2.0 | 1.0 | |
|  |  | **Median** | 2.0 | 2.0 | 1.0 | 1.0 | |
|  |  | **25% Percentile** | 1.0 | 2.0 | 1.0 | 1.0 | |
|  |  | **10% Percentile** | 1.0 | 2.0 | 1.0 | 1.0 | |
|  |  | **Min** | 1.0 | 1.0 | 1.0 | 1.0 | |
|  |  | **#** **of** **Events** | 29 | 29 | 29 | 29 | |
| **Systemic** | **Arthralgia** | **Max** | 3.0 | 2.0 | 2.0 | 2.0 | |
|  |  | **90% Percentile** | 3.0 | 2.0 | 2.0 | 2.0 | |
|  |  | **75% Percentile** | 1.0 | 2.0 | 2.0 | 2.0 | |
|  |  | **Median** | 1.0 | 1.0 | 2.0 | 2.0 | |
|  |  | **25% Percentile** | 1.0 | 1.0 | 2.0 | 1.5 | |
|  |  | **10% Percentile** | 1.0 | 1.0 | 1.0 | 1.0 | |
|  |  | **Min** | 1.0 | 1.0 | 1.0 | 1.0 | |
|  |  | **# of Events** | 5 | 8 | 5 | 8 | |
|  | **Chills** | **Max** | 3.0 | 2.0 | 5.0 | 4.0 | |
|  |  | **90% Percentile** | 3.0 | 2.0 | 5.0 | 4.0 | |
|  |  | **75% Percentile** | 3.0 | 1.5 | 5.0 | 2.0 | |
|  |  | **Median** | 1.0 | 1.0 | 2.0 | 2.0 | |
|  |  | **25% Percentile** | 1.0 | 1.0 | 2.0 | 1.5 | |
|  |  | **10% Percentile** | 1.0 | 1.0 | 2.0 | 1.0 | |
|  |  | **Min** | 1.0 | 1.0 | 2.0 | 1.0 | |
|  |  | **#** **of** **Events** | 3 | 8 | 3 | 8 | |
|  | **Fatigue** | **Max** | 11.0 | 18.0 | 2.0 | 2.0 | |
|  |  | **90% Percentile** | 6.0 | 4.0 | 2.0 | 2.0 | |
|  |  | **75% Percentile** | 2.0 | 2.0 | 2.0 | 2.0 | |
|  |  | **Median** | 1.0 | 2.0 | 2.0 | 1.0 | |
|  |  | **25% Percentile** | 1.0 | 1.0 | 1.0 | 1.0 | |
|  |  | **10% Percentile** | 1.0 | 1.0 | 1.0 | 1.0 | |
|  |  | **Min** | 1.0 | 1.0 | 1.0 | 1.0 | |
|  |  | **#** **of** **Events** | 13 | 24 | 13 | 24 | |
|  | **Fever** | **Max** | 1.0 | 2.0 | 2.0 | 2.0 | |
|  |  | **90% Percentile** | 1.0 | 2.0 | 2.0 | 2.0 | |
|  |  | **75% Percentile** | 1.0 | 2.0 | 2.0 | 2.0 | |
|  |  | **Median** | 1.0 | 1.0 | 2.0 | 2.0 | |
|  |  | **25% Percentile** | 1.0 | 1.0 | 2.0 | 1.0 | |
|  |  | **10% Percentile** | 1.0 | 1.0 | 2.0 | 1.0 | |
|  |  | **Min** | 1.0 | 1.0 | 2.0 | 1.0 | |
|  |  | **#** **of** **Events** | 2 | 5 | 2 | 5 | |
|  | **Headache** | **Max** | 4.0 | 4.0 | 3.0 | 7.0 | |
|  |  | **90% Percentile** | 4.0 | 4.0 | 3.0 | 4.0 | |
|  |  | **75% Percentile** | 2.0 | 2.5 | 2.0 | 2.5 | |
|  |  | **Median** | 1.0 | 1.5 | 2.0 | 2.0 | |
|  |  | **25% Percentile** | 1.0 | 1.0 | 1.0 | 1.5 | |
|  |  | **10% Percentile** | 1.0 | 1.0 | 1.0 | 1.0 | |
|  |  | **Min** | 1.0 | 1.0 | 1.0 | 1.0 | |
|  |  | **#** **of** **Events** | 19 | 16 | 19 | 16 | |
|  | **Myalgia** | **Max** | 6.0 | 3.0 | 2.0 | 7.0 | |
|  |  | **90% Percentile** | 6.0 | 2.0 | 2.0 | 2.0 | |
|  |  | **75% Percentile** | 5.0 | 2.0 | 2.0 | 2.0 | |
|  |  | **Median** | 2.5 | 2.0 | 2.0 | 1.0 | |
|  |  | **25% Percentile** | 1.0 | 1.0 | 1.5 | 1.0 | |
|  |  | **10% Percentile** | 1.0 | 1.0 | 1.0 | 1.0 | |
|  |  | **Min** | 1.0 | 1.0 | 1.0 | 1.0 | |
|  |  | **#** **of** **Events** | 4 | 15 | 4 | 15 | |
|  | **Nausea** | **Max** | 2.0 | 2.0 | 1.0 | 3.0 | |
|  |  | **90% Percentile** | 2.0 | 2.0 | 1.0 | 3.0 | |
|  |  | **75% Percentile** | 2.0 | 2.0 | 1.0 | 3.0 | |
|  |  | **Median** | 2.0 | 2.0 | 1.0 | 3.0 | |
|  |  | **25% Percentile** | 2.0 | 1.0 | 1.0 | 2.0 | |
|  |  | **10% Percentile** | 2.0 | 1.0 | 1.0 | 2.0 | |
|  |  | **Min** | 2.0 | 1.0 | 1.0 | 2.0 | |
|  |  | **#** **of** **Events** | 1 | 3 | 1 | 3 | |
| **All** | | **Max** | 11.0 | 18.0 | 5.0 | 7.0 | |
|  |  | **90% Percentile** | 3.0 | 4.0 | 2.0 | 3.0 | |
|  |  | **75% Percentile** | 2.0 | 2.0 | 2.0 | 2.0 | |
|  |  | **Median** | 1.0 | 2.0 | 2.0 | 1.0 | |
|  |  | **25% Percentile** | 1.0 | 1.0 | 1.0 | 1.0 | |
|  |  | **10% Percentile** | 1.0 | 1.0 | 1.0 | 1.0 | |
|  |  | **Min** | 1.0 | 1.0 | 1.0 | 1.0 | |
|  |  | **#** **of** **Events** | 91 | 124 | 91 | 124 | |

**Table S25. Moderna primary vaccination studies pooled for urticaria analysis.** This is the first of two sets of pooled studies.

| Study (mRNA vaccine) | mRNA (n) | Non-mRNA  control (n) |
| --- | --- | --- |
| mRNA-1010 (influenza) P101 | 728 | 150 (Placebo/Afluria™) |
| mRNA-1010 (influenza) P301 | 3035 | 3048 (Fluarix™) |
| mRNA-1010 (influenza) P302 | 11210 | 11200 (Fluarix™) |
| mRNA-1011 (influenza) P101 | 695 | 0 |
| mRNA-1020 (influenza) P101 | 494 | 71 (Flublok™) |
| mRNA-1073 (influenza/COVID-19) P101 | 547 | 0 |
| mRNA-1083 (influenza/COVID-19) P101 | 843 | 159 (Fluarix™/Fluzone™) |
| mRNA-1083 (influenza/COVID-19) P301 | 4004 | 0 |
| mRNA-1273 (COVID-19) P201 Part A | 400 | 200 (Placebo) |
| mRNA-1273 (COVID-19) P203 Part 1A | 2486 | 1240 (Placebo) |
| mRNA-1273 (COVID-19) P204 Part 1 (open label phase) | 1125 | 0 |
| mRNA-1273 (COVID-19) P204 Part 2 (blinded phase) | 8031 | 2669 (Placebo) |
| mRNA-1273 (COVID-19) P301 Part A | 15184 | 15162 (Placebo) |
| mRNA-1273 (COVID-19) P304 Part A | 152 | 0 |
| mRNA-1283 (COVID-19) P101 | 104 | 0 |
| mRNA-1345 (RSV) P101 | 339 | 83 (Placebo) |
| mRNA-1345 (RSV) P301 | 18245 | 18184 (Placebo) |
| mRNA-1647-1443 (CMV) P101 Phase A | 24 | 3 (Placebo) |
| mRNA-1647-1443 (CMV) P101 Phase B | 89 | 30 (Placebo) |
| mRNA-1647-1443 (CMV) P101 Phase C | 29 | 6 (Placebo) |
| mRNA-1647 CMV) P103 | 8 | 1 (Placebo) |
| mRNA-1647 (CMV) P202 | 235 | 80 (Placebo) |
| mRNA-1653 (hMPV/PIV3) P101 | 94 | 30 (Placebo) |
| mRNA-1653 (hMPV/PIV3) P102 Adult Cohort | 16 | 8 (Placebo) |
| mRNA-1653 (hMPV/PIV3) P102 Pediatric Cohort | 17 | 9 (Placebo) |
| mRNA-1893 (Zika) P101 | 96 | 24 (Placebo) |
| mRNA-1893 (Zika) P201 | 605 | 198 (Placebo) |
| mRNA-CRID (influenza) 004 | 270 | 0 |
| Total | 69105 | 52555 |

**Table S26. Moderna mRNA-1273 booster vaccination studies pooled for urticaria analysis.** This is the second of two sets of pooled studies.

| Study | mRNA (n) |
| --- | --- |
| mRNA-1083 (influenza/COVID-19) P101 Part 1 | 214 |
| mRNA-1083 (influenza/COVID-19) P301 | 4011 |
| mRNA-1273 (COVID-19) P201 Part B | 344 |
| mRNA-1273 (COVID-19) P201 Part C | 60 |
| mRNA-1273 (COVID-19) P203 Part 1C | 1405 |
| mRNA-1273 (COVID-19) P205 Part (A1,B,C,D,E,F) | 3450 |
| mRNA-1273 (COVID-19) P205 Part A2 | 135 |
| mRNA-1273 (COVID-19) P205 Part (F2,G,H) | 1698 |
| mRNA-1273 (COVID-19) P205 Part J | 100 |
| mRNA-1273 (COVID-19) P301 Part C | 19609 |
| mRNA-1273 (COVID-19) P304 Part B | 169 |
| mRNA-1273 (COVID-19) P305 Part 1 | 724 |
| mRNA-1273 (COVID-19) P305 Part 2 | 2824 |
| mRNA-1273 (COVID-19) P306 Part 2 | 539 |
| mRNA-1283 (COVID-19) P201 | 540 |
| mRNA-1283 (COVID-19) P301 | 11417 |
| Total | 47239 |

**Table S27. Adverse Event search criteria for urticaria or angioedema (MedDRA Version 26.1) used in the analysis of Moderna mRNA studies.**

| MedDRA Terms |
| --- |
| Preferred Terms (PT)  Angioedema  Idiopathic angioedema  Idiopathic histaminergic angioedema  Intestinal angioedema  High Level Term (HLT)  Urticaria |

**Table S28. Rate of urticaria or angioedema across Moderna interventional mRNA studies.** The rates reported here were computed from the pooled studies in tables S25 and S26 using the search criteria in table S27. Adverse events were included regardless of their relationship to study vaccination.

| Pooled Group | Treatment Group | Total study participants (n) | Participants (n) Reporting Urticaria or Angioedema Up to 28 Days After Vaccination | Rate (%) | Participants (n) Reporting Urticaria or Angioedema Up to Data Cut Off Date or End of Study | Rate (%) |
| --- | --- | --- | --- | --- | --- | --- |
| Primary Vaccination Studies | mRNA | 69105 | 143 | 0.21 | 244 | 0.35 |
|  | Control | 52555 | 70 | 0.13 | 140 | 0.27 |
| Booster Vaccination Studies | mRNA | 47239 | 68 | 0.14 | 142 | 0.30 |

**Table S29. Rate of urticaria or angioedema lasting <6 weeks or ≥6 weeks across Moderna interventional mRNA studies.** The rates reported here were computed from the pooled studies in tables S25 and S26 using the search criteria in table S27. Adverse events were included regardless of their relationship to study vaccination.

| Pooled Group | Treatment Group | Total study participants (n) | Participants (n) Reporting Urticaria or Angioedema Lasting <6 Weeks | Rate (%) | Participants (n) Reporting Urticaria or Angioedema Lasting ≥6 Weeks | Rate (%) |
| --- | --- | --- | --- | --- | --- | --- |
| Primary Vaccination Studies | mRNA | 69105 | 205 | 0.30 | 37 | 0.05 |
|  | Control | 52555 | 111 | 0.21 | 27 | 0.05 |
| Booster Vaccination Studies | mRNA | 47239 | 102 | 0.22 | 33 | 0.07 |

Participants with event still ongoing and duration <6 weeks by the Data Cut Off Date or End of Study (DCO/EoS) are considered as missing data for duration. The numbers of participants with such missing data are as follows: mRNA primary vaccination studies (mRNA, n=2; control, n=2) and booster vaccination studies (mRNA, n=7).

**Table S30. Rate of urticaria or angioedema versus number of prior Moderna COVID-19 doses, for the NextCOVE (mRNA-1283 P301) trial evaluating Moderna COVID-19 boosters mRNA-1273 and mRNA-1283.** The rates reported here were computed from the mRNA-1283 P301 study using search criteria in table S27. Adverse events were included regardless of their relationship to study vaccination.

| Number of Moderna COVID-19 vaccine doses prior to enrollment in NextCOVE study^†^ | Number of Moderna COVID-19 vaccine doses after vaccination in NextCOVE study^†^ | Study participants (n) | Participants (n) reporting urticaria or angioedema up to data cutoff or end of study | Rate (%) |
| --- | --- | --- | --- | --- |
| 0 to 5  (all participants) | 1 to 6  (all participants) | 11,417 | 12 | 0.11 |
| 0 | 1 | 5,435 | 8 | 0.15 |
| 1 | 2 | 1,923 | 1 | 0.05 |
| 2 | 3 | 896 | 2 | 0.22 |
| 3 | 4 | 1,660 | 0 | 0.00 |
| 4 | 5 | 1,049 | 1 | 0.10 |
| 5 | 6 | 452 | 0 | 0.00 |
| ≥1 | ≥2 | 5,980 | 4 | 0.07 |

^†^One participant did not report a history of COVID-19 vaccination, and one other participant reported ‘NA’ for COVID-19 vaccination history prior to enrollment in the study

**Table S31. Serum IgG binding antibody response rates to eOD-GT8 60mer and related antigens, for recipients of eOD-GT8 60mer mRNA-LNP in G002 and G003 or eOD-GT8 60mer protein and AS01_B_ in G001.** Two-sided Barnard tests at a .05 significance level for pairwise comparisons of response rates between G001, G002 and G003 for weeks 2, 8, 10 and 16 and antigens eOD-GT8 60mer, eOD-GT8.1, eOD-GT8 KO11, eOD-GT8 CD4bs, and Lumazine Synthase, are shown. Response rates with 95 percent CI are displayed. False discovery rate (FDR)-adjusted *P* values (*Q* values) are indicated as “FDR”. For G001, data from low (20 µg) and high (100 µg) dose groups, which had similar serum IgG responses (*15*), were pooled. For G002, data from groups receiving identical treatment at earlier time points were pooled and analyzed together until their vaccine regimens diverged at later time points. G003 was excluded from comparisons involving eOD-GT8 CD4bs and Lumazine Synthase due to insufficient concordance between KAVI and Duke lab assays for those antigens.

**Table S32. Serum IgG binding antibody response magnitudes to eOD-GT8 60mer and related antigens, for recipients of eOD-GT8 60mer mRNA-LNP in G002 and G003 or eOD-GT8 60mer protein and AS01_B_ in G001.** Two-sided Wilcox tests at a .05 significance level for pairwise comparisons of response magnitudes (AUTC and delta AUTC) between G001, G002 and G003 for baseline and weeks 2, 8, 10 and 16 and antigens eOD-GT8 60mer, eOD-GT8.1, eOD-GT8 KO11, eOD-GT8 CD4bs, and Lumazine Synthase, are shown. False discovery rate (FDR)-adjusted *P* values (*Q* values) are indicated as “FDR”. For G001, data from low (20 µg) and high (100 µg) dose groups, which had similar serum IgG responses (*15*), were pooled. For G002, data from groups receiving identical treatment at earlier time points were pooled and analyzed together until their vaccine regimens diverged at later time points. G003 was excluded from comparisons involving eOD-GT8 CD4bs and Lumazine Synthase due to insufficient concordance between KAVI and Duke lab assays for those antigens.

**Table S33. Week 2 (post first vaccination) vs. week 10 (two weeks post second vaccination) comparisons of** **serum IgG binding antibody response rates to eOD-GT8 60mer and related antigens, for recipients of eOD-GT8 60mer mRNA-LNP in G002 and G003 or eOD-GT8 60mer protein and AS01_B_ in G001.** Two-sided McNemar’s tests at a .05 significance level for pairwise comparisons of week 2 and week 10 response rates for G001, G002 and G003 for antigens eOD-GT8 60mer, eOD-GT8.1, eOD-GT8 KO11, eOD-GT8 CD4bs, and Lumazine Synthase, are shown. False discovery rate (FDR)-adjusted *P* values (*Q* values) are indicated as “FDR”. For G001, data from low (20 µg) and high (100 µg) dose groups, which had similar serum IgG responses (*15*), were pooled. For G002, data from groups receiving identical treatment at earlier time points were pooled and analyzed together until their vaccine regimens diverged at later time points.

**Table S34. Week 2 (post first vaccination) vs. week 10 (two weeks post second vaccination) comparisons of** **serum IgG binding antibody magnitudes to eOD-GT8 60mer and related antigens, for recipients of eOD-GT8 60mer mRNA-LNP in G002 and G003 or eOD-GT8 60mer protein and AS01_B_ in G001.** Two-sided Wilcox signed-rank tests at a .05 significance level for pairwise comparisons of week 2 and week 10 response magnitudes (AUTC and delta AUTC) for G001, G002 and G003 for antigens eOD-GT8 60mer, eOD-GT8.1, eOD-GT8 KO11, eOD-GT8 CD4bs, and Lumazine Synthase, are shown. False discovery rate (FDR)-adjusted *P* values (*Q* values) are indicated as “FDR”. For G001, data from low (20 µg) and high (100 µg) dose groups, which had similar serum IgG responses (*15*), were pooled. For G002, data from groups receiving identical treatment at earlier time points were pooled and analyzed together until their vaccine regimens diverged at later time points.

**Table S35. Serum IgG binding antibody response rate comparisons between different heterologous prime-boost regimens at fixed timepoints in G002.** Two-sided Barnard tests at a .05 significance level for comparisons of response rates between G002 groups were made at all timepoints. A false discovery rate (FDR) adjustment was made within pairwise comparisons by antigen in the column FDR.

**Table S36.** **Serum IgG binding antibody magnitude comparisons between different heterologous prime-boost regimens at fixed timepoints in G002.** Two-sided Wilcox tests at a .05 significance level for pairwise comparisons of magnitudes of response between G002 groups were made at all timepoints. A false discovery rate (FDR) adjustment was made within pairwise comparisons by antigen in the column FDR.

**Table S37. G002 numbers of cells sorted from the different groups and timepoints.** PBMC sampling timepoints, the number of PBMC processed at each of the PBMC sampling timepoints and the probesets used for processing PBMC from each of the PBMC sampling timepoints are shown.

|  |  | **Study Week** | | | | | | | | | | |
| --- | --- | --- | --- | --- | --- | --- | --- | --- | --- | --- | --- | --- |
| **Group** | **Sample Type** | **-5** | **0** | **3** | **4** | **7.5** | **8** | **11** | **15.5** | **16** | **19** | **24** |
| **1** | **PBMC** | **eOD-GT8** | **eOD-GT8 vaccination** |  | **eOD-GT8** | **eOD-GT8** | **eOD-GT8 vaccination** |  |  | **eOD-GT8** & **Core-g28v2** |  | **eOD-GT8** & **Core-g28v2** |
| **2** | **PBMC** | **eOD-GT8** | **eOD-GT8 vaccination** |  | **eOD-GT8** | **eOD-GT8** & **Core-g28v2** | **Core-g28v2 vaccination** |  |  | **Core-g28v2** |  | **Core-g28v2** |
| **3** | **PBMC** | **eOD-GT8** | **eOD-GT8 vaccination** |  | **eOD-GT8** | **eOD-GT8** | **eOD-GT8 vaccination** |  | **eOD-GT8** & **Core-g28v2** | **Core-g28v2 vaccination** |  | **Core-g28v2** |
| **4** | **PBMC** | **Core-g28v2** | **Core-g28v2 vaccination** |  | **Core-g28v2** |  | **Core-g28v2** |  |  |  |  |  |
|  | **Cell numbers sorted** |  |  |  |  |  |  |  |  |  |  |  |
|  | **300 million** |  |  |  |  |  |  |  |  |  |  |  |
|  | **200 million** |  |  |  |  |  |  |  |  |  |  |  |
|  | **100 million** |  |  |  |  |  |  |  |  |  |  |  |

**Table S38. G002 flow cytometry phenotyping panel.**

The different markers included in the flow cytometry phenotyping panel are listed.

| **Marker** | **Clone** | **Vendor** | **Catalog No.** |
| --- | --- | --- | --- |
| IgM | G20-127 | BD-custom | NA |
| CD11c | bly6 | BD-custom | NA |
| CD21 | bly4 | BD | 563474 |
| CD85J | GH1/75 | Biolegend | 333712 |
| IgA | IS11-8E10 | Miltenyi | 130-113-472 |
| CD20 | 2H7 | Biolegend | 302358 |
| IgG | G18-145 | BD | 564229 |
| CD38 | HIT2 | BD | 612969 |
| CD62L | DREG-56 | BD | 742024 |
| CD3 | OKT3 | Biolegend | 317332 |
| CD14 | NCAM16.2 | Biolegend | 301842 |
| CD16 | 3G8 | Biolegend | 302048 |
| CD56 | M5E2 | Biolegend | 563041 |
| IgD | IA6-2 | BD | Custom |
| CD27 | O323 | Biolegend | 302830 |
| CD71 | CY1G4 | Biolegend | 334116 |
| CD19 | SJ25C1 | BD | 747161 |
| FCRL5 | 509F6 | BD | 749602 |

**Table S39. G003 flow cytometry phenotyping panel.**

| **Marker** | **Clone** | **Vendor** | **Catalogue No.** |
| --- | --- | --- | --- |
| CD3 | IA6-2 | BD | 317342 |
| CD14 | M5E2 | BD | 561384 |
| CD16 | 3G8 | BD | 560195 |
| CD56 | 5.1H11 | Biolegend | 362512 |
| CD19 | HIB19 (RUO) | BD | 555413 |
| IgD | IA6-2 | BD | 561315 |
| IgM | MHM-88 | Biolegend | 314532 |
| IgG | G18-145 | BD | 564230 |

**Table S40. Statistical testing of differences in fig. S19 where key B cell analysis workflows for the three trials were applied to control samples.** The data panels analyzed correspond to: percentage of IgG memory B cells that are eOD-GT8–specific (GT8^++^) [(A) and (F)], percentage of IgG memory B cells that are eOD-GT8 CD4bs-specific [(B) and (G)]; percentage of VRC01-class IgG B cells among IgG memory B cells (C); VRC01-class BCR V_H_ percent amino acid (aa) mutation (D), and VRC01-class BCR V_K/L_ percent amino acid (aa) mutation (E). Testing was done using a paired T test with FDR adjustment done across panels within a comparison of methodology (e.g. G001 vs. G002). The mean and 95% CI are on the fold change scale for panels A, B, C, F and G and difference scale for the panels D and E. P values less than 0.05 and Q values less than 0.2 are highlighted.

**Table S41. Number of BCR sequences of any isotype obtained for each sample analyzed by eOD-GT8 or core-g28v2 sorting in G002.**

**Table S42. Number of IgG BCR sequences obtained for each sample analyzed by eOD-GT8 or core-g28v2 sorting in G002.**

**Table S43. Number of eOD-GT8 CD4bs-specific BCR sequences of any isotype obtained per participant in G003.**

**
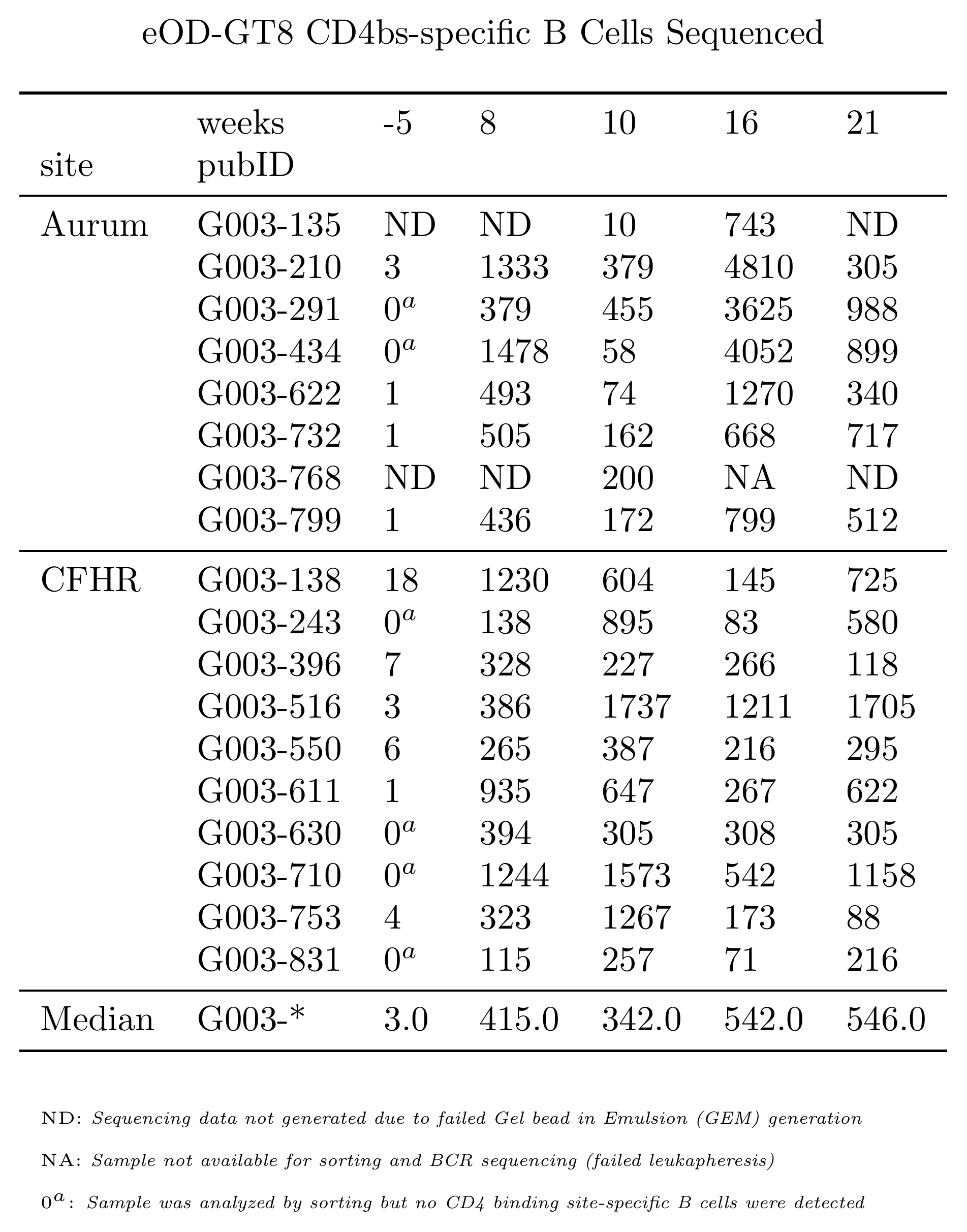
**

**Table S44. Number of eOD-GT8 CD4bs-specific IgG BCR sequences obtained per participant in G003.**

**
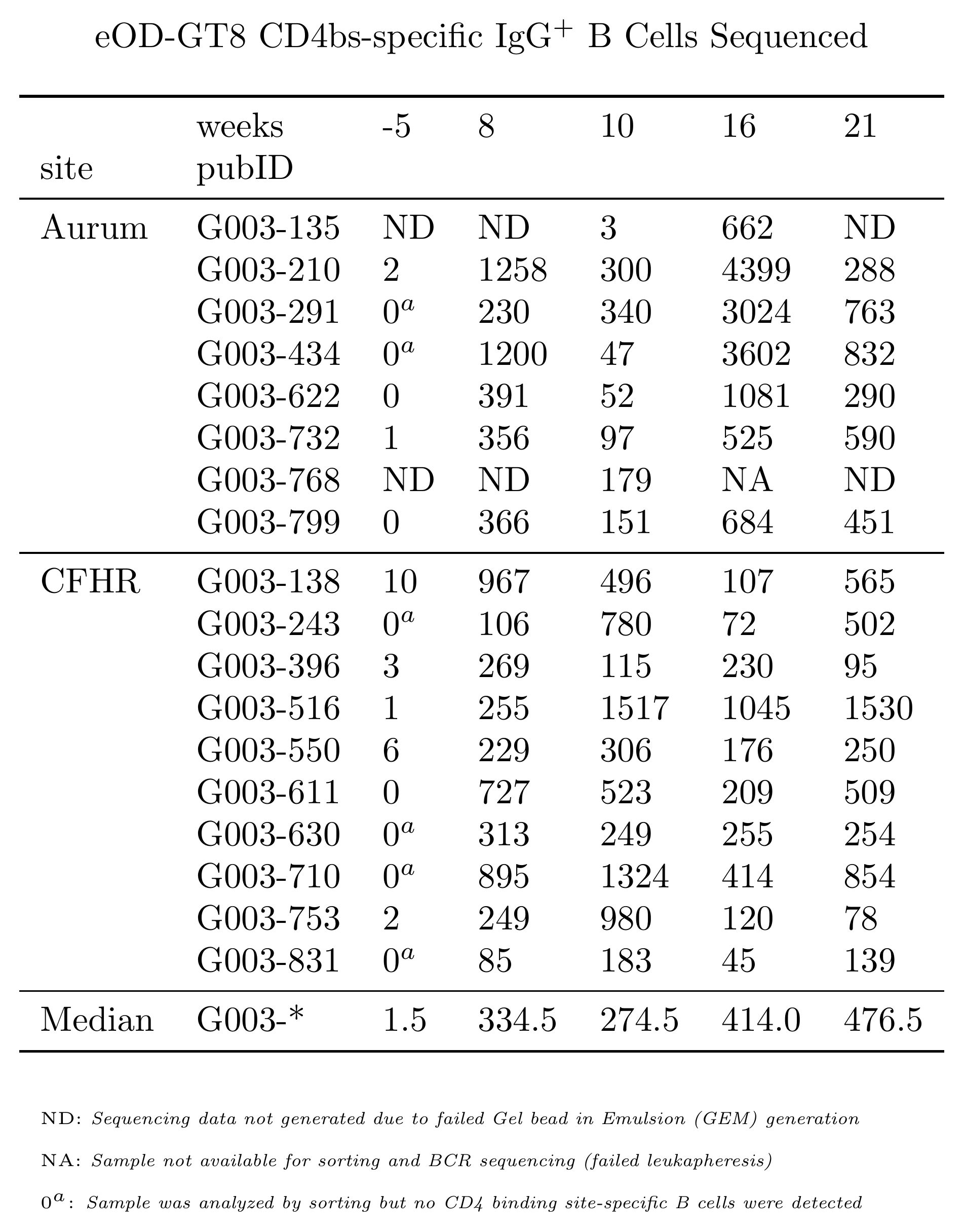
**

**Table S45. IgG B cell response rates to eOD-GT8 and eOD-GT8 CD4bs, for recipients of eOD-GT8 60mer mRNA-LNP in G002 and G003 or eOD-GT8 60mer protein and AS01_B_ in G001.** The analysis corresponds to Fig. 2A (%eOD-GT8^++^ among IgG^+^ B cells) and Fig. 2B (%eOD-GT8 CD4bs-specific among IgG^+^ B cells). Wilson score confidence intervals are indicated.

**
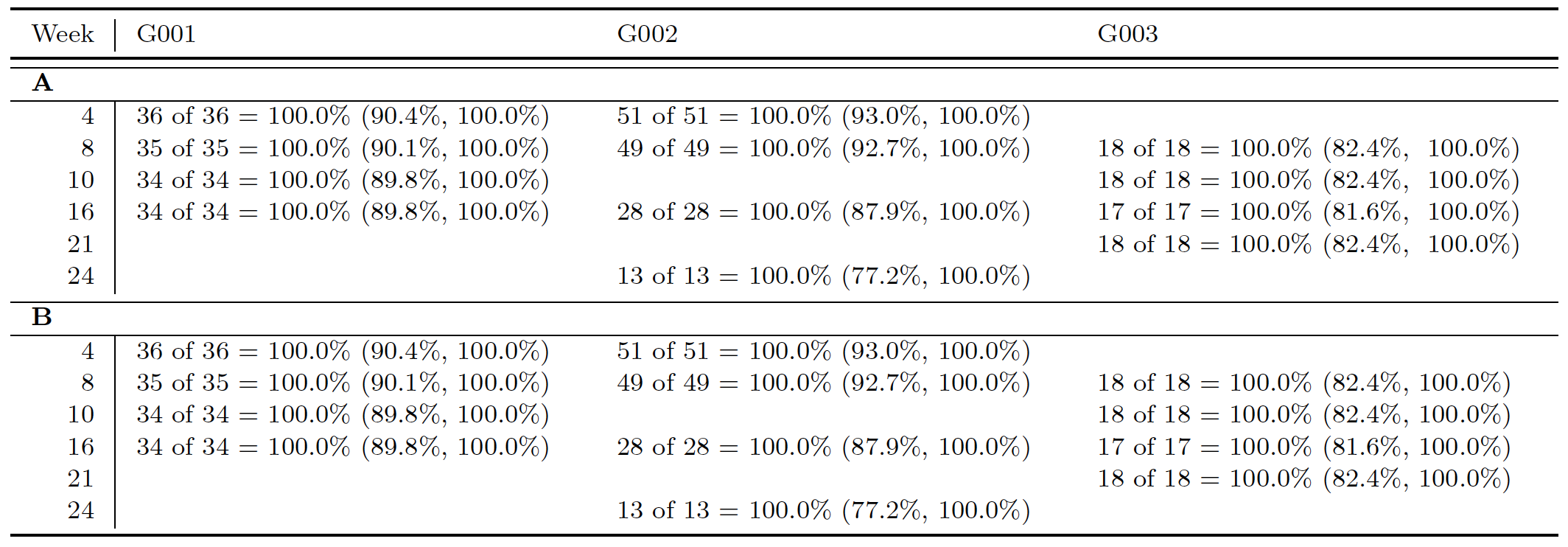
**

**Table S46. IgG B cell response magnitudes compared to baseline, for B cell quantities in Fig. 2A-C and E, for recipients of eOD-GT8 60mer mRNA-LNP in G002 and G003 or eOD-GT8 60mer protein and AS01_B_ in G001.** The analysis corresponds to Fig. 2A (%eOD-GT8^++^ among IgG^+^ B cells), Fig. 2B (%eOD-GT8 CD4bs-specific among IgG^+^ B cells), Fig. 2C (%KO^-^ among eOD-GT8^++^ IgG^+^ B cells), and Fig. 2E (%VRC01-class among IgG^+^ B cells). Testing was done using Wilcoxon signed-rank test for paired data. False discovery rate (FDR)-adjusted *P* values (*Q* values) are indicated as “FDR”. P values less than 0.05 and Q values less than 0.2 are highlighted.

**Table S47. IgG B cell response magnitudes compared between non-baseline timepoints, for B cell quantities in Fig. 2A-C and E-F, for recipients of eOD-GT8 60mer mRNA-LNP in G002 and G003 or eOD-GT8 60mer protein and AS01_B_ in G001.** The analysis corresponds to Fig. 2A (%eOD-GT8^++^ among IgG^+^ B cells), Fig. 2B (%eOD-GT8 CD4bs-specific among IgG^+^ B cells), Fig. 2C (%KO^-^ among eOD-GT8^++^ IgG^+^ B cells), Fig. 2E (%VRC01-class among IgG^+^ B cells), Fig. 2F (%VRC01-class among eOD-GT8 CD4bs-specific IgG B cells), and Fig. 2G (%VRC01-class among eOD-GT8-specific IgG B cells). Testing was done using Wilcoxon signed-rank test for paired data. False discovery rate (FDR)-adjusted *P* values (*Q* values) are indicated as “FDR”. P values less than 0.05 and Q values less than 0.2 are highlighted.

**Table S48. Comparisons for Fig. 2E.** Comparisons were made between G001 and G002 for the frequency of VRC01-class B cells among IgG+ B cells. Difference is the geometric mean ratio for G002 relative to G001 (analysis on log-transformed data), adjusted for the quantitative differences in methods used in the two trials identified in fig. S19 and table S40. UCI and LCI give the 95% CI about the difference. False discovery rate (FDR)-adjusted *P* values (*Q* values) are indicated as “FDR”. P values less than 0.05 and Q values less than 0.2 are highlighted.

**
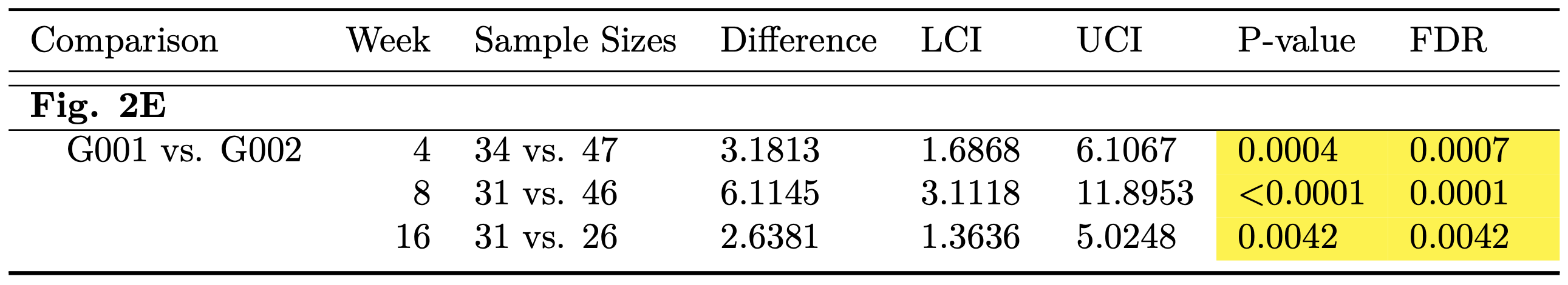
**

**Table S49. G002 IGHV1-2 genotypes and detection of VRC01-class B cells of any isotype by participant.**

**Table S50. G002 IGHV1-2 genotypes and detection of VRC01-class IgG B cells by participant.**

**Table S51. Number of eOD-GT8 CD4bs-specific VRC01-class BCR sequences of any isotype obtained per participant in G003.**

**
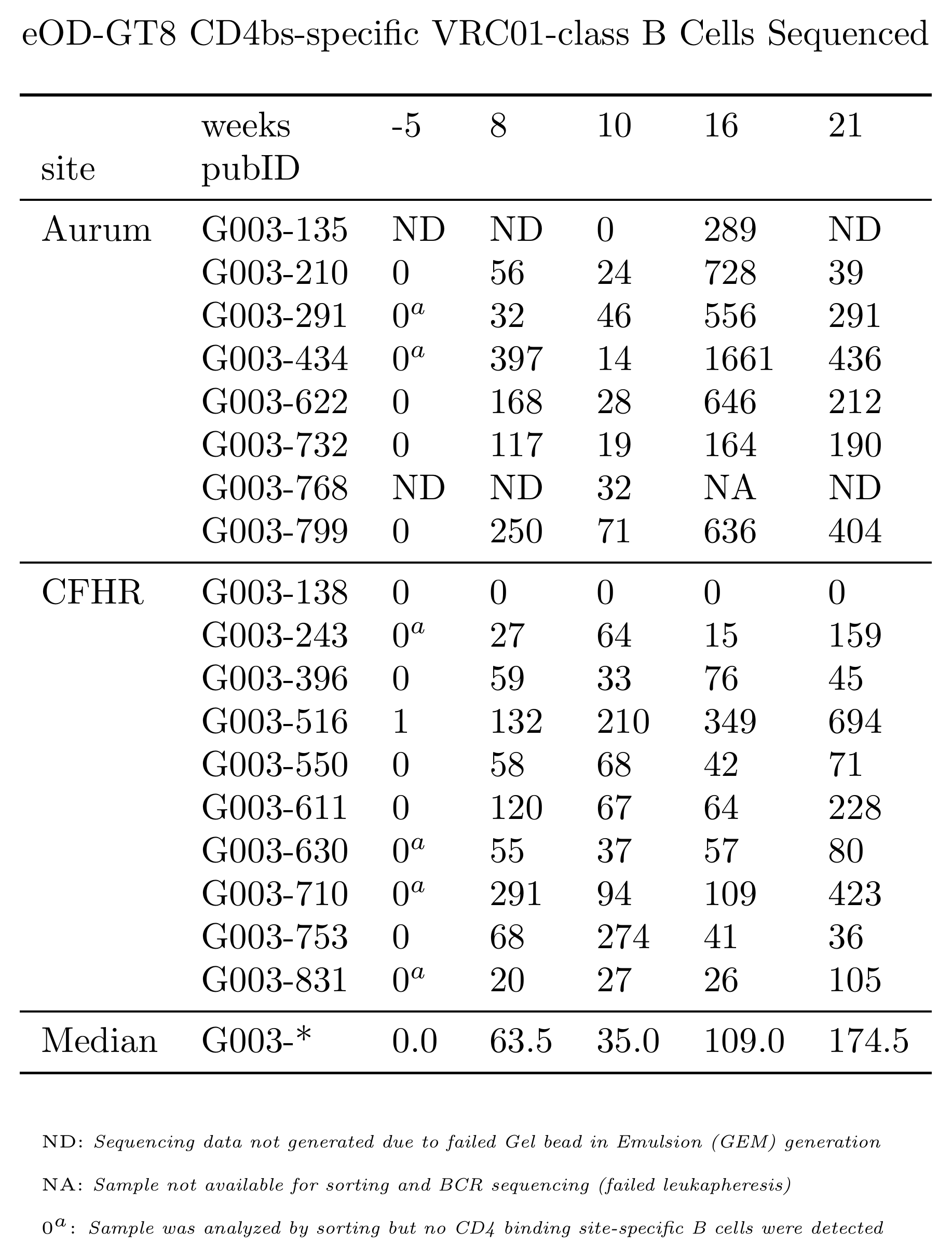
**

**Table S52. Number of eOD-GT8 CD4bs-specific VRC01-class IgG BCR sequences obtained per participant in G003.**

**
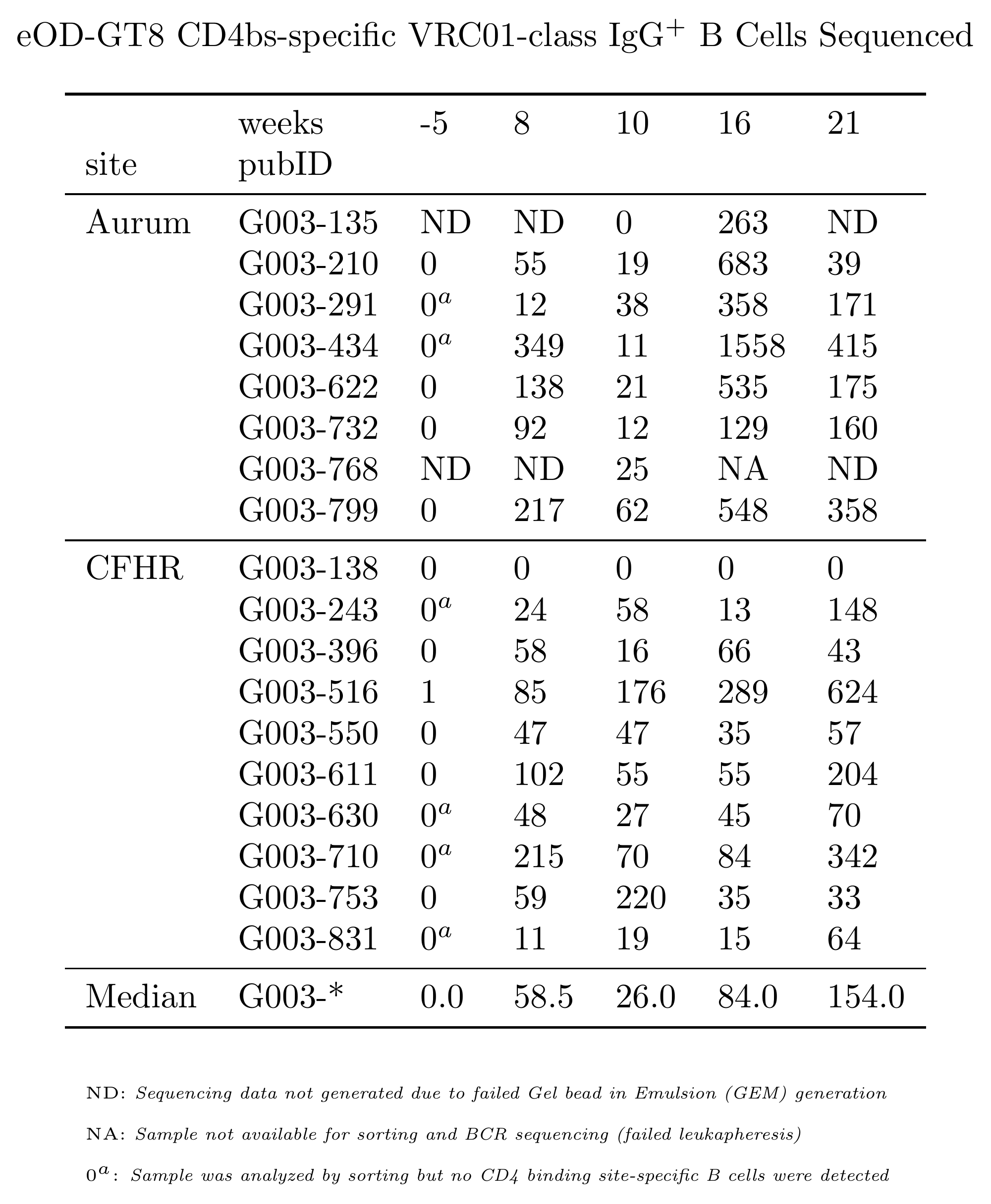
**

**Table S53. Comparisons for Fig. 3, A and B.** Comparisons were made between G001 and G002 for the percentage VH mutation (aa) (Fig. 3A), and the percentage VK/L mutation (aa) (Fig. 3B). Difference is the mean difference of G002 minus G001 for Figs. 3A and 3B, adjusted for the quantitative differences in methods used in the two trials identified in fig. S19 and table S40. UCI and LCI give the 95% CI about the difference. False discovery rate (FDR)-adjusted *P* values (*Q* values) are indicated as “FDR”. P values less than 0.05 and Q values less than 0.2 are highlighted.

**
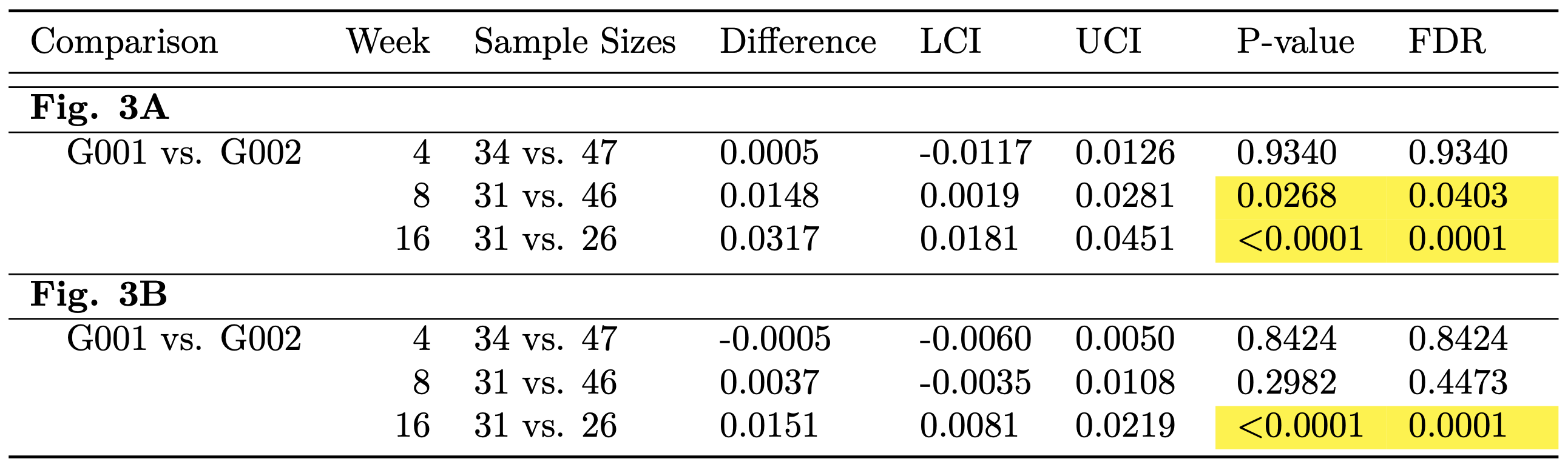
**

**Table S54. Statistical comparisons of percent nucleotide SHM after immunization with eOD-GT8 60mer in G001 vs. G002, related to fig. S25.** Testing between groups was done using the Wilcoxon rank-sum test for unpaired data. No adjustment was made for the difference in methods for measuring outcomes across trials. P values less than 0.05 and Q values less than 0.2 are highlighted.

**Table S55. Comparisons of amino acid SHM measures over time for recipients of eOD-GT8 60mer mRNA-LNP in G002 and G003 or eOD-GT8 60mer protein and AS01_B_ in G001, related to Fig. 3.** Magnitude testing was performed between non-baseline timepoints for each trial for (A) Percent mutation of VH gene (aa), (B) Percent mutation of VK/L gene (aa), and (E) 90th percentile of number of key VRC01-class HC residues. Testing was done using Wilcoxon signed-rank test for paired data. False discovery rate (FDR)-adjusted *P* values (*Q* values) are indicated as “FDR”. P values less than 0.05 and Q values less than 0.2 are highlighted.

**Table S56. Comparisons of nucleotide SHM over time for recipients of eOD-GT8 60mer mRNA-LNP in G002 and G003 or eOD-GT8 60mer protein and AS01_B_ in G001, related to fig. S25.** Magnitude testing was performed between non-baseline timepoints for each trial for (A) Percent mutation of VH gene (aa) and (B) Percent mutation of VK/L gene (aa). Testing was done using Wilcoxon signed-rank test for paired data. False discovery rate (FDR)-adjusted *P* values (*Q* values) are indicated as “FDR”. P values less than 0.05 and Q values less than 0.2 are highlighted.

**Table S57. Statistical comparisons for Fig. 4.** Testing between independent groups was done using the Wilcoxon rank-sum test for continuous outcomes (all panels except E) and Barnard’s exact test for the binary outcome in panel E. Comparisons across time within a single regimen use a Wilcoxon signed-rank test (all panels except E) and McNemar’s test (panel E; the P-value is NA for comparisons at weeks 16 vs. 24 when the responses are perfectly concordant). Summary gives group values for the median and range for continuous outcomes and counts of positive responses out of the total with response rate and 95% Wilson confidence interval for the binary outcome of panel E. P values less than 0.05 and Q values less than 0.2 are highlighted.

**Table S58. Comparisons of the percentage VRC01-class among CD4b-specific and antigen-specific responses, for regimens with B cells sorted by core-g28v2 vs sorted by eOD-GT8, related to Figs. 2, F and G, and 4, G and H.** Testing between groups was done using the Wilcoxon rank-sum test for unpaired data or a Wilcoxon signed-rank test for paired data. Paired data lists one sample size otherwise the data are unpaired. P values less than 0.05 and Q values less than 0.2 are highlighted. For VRC01-class among CD4bs-specific IgG B cells, comparisons correspond to Fig. 4G (sorted with core-g28v2 probes) vs. Fig. 2F (sorted with eOD-GT8 probes). For VRC01-class among antigen-specific IgG B cells, comparisons correspond to Fig. 4H (sorted with core-g28v2 probes) vs. Fig. 2G (sorted with eOD-GT8 probes).

**Table S59. Comparisons of polyclonality and number of unique clones for different G002 regimens at fixed timepoints, related to fig. S35.** Testing between groups was done using the Wilcoxon rank-sum test for unpaired data or a signed rank test for paired data. Sample sizes with two numbers are unpaired data and one number if paired. P values less than 0.05 and Q values less than 0.2 are highlighted.

**Table S60. Comparisons of polyclonality and number of unique clones for different G002 regimens at different timepoints, related to fig. S35.** Testing between groups was done using the Wilcoxon rank-sum test. P values less than 0.05 and Q values less than 0.2 are highlighted.

**Table S61. Statistical comparisons for Fig. 5.** Testing was performed for: **(A)** V_H_ gene % mutation (aa); **(B)** V_K/L_ gene % mutation (aa); **(E)** 90^th^ percentile number of key VRC01-class heavy chain (HC) residues; and **(F)** 90^th^ percentile number of key VRC01-class HCDR2 residues. Testing between independent groups was done using the Wilcoxon rank-sum test for continuous outcomes. Testing across time was done using the Wilcoxon signed-rank test for continuous outcomes and paired data except for the eOD®core at wk16 vs. eOD®eOD®core at wk24 comparison which used the Wilcoxon rank-sum test for independent continous outcomes. P values less than 0.05 and Q values less than 0.2 are highlighted.

**Table S62. Statistical comparisons of VRC01-class sequence metrics for different G002 prime-boost regimens, related to fig. S40.** Testing between groups was done using the Wilcoxon rank-sum test. P values less than 0.05 and Q values less than 0.2 are highlighted.

**Table S63.** Comparisons for Fig. 6 (SPR data). Testing between groups was done using a marginal mean model fit with generalized estimating equations (GEE). Difference is the geometric mean ratio of Group 2 versus Group 1 with the lower (LCI) and upper 95% confidence interval (UCI). Analyses are conditional on *K*_D_ less than 100μM. P values less than 0.05 and Q values less than 0.2 are highlighted.

**Table S64. Comparisons for fig. S48 (SPR data on N276-lacking trimers binding to “selected” mAbs from different prime-boost groups in G002).** Testing between groups was done using a marginal mean model fit with generalized estimating equations (GEE). Difference is the geometric mean ratio of Group 2 versus Group 1 with the lower (LCI) and upper 95% confidence interval (UCI). Analyses are conditional on *K*_D_ less than 50μM. Comparisons resulting in NA do not have any values less than than 50μM in one of the comparator groups. P values less than 0.05 and Q values less than 0.2 are highlighted.

**Table S65. Neutralization IC_50_s for the indicated VRC01-class mAbs against the indicated pseudoviruses.** The data shown are representative of two independent assays. All mAbs in this figure correspond to B cells sorted with core-g28v2 probes. Source data can be found in Data S10.

**Table S66. Statistical testing of neutralization of N276-lacking pseudoviruses.** Testing between groups was done using a marginal mean model fit with generalized estimating equations (GEE). Difference is the geometric mean IC_50_ of Group 2 versus Group 1 with the lower (LCI) and upper 95% confidence interval (UCI). IC_50_ values less than the limit of detection (10 or 50 μg/mL) are imputed as the limit of detection for analysis. P values less than 0.05 and Q values less than 0.2 are highlighted.

**
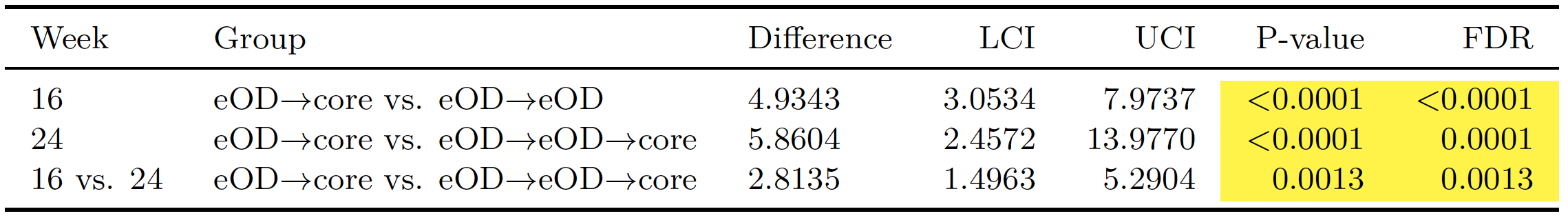
**

**Table S67. Statistical comparisons for Fig. 7.** Testing between groups was done using Barnard’s exact test for the binary outcome in panels B and F and the Wilcoxon rank-sum test for the continuous outcomes in the other panels. Summary gives counts of positive responses out of the total with response rate and 95% Wilson confidence interval for the binary outcomes of panels B and F and group values for the median and range for continuous outcomes in the other panels. P values less than 0.05 and Q values less than 0.2 are highlighted.

**Table S68. Comparisons in Fig. 4 and 6 related to conditions for the core-g28v2 60mer mRNA-LNP boost at week 8 after eOD versus at week 16 after eOD®eOD.** Testing between independent groups was done using: (i) the Wilcoxon rank-sum test for continuous outcomes in Fig. 4D; (ii) Barnard’s exact test for the binary outcome in Fig. 4E; and (iii) a marginal mean model fit with generalized estimating equations (GEE) for the binding *K*_D_ in Fig. 6A. Summary gives group values for the median and range for continuous outcomes in Fig. 4D; counts of positive responses out of the total with response rate and 95% Wilson confidence interval for the binary outcome of Fig. 4E; and the geometric mean ratio of week 16 after eOD®eOD versus week 8 after eOD with the lower and upper 95% confidence interval for the analyses for Fig. 6A. Analyses for Fig. 6A were conditional on *K*_D_ less than 100μM. P values less than 0.05 and Q values less than 0.2 are highlighted.

**Table S69. Comparisons of serum binding antibody response rates to the indicated antigens at week 8 after eOD versus at week 16 after eOD®eOD**. Two-sided McNemar tests at a .05 significance level for pairwise comparisons of week 8 and week 16 response rates are shown for antigens core-g28v2 60mer, core-g28v2 monomer, core-g28v2 KO11b monomer, core-g28v2 CD4bs and Lumazine Synthase.

**Table S70. Comparisons of serum binding antibody response magnitudes to the indicated antigens at week 8 after eOD versus at week 16 after eOD®eOD**. Two-sided Wilcox signed-rank tests at a .05 significance level for pairwise comparisons of week 8 and week 16 response magnitudes (AUTC and delta AUTC) for pooled G002 groups for antigens core-g28v2 60mer, core-g28v2 monomer, core-g28v2 KO11b monomer, core-g28v2 CD4bs and Lumazine Synthase.

**Table S71. Comparisons related to the fold-change in core-g28v2-specific IgG B cells (Fig. 4A), core-g28v2 CD4bs-specific IgG B cells (Fig. 4B), and core-g28v2 CD4bs-specific VRC01-class IgG B cells (Fig. 4D) after the core-g28v2 60mer boost in Group 2 (eOD®core) and Group 3 (eOD®eOD®core).** Comparison of Group 2 week 8 vs. week 16 responses and Group 3 week 16 vs. week 24 responses used a Wilcoxon signed-rank test. Comparison of Group 2 fold-change of week 16 responses over week 8 responses to Group 3 fold-change of week 24 responses over week 16 responses used a Wilcoxon rank-sum test. Note, fold-change analysis was conditional on a defined fold-change (i.e., denominator greater than zero). Summary gives group values for the median and range. P values less than 0.05 and Q values less than 0.2 are highlighted.

**Table S72. Percent of eOD→core participants with at least a 3-fold increase in serum IgG binding to next-stage trimer booster candidates and control antigens at week 8, 16, or 24 compared to baseline.** A modified positivity call was implemented that was more sensitive than typically employed for other studies where optimal specificity is the goal. Response counts and percentages relate to changes in AUTC unless noted as Delta AUTC.

| Antigen | Week 8 | Week 16 | Week 24 |
| --- | --- | --- | --- |
|  |  | N (%) | N (%) |
| 001428-T278M |  | 12 (85.7) | 14 (87.5) |
| 001428-T278M-KO |  | 0 (0.0) | 0 (0.0) |
| 001428-T278M Delta AUTC (CD4bs) |  | 9 (64.3) | 10 (62.5) |
| BGHxB2-T278M |  | 12 (85.7) | 14 (87.5) |
| BGHxB2-T278M-KO |  | 0 (0.0) | 0 (0.0) |
| BGHxB2-T278M Delta AUTC (CD4bs) |  | 12 (85.7) | 14 (87.5) |
| CNE40-T278M |  | 0 (0.0) | 2 (12.5) |
| CNE40-T278M-KO |  | 0 (0.0) | 0 (0.0) |
| CNE40-T278M Delta AUTC (CD4bs) |  | 0 (0.0) | 1 (6.3) |
| V703-0537-T278M |  | 4 (28.6) | 7 (43.8) |
| V703-0537-T278M-KO |  | 0 (0.0) | 0 (0.0) |
| V703-0537-T278M Delta AUTC (CD4bs) |  | 4 (28.6) | 7 (43.8) |
| eOD-GT8 60mer | 16 (100) | 14 (100) |  |
| eOD-GT8 | 16 (100) | 14 (100) |  |
| eOD-GT8 KO11 | 16 (100) | 14 (100) |  |
| eOD-GT8 Delta AUTC (CD4bs) | 16 (100) | 14 (100) |  |
| core-g28v2 60mer | 16 (100) | 14 (100) |  |
| core-g28v2 | 7 (43.8) | 14 (100) |  |
| core-g28v2 KO11 | 2 (12.5) | 14 (100) |  |
| core-g28v2 Delta AUTC (CD4bs) | 7 (43.8) | 14 (100) |  |

**Table S73. Cryo-EM data collection, refinement and validation statistics.**

|  | G002-293-0536 + 001428_T278M_L14 + RM20A3  (EMD-48575)  (PDB 9MSD) | G002-480-0546 + V703-0537_T278M_L14 + BG18  (EMD-48591) (PDB 9MSY) |
| --- | --- | --- |
| **Data collection and processing** |  |  |
| Microscope | TFS Glacios | TFS Glacios |
| Voltage (keV) | 200 | 200 |
| Camera  Collection mode  Magnification  Pixel size at detector (Å) | TFS Falcon 4i  Counting  190,000x  0.718 | TFS Falcon 4i  Counting  190,000x  0.718 |
| Total electron exposure (e–/Å^2^) | 50.0 | 45.0 |
| Exposure rate (e-/pixel/sec)  Number of EER frames  Defocus range (μm) | 7.224  40  -0.7 to -1.8 | 6.818  40  -0.8 to -1.8 |
| Automation software | EPU | EPU |
| Micrographs collected (no.) | 5,003 | 4,743 |
| Micrographs used (no.) | 4,458 | 3,884 |
| Initial particle images (no.) | 534,235 | 488,374 |
| Final particle images (no.) | 47,344 | 51,495 |
| Symmetry  Map resolution (masked/unmasked Å)  FSC threshold | C3  3.2/3.8  0.143 | C3  3.4/4.1  0.143 |
| Map sharpening *B* factor (Å^2^) | -77.2 | -68.5 |
| Map pixel size (Å) | 1.0052 | 1.0339 |
| Map resolution range (Å) | 2.5-4.5 | 3.0-5.0 |
|  |  |  |
| **Refinement** |  |  |
| Initial model used (PDB code) | AlphaFold 3 | AlphaFold 3 |
| Refinement package | Phenix real space refine | Phenix real space refine |
| Model resolution (Å)  FSC threshold | 3.3  0.5 | 3.6  0.5 |
| EMRinger score | 3.56 | 2.47 |
| CC (mask) | 0.79 | 0.79 |
| Model composition  Non-hydrogen atoms  Protein residues  Ligands | 24,288  3,036  48 | 24,315  2,952  90 |
| Mean *B* factors (Å^2^)  Protein  Ligand | 61.62  77.43 | 97.62  88.44 |
| R.m.s. deviations  Bond lengths (Å)  Bond angles (°) | 0.005  0.909 | 0.005  1.006 |
| Validation  MolProbity score  Clashscore  Poor rotamers (%) | 1.08  0.69  0.57 | 1.48  2.53  0.47 |
| Ramachandran plot  Favored (%)  Allowed (%)  Disallowed (%) | 94.86  5.14  0.00 | 93.20  6.80  0.00 |
| Cβ outliers (%) | 0.00 | 0.00 |
| CaBLAM outliers (%) | 2.16 | 3.99 |
|  |  |  |
|  |  |  |

**Table S74. Sample size considerations for Group 4.**

Upper limit of the 95% confidence interval for the true response rate given 0, 1 or 2 positive responders for group sizes of n=4 to 10, is shown.

**Table S75. PBMC sort gates for G003.**


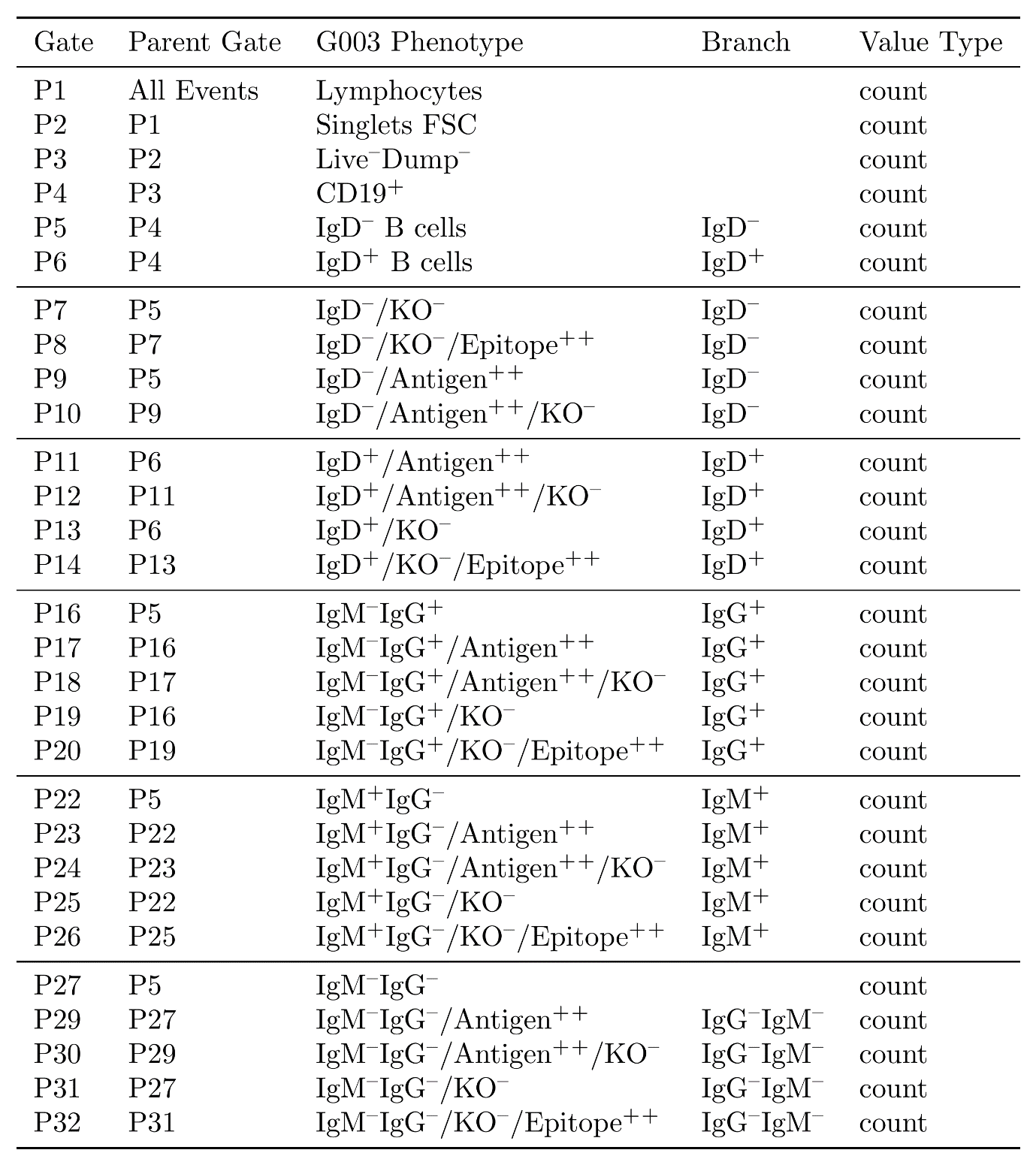


**Table S76. B cell frequencies measured for G003.**

**Table S77.** **Durability of serum IgG antibody binding responses after immunization with eOD-GT8 60mer mRNA-LNP in G002.** Two sided Wilcox signed rank tests at a .05 significance level for pairwise comparisons of week 8 and week 24 response magnitudes (AUTC and delta AUTC) for pooled G002 groups for antigens eOD-GT8 60mer, eOD-GT8.1, eOD-GT8 KO11, eOD-GT8 CD4bs, and Lumazine Synthase.


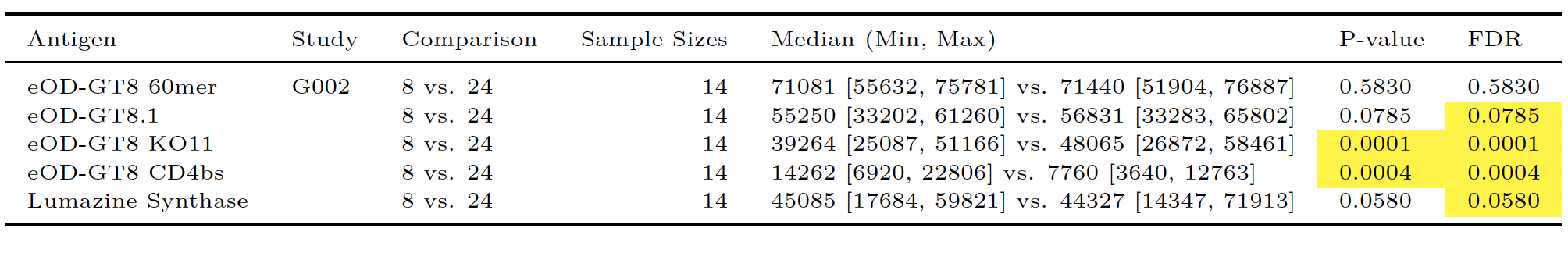


**Table S78.** **Comparisons for Fig. 8A**. Testing between groups was done using GEE. The estimated geometric mean *K*_D_ values in μM for binders (*K*_D_ <50 µM) at weeks 16 and 24 are shown in the GM16 and GM24 columns, respectively. Geometric Mean Ratio, the ratio of the Geometric Mean estimated at Week 24 versus that at Week 16, is shown with the lower (LCI) and upper 95% confidence interval (UCI). Testing was done using GEE conditional on *K*_D_ < 50μM. P values less than 0.05 and Q values less than 0.2 are highlighted.

**Data S1 to S10 are provided as separate spreadsheet files.**

**Data S1.** IAVI G002 visit Day Listing (Randomized Population).

**Data S2.** IAVI G002 local Solicited Adverse Events by Treatment Group and Maximum Reported Severity (Safety Population).

**Data S3.** IAVI G002 systemic Solicited Adverse Events by Treatment Group and Maximum Reported Severity (Safety Population).

**Data S4.** IAVI G002 summary of Unsolicited Adverse Events by MedDRA SOC, PT and Treatment Group (Safety Population)

**Data S5.** IAVI G002 summary of Unsolicited Adverse Events by Treatment Group, Severity and Relationship to Study Treatment (Safety Population).

**Data S6.** IAVI G002 data manifest architectures used for flow data.

**Data S7.** IAVI G002 example merged DataFrame.

**Data S8.** IAVI G002 list of PBMC sort gates.

**Data S9.** IAVI G002 list of B cell frequencies measured.

**Data S10.** Source data for immunology figures and tables. Includes data for Figs. 2 to 8, Table 1, figs. S8 to S10, S19 to S36, S40 to S43, S46 to S48, and S52 to S55, table S65, and all tables showing statistical analyses of the figures listed.

1. [↑](#endnote-ref-2)
2. [↑](#endnote-ref-3)
3. [↑](#endnote-ref-4)
4. [↑](#endnote-ref-5)
5. [↑](#endnote-ref-6)
6. [↑](#endnote-ref-7)
7. [↑](#endnote-ref-8)
8. [↑](#endnote-ref-9)
9. [↑](#endnote-ref-10)
10. [↑](#endnote-ref-11)
11. [↑](#endnote-ref-12)
12. [↑](#endnote-ref-13)
13. [↑](#endnote-ref-14)
14. [↑](#endnote-ref-15)
15. [↑](#endnote-ref-16)
16. [↑](#endnote-ref-17)
17. [↑](#endnote-ref-18)
18. [↑](#endnote-ref-19)
19. [↑](#endnote-ref-20)
20. [↑](#endnote-ref-21)
21. [↑](#endnote-ref-22)
22. [↑](#endnote-ref-23)
23. [↑](#endnote-ref-24)
24. [↑](#endnote-ref-25)
25. [↑](#endnote-ref-26)
26. [↑](#endnote-ref-27)
27. [↑](#endnote-ref-28)
28. [↑](#endnote-ref-29)
29. [↑](#endnote-ref-30)
30. [↑](#endnote-ref-31)
31. [↑](#endnote-ref-32)
